# New Australian sauropods shed light on Cretaceous dinosaur palaeobiogeography

**DOI:** 10.1038/srep34467

**Published:** 2016-10-20

**Authors:** Stephen F. Poropat, Philip D. Mannion, Paul Upchurch, Scott A. Hocknull, Benjamin P. Kear, Martin Kundrát, Travis R. Tischler, Trish Sloan, George H. K. Sinapius, Judy A. Elliott, David A. Elliott

**Affiliations:** 1 Department of Earth Sciences, Uppsala University, Uppsala, Sweden; 2 Australian Age of Dinosaurs Museum of Natural History, The Jump-Up, Winton, Queensland, Australia; 3 Department of Earth Science and Engineering, Imperial College London, South Kensington Campus, London SW7 2AZ, United Kingdom; 4 Department of Earth Sciences, University College London, Gower Street, London WC1E 6BT, United Kingdom; 5 Geosciences, Queensland Museum, Hendra, Queensland, Australia; 6 Museum of Evolution, Uppsala University, Norbyvägen 16, SE-752 36 Uppsala, Sweden; 7 Department of Ecology, Faculty of Natural Sciences, Comenius University, Ilkovicova 6, SK-84215, Bratislava, Slovak Republic; 8 Center for Interdisciplinary Biosciences, Faculty of Science, University of Pavol Jozef Šafárik, Jesenná 5, SK-04154, Košice, Slovak Republic

## Abstract

Australian dinosaurs have played a rare but controversial role in the debate surrounding the effect of Gondwanan break-up on Cretaceous dinosaur distribution. Major spatiotemporal gaps in the Gondwanan Cretaceous fossil record, coupled with taxon incompleteness, have hindered research on this effect, especially in Australia. Here we report on two new sauropod specimens from the early Late Cretaceous of Queensland, Australia, that have important implications for Cretaceous dinosaur palaeobiogeography. *Savannasaurus elliottorum* gen. et sp. nov. comprises one of the most complete Cretaceous sauropod skeletons ever found in Australia, whereas a new specimen of *Diamantinasaurus matildae* includes the first ever cranial remains of an Australian sauropod. The results of a new phylogenetic analysis, in which both *Savannasaurus* and *Diamantinasaurus* are recovered within Titanosauria, were used as the basis for a quantitative palaeobiogeographical analysis of macronarian sauropods. Titanosaurs achieved a worldwide distribution by at least 125 million years ago, suggesting that mid-Cretaceous Australian sauropods represent remnants of clades which were widespread during the Early Cretaceous. These lineages would have entered Australasia via dispersal from South America, presumably across Antarctica. High latitude sauropod dispersal might have been facilitated by Albian–Turonian warming that lifted a palaeoclimatic dispersal barrier between Antarctica and South America.

The effect of the break-up of the Gondwanan supercontinent on the distribution of terrestrial animals during the Cretaceous remains the subject of heated debate^1^, despite marked improvements in the quality of palaeogeographic models^2^. A major limiting factor has been the temporal and spatial coverage of the mid-Cretaceous (~130–90 million years ago [Ma]) terrestrial fossil record^3^. Few dinosaur remains have been recovered from the mid-Cretaceous of Antarctica, Zealandia, or Indo-Madagascar. In contrast, diverse mid-Cretaceous dinosaur faunas have been identified in southwest South America (Patagonia)^4^, northern and southeast Africa^5^, and eastern Australia^6^,^7^. The distribution of dinosaur-bearing strata in the latest Cretaceous (84–66 Ma) is rather different: the African^5^ and Australian^6^ records are effectively non-existent, whereas diverse faunas are known from South America^4^, India^8^, Madagascar^9^ and Antarctica^10^. Of all of the Gondwanan continents, only South America has an adequate dinosaur record spanning virtually the entire Cretaceous period^4^. Accordingly, interpretations of the impact of Gondwanan palaeogeography on dinosaur distribution must account for this.

On the basis of palaeogeographic reconstructions alone, dinosaurs from the mid-Cretaceous of Australia would be expected to be most similar to those from South America and Antarctica. This is because these three continents (along with Zealandia) formed a single contiguous landmass for the majority of the Cretaceous^2^. Intriguingly, this hypothesis has found only limited support from the fossil record^11^,^12^. Fragmentary theropod and ornithischian remains from the late Early Cretaceous of southeast Australia have been interpreted to show close affinities to Laurasian forms by some^13^,^14^, although others have concluded that closer ties to Gondwanan lineages are evident^11^,^12^,^15^. *Muttaburrasaurus* from the late Early Cretaceous of northeast Australia has been resolved either as a rhabdodontid^16^ or a basal iguanodontian^17^ with close ties to European taxa. The more-or-less coeval small ankylosaur *Kunbarrasaurus* (formerly *Minmi* sp.) has been recovered as either the most basal ankylosaurid^18^ or the most basal ankylosaurian^19^; whichever of these interpretations (if either) is correct has significant implications for ankylosaur palaeobiogeography, since Ankylosauridae is otherwise known exclusively from Laurasia, whereas Ankylosauria is represented across both Laurasia and Gondwana^18^,^19^. Australia’s only reasonably complete non-avian theropod, the early Late Cretaceous *Australovenator*, has been resolved as a megaraptoran with close ties to Japanese, Argentinean, and North American taxa^20^,^21^, whereas the contemporary *Diamantinasaurus*, Australia’s most completely known Cretaceous sauropod, has been recovered as a lithostrotian titanosaur with close ties to South American and Asian forms^22^,^23^,^24^.

In sum, the apparent close affinity of many Australian dinosaurs with Laurasian taxa, despite their prolonged geographic separation by the Tethys Ocean^2^, presents a potential palaeobiogeographical conundrum. The only ways to improve assessments of Cretaceous Gondwanan dinosaur palaeobiogeography are to amplify the Gondwanan Cretaceous fossil record, and to utilise more rigorous analytical tools to assess the limited data at hand.

Here we report on, and briefly describe, two new sauropod dinosaur specimens from the Cenomanian–lower Turonian (lower Upper Cretaceous) Winton Formation of Queensland, northeast Australia (Figs 1 and 2). The first of these specimens forms the basis for *Savannasaurus elliottorum* gen. et sp. nov., and comprises one of the most complete sauropod skeletons ever found in Australia. The other specimen is referred to *Diamantinasaurus matildae*^20^,^22^ and includes the first partial sauropod skull identified from the Australian continent^25^. These new data are utilised to provide a revised view of Cretaceous Gondwanan sauropod dinosaur palaeobiogeography.

## Results

### Systematic Palaeontology

Dinosauria Owen, 1842

Saurischia Seeley, 1887

Sauropoda Marsh, 1878

Macronaria Wilson and Sereno, 1998

Titanosauriformes Salgado, Coria and Calvo, 1997

Titanosauria Bonaparte and Coria, 1993

***Savannasaurus elliottorum*** gen. et sp. nov.

### Etymology

From the Spanish (Taino) *zavana* (savanna), in reference to the countryside in which the specimen was found, and the Greek *σαῦρος* (lizard). The species name honours the Elliott family for their ongoing contributions to Australian palaeontology.

### Holotype

**Figure 3:**
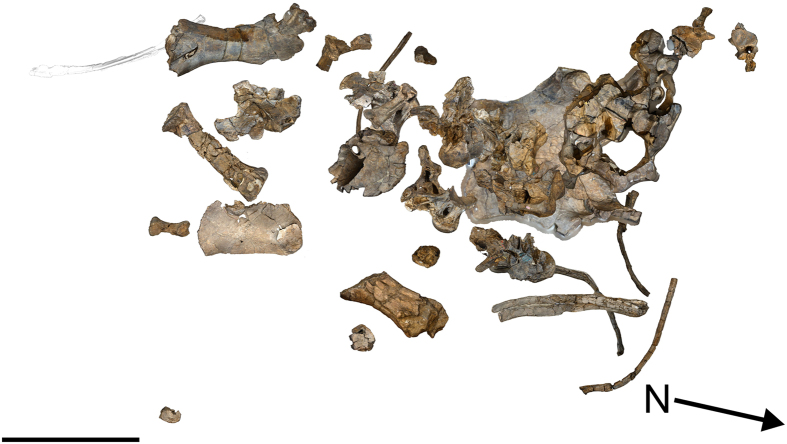
*Savannasaurus elliottorum* gen. et sp. nov., holotype specimen AODF 660. Type site map showing the approximate association of the bones. Scale bar = 1 m.

Australian Age of Dinosaurs Fossil (AODF) 660: one posterior cervical vertebra; several cervical ribs; eight dorsal vertebrae; several dorsal ribs; at least four coalesced sacral vertebrae with processes; at least five partial caudal vertebrae; fragmentary scapula; left coracoid; left and right sternal plates; incomplete left and right humeri; shattered ulna; left radius; right metacarpals I–V; left metacarpal IV; two manual phalanges; fragments of left and right ilia; left and right pubes and ischia, fused together; left astragalus; right metatarsal III; and associated fragments. This disarticulated skeleton was found within a single concretion. The dorsal vertebrae and ribs were in approximate order but were somewhat scattered immediately in front of the incomplete sacrum and puboischiadic sheet (Fig. 3).

### Type horizon and locality

Winton Formation (Cenomanian–lower Turonian^26^); Australian Age of Dinosaurs Locality (AODL) 82 (the “Ho-Hum site”), Belmont Station, Winton, Queensland, Australia.

### Diagnosis

Wide-bodied titanosaur diagnosed by the following autapomorphies: (1) anterior-most caudal centra with shallow lateral pneumatic fossae; (2) sternal plate with straight lateral margin (reversal); (3) metacarpal IV distal end hourglass shaped; (4) pubis with ridge extending anteroventrally from ventral margin of obturator foramen on lateral surface; and (5) astragalus proximodistally taller than mediolaterally wide or anteroposteriorly long.

### Description

**Figure 4:**
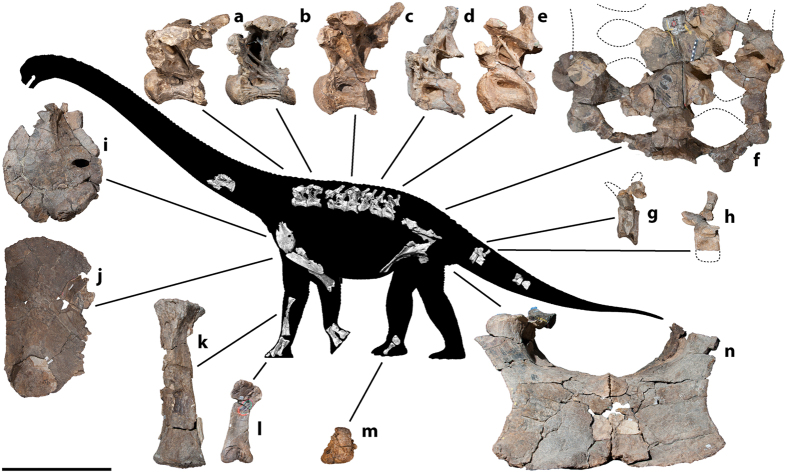
*Savannasaurus elliottorum* gen. et sp. nov., holotype specimen AODF 660. (**a–e**) Dorsal vertebrae (left lateral view). (**f**) Sacrum (ventral view). (**g,h**) Caudal vertebrae (left lateral view). (**i**) Left coracoid (lateral view). (**j**) Right sternal plate (ventral view). (**k**) Left radius (posterior view). (**l**) Right metacarpal III (anterior view). (**m**) Left astragalus (anterior view). (**n**) Coossified right and left pubes (anterior view). A number of ribs were preserved but have been omitted for clarity. Scale bar = 500 mm.

The sole preserved cervical vertebra of *Savannasaurus* is opisthocoelous and possesses a deep lateral pneumatic foramen. It bears a mid-line ventral keel, a feature uncommon among Macronaria^27^. The cervical ribs are elongate, such that they overlap at least two vertebrae additional to the one to which they were attached. All preserved dorsal centra are opisthocoelous and show camellate internal texture as in Titanosauriformes^28^,^29^. They possess deep, posteriorly acuminate, lateral pneumatic foramina that are set within fossae (Fig. 4a–e); the latter characteristic is mainly restricted to somphospondylans^29^. All preserved dorsal vertebrae possess ventrolateral ridges but lack ventral keels; both keels and ridges are present in *Opisthocoelicaudia*^30^ and *Diamantinasaurus*^22^. As in most advanced titanosaurs^27^,^31^, hyposphenes and hypantra are absent in all preserved vertebrae of *Savannasaurus*. The dorsal neural spines are not bifid, distinguishing *Savannasaurus* from *Opisthocoelicaudia*^30^. The dorsal neural spines are angled posterodorsally at 45° to the long axis of the centrum in the anterior-most vertebrae, a synapomorphy of Somphospondyli^29^; this angle decreases along the column, with the posterior-most spines sub-vertical. As in all members of Titanosauriformes^29^, the dorsal ribs bear proximal pneumatic cavities. The incomplete sacrum, comprising at least four vertebrae with lower sacral acetabular processes, is over one metre wide transversely at its narrowest point (Fig. 4f), contributing to the wide-hipped appearance of *Savannasaurus*. All preserved caudal vertebrae are amphicoelous (Fig. 4g,h), distinguishing *Savannasaurus* from most titanosaurs^32^. The anterior-most caudal vertebra preserved bears shallow lateral pneumatic fossae, unlike those of most somphospondylans^27^, including *Wintonotitan*^33^. Within Macronaria, the presence of such fossae has been regarded as a synapomorphy of Brachiosauridae (or a slightly less inclusive clade)^31^; as such, the discovery of fossae in the anterior caudal vertebrae of *Savannasaurus* indicates that this feature was more widespread within Titanosauriformes.

Unlike those of titanosaurs^29^,^32^, the dorsoventrally thin, but transversely broad, sternal plates (Fig. 4j) lack a reniform shape, although each sternal plate is approximately 70% the length of the humerus, a feature shared with other titanosaurs^34^. Relative to the long axis of the shaft, the distal end of the radius is bevelled at ~20° (Fig. 4k), and the mediolateral width of the proximal end of the radius is one-third its overall proximodistal length, characteristic of Titanosauria^35^. As is known for *Diamantinasaurus*^22^, and presumed in *Wintonotitan*^33^, the metacarpals are, from longest to shortest, III-II-I-IV-V, and manual phalanges were present on at least some of the digits. The maximum length of the longest metacarpal (Fig. 4l) is greater than 0.45 times that of the radius (0.49), a synapomorphy of Macronaria^29^, but this value is lower than in both *Diamantinasaurus*^22^ and *Wintonotitan*^33^. The distal condyle of metacarpal I is reduced, as in other Titanosauriformes^29^, and the distal end of metacarpal IV has an autapomorphic hourglass shape.

Both pubes and ischia are fused, forming a sheet-like structure over one metre wide at its narrowest point (Fig. 4n), and less than one centimetre thick at the junction of the four elements. An autapomorphic ridge extends anteroventrally from the ventral margin of the obturator foramen along the lateral surface of the pubis. The posterolateral process of the ischium is less-developed than in *Wintonotitan*^33^. Distally, the ischia are coplanar, and are significantly shorter than the pubes (ratio < 0.8), as in most somphospondylans^27^,^34^. As is typical for Neosauropoda^29^, the astragalus of *Savannasaurus* is wedge-shaped; however, its morphology differs markedly from that of *Diamantinasaurus*^22^ and, indeed other sauropods, in that it is proximodistally taller than either mediolaterally broad or anteroposteriorly long (Fig. 4m).

Titanosauria Bonaparte and Coria, 1993

***Diamantinasaurus matildae*** Hocknull, White, Tischler, Cook, Calleja, Sloan and Elliott, 2009

### Holotype (including paratypes from the same individual [marked with an asterisk])

AODF 603: three partial cervical ribs; two incomplete dorsal vertebrae*; dorsal ribs; four coalesced sacral vertebrae with bases of two sacral processes*; two isolated sacral processes; right scapula; right coracoid*; right and left humeri; right ulna; right radius*; left metacarpal I; right metacarpals II–V; five manual phalanges; left ilium; right and left pubes; right and left ischia; right femur; right tibia; right fibula; right astragalus^20^,^22^.

### Referred specimen

**Figure 5:**
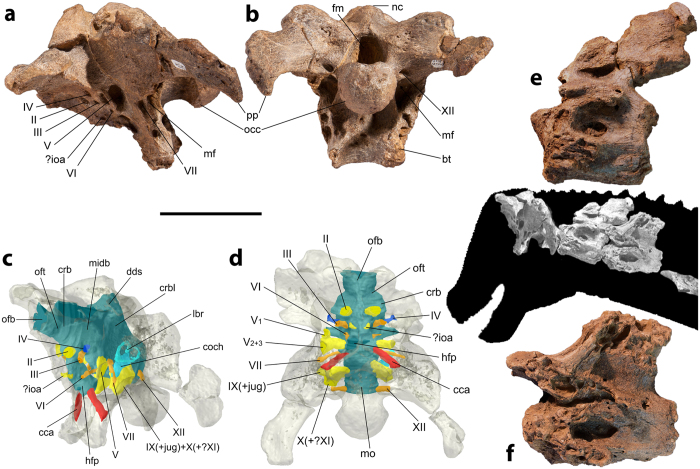
*Diamantinasaurus matildae*, referred specimen AODF 836. (**a**,**b**) Braincase (left lateral and caudal views). (**c**,**d**) endocranium (left lateral oblique and ventral views). (**e**) Axis (left lateral view). (**f**) Cervical vertebra III (left lateral view). Abbreviations: bt, basal tuber; cca, internal carotid artery; coch, cochlea; crb, cerebral hemisphere; crbl, cerebellum; dds, dorsal dural sinus; fm, foramen magnum; hfp, hypophyseal fossa placement; ioa, internal ophthalmic artery; jug, jugular vein; lbr, endosseous labyrinth; mf, metotic foramen; midb, midbrain; mo, medulla oblongata; nc, nuchal crest; occ, occipital condyle; ofb, olfactory bulb; oft, olfactory tract; pp, paroccipital process; II, optic tract; III, oculomotor nerve; IV, trochlear nerve; V, trigeminal nerve; V_1_, ophthalmic branch of the trigeminal nerve; V_2+3_, maxillo-mandibular branch of the trigeminal nerve; VI, abducens nerve; VII, facial nerve; IX, glossopharyngeal nerve; X, vagus nerve; XI, accessory nerve; XII, hypoglossal nerve? structure of unknown or disputable identity/placement. Scale bar = 100 mm.

AODF 836: left squamosal; nearly complete braincase; right surangular; skull fragments; atlas-axis; five post-axial cervical vertebrae; three dorsal vertebrae; partial sacrum; dorsal ribs; right scapula; both iliac preacetabular processes; paired pubes and ischia; associated fragments (Fig. 5).

### Horizon and locality

Winton Formation (Cenomanian–early Turonian^26^); AODL 127 (the “Elliot site”), Belmont Station, Winton, Queensland, Australia.

### Description of AODF 836

The frontal of *Diamantinasaurus* would have formed the anterior margin of the supratemporal fenestra, a feature shared with *Saltasaurus*^36^ and *Rapetosaurus*^37^, but not *Nemegtosaurus*^38^. A posteroventrally directed occipital condyle (Fig. 5a) and the extension of the paroccipital processes as distoventral prongs (Fig. 5b) are both features characteristic of titanosaurs^29^. The dorsoventral height of the supraoccipital is less than that of the foramen magnum, and the basal tubera are greater than 1.5 times the width of the occipital condyle, lacking a raised lip and diverging at less than 50°—these features are shared with saltasaurids (e.g. *Saltasaurus*^36^), but not with nemegtosaurids (e.g. *Nemegtosaurus* and *Rapetosaurus*^38^). The foramen on the posterior surface of the basal tubera is also present in most titanosauriforms, but is absent in *Nemegtosaurus* and *Rapetosaurus*^38^. As is also the case in derived titanosaurs^39^, the opening for cranial nerve VI does not penetrate the pituitary fossa (Fig. 5c). The external foramen for the internal carotid artery lies medial to the basipterygoid process (Fig. 5d), a characteristic only observed in derived titanosaurs^39^.

All preserved postaxial presacral vertebrae are opisthocoelous, and show a camellate internal tissue texture. *Diamantinasaurus* has an anteroposteriorly short axis (Fig. 5e), a feature previously suggested as characterizing Saltasauridae^31^. The prezygapophyses of each preserved anterior cervical vertebra project further anteriorly than the anterior condyle of the centrum (Fig. 5f), distinguishing *Diamantinasaurus* from *Saltasaurus*^36^ and *Rapetosaurus*^37^. As is also the case in the holotype of *Diamantinasaurus*^22^, the dorsal surfaces of the cervical ribs are not excavated. In the middle dorsal vertebrae, the postspinal lamina extends ventral to the neural spine.

**Figure 6:**
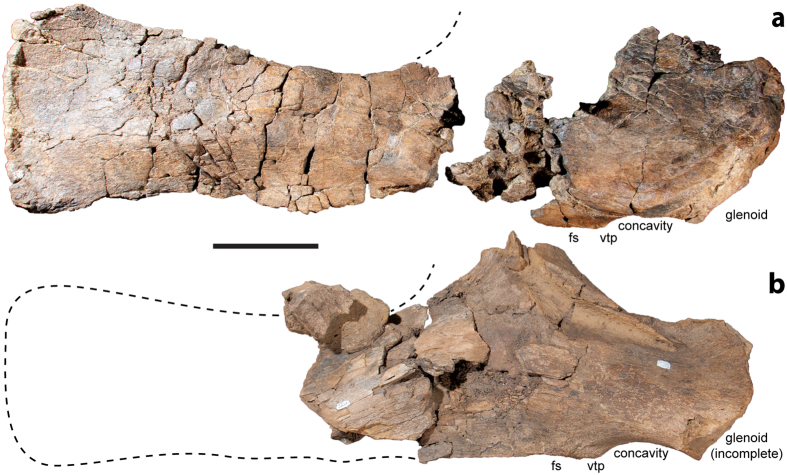
Scapulae of *Diamantinasaurus matildae*. (**a**) *Diamantinasaurus matildae* holotype right scapula AODF 603 (right lateral view). (**b**) *Diamantinasaurus matildae* referred right scapula AODF 836 (right lateral view). Abbreviations: fs, flattened surface; vtp, ventral triangular process. Scale bar = 200 mm for (**a**) and 140 mm for (**b**).

The scapular glenoid is laterally bevelled, and a flattened surface posterior to the ventral triangular process is present (Fig. 6), as in the holotype of *Diamantinasaurus*^22^, but not *Wintonotitan*^33^. No fossa is present on the medial surface of the scapula, and the posterolateral process of the ischium is weak; both of these features distinguish *Diamantinasaurus* from *Wintonotitan*^33^. The pubes and ischia are robust, and the morphology of these elements far more closely approximates those of the *Diamantinasaurus* holotype^33^ than those of the *Savannasaurus* or *Wintonotitan* type specimens^33^.

### Additional comparisons between *Savannasaurus* and *Diamantinasaurus*

The dorsal vertebrae of *Savannasaurus* and *Diamantinasaurus* are quite similar overall, but there are several differences. The type specimen of *Diamantinasaurus* includes two dorsal vertebrae^22^, one posterior (described as “dorsal vertebra A” by Poropat *et al*.^22^) and one anterior (“dorsal vertebra B”). Based on comparisons with *Savannasaurus*, the type anterior dorsal vertebra of *Diamantinasaurus* is Dv3, and its morphology is extremely similar to that of *Savannasaurus*. Both lack ventral keels, and both possess paired posterior centroparapophyseal laminae (PCPLs). However, the centroprezygapophyseal laminae (CPRLs) of *Savannasaurus* are paired, whereas those of *Diamantinasaurus* are not. Ventrally, the middle–posterior dorsal centra of both taxa are transversely concave, between ventrolateral ridges. However, the type posterior dorsal vertebra of *Diamantinasaurus* is quite different from the posterior dorsal vertebrae of *Savannasaurus* inasmuch as it possesses a ventral mid-line keel and has a vertical neural spine. In both taxa, the postspinal lamina extends ventral to the neural spine, beyond the postzygapophyseal articular surfaces.

The forelimbs of *Savannasaurus* are proportionally quite different from those of *Diamantinasaurus*. In *Savannasaurus*, the longest metacarpal (III) is 0.49 times the length of the radius, and the radius is less than 0.75 times the length of the humerus, whereas in *Diamantinasaurus*, the longest metacarpal (III) is 0.61 times the length of the radius, and the radius is 0.63 times the length of the humerus. The maximum diameter of the proximal end of the radius divided by the proximodistal length is 0.3 or greater in both taxa.

Perhaps the most notable differences between the two specimens lie in the pelvic girdle. Whereas the pubis and ischium of *Diamantinasaurus* are slightly proximodistally longer than those of *Savannasaurus*, the mediolateral width of the articulated pubes and ischia of the latter greatly exceeds that of the former (ratio of 1.2–1.4 depending on point of measurement). Thus, *Savannasaurus* must have been proportionally wider across the hips than *Diamantinasaurus*, which is corroborated by measurements of the sacral vertebrae. Both taxa share the presence of an anteriorly expanded ‘boot’^27^ at the distal end of the pubis.

### Phylogenetic results

**Figure 7:**
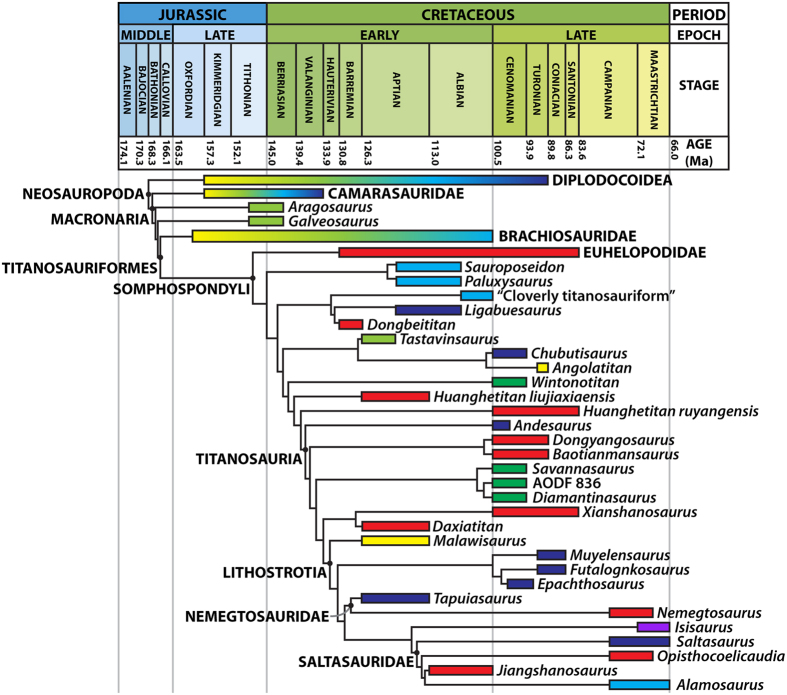
Time-calibrated phylogenetic tree, with basal nodes collapsed for simplicity. (see Supplementary Fig. S2 for full version). The box next to each taxon demarcates its temporal range (including stratigraphic uncertainty), whereas the colour of the box reflects the continent(s) from which the taxon derives (light blue = North America; light green = Europe; red = Asia; dark blue = South America; yellow = Africa; purple = India; dark green = Australia).

Following *a priori* pruning of ten unstable and highly incomplete taxa (see Supplementary Information), our equal weights analysis resulted in 12 MPTs of 1,508 steps and produced a largely resolved strict consensus tree (Supplementary Fig. S1), with polytomies restricted to: (1) a clade within Brachiosauridae; (2) the base of Titanosauria; and (3) several lithostrotian taxa outside of Saltasauridae. The agreement subtree (i.e. the largest fully resolved topology common to all MPTs) required the *a posteriori* pruning of four further taxa (Supplementary Fig. S2) and is shown in Fig. 7 as a time-calibrated phylogenetic tree, with basal nodes collapsed for simplicity. Bremer supports vary from 1 to 3 throughout the tree, with the best supported clades including Euhelopodidae and Lithostrotia. The tree topology is largely congruent with that presented in previous iterations of this data matrix^22^,^23^,^27^,^33^; consequently, we focus on the results pertaining to the Australian taxa.

*Wintonotitan* is recovered as a non-titanosaurian somphospondylan, just basal to the titanosaur radiation (Fig. 7), similar to its position in previous analyses of this data matrix^22^,^27^,^33^. *Diamantinasaurus* was recovered as an opisthocoelicaudine by Poropat *et al*.^22^; by contrast, it is resolved herein as a non-lithostrotian titanosaur (Fig. 7), forming the clade *Savannasaurus* + (*Diamantinasaurus* + AODF 836) (Bremer support = 2). Further results pertaining to Titanosauria, and those based on our implied weights analysis, are reported in the Supplementary Information and in Supplementary Figs S1–S8.

### Palaeobiogeographic results

The results of our unconstrained BioGeoBEARS analyses (i.e. those that do not take palaeogeography into account) estimate Asia as being the sole area occupied by the most recent common ancestor (MRCA) of the *Diamantinasaurus* + *Savannasaurus* lineage and other titanosaurs, as well as the MRCA of *Wintonotitan* and other somphospondylans (Supplementary Table S26; Supplementary Figs S9–14). These results are consistent with previous suggestions that mid-Cretaceous Australian dinosaurian faunas are most similar to those of East Asia^13^,^14^, and that such faunas represent the product of direct trans-oceanic dispersal between these two regions^40^. The incorporation of palaeogeographic data in our analyses, however, has a marked effect on the inferred biogeographic history. In particular, the MRCAs of the two early Late Cretaceous Australian sauropod lineages are estimated to have occupied both Asia and South America minimally, and in several analyses these ancestral ranges also encompass Africa and Indo-Madagascar (Supplementary Table S26; Supplementary Figs S15–22). Moreover, when palaeogeographic data are included, the best-fitting maximum likelihood (ML) models are BAYAREALIKE and BAYAREALIKE + J (although DEC + J is also favoured in analyses where taxon midpoint ages are used to time-calibrate the tree, and constraints on intercontinental dispersal are more relaxed—see discussion in Supplementary Information for further details). BAYAREALIKE and BAYAREALIKE + J are ML models that exclude the possibility of vicariance^41^. Although it would be premature to rule out a role for vicariance in determining the palaeogeographic distributions of Cretaceous macronarians (see Supplementary Information), such a result does imply that the dominant biogeographic processes at work include dispersal, founder-event speciation, sympatry, and regional extinction.

## Discussion

The time-calibrated phylogenies and ancestral range estimations shown in Supplementary Figs S15–22 indicate that a number of somphospondylan and titanosaurian lineages had achieved widespread distributions across several continents by the Barremian (131–126 Ma) at the latest (although even earlier dates are possible given that we are dealing with minimum divergence times). The much more restricted geographic ranges of these lineages, observed ~20–30 million years later in the early Late Cretaceous, probably reflect range contractions caused by regional extinction events. Although such patterns could reflect sampling failures (at least in part), it is interesting to note that our conclusions are in line with several recent studies that have highlighted an important role for regional extinction as a mechanism for increasing endemism among dinosaurian faunas during the Cretaceous e.g. refs^12^,^42^. We regard this hypothesis of pre-Aptian dispersal across much of Pangaea, followed by endemism reflecting regional extinction, as a more plausible explanation for the affinities of mid-Cretaceous Australian sauropods than long-distance trans-oceanic dispersal of such large-bodied and highly terrestrial animals that occur relatively rarely in coastal and marine sediments^43^. If correct, our interpretation calls into question the biotic and/or abiotic factors that controlled the timing and direction of the dispersal events which produced the Australian faunas of the early Late Cretaceous. In this regard, climatic shifts provide a potential mechanism.

Our biogeographic results indicate that at least two somphospondylan lineages reached Australia in the Early Cretaceous: these events must have occurred by the late Albian at the latest, but they could have happened during the Barremian or even earlier. Our constrained biogeographic results are equivocal concerning the timing of these invasion events, with four analyses suggesting that the MRCAs of Australian lineages + other macronarians were already present in Australia prior to the Aptian, and six estimating these MRCAs as occupying Asia and South America in the Barremian and then dispersing into Australia later (see Supplementary Table S26). Constraining the timing of these events is critical if we wish to determine both the geographic route exploited by dispersing sauropods and the factors that potentially facilitated or hindered these events. At present, the oldest confirmed Australian macronarians, from stratigraphically well constrained units, are the "Hughenden sauropod" from the Toolebuc Formation, and the titanosauriform Austrosaurus mckillopi from the Allaru Mudstone Formation (late Albian, ~105–100 Ma)^6^,^20^,^22^. This is consistent with a relatively late arrival of macronarian sauropods into Australia. Although this observation might simply reflect poor sampling of earlier deposits, there is some evidence to support this ‘late date’ for somphospondylan dispersals.

Despite extensive prospecting of 115–105 Ma sediments in southeast Australia (mainly Victoria) over the past thirty years, and despite the recovery of a plethora of vertebrate fossils (including other dinosaurs such as theropods, ornithopods and ankylosaurs), no sauropod remains have been identified from these strata to date^7^,^14^. Although the absence of evidence of sauropods in these southeast Australian sediments is not necessarily evidence of their genuine absence, it should be borne in mind that no sauropods are yet known from palaeolatitudes higher than 66° in either hemisphere (Supplementary Information); southeast Australia was situated at ~70°S from 125–105 Ma^2^. Furthermore, sauropods were less diverse at high latitudes than at mid–low latitudes throughout the Cretaceous^44^, suggesting that they were likely best adapted to life in warmer climes. The late Early Cretaceous climate of southeast Australia has been interpreted as cool temperate^45^, with evidence for sporadic freezing in the south^7^. The apparent disinclination shown by sauropods towards cool climatic zones suggests that they would have avoided the polar regions, especially when the latitudinal thermal gradient was steep. Therefore, the absence of sauropod remains in southeast Australia from 115–105 Ma, coupled with the high palaeolatitude and polar palaeoclimate of this region, suggests that they were genuinely absent from at least southeast Australia during this period. Intriguingly, however, palaeogeographic reconstructions indicate that the only land route into Australia from Antarctica during the Aptian–Albian was via the cold, high latitude region of southeast Australia/Tasmania which was potentially impassable for sauropods (see below).

Palaeogeographically and palaeobiogeographically, South America is the most plausible ‘source area’ for Cretaceous sauropod immigrants into Australia. This would require dispersal to have taken place via a Patagonia–West Antarctica land connection, and across Antarctica itself. Indo-Madagascar could also have played a role in these dispersals, provided that they occurred prior to ~119 Ma (i.e. the timing of the separation of Indo-Madagascar from East Gondwana^2^; see Supplementary Information). Other dispersal routes via Africa and Indo-Madagascar are plausible and would have allowed Antarctica to be circumvented, although these dispersal events would have to have taken place before the end of the Late Jurassic (i.e. the timing of the separation of Africa from Indo-Madagascar and Antarctica^2^; see Supplementary Information). The latter seems less probable because it would require substantially longer ghost ranges and greater sampling failures than those already implied by our time-calibrated phylogenies. Thus, if somphospondylan lineages did not disperse into Australia until ~105–100 Ma, the only feasible land route for non-volant terrestrial organisms from South America would be via Antarctica.

Interestingly, floral evidence suggests that a sharp climatic barrier existed between Antarctica and South America during the Aptian and early Albian^46^. Thus, the climatic conditions of the land routes across both Patagonia–West Antarctica and East Antarctica–Australia (the latter requiring passage through southeast Australia and Tasmania^47^) would not have been conducive to sauropod dispersal during this interval. As a corollary of the above scenario, we hypothesize that the appearance of somphospondylan sauropods in Australia in the late Albian and Cenomanian–early Turonian reflects climatic shifts that removed these barriers to dispersal via this relatively high latitude route. Global warming during the late Albian–Turonian^48^ flattened the latitudinal thermal gradient^49^,^50^, which in turn would have enabled sauropods to disperse from South America, across Antarctica, to Australia via a set of suitable habitats.

Finally, it has been proposed that the retention of more mesic conditions in higher temperate latitudes, compared to more arid conditions at lower latitudes, might explain the palaeogeographically anomalous ‘Laurasian’ affinities of many Early Cretaceous dinosaurs from southeast Australia^14^. A recent phylogenetic reassessment of the relationships of *Diamantinasaurus*^22^ found that this taxon clustered with Late Cretaceous East Asian forms such as *Opisthocoelicaudia*. Such a result reinforces the previous notion of similarity between Australian and Asian dinosaurian faunas, but poses problems for the climatic zonation hypothesis proposed by Benson *et al*.^14^. If the differences between southeast Australian and South American faunas during the Early Cretaceous largely reflect ecological factors (e.g. habitat preferences) related to higher and lower latitude climates, then we might expect the lower latitude dinosaurian faunas of Queensland to display greater similarities with those of South America, rather than Asia. This complication, however, is resolved here by our current phylogenetic analyses which no longer support sister-taxon relationships between any of the Australian mid-Cretaceous sauropods and Asian forms.

**Figure 8:**
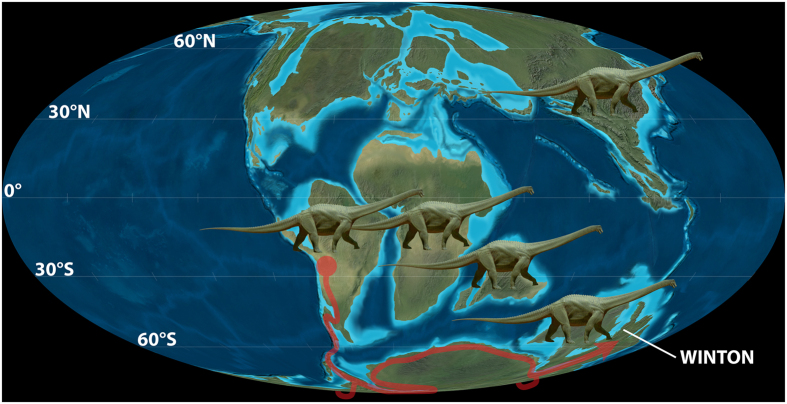
Palaeogeographic map of the mid-Cretaceous world. Showing the possible high latitude dispersal routes that might have been utilised by titanosaurs and other sauropods during the late Albian–Turonian. The base map is the 105 Ma time slice from the *Global Paleogeography and Tectonics in Deep Time* series by Ron Blakey [© Colorado Plateau Geosystems Inc.].

In short, current evidence suggests that a number of somphospondylan lineages were widespread across several continents during the Early Cretaceous. Furthermore, these lineages were prevented from reaching Australia until climatic warming of southern higher latitudes occurred during the late Albian. This facilitated sauropod dispersal from South America to Australia, via Antarctica (Fig. 8). Faunal turnover during the mid-Cretaceous, which was potentially driven by global warming^48^,^51^ and rising sea levels^52^, subsequently resulted in regional extinctions which increased continent-scale endemicity. Our hypothesis provides a framework within which the significance of future fossil discoveries and the results of more detailed phylogenetic and biogeographic analyses can be assessed. Given the very patchy nature of the Early Cretaceous fossil record^3^, especially in East Gondwana, considerable further work is required before the complex biogeographic history of the Australian Cretaceous terrestrial vertebrate fauna can be unraveled.

## Methods

### Phylogenetic approach

In order to constrain the phylogenetic positions of *Diamantinasaurus* and *Savannasaurus*, we conducted a phylogenetic analysis using an updated and expanded version of an existing titanosauriform data matrix^22^,^23^,^27^,^33^, which now comprises 397 characters (see SOM) scored for 72 operational taxonomic units (OTUs).

Character scores for the type specimens of *Diamantinasaurus matildae* (AODF 603) and *Wintonotitan wattsi* (Queensland Museum [QM] F7292) were updated following recent revisions^22^,^33^. *Savannasaurus elliottorum* (AODF 660) and the new specimen of *Diamantinasaurus matildae* (AODF 836) were added as separate OTUs, along with the titanosaurs *Aeolosaurus rionegrinus*^53^, *Epachthosaurus sciuttoi*^54^, *Futalognkosaurus dukei*^55^, *Isisaurus colberti*^56^, *Muyelensaurus pecheni*^57^, *Nemegtosaurus mongoliensis*^38^, and *Tapuiasaurus macedoi*^58^, which were identified as potentially important taxa due to temporal and/or anatomical overlap. Character parameters were set following Mannion *et al*.^27^ and analyses were run in TNT version 1.1^59^. We also analysed this data matrix using implied weights (see SOM).

### Palaeobiogeographic analyses

In order to investigate the biogeographic origins of Cretaceous Australian sauropods, we used a maximum likelihood approach to estimate the geographic ranges of their ancestral lineages. These palaeobiogeographic analyses of macronarian sauropods were performed using the R package BioGeoBEARS^41^, which implements six different models of how geographic ranges might evolve at ancestral nodes and along lineages (SOM). The phylogenetic topology employed in these analyses used the equal weights agreement subtree (Supplementary Fig. S2; see simplified version in Fig. 7), which was time-calibrated by applying two alternative approaches to assigning ages to taxa (see Supplementary Information). Seven continental areas were designated for the analyses: North America, Europe, Asia, South America, Africa, Indo-Madagascar, and Australia. Antarctica was excluded because of insufficient data. A total of eight analyses were run: two were unconstrained, whereas six were constrained using different dispersal multipliers reflecting Mesozoic palaeogeography. For the constrained analyses, the timespan from the Bajocian (Middle Jurassic, 170.3 Ma) to the terminal Maastrichtian (end-Cretaceous, 66 Ma) was divided into 22 time slices on the basis of the emplacement and removal of geographic barriers to dispersal (derived from a survey of the geophysical and palaeogeographic literature—see SOM). Log likelihood ratio tests and AIC analyses were used in order to determine which of the six ML models best fit the data.

## Additional Information

**How to cite this article**: Poropat, S. F. *et al*. New Australian sauropods shed light on Cretaceous dinosaur palaeobiogeography. *Sci. Rep*. **6**, 34467; doi: 10.1038/srep34467 (2016).

## Supplementary Material

Supplementary Information

Supplementary Data 1

## Figures and Tables

**Figure 1:**
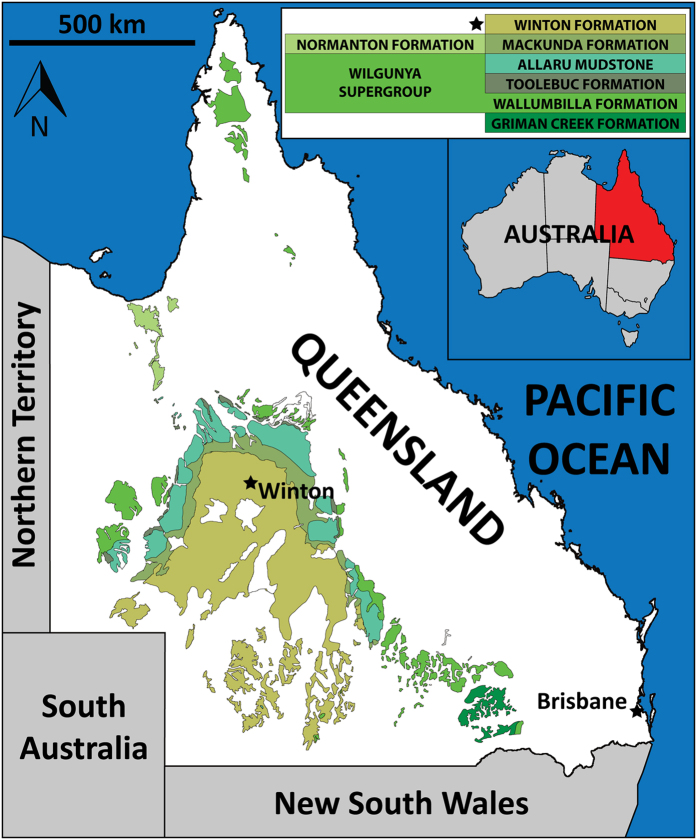
Map of Queensland, northeast Australia, showing the distribution of Cretaceous outcrop. From Poropat *et al*.^22^.

**Figure 2:**
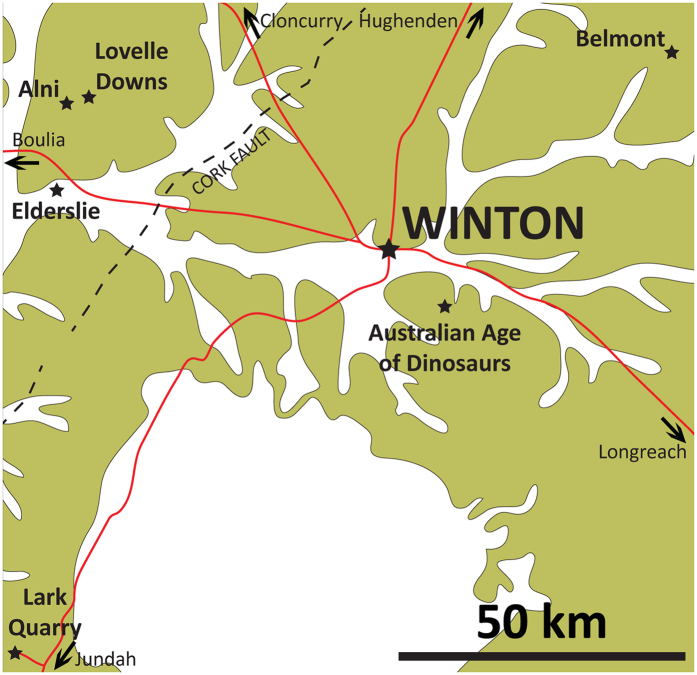
Winton Formation outcrop surrounding the town of Winton, with key localities marked. The holotype of *Savannasaurus elliottorum* (AODF 660) and the new specimen of *Diamantinasaurus matildae* (AODF 836) were both found on Belmont sheep station, whereas the type specimen of *Diamantinasaurus matildae* (AODF 603) was found on Elderslie sheep station. This map was drafted by the senior author (S.F.P.) in Adobe Illustrator CS5, and incorporates geological information from Vine^60^ and Vine & Casey^61^ [© Commonwealth of Australia (Geoscience Australia) 2016. This product is released under the Creative Commons Attribution 4.0 International Licence. http://creativecommons.org/licenses/by/4.0/legalcode].
