# Supplementary material for: New Australian sauropods shed light on Cretaceous dinosaur palaeobiogeography
Source: Sci Rep. 2016 Oct 20;6:34467. doi: 10.1038/srep34467 (PMC5072287; doi:10.1038/srep34467)
Supplement: Supplementary Information [file srep34467-s1.pdf]

New Australian sauropods shed light on Cretaceous dinosaur palaeobiogeography

Stephen F. Poropat, Philip D. Mannion, Paul Upchurch, Scott A. Hocknull, Benjamin P. Kear, Martin Kundrát, Travis R. Tischler, Trish Sloan, George H. K. Sinapius, Judy A. Elliott, and David A. Elliott

Supplementary Information:

I. Institutional abbreviations

II. Published sauropod occurrences in Australia, New Zealand and Antarctica

III. Phylogenetic analyses

IV. Biogeographic analyses

V. BioGeoBEARS analyses

I. Institutional abbreviations

AODF, Australian Age of Dinosaurs Museum of Natural History, The Jump-Up, Winton, Queensland, Australia; **MAU**, Museo Argentino Urquiza, Rincón de los Sauces, Neuquén, Argentina; **MUCPv**, Museo de la Universidad Nacional del Comahue, Neuquén, Argentina; **UNPSJB-PV**, Universidad Nacional de la Patagonia "San Juan Bosco" Paleovertebrados collections, Comodoro Rivadavia, Argentina.

II. Published sauropod occurrences in Australia, New Zealand and Antarctica

Australia

Winton Formation (uppermost Albian–lowermost Turonian)

Diamantinasaurus matildae Hocknull, White, Tischler, Cook, Calleja, Sloan and Elliott, 2009

AODF 603 (holotype and paratypes; incomplete postcranial skeleton): AODL 085 ("Matilda Site"), Elderslie Station, near Winton, Queensland[1,2].

AODF 836 (referred; incomplete skull, postcranial skeleton): QM L1333 / AODL 001 ("Elliot Site"), Belmont Station, near Winton, Queensland (this paper).

Wintonotitan wattsi Hocknull, White, Tischler, Cook, Calleja, Sloan and Elliott, 2009

QM F7292 (holotype; incomplete postcranial skeleton): QM L313 ("Triangle Paddock Site"), Elderslie Station, near Winton, Queensland[1,3-8].

QM F10916 (referred; four caudal vertebrae): Selwyn Park Station, near Winton, Queensland[1,3,7,8].

Savannasaurus elliottorum gen. et sp. nov.

AODF 660 (holotype; incomplete postcranial skeleton): AODL 082 (“Ho-Hum Site”), Belmont Station, near Winton, Queensland (this paper).

Titanosauriformes indet.

QM F311 (incomplete humerus): Navarre Station, near Blackall, Queensland[7,9].

QM F2470 (six incomplete caudal vertebrae): precise provenance unknown, Queensland[3,6,7,10].

QM F3390 (partial humerus, three incomplete metacarpals, partial femur): QM L031, Alni Station, near Winton, Queensland[3-7,11].

QM F6737 (incomplete scapula, incomplete ischium, eight incomplete caudal vertebrae): QM L244, Lovelle Downs Station, near Winton, Queensland[3-7].

QM F7291 (incomplete humerus, two incomplete metacarpals, incomplete femora): QM L315, Lovelle Downs Station, near Winton, Queensland[3-7].

QM F7880 (incomplete scapulocoracoid): QM L245 (“Joyce Site”), Elderslie Station, near Winton, Queensland[3-7].

Allaru Mudstone (upper Albian)

Austrosaurus mckillopi Longman, 1933

QM F2316 (incomplete presacral vertebrae, rib fragments): Clutha Station, near Maxwelton, Queensland[3,6,7,9,10,12-15].

Toolebuc Formation (upper Albian)

Titanosauriformes indet.

QM F6142 (incomplete cervical vertebra): Dunraven Station, near Hughenden, Queensland[3-5,7,14,16,17].

QM F13712 (incomplete caudal vertebra): Dunraven Station, near Hughenden, Queensland[7].

QM F40347 (incomplete humerus): QM L349, Silver Hills Station, near Richmond, Queensland[3-5,7,11].

Griman Creek Formation (upper Albian)

Sauropoda indet.

QM F10230 (cast: tooth): Lightning Ridge, Orana, New South Wales[3].

AM F66769 (tooth): Lightning Ridge, Orana, New South Wales[7,13,18].

AM F66770 (tooth): Lightning Ridge, Orana, New South Wales[7,13,18].

QM F11043: QM L380, Bymount, near Surat, Queensland[7,18].

QM F54817: QM L380, Bymount, near Surat, Queensland[7,18].

Broome Sandstone (Berriasian–Hauterivian)

Sauropoda indet.

Footprints *in situ* (not collected): Southwest Dampier Peninsula, Western Australia[19,20].

Walloon Subgroup, Injune Creek Group (middle Bathonian–Callovian)

Rhoetosaurus brownei Longman, 1926

QM F1659 (syntype series and referred specimens [hypodigm]: incomplete postcranial skeleton): Taloona Station (originally part of Durham Downs Station), near Roma, Queensland[21-27].

Colalura Sandstone (Bajocian)

Sauropoda indet.

UWA 82468 (incomplete caudal vertebra): Bringo Railway Cutting, near Geraldton, Western Australia[28].

New Zealand

Maungataniwha Member, Tahora Formation (upper Campanian–lower Maastrichtian)

Sauropoda indet.

GNS CD542 (rib): Mangahouanga Stream, tributary of Te Hoe River, North Island[29,30].

Titanosauria indet.

GN CD586 (incomplete caudal vertebra): Mangahouanga Stream, tributary of Te Hoe River, North Island[31].

Antarctica

López de Bertodano Formation (upper Maastrichtian)

?Sauropoda indet.

Footprints *in situ* (not collected): Tessore Hill, Snow Hill Island[32,33].

Gamma Member, Snow Hill Island Formation (upper Campanian)

Titanosauria indet.

MLP 11-II-20-1 (incomplete caudal vertebra): Santa Marta Cove, James Ross Island[33,34].

III. Phylogenetic analyses

Methods

Characters 1–279 are the same as those presented in Mannion *et al.*[35] and are not repeated here, although six of these (characters 40, 51, 142, 164, 195 and 225) are revised (see below). Characters 280–285 are those that were added to this data matrix by Upchurch *et al.*[36] and are repeated below. Characters 286–397 are newly added to this data matrix and presented below, including their sources. The full data matrix is provided as a nexus file.

Taxon scores follow Mannion *et al.*[35] and the subsequent revisions presented in Poropat *et al.*[2,8] and Upchurch *et al.*[36], with the following additional changes (the first number denotes the character, and the number/symbol in parentheses denotes the new score). Score changes are based on: (1) the references and personal observations cited in Mannion *et al.*[35] (table 4); (2) Upchurch and Mannion (unpublished data); (3) new publications (Royo-Torres *et al.*[37]; Carrano and D'Emic[38]); and (4) personal observations of *Saltasaurus* and *Tehuelchesaurus* (PDM, PU & SFP, 2013), and *Erketu* (PDM, 2015):

Omeisaurus: 40 (1)

Camarasaurus: 99 (0); 116 (0)

Nigersaurus: 51 (1); 224 (1)

Apatosaurus: 99 (1)

Diplodocus: 72 (0); 142 (0)

Alamosaurus: 43 (0); 57 (0); 164 (1); 256 (1); 279 (1)

Angolatitan: 51 (1)

Aragosaurus: 11 (?); 40 (1); 105 (?); 108 (?); 109 (?); 110 (?); 111 (?); 112 (?); 113 (?); 225 (0)

Atlasaurus: 40 (2)

Brachiosaurus: 40 (2)

Cedarosaurus: 40 (2); 51 (2); 164 (0); 195 (2); 218 (?)

Chubutisaurus: 40 (1)

Daxiatitan: 164 (1)

Diamantinasaurus: 51 (1); 164 (?); 231 (0); 232 (1); 264 (0)

Dongyangosaurus: 164 (1)

Erketu: 68 (0); 71 (1); 121 (0); 261 (1)

Euhelopus: 142 (0); 218 (?)

Europasaurus: 99 (0); 114 (0); 116 (0)

"French *Bothriospondylus*": 40 (2)

Galveosaurus: 225 (1)

Giraffatitan: 40 (2); 51 (1); 99 (0)

Janenschia: 51 (1)

Jiangshanosaurus: 164 (?); 279 (?)

Ligabuesaurus: 40 (1)

Malawisaurus: 51 (1); 92 (0); 98 (0); 178 (?); 179 (?)

Opisthocoelicaudia: 51 (1)

Paluxysaurus: 40 (1); 57 (1)

Phuwiangosaurus: 92 (0); 142 (0/1)

Rapetosaurus: 51 (1); 116 (0); 225 (1); 241 (0)

Saltasaurus: 51 (1); 53 (0); 57 (0); 69 (1); 99 (1); 100 (0); 119 (0); 122 (2); 130 (0); 148 (2); 170 (1); 171 (1); 174 (1); 180 (1); 183 (0); 187 (1); 188 (?); 190 (1); 194 (0); 201 (0); 209 (0); 212 (0); 225 (0); 231 (0); 236 (1); 239 (1); 241 (1); 248 (1); 250 (1); 252 (1); 261 (1); 262 (1); 272 (1); 273 (1); 274 (0)

Tehuelchesaurus: 51 (1); 218 (0); 224 (0); 258 (?); 261 (1)

Tendaguria: 142 (?)

Venenosaurus: 51 (2); 195 (1/2)

Newly added operational taxonomic units (OTUs)

In addition to the 63 taxa included in the data matrix of Mannion *et al.*[35], we have incorporated the titanosaurs *Aeolosaurus rionegrinus*, *Epachthosaurus sciuttoi*, *Futalognkosaurus dukei*, *Isisaurus colberti*, *Muyelensaurus pecheni*, *Nemegtosaurus mongoliensis* and *Tapuiasaurus macedoi*, as well as our new taxon *Savannasaurus elliottorum*. We also include AODF 836 (referred herein to *Diamantinasaurus*) as a separate OTU. *Aeolosaurus rionegrinus* is scored based on Powell[39], and restricted to the type specimen; *Epachthosaurus* is scored based on Martínez *et al.*[40] and personal observations of that specimen (UNPSJB-PV 920) by PDM, PU and SFP (2013); *Futalognkosaurus* is scored based on Calvo *et al.*[41], Calvo[42], and personal observations of that specimen (MUCPv 323) by PDM (2009); *Isisaurus* is scored based on Jain and Bandyopadhyay[43], supplemented with Wilson[44] and Curry Rogers[45], and we follow Wilson *et al.*[46,47] in referring the 'Dongargaon braincase'[48] to this taxon; *Muyelensaurus* is scored based on Calvo *et al.*[49] and personal observations of that specimen (MAU-PV-LL [multiple specimen numbers]) by PDM (2014) – as there are multiple individuals of this taxon from one bonebed, we take a conservative approach to assessing the referral of elements to discrete individuals, and therefore do not use

anatomical ratios relating to more than one element; *Nemegtosaurus* is scored based on Wilson[50]; *Tapuiasaurus* is scored based on Zaher *et al.*[51].

Revised characters

C40. Humerus to femur proximodistal length ratio: 0.8 or less (0); >0.8 to 0.9 (1); greater than 0.9 (2) (Wilson[44]; Upchurch *et al.*[52]; an additional state has been added to capture greater variation) [ordered].

C51. Ulna, ratio of mediolateral width of proximal end (equivalent to anteromedial arm) to anteroposterior width of proximal end (equivalent to anterolateral arm): less than 1.4 (0); 1.4 to <2.0 (1); 2.0 or greater (2) (Mannion *et al.*[35]; Upchurch *et al.*[36]; Wilson[44]; note that in this revised version of the character, the long-axes of the anteromedial and anterolateral processes are extrapolated posteriorly so that they intersect close to the position of the olecranon, and each process length is then measured from this intersection to the tip of each process) [ordered].

C142. Middle–posterior dorsal centra, ventral keel: absent (0); present (1) (Mannion *et al.*[35]; Upchurch and Mannion[53]; modified here to separate anterior and middle–posterior dorsal vertebrae – see C332).

C164. Middle-posterior dorsal neural spines, orientation: vertical or slightly posterodorsal (0); strongly posterodorsal, oriented at 45° to the horizontal or greater (1) (Wilson[44]; modified here to remove dependence on neural spine height and orientation of diapophyses; note that this excludes the posteriormost dorsal neural spines, which usually revert to being dorsally directed).

C195. Anterior caudal neural spines, project: posterodorsally (0); dorsally (sometimes with a subtle anterior deflection) (1); anterodorsally, such that the anterodorsal margin of the neural spine projects beyond the anterior margin of the centrum (2) (Mannion *et al.*[35]; González Riga *et al.*[54]; modified here to include taxa with anterodorsally oriented neural spines) [ordered].

C225. Humeral deltopectoral crest, projection: anteriorly or anterolaterally (0); anteromedially, extending across the anterior face of the humerus (1) (Mannion *et al.*[35]; Upchurch *et al.*[52]; Upchurch and Mannion[53]; modified here to separate orientation and distal thickening of the deltopectoral crest – see C360).

Characters from Upchurch *et al.*[36]

C280. Humerus, strong bulge or tuberosity (site for M. scapulohumeralis anterior) on the lateral margin of the posterior surface (usually visible in anterior view), approximately level with the most prominently developed portion of the deltopectoral crest: absent or weakly developed, and not visible in anterior view (0); present, forms a distinct lateral bulge that interrupts the line of the lateral humeral margin in anterior view (1) (Upchurch *et al.*[36]; Borsuk-Białynicka[55]).

C281. Ulna, posterior process of proximal end: weakly developed, so that the proximal profile of the ulna is ‘V’-shaped (formed by the anteromedial and anterolateral processes) (0); strongly developed, so that the proximal profile of the ulna is ‘T’- or ‘Y’-shaped, and there is a deep fossa between the anteromedial and posterior processes, rivalling the radial fossa in depth (1) (Upchurch *et al.*[36]).

C282. Ulna, shape of the distal end: comma-shaped, with tapering curved anterior process associated with an anteromedial fossa for reception of the radius (0); elliptical or oval in outline, with the anteromedial fossa strongly reduced or absent (1) (Upchurch *et al.*[36]).

C283. Radius, profile of proximal end: 'D'-shaped or elliptical (0); oval or subtriangular, with marked tapering towards the medial process (1) (Upchurch *et al.*[36]).

C284. Radius, ridge or flange on medial margin, near proximal end, for attachment of the M. biceps brachii and M. brachialis inferior: absent or very weakly developed (0); present, projecting beyond the medial margin of the main radial shaft (1) (Upchurch *et al.*[36]; Borsuk-Białynicka[55]).

C285. Radius, posterior margin of distal end: lacks condylar-like processes and fossa (0); forms two low rounded processes (posteromedial and posterolateral), with a shallow fossa between them (1) (Upchurch *et al.*[36]; D'Emic[56]).

Added characters

Cranial characters

C286. Snout, shape in dorsal view, Premaxillary-Maxillary Index (PMI): 60% or less (0); >60% to 75% (1); greater than 75% (2) (Upchurch[57]; Whitlock[58]; modified here; note that this can be calculated using the lower jaw when the skull is incomplete) [ordered].

C287. Premaxilla, shape of ascending process in lateral view: convex (0); concave, with a large dorsal projection (1); sub-rectilinear and directed posterodorsally (2) (Whitlock[58]).

C288. Maxilla, anterior (dentigerous) portion of lateral surface excavated by dorsoventrally elongate, deep vascular grooves: absent (0); present (1) (Wilson[44]; Wilson[50]).

C289. Maxilla, foramen anterior to the preantorbital fenestra: absent (0); present (1) (Zaher *et al.*[51]).

C290. Maxilla, position of external opening of the preantorbital fenestra: lies below antorbital fenestra (0); lies anterior to antorbital fenstra, with the posterior margin of the preantorbital fenestra lying entirely anterior to the anterior margin of the antorbital fenestra (1) (Upchurch and Mannion[53]; Marpmann *et al.*[59]).

C291. Maxilla, preantorbital fenestra development: weakly developed, shallow fossa (difficult to distinguish from posterior maxillary foramen) (0); deep, sharp-lipped fossa (1) (Zaher *et al.*[51]; Upchurch and Mannion[53]; Carballido *et al.*[60]).

C292. Maxilla, posterior extent of dorsal (ascending) process: anterior to, or level with, posterior end of main body (0); extending posterior to posterior end of main body (1) (Whitlock[58]).

C293. Maxilla, ventral margin of jugal process: reduced in dorsoventral height, such that the ventral margin is strongly emarginated relative to the remainder of the ventral margin of the maxilla (0); continuous with ventral margin of remainder of maxilla, or very gently emarginated (1) (Upchurch and Mannion[53]; Curry Rogers[61]).

C294. Maxilla, contact with quadratojugal: absent or small (i.e. no more than a point contact) (0); extensive (1) (Upchurch and Mannion[53]; Upchurch[62]).

C295. Jugal, dorsal process: present (0); absent (1) (Tschopp *et al.*[63]).

C296. Jugal, contact with ectopterygoid: present (0), absent, ectopterygoid contacts maxilla instead (1) (Upchurch[62]).

C297. Frontal, anteroposterior length to transverse width ratio: 1.0 or greater (0); less than 1.0 (1) (Whitlock[58]; Tschopp and Mateus[64]; modified here)

Quantitative values: *Shunosaurus*=1.26; *Omeisaurus*=0.95; *Mamenchisaurus*=1.0; *Camarasaurus*=1.0; *Nigersaurus*=1.4; *Apatosaurus*=0.72; *Diplodocus*=0.75; *Europasaurus*=1.17; *Giraffatitan*=0.93; *Phuwiangosaurus*=1.09; *Rapetosaurus*=0.82; *Saltasaurus*=0.96; *Nemegtosaurus*=0.76; AODF 836=0.66 (note that other taxa can be scored for this character, but exact values are unknown).

C298. Frontal, lateral margin: expands posteriorly, orbital margin concave in dorsal view (0); unexpanded posteriorly, orbital margin straight or convex in dorsal view (1) (Upchurch and Mannion[53]; Whitlock *et al.*[65]).

C299. Frontal, contribution to margin of supratemporal fenestra: present (0); absent, frontal excluded from anterior margin of fenestra by a postorbital-parietal contact (1) (Wilson and Sereno[66]).

C300. Orbit, anterior-most point: anterior to the anterior extremity of lateral temporal fenestra (0); approximately level with or posterior to anterior extent of lateral temporal fenestra (1) (Upchurch and Mannion[53]; Upchurch[62]; Gauthier[67]).

C301. Lateral temporal fenestra, shape in lateral view: taller than wide anteroposteriorly and subtriangular (anteroposteriorly broader ventral margin and narrower dorsal apex) (0); linear, slit-like, crescentic (longer anteroposteriorly than high dorsoventrally) (1) (Upchurch and Mannion[53]; Harris[68]).

C302. Postorbital, posterior (squamosal) process: present as a distinct process (0); absent (1) (Wilson[44]; Wilson[50]).

C303. Parietal, relative height of suture with frontal: lies level with or above the dorsal surfaces of the frontals and parietals (0); lies below the dorsal surfaces of the parietals and frontals (i.e. in a deep transverse trough) (1) (Upchurch and Mannion[53]; Curry Rogers[61]).

C304. Parietal, elevation of anterior margin creates a step-like curving crest transversely, where the parietal meets the frontal: absent (0); present (1) (Upchurch and Mannion[53]; Curry Rogers[61]; Curry Rogers and Forster[69]).

C305. Pterygoid, morphology: robust element (0); plate-like, with its three processes coplanar (1) (new character: based on Curry Rogers and Forster[69] and Wilson[50]).

C306. Pterygoid, sutural contact with ectopterygoid: anteroposteriorly elongate, along the medial or lateral surface (0); anteroposteriorly short, restricted to the anterior tip of the pterygoid (1) (Zaher *et al.*[51]; modified here).

C307. Pterygoid, palatobasal contact for basipterygoid articulation with a convex, rocker-like articular surface: absent (0); present (1) (Wilson[44]).

C308. Supraoccipital, longitudinal groove along posterodorsal surface: absent (0); present (i.e. sagittal crest divided into two subparallel parasagittal crests with central groove) (1) (González Riga *et al.*[54]; Curry Rogers[61]; Curry Rogers and Forster[69]).

C309. Supraoccipital-exoccipital-opisthotic, paired facets for articulation with the proatlas: absent (0); present (1) (Upchurch and Mannion[53]).

C310. Parasphenoid rostrum, cross-sectional shape: triangular (0); transversely thin, sheet-like (1) (Upchurch and Mannion[53]; Upchurch[57]; Upchurch[62]; Tschopp *et al.*[63]; Berman and McIntosh[70])

C311. Basipterygoid processes, shape: widely diverging at 30° or more (0); narrowly diverging at less than 30° (1) (Upchurch[57]; Upchurch[62]; Mannion *et al.*[71]).

C312. Basipterygoid processes, orientation in lateral view: directed 80° or more to skull roof (normally perpendicular) (0); angled less than 80° to skull roof (anteroventrally directed) (1) (Wilson[44]; Whitlock[58]; Mannion *et al.*[71]; McIntosh[72]; modified here).

C313. Basioccipital, orientation of occipital condyle relative to the horizontal plane (in lateral view with supraoccipital held in a vertical plane): 60° or less (0); greater than 60° (typically close to 90°) (1) (Upchurch[57]; Upchurch[62]).

C314. Basal tubera, angle of divergence in posterior view: less than 50° (0); more than or equal to 50° (1) (Upchurch and Mannion[53]; Curry Rogers[61]).

C315. Basisphenoid, relative position of the external opening for cranial nerve VI: lies ventral, and generally close, to the opening for cranial nerve III (0); lies anteroventral to, and more distant from, the opening for cranial nerve III (1) (Upchurch and Mannion[53]; Remes[73]).

C316. Basisphenoid, opening for cranial nerve VI: penetrates the pituitary fossa (0); does not penetrate the pituitary fossa (1) (Upchurch and Mannion[53]; Paulina Carabajal[74]).

C317. Basioccipital+exoccipital-opisthotic, number of exits for cranial nerve XII: 2 (0); 1 (1) (Upchurch and Mannion[53]; Paulina Carabajal[74]).

C318. Basisphenoid, position of the external foramen of the internal carotid artery: lateral to basipterygoid process (0); medial to basipterygoid process (1) (Upchurch and Mannion[53]; Paulina Carabajal[74]).

C319. Dentary, angle between the long-axis of the anterior margin (mandibular symphysis) and the long-axis of the main body of the dentary, in lateral view: greater than 90°, with the dorsal margin of the dentary extending further anteriorly than the ventral margin (0); approximately 90°, with the dorsal and ventral margins extending an equal distance anteriorly (1) (Wilson[44]; Upchurch and Mannion[53]; Upchurch[57]; Salgado and Calvo[75]).

C320. Tooth crowns, longitudinal groove paralleling mesial and distal margins on the labial surface: labial grooves present (0); absent (1) (Upchurch and Mannion[53]; Upchurch[62]).

Axial characters

C321. Atlantal intercentrum, ventral margin of posterior surface: straight or convex (0); concave, forming ventrolateral projections (1) (Upchurch and Mannion[53]; González Riga and Ortíz David[76]).

C322. Axis, aEI (average elongation index: anteroposterior length of centrum (excluding articular ball if present) divided by mean average value of the mediolateral width and dorsoventral height of posterior articular surface of centrum): 2.0 or greater (0); less than 2.0 (1) (Upchurch and Mannion[53]; Upchurch *et al.*[77]).

Quantitative values: *Shunosaurus*=2.19; *Omeisaurus*=3.08; *Mamenchisaurus*=2.18; *Camarasaurus*=1.52; *Apatosaurus*=2.25; *Alamosaurus*=1.43; *Erketu*=2.71; *Euhelopus*=2.84; *Giraffatitan*=2.21; *Mongolosaurus*=2.29; *Saltasaurus*=1.41; AODF 836=1.46; (note that other taxa can be scored for this character, but exact values are unknown).

C323. Postaxial cervical centra, pneumatization of lateral surface: lateral pneumatic opening occupies approximately anterior two-thirds of centrum or more (0); reduced and restricted to less than the anterior two-thirds of the centrum (1) (Upchurch and Mannion[53]; Whitlock[58]).

C324. Postaxial cervical centra, midline notch on the dorsal margin of the posterior articular surface: absent (0); present (1) (Carballido *et al.*[60]).

C325. Postaxial anterior cervical vertebrae, prezygapophyses: extend anterior to the anterior tip of the condyle (0); terminate level with or posterior to the anterior tip of the condyle (1) (Curry Rogers[45]; Upchurch and Mannion[53]; Curry Rogers[61]).

C326. Postaxial anterior cervical neural spines, orientation of posterior margin in lateral view: dorsal (vertical) or anterodorsal (0); posterodorsal (1) (Curry Rogers[61]).

C327. Middle cervical neural spines, height to arch height ratio: 2.0 or lower (0); greater than 2.0 (1) (Whitlock[58]; Rauhut *et al.*[78]).

C328. Posterior (usually just the posterior-most) cervical neural arches, postzygapophyses: terminate at or beyond the posterior edge of the centrum (0); terminate in front of the posterior edge of the centrum (1) (Upchurch and Mannion[53]; Tschopp and Mateus[64]).

C329. Posterior cervical neural spines, horizontal, rugose ridge immediately below spine summit on lateral surface: absent, spinodiapophyseal fossa fades out gradually dorsally (0); present, serves as distinct dorsal edge of the spinodiapophyseal fossa (1) (Tschopp and Mateus[64]).

C330. Posteriormost cervical and anteriormost dorsal neural spines, shape in anterior/posterior view: taper dorsally, or mediolateral width remains constant along length (0); expand dorsally, with a strongly convex dorsal margin (‘paddle-shaped’) (1) (González Riga[79]; D'Emic[80]; modified here).

C331. Cervical ribs, dorsal surface of proximal portion of shaft: excavated, forming a longitudinal groove (0); unexcavated (1) (new character: based on Poropat *et al.*[8]).

C332. Anterior dorsal centra, ventral keel on midline: absent (0); present (1) (Upchurch and Mannion[53]; Mannion *et al.*[71]).

C333. Middle–posterior dorsal centra, ventral surface: flat or transversely convex (0); transversely concave, between ventrolateral ridges (1) (Upchurch *et al.*[52]; Curry Rogers[61]; modified here).

C334. Middle–posterior dorsal centra, lateral pneumatic foramina divided by internal ridge/s: absent (0); present (1) (Mannion *et al.*[71]; Salgado *et al.*[81]).

C335. Anterior dorsal neural arches, shape of anterior neural canal opening: height greater than or equal to width (0); height is less than width (1) (Upchurch and Mannion[53]; Curry Rogers[61]).

C336. Anterior–middle dorsal neural arches, vertical midline ridge ('median infrapostzygapophyseal lamina') extending from roof of neural canal to ventral midpoint of postzygapophyses/intrapostzygapophyseal lamina (TPOL): absent (0); present (1) (Curry Rogers[45]; Curry Rogers[61]; González Riga[82]; Gallina[83]; Gallina and Apesteguía[84]).

C337. Anterior–middle dorsal neural arches, zygapophyseal articulation angle: between horizontal and less than 40° to the horizontal (0); strongly dorsomedially oriented (40° or more) (1) (Upchurch and Mannion[53]; Carballido *et al.*[60]).

C338. Middle–posterior dorsal neural arches, neural canal in anterior view: entirely surrounded by the neural arch (0); enclosed in a deep fossa in the dorsal surface of the centrum (i.e. much of the canal is enclosed laterally by pedicels that are part of the centrum rather than the neural arch) (1) (Upchurch and Mannion[53]; Carballido *et al.*[60]).

C339. Middle–posterior dorsal neural arches, position of parapophysis: posterior to the vertical plane defined by the anterior margin of the centrum (excluding any convex articular condyle) (0); level with, or anterior to, the vertical plane defined by the margin of the centrum (excluding any convex articular condyle) (1) (Tschopp and Mateus[64]).

C340. Middle–posterior dorsal neural arches, anterior centradiapophyseal lamina (ACDL): absent (0); present (1) (Upchurch and Mannion[53]; Mannion *et al.*[71]).

C341. Posterior dorsal neural arches, zygapophyseal articulation angle relative to horizontal line: less than 30°, usually close to horizontal (0); steeply oriented, 30° or greater (1) (new character: based on Carballido *et al.*[60]).

C342. Middle–posterior dorsal neural spines (single, not bifid), SPRLs: remain separate or converge at about spine midheight (or above) to form a dorsally restricted median composite lamina (SPRF well-developed and occupies the ventral half of the anterior spine surface) (0); SPRLs, if present, are short and merge into the PRSL close to the base of the spine (the PRSL may extend between the bases of the SPRLs to the top of the TPRL) (1) (Upchurch and Mannion[53]; Whitlock[58]; Carballido *et al.*[60]; Upchurch[62]).

C343. Middle–posterior dorsal neural spines, postspinal lamina: does not extend ventral to the neural spine (0); extends ventral to the neural spine, beyond the postzygapophyseal articular surfaces (1) (new character; note that only taxa scored as "0" for C169 are scored for this character).

C344. Middle–posterior dorsal neural spines, anterior spinodiapophyseal lamina (aSPDL): absent (0); present (1) (Upchurch *et al.*[52]; Upchurch and Mannion[53]; note that the presence of an aSPDL can only be confirmed when a pSPDL is also present).

C345. Middle–posterior dorsal neural spines, SPDL bifurcates at its dorsal end to create a SPDL-F: absent (0); present (1) (Upchurch and Mannion[53]; note that this refers to a bifurcation of the posterior SPDL when there are two SPDLs, rather than just the presence of an aSPDL and pSPDL).

C346. Sacrum, ratio of mediolateral width across sacral vertebrae and ribs (taken at midlength on the coossified sacrum) to average length of a sacral centrum: less than 4.0 (0); 4.0 or higher (1) (Upchurch[57]).

C347. Sacral centra, ratio of mediolateral width of middle sacral centra to first and last sacral centra: approximately constant, ratio less than 1.3 (0); 1.3 or greater (1) (Upchurch and Mannion[53]; Salgado *et al.*[85]; D'Emic[86]).

C348. Sacral neural spines, all fused, forming a dorsal ‘platform’: absent (0); present (1) (new character: based on Martínez *et al.*[40]).

C349. First caudal centrum, anterior articular face shape: flat or concave (0); convex (1) (Wilson[44]; Upchurch and Mannion[53]; Whitlock[58]).

C350. Anterior–middle caudal centra (excluding Cd1), comparison of anterior and posterior articular faces: anterior face more concave than posterior one, or these two faces are equally concave (0); posterior face more deeply concave than anterior face (1) (Upchurch and Mannion[53]; González Riga *et al.*[54]; Carballido *et al.*[60]; D'Emic *et al.*[87]).

C351. Middle caudal centra with convex posterior articular surface, condyle dorsally displaced: absent (0); present (1) (new character: based on González Riga[82]).

C352. Anteriormost caudal neural arches, prezygapophyses curve downwards (‘droop’) at their distal ends: absent (0); present (1) (Santucci and Arruda-Campos[88]).

C353. Anterior caudal neural spines, anterior expansion of lower portion of spinoprezygapophyseal lamina (SPRL): absent (0); present (‘SPRL-process’) (1) (D'Emic[80]; Mannion *et al.*[71]; note that this is best observed in lateral view).

C354. First caudal rib, subtriangular process projects posteriorly at approximately midlength: absent (0); present (1) (Upchurch and Mannion[53]; Mannion and Calvo[89]).

C355. Anteriormost caudal ribs, tubercle on dorsal surface at approximately midlength: absent (0); present (1) (Upchurch and Mannion[53]; D'Emic *et al.*[87]; Kellner *et al.*[90]).

C356. Anterior–middle chevrons, articular facet surface: flat or anteroposteriorly convex (0); divided into anterior and posterior facets by a furrow (1) (Powell[39]; D'Emic[80]; Santucci and Arruda-Campos[88]; Powell[91]).

C357. Anterior–middle chevrons, posteroventrally directed ridge or bulge on lateral surface of distal half of proximal ramus: absent (0); present (1) (Upchurch and Mannion[53]; Santucci and Arruda-Campos[88]).

Appendicular characters

C358. Scapula, ventrolateral margin of acromion, anteroposteriorly concave region posterior to glenoid, followed by a flattened area: absent (0); present (1) (new character: based on Poropat *et al.*[2]).

C359. Scapular blade, ridge on medial surface, close to junction with acromial plate and near dorsal margin: absent (0); present (1) (Upchurch *et al*.[52]; Sanz *et al*.[92]).

C360. Scapular blade, orientation of blade long-axis with respect to coracoid articulation: more than 70° (usually approximately perpendicular) (0); 70° or less (1) (Wilson[44]; Upchurch and Mannion[53]).

Quantitative values: *Shunosaurus*=90°; *Omeisaurus*=100°; *Mamenchisaurus*=80°; *Camarasaurus*=90°; *Nigersaurus*=65°; *Apatosaurus*=80–90°; *Diplodocus*=70–75°; *Alamosaurus*=70°; *Angolatitan*=65°; *Aragosaurus*=75°; *Cedarosaurus*=90°; *Chubutisaurus*=60°; *Daxiatitan*=80°; *Euhelopus*=80–90°; *Europasaurus*=80°; *Giraffatitan*=85°; *Huanghetitan ruyangensis*=85°; *Ligabuesaurus*=65°; *Opisthocoelicaudia*=80°; *Saltasaurus*=60°; *Tehuelchesaurus*=90° (note that other taxa can be scored for this character, but exact values are unknown).

C361. Coracoid, glenoid: does not expand strongly laterally relative to the lateral surface of the coracoid (0); expands prominently laterally and curves dorsolaterally so that part of the glenoid articular surface can be seen in lateral view (1) (Upchurch and Mannion[53]).

C362. Sternal plate, shape of posterior margin in dorsal/ventral view: convex (0); straight (1) (González Riga *et al*.[54]; González Riga[82]).

C363. Sternal plate, anteroposteriorly directed ridge on ventral surface, at the anterior end: absent (0); present (1) (Upchurch *et al*.[52]; Sanz *et al*.[92]).

C364. Humerus, proximal margin in anterior/posterior view: straight or convex (0); sinuous, as a result of a prominently developed process (attachment site for M. supracoracoideus) on the lateral margin of the proximal end (1) (Upchurch and Mannion[53]; González Riga *et al*.[54]; Upchurch[57]; González Riga[82]).

C365. Humerus, proximal end: expands laterally relative to the shaft, giving the humerus an hourglass outline in anterior view (0); asymmetrical, with no expansion of lateral margin relative to shaft (1) (Upchurch and Mannion[53]; Tschopp *et al*.[63]).

C366. Humerus, humeral head forms a prominent subcircular process on the posterior surface of the proximal end: absent (0); present (1) (new character: based on Bonaparte *et al.*[93]; Upchurch *et al.*[36]).

C367. Humerus, prominent vertical ridge extends along the lateral margin of the posterior surface, from the proximolateral corner to approximately the level of the deltopectoral crest (this ridge defines the lateral margin of the lateral triceps fossa and causes this fossa to be much deeper than the medial one): absent (0); present (1) (Upchurch and Mannion[53]).

C368. Humerus, tuberosity for attachment of the M. coracobrachialis on the anterior surface of the proximal third: absent (0); present (1) (Powell[39]; Upchurch and Mannion[53]; Otero[94]; Harris[95]).

C369. Humerus, deltopectoral crest, mediolateral thickness of anterior attachment surface: approximately constant along length (0); distal half mediolaterally expanded relative to proximal half (often doubling in thickness) (1) (Wilson[44]; Upchurch and Mannion[53]; note that the anterior attachment surface of taxa with a medially deflected deltopectoral crest faces primarily medially).

C370. Humerus, ratio of maximum mediolateral width of distal end to proximodistal length: 0.30 or greater (0); less than 0.30 (1) (Upchurch and Mannion[53]).

Quantitative values: *Shunosaurus*=0.33; *Omeisaurus*=0.29; *Mamenchisaurus*=0.28; *Camarasaurus*=0.30; *Nigersaurus*=0.27; *Apatosaurus*=0.38; *Diplodocus*=0.29; *Alamosaurus*=0.34; *Angolatitan*=0.29; *Aragosaurus*=0.29; *Brachiosaurus*=0.20; *Cedarosaurus*=0.24; *Chubutisaurus*=0.27; *Diamantinasaurus*=0.34; *Epachthosaurus*=0.33; *Euhelopus*=0.25; "French *Bothriospondylus*"=0.27; *Galveosaurus*=0.25; *Giraffatitan*=0.23; *Haestasaurus*=0.35; *Janenschia*=0.35; *Lapparentosaurus*=0.27; *Ligabuesaurus*=0.22; *Malawisaurus*=0.29; *Opisthocoelicaudia*=0.42; *Phuwiangosaurus*=0.29; *Rapetosaurus*=0.26; *Saltasaurus*=0.42; *Tehuelchesaurus*=0.29 (note that other taxa can be scored for this character, but exact values are unknown).

C371. Radius, beveling of distal end relative to long-axis of shaft: restricted to lateral half (0); extends across the entire distal end (1) (Upchurch and Mannion[53]).

C372. Ulna, angle between long-axes of anteromedial and anterolateral processes in proximal end view: 80° or greater (usually approximately a right-angle) (0); less than 80° (acute) (1) (Tschopp *et al.*[63]).

C373. Metacarpals, longest metacarpal to radius proximodistal length ratio: less than 0.50 (0); 0.50 or greater (1) (Upchurch and Mannion[53]).

C374. Metacarpals, metacarpal II, ratio of minimum transverse width of shaft to metacarpal length: 0.2 or higher (0); less than 0.2 (1) (Sekiya[96]).

C375. Metacarpals, metacarpal III: longest metacarpal (0); shorter than at least one other metacarpal (1) (Upchurch and Mannion[53]; Curry Rogers[61]).

C376. Metacarpals, metacarpal IV, distal end profile: subrectangular (0); possesses small pointed lateral and medial projections such that the dorsal margin is longer than the ventral margin, producing a dorsoventrally compressed hexagonal or trapezoidal outline (1) (Upchurch and Mannion[53]).

C377. Metacarpals, metacarpal V, ratio of proximal end long axis diameter to that of metacarpal I: less than 1.0 (0); 1.0 or greater (1) (Upchurch and Mannion[53]; D'Emic[80]).

Quantitative values: *Shunosaurus*=0.68; *Mamenchisaurus*=0.83; *Camarasaurus*=0.70; *Apatosaurus*=0.76; *Alamosaurus*=1.07; *Atlasaurus*=0.82; *Diamantinasaurus*=0.84; *Epachthosaurus*=0.80; *Giraffatitan*=0.64; *Opisthocoelicaudia*=0.85; *Rapetosaurus*=1.27; *Venenosaurus*=0.69; *Wintonotitan*=0.74 (note that other taxa can be scored for this character, but exact values are unknown).

C378. Metacarpals, metacarpal V, ratio of proximal end long axis diameter to that of metacarpal IV: equal or smaller (0); larger than that for metacarpal IV (1) (Poropat *et al.*[8]; Upchurch and Mannion[53]).

C379. Metacarpals, metacarpal V with a medially biased flange-like swelling along proximal half of ventral surface: absent (0); present (1) (Upchurch and Mannion[53]; Mannion and Calvo[89]; Apesteguía[97]).

C380. Ilium, ratio of dorsoventral height of iliac blade above pubic peduncle to anteroposterior length of ilium: less than 0.35 (0); 0.35 or greater (1) (Upchurch and Mannion[53]; Tschopp *et al.*[63]).

C381. Ilium, projected line (chord) connecting articular surfaces of ischiadic and pubic processes: passes ventral to ventral margin of postacetabular portion of ilium (0); passes through or dorsal to ventral edge of postacetabular portion of ilium (1) (Upchurch[57]).

C382. Ilium, orientation of the pubic peduncle with respect to the long axis of the ilium: anteriorly deflected (0); perpendicular (1) (Salgado *et al.*[81]).

C383. Ilium, protuberance on the lateral surface of the ischiadic articulation: absent (0); present (1) (new character: based on Poropat *et al.*[2]; Borsuk-Białynicka[55]).

C384. Pubis, proximodistally oriented ridge on lateral surface of blade, separated from the anterior margin of the pubis by a longitudinal groove: absent (0) present (1) (Powell[39]; Upchurch and Mannion[53]; Otero[94]; Salgado and Carvalho[98]).

C385. Pubis, distal end transversely expanded along lateral surface relative to shaft: present (0); absent (laminar distal blade) (1) (Upchurch and Mannion[53]; Curry Rogers[61]).

C386. Ischium, iliac articular surface, anteroposterior length to mediolateral width ratio: 1.0 or greater (0); less than 1.0 (1) (Upchurch and Mannion[53]).

C387. Ischium, ridge (for attachment of M. flexor tibialis internus III) on lateral surface of the lower part of the proximal plate/proximal portion of shaft, close to the posterior/dorsal margin of ischium: associated with parallel groove, posterior/dorsal to ridge (0); groove absent (1) (Upchurch and Mannion[53]; D'Emic[80]; Sereno *et al.*[99]).

C388. Femur, femoral head, projection: directed medially (0); directed dorsomedially (1) (Upchurch *et al.*[52]; Upchurch and Mannion[53]; Curry Rogers[61]).

C389. Femur, ratio of mediolateral breadth of tibial condyle to breadth of fibular condyle: greater than 0.8 (0); 0.8 or less (1) (Wilson[44]; Upchurch and Mannion[53]; polarity reversed here).

C390. Femur, shape of distal condyles: articular surface restricted to distal portion of femur (0); expanded onto anterior portion of femoral shaft (1) (Wilson[44]; Wilson and Carrano[100]).

C391. Tibia to femur length ratio: less than 0.6 (0); 0.6 or greater (1) (Upchurch and Mannion[53]; Gauthier[67]).

Quantitative values: *Omeisaurus*=0.63–0.68; *Mamenchisaurus*=0.57; *Camarasaurus*=0.60; *Apatosaurus*=0.62; *Diplodocus*=0.68; *Cedarosaurus*=0.63; *Chubutisaurus*=0.59; *Diamantinasaurus*=0.59; *Dongbeititan*=0.56; *Epachthosaurus*=0.64; *Euhelopus*=0.63; “French *Bothriospondylus*”=0.60; *Opisthocoelicaudia*=0.58; *Phuwiangosaurus*=0.62; *Tangvayosaurus*=0.58; *Tastavinsaurus*=0.57 (note that other taxa can be scored for this character, but exact values are unknown).

C392. Fibula, articular surface of lateral trochanter: not visible in anterior view (0); visible in anterior view (1) (new character).

C393. Fibula, distal end mediolateral width to anteroposterior width ratio: 0.8 or less (0); greater than 0.8 (1) (Upchurch and Mannion[53]).

C394. Fibula, distal end profile: elliptical or semicircular (with a straight medial margin) (0); subtriangular (with a rounded or sharper apex projecting laterally or anterolaterally where flattened anterolateral and posterolateral margins meet) (1) (Upchurch and Mannion[53]).

C395. Metatarsals, metatarsal V, proximal end: dorsoventrally expanded relative to shaft, with a domed dorsal margin (0); not expanded relative to shaft (1) (Upchurch and Mannion[53]).

C396. Metatarsals, metatarsal V, tubercle or ridge on ventral surface, at approximately midlength, equidistant from the medial and lateral margins: absent (0); present (1) (Upchurch and Mannion[53]).

C397. Pedal digit I, proximal articular surface of ungual (phalanx I-2): perpendicular to long axis of ungual (0); bevelled so that the proximal articular surface faces proximolaterally and thus lies at a distinct angle to the long axis of the ungual (1) (Upchurch and Mannion[53]; Wilson and Upchurch[101]).

Methods

Characters 11, 14, 15, 27, 40, 51, 104, 122, 147, 148, 177, 195, 205 and 259 were treated as ordered multistate characters. The data matrix was then analysed using the ‘Stabilize Consensus’ option in the ‘New Technology Search’ in TNT vs. 1.1 (Goloboff *et al.*[102]). Searches were carried out using sectorial searches, drift, and tree fusing, with the consensus stabilized five times, prior to using the resultant trees as the starting trees for a ‘Traditional Search’ using Tree Bisection-Reconstruction. This resulted in 456,528 MPTs of 1,544 steps, with little resolution.

As such, these analyses were re-run with ten unstable (identified through the Pruned Trees option in TNT) and highly incomplete taxa (*Astrophocaudia*, *Australodocus*, *Brontomerus*, *Fukuititan*, *Fusuisaurus*, *Liubangosaurus*, *Malarguesaurus*, *Mongolosaurus*, *Sonorasaurus*, *Tendaguria*) excluded *a priori*. This resulted in 12 MPTs of 1,508 steps and produced a largely resolved strict consensus tree (Figure S1), with polytomies restricted to: (1) a clade within Brachiosauridae; (2) the base of Titanosauria; and (3) several lithostrotian taxa outside of Saltasauridae. The agreement subtree (i.e. the largest fully resolved topology common to all MPTs) was then calculated in TNT, requiring the *a posteriori* pruning of four further taxa (*Aeolosaurus*, *Rapetosaurus*, *Ruyangosaurus*, *Venenosaurus*) (Figure S2).

We also analysed this data matrix using implied weights in TNT vs. 1.1 with a *k*-value of 3 (Goloboff *et al.*[103]), following the same protocol as our equally weighted analysis. This resulted in 54 MPTs of 160.73 steps, with a fairly resolved tree (Figure S3), including a fully resolved Lithostrotia, with polytomies restricted to: (1) derived brachiosaurids; (2) the base of Euhelopodidae; and (3) the base of Titanosauria. The agreement subtree required the *a posteriori* pruning of five taxa (*Abydosaurus*, *Angolatitan*, *Daxiatitan*, *Huanghetitan*, *Liubangosaurus*) (Figure S4).

In both sets of analyses, symmetric resampling was used to generate the relative frequencies of groups of taxa in the trees, expressed as a GC value, using 5,000 replicates in TNT, with all

tree searches run using a ‘Traditional Search’ with TBR[104]. GC values were also recalculated for the taxa in the agreement subtrees. We also calculated Bremer supports for our equal weights analysis.

Results

Equal weights analysis

Bremer supports vary from 1 to 3 throughout the tree, with the best supported clades including Euhelopodidae and Lithostrotia. GC values are in general low, with only 15 nodes displaying values higher than zero (Figure S5), with a slight increase in numbers of nodes (16) and values (Figure S6) for the taxa in our agreement subtree. Most of the tree topology is largely congruent with that presented in previous iterations of this data [2,8,35,36], and here we focus on results pertaining to Titanosauria and the Australian taxa. *Wintonotitan* is recovered as a non-titanosaurian somphospondylan, just outside of the titanosaur radiation, similar to its position in previous versions of this data matrix[2,8,35]. In contrast to previous analyses, the East Asian taxa *Baotianmansaurus* and *Dongyangosaurus* are not members of Opisthocoelicaudiinae, but instead form a clade of basal titanosaurs, outside of Lithostrotia. Similarly, whereas *Diamantinasaurus* was recovered as an opisthocoelicaudine by Poropat *et al.*[2], here it is placed as a non-lithostrotian titanosaur, forming the clade *Savannasaurus* + (*Diamantinasaurus* + AODF 836) (Bremer support=2). As in previous analyses, *Daxiatitan* and *Xianshanosaurus* form a clade just outside Lithostrotia. The placement of *Epachthosaurus* within Lithostrotia is congruent with the analyses of most authors (e.g. González Riga *et al.*[54]; Curry Rogers[61]; Salgado *et al.*[81]), and contrasts with the basal titanosaurian position recovered by some (e.g. Martínez *et al.*[40]; Upchurch *et al.*[52]; Carballido *et al.*[60]). It forms the clade *Muyelensaurus* + (*Epachthosaurus* + *Futalognkosaurus*). Although its exact placement is uncertain, *Aeolosaurus* is recovered as a non-saltasaurid lithostrotian. Nemegtosauridae is recovered as the sister taxon to *Isisaurus* + Saltasauridae. Nemegtosauridae comprises *Nemegtosaurus* + *Tapuiasaurus*, supporting the conclusions of Zaher *et al.*[51] regarding the affinities of the latter taxon. Although pruned to produce our agreement subtree, *Rapetosaurus* is also recovered as a nemegtosaurid if we exclude *Tapuiasaurus a priori*, supporting previous work[44,61]. *Alamosaurus* + *Jiangshanosaurus* forms a clade, as in all previous versions of this data matrix, but this is the sister taxon to *Opisthocoelicaudia*, rather than *Saltasaurus* (which lies outside of this clade), contrasting with previous iterations. Below, we provide recovered synapomorphies for the clade of Australian titanosaurs, through character mapping in TNT. Each character number is placed in parentheses.

The clade of *Savannasaurus* + (*Diamantinasaurus* + AODF 836) is characterized by:

- Middle–posterior dorsal centra, ventral surface transversely concave, between ventrolateral ridges (333).
- Middle–posterior dorsal neural spines, postspinal lamina extends ventral to the neural spine, beyond the postzygapophyseal articular surfaces (343).
- Radius, maximum diameter of the proximal end divided by proximodistal length is 0.3 or greater (45).

- Scapula, ventrolateral margin of acromion forms an anteroposteriorly concave region posterior to glenoid, followed by a flattened area (358) [note that this might instead be an autapomorphy of *Diamantinasaurus*].

- Pubis, anterior margin of distal end strongly concave in lateral view, such that the distal end forms a prominent, anteriorly expanded boot (251).

Implied weights analysis

GC values are in general low, with only 16 nodes displaying values higher than zero (Figure S7), with a slight increase in numbers of nodes (18) and values (Figure S8) for the taxa in our agreement subtree. In general, the overall topology (Figure S4) is not too dissimilar to that presented in Mannion *et al.*[35], and we focus primarily on titanosaur interrelationships and the position of the Australian taxa. Novel results outside of these parts of the tree include: (1) *Haestasaurus* is the sister taxon to *Janenschia*, at the base of Macronaria, rather than a titanosaur, supporting the conclusions of Upchurch *et al.*[36]; (2) the recovery of a diverse Euhelopodidae that closely matches the equal weights topology, although *Fusuisaurus*, *Liubangosaurus*, '*Huanghetitan*' *ruyangensis* and *Mongolosaurus* are also placed in this clade; (3) *Australodocus* occupies a similar relative position in the tree as that recovered by Mannion *et al.*[35], but the change in placement of *Andesaurus* (see below) means that the former is now a non-titanosaurian somphospondylan. In contrast to Mannion *et al.* [35], there is no longer an Andesauroidea composed of taxa generally regarded as non-titanosaurs (e.g. *Chubutisaurus*, *Sauroposeidon*, *Wintonotitan*). Instead these taxa are recovered as non-titanosaurian somphospondylans. *Ruyangosaurus* and *Daxiatitan* are recovered as non-lithostrotian titanosaurs, more derived than *Andesaurus*. All other titanosaurs are lithostrotians, with *Xianshanosaurus* positioned as the sister taxon to *Malawisaurus*. *Aeolosaurus* + *Muyelensaurus* forms a basal lithostrotian clade that is the sister taxon of Nemegtosauridae + all remaining titanosaurs. Nemegtosauridae comprises *Rapetosaurus* + (*Nemegtosaurus* + *Tapuiasaurus*). *Futalognkosaurus* is recovered at the base of the clade comprising the remaining titanosaurs, with the latter forming two clades. One consists of (*Baotianmansaurus* + *Dongyangosaurus*) + (*Savannasaurus* + (*Diamantinasaurus* + AODF 836)), and the other comprises *Epachthosaurus* + (*Isisaurus* + Saltasauridae). The interrelationships of *Isisaurus* + Saltasauridae do not differ from our equal weights analysis. Below, we provide recovered synapomorphies based on our implied weights analysis for the clade of Australian titanosaurs, as well as the clade they form with *Baotianmansaurus* + *Dongyangosaurus*, through character mapping in TNT. Each character number is placed in parentheses.

<u>The clade of (*Baotianmansaurus* + *Dongyangosaurus*) + (*Savannasaurus* + (*Diamantinasaurus* + AODF 836)) is characterized by:</u>

- Middle-posterior dorsal neural arches, posterior centrodiapophyseal lamina (PCDL) has an unexpanded ventral tip (151 [reversal]).

- Middle-posterior dorsal neural spines, midline prespinal lamina (forming distinct ridge) along proximal (lower) half of neural spine absent (168).

- Middle–posterior dorsal neural arches, neural canal in anterior view enclosed in a deep fossa in the dorsal surface of the centrum (i.e. much of the canal is enclosed laterally by pedicels that are part of the centrum rather than the neural arch) (338).

- Middle–posterior dorsal neural spines (single, not bifid), SPRLs remain separate or converge at about spine mid-height (or above) to form a dorsally restricted median composite lamina (SPRF well-developed and occupies the ventral half of the anterior spine surface) (342 [reversal]).

- Middle–posterior dorsal neural spines, SPDL does not bifurcate at its dorsal end to create a SPDL-F (345 [reversal]).

- Anterior caudal centra, posterior articular surface flat or concave throughout series (27, 177).

The clade of *Savannasaurus* + (*Diamantinasaurus* + AODF 836) is characterized by:

- Middle–posterior dorsal neural spines, anterior spinodiapophyseal lamina (aSPDL) absent (344 [reversal]).

- Pubis, anterior margin of distal end strongly concave in lateral view, such that the distal end forms a prominent, anteriorly expanded boot (251).

IV. Biogeographic analyses

<u>Data sets and methodological approach</u>

In order to reconstruct the biogeographic history of macronarian sauropods, we have applied the maximum likelihood approach implemented in BioGeoBEARS[105,106] in R[107]. The R script used to run the analyses is provided in the file 'PoropatetalBioGeoBEARSRscript.txt' (N.B. readers wishing to replicate the analyses will need to change the names of some files in this script if, for example, they wish to run the 'relaxed' or 'harsh' versions of our dispersal multiplier matrices).

Calibrated trees

A BioGeoBEARS analysis requires a time-calibrated phylogeny. BioGeoBEARS cannot deal with phylogenetic topologies that contain polytomies (as is also the case for nearly all phylogenetic biogeographic methods). Given that our pruned phylogenetic analysis yielded 12 MPTs, we consider it impractical to run divergence time and biogeographic analyses on every topology. In order to reduce the time required for analyses, the time-calibrated trees are based on the agreement subtree resulting from our cladistic analysis (Figure S2). We have only included macronarian taxa in our biogeographic analysis because this clade is thoroughly sampled for Late Jurassic and Early Cretaceous taxa, whereas other sauropods (i.e. diplodocoids and some basal eusauropods) are only represented by a few taxa for the purposes of outgroup comparison. The resulting phylogeny contains 48 out of the original 62 macronarian taxa included in the data set employed in this study.

Our data set suffers from a common problem that affects the dating of many extinct vertebrate taxa (especially those recovered from non-marine formations). Such taxa are often known from only a single individual, or single locality, so that they represent point occurrences rather than a taxon's true stratigraphic range. Moreover, limits on the dating of many terrestrial formations mean that the age of a given point occurrence might not be known more precisely than, for example, 'Cenomanian–Santonian'. This problem is clearly present in our sauropod data set. Of the 48 taxa included in our sample of macronarians, only *Camarasaurus* and *Alamosaurus* are regarded as having stratigraphic ranges based on numerous specimens from multiple horizons. The remaining 46 taxa are typically known from a single individual or small number of specimens, whose geological age can only be determined to sub-stage level at best (and often much less precisely – see Table S1). Recent studies have dealt with this issue by regarding each taxon as occurring at the mid-point of its possible age range[137,138], and we have applied this assumption here in our 'srpdmidtree.newick' tree. However, the ages of fossils provide only a minimum divergence time for each set of sister taxa, so it is likely that the true divergence times were older than those implied by a simple reading of the fossil record. We have therefore created a 'maximum' divergence time tree (see the file named 'srpdmaxtree.newick'), using the assumption that point occurrence taxa first appear at the oldest point within the uncertainty of their age estimate. Thus, for example, *Xianshanosaurus* has been dated as occurring at some point during the Cenomanian–Santonian (Table S1): this gives a mid-point age of 92.1 Ma and a maximum age of 100.5 Ma (i.e. the Albian–Cenomanian boundary). N.B. here 'maximum' age, refers to the oldest date possible for a taxon within the currently accepted uncertainty of its age: it does not refer to the true origination time of that taxon that would be observed if we had a complete fossil record.

Bapst[108,109] has proposed the cal3 method, which uses estimates of the rates of speciation, extinction and sampling for the clade under investigation, to generate more accurate estimates

of the true divergence times of lineages. Although simulations suggest that this approach is often superior to previously applied methods, we have not used it here. This is because it is difficult to obtain well-supported estimates of the sampling rates for most vertebrate groups (with the exception of Cenozoic mammals), because such data sets are usually dominated by many point occurrences (see http://nemagraptus.blogspot.co.uk/2013/06/sampling-rates-and-dealing-with-fossil.html).

Our two time-calibrated trees were created using the R package strap[110]. In order to convert the Stage-based ages of taxa into absolute ages, we have used the 2015 Chronostratigraphic Timescale of the International Commission on Stratigraphy[111]. For both trees, we used the 'equal' method for re-distributing the node ages of adjacent zero-length branches[112], implemented in a slightly modified form in strap (see discussion of this issue in Bell and Lloyd[110]). The 'equal' method in strap also requires a root length ('rLen') to be defined. Here we have assumed that Macronaria diverged from other sauropod lineages by the beginning of the Bajocian (170.3 Ma), which also represents the start of our first palaeogeographic time slice (see below). This assumption is based on the recommendation of Bell and Lloyd[110], who suggested that the root length for the ingroup clade should be based on the first appearance date of the nearest outgroup, provided that this outgroup is older than the root node of the ingroup. In the case of our data set, Diplodocoidea is the nearest outgroup, but it appears slightly after the first known macronarian (i.e. in the Kimmeridgian). Thus, we have used taxa such as *Atlasaurus* and *Lapparentosaurus*, from the Bathonian–Callovian and Bathonian respectively (Table S1), to support the view that macronarians are likely to have been present by the beginning of the Bajocian. This seems reasonable, given that the presence of a late Oxfordian brachiosaurid (French "*Bothriospondylus*") means that only approximately 10 million years is allowed for the clade to diversify into the lineages leading to *Camarasaurus*, brachiosaurids and somphospondylans. The root length for each of the two trees is therefore defined as the time (in millions of years) from the start of the Bajocian to the first appearance of the oldest macronarian in our data set (i.e. French "*Bothriospondylus*").

Geographic areas

Each terminal sauropod taxon in the time-calibrated trees has been assigned to one of seven palaeocontinental areas (N.B. there are no widespread taxa that occupy two or more areas). These areas and their abbreviations are: A, Asia (excluding India); E, Europe; F, Africa; I, Indo-Madagascar; N, North America; S, South America; U, Australia. The file containing information on the geographic ranges of terminal taxa is 'srpdgeogranges.txt'. The maximum range size is set to 7: this means that the ancestral range estimations in BioGeoBEARS, for a given node or lineage, can include all seven specified areas if required.

Time-stratified analyses and dispersal multiplier matrices

BioGeoBEARS allows stratified analyses in which the dispersal probabilities between areas can vary from one time-slice to the next[105,106]. This allows researchers to use palaeogeographic data to constrain or otherwise inform the analysis. The probability of dispersing from one area to another is modified in the 'dispersal multiplier matrix', with one matrix per time slice in a time-stratified analysis (see below).

Here we have created 22 time slices in order to reflect palaeogeographic events that occurred during the Late Jurassic and Cretaceous. The end of the Cretaceous (time slice 22) is treated as time 0, so all dates that define time-slice boundaries are in millions of years prior to that

Table S1. Ages of sauropod taxa included in this study. Taxa are ordered stratigraphically.

Taxon	Continent	Formation	Stage (s)
Rapetosaurus krausei	Madagascar	Maevarano	Maastrichtian[113,114]
Alamosaurus sanjuanensis	North America	Ojo Alamo/North Horn	Maastrichtian[115]
Isisaurus colberti	India	Lameta	Maastrichtian[116]
Nemegtosaurus mongoliensis	Asia	Nemegt	L Campanian–E Maastrichtian[117]
Opisthocoelicaudia skarzynskii	Asia	Nemegt	L Campanian–E Maastrichtian[117]
Saltasaurus loricatus	South America	Lecho	L Campanian–Maastrichtian[118]
Aeolosaurus rionegrinus	South America	Angostura Colorada	L Campanian–Maastrichtian[39]
Muyelensaurus pecheni	South America	Plottier	L Coniacian–Santonian[119]
Futalognkosaurus dukei	South America	Portezuelo	L Turonian–E Coniacian[54,120,121]
Malarguesaurus florenciae	South America	Portezuelo	L Turonian–E Coniacian[54,120,121]
Angolatitan adamastor	Africa	Itombe	L Turonian[122,123]
Epachthosaurus sciuttoi	South America	Bajo Barreal	L Cenomanian–E Turonian[124]
Erketu ellisoni	Asia	Baynshiree	Cenomanian–Santonian[125]
Huanghetitan ruyangensis	Asia	Mangchuan	Cenomanian–Santonian[126]
Ruyangosaurus giganteus	Asia	Mangchuan	Cenomanian–Santonian[127]
Xianshanosaurus shijiagouensis	Asia	Mangchuan	Cenomanian–Santonian[128]
Baotianmansaurus henanensis	Asia	Gaogou	Cenomanian–Turonian[129,130]
Dongyangosaurus sinensis	Asia	Jinhua	Cenomanian–Turonian[131]
Jiangshanosaurus lixianensis	Asia	Jinhua	Cenomanian–Turonian[131]
Diamantinasaurus matildae	Australia	Winton	Cenomanian[132,133]
Savannasaurus elliottorum gen. et sp. nov.	Australia	Winton	Cenomanian[132,133]
Wintonotitan wattsi	Australia	Winton	Cenomanian[132,133]
Chubutisaurus insignis	South America	Cerro Barcino	Cenomanian[134,135]
Andesaurus delgadoi	South America	Rio Limay	E Cenomanian[120]
Sonorasaurus thompsoni	North America	Turney Ranch	L Albian–E Cenomanian[136]

Taxon	Continent	Formation	Stage (s)
Abydosaurus mcintoshi	North America	Cedar Mountain (Mussentuchit Member)	L Albian[137]
“Cloverly titanosauriform”	North America	Cloverly	L Albian[138]
Gobititan shenzhouensis	Asia	Middle Grey Unit (Xinminbao Group)	Albian[139,140]
Astrophocaudia slaughteri	North America	Paluxy	E Albian[56,141]
Ligabuesaurus lilloi	South America	Lohan Cura	L Aptian–E Albian[120]
Paluxysaurus jonesi	North America	Twin Mountains	L Aptian–E Albian[142]
Mongolosaurus haplodon	Asia	On Gong	?Aptian–Albian[143]
Brontomerus mcintoshi	North America	Cedar Mountain (Ruby Ranch Member)	Aptian–Albian[144,145]
Sauroposeidon proteles	North America	Antlers	Aptian–Albian[146,147]
Nigersaurus taqueti	Africa	Elhraz	Aptian–Albian[148,149]
Qiaowanlong kangxii	Asia	Xinminpu	Aptian–Albian[150]
Tangvayosaurus hoffeti	Asia	Grès supérieurs	Aptian–Albian[151]
Daxiatitan binglingi	Asia	Hekou Group	Aptian[152]
Huanghetitan liujiaxiaensis	Asia	Hekou Group	Aptian[153]
Fusuisaurus zhaoi	Asia	Napai	Aptian[154,155]
Liubangosaurus hei	Asia	Napai	Aptian[155,156]
Malawisaurus dixeyi	Africa	Dinosaur Beds	Aptian[157,158]
Tapuiasaurus macedoi	South America	Quiricó	Aptian[51]
Tastavinsaurus sanzi	Europe	Xert	E Aptian[159]
Cedarosaurus weiskopfae	North America	Cedar Mountain (Yellow Cat Member) & Paluxy	Barremian–E Albian[56,141,160]
Euhelopus zdanskyi	Asia	Mengyin	Barremian–Aptian[101]
Phuwiangosaurus sirindhornae	Asia	Sao Khua	Barremian–Aptian[161]
Venenosaurus dicrocei	North America	Cedar Mountain	Barremian–Aptian[162]
Dongbeititan dongi	Asia	Yixian	Barremian[163,164]
Fukuititan nipponensis	Asia	Kitadani	Barremian[165]
Haestasaurus becklesii	Europe	Hastings Group	L Berriasian–Valanginian[166]
Aragosaurus ischiaticus	Europe	Villar del Arzobispo	L Tithonian–M Berriasian[37]
Galveosaurus herreroi	Europe	Villar del Arzobispo	L Tithonian–M Berriasian[167]
Australodocus bohetii	Africa	Tendaguru (Upper Dinosaur Member)	Tithonian[168,169]

Taxon	Continent	Formation	Stage (s)
Janenschia robusta	Africa	Tendaguru (Upper Dinosaur Member)	Tithonian[169,170]
Tendaguria tanzaniensis	Africa	Tendaguru (Upper Dinosaur Member)	Tithonian[169,170]
HMN MB.R.2091.1–30	Africa	Tendaguru (Upper Dinosaur Member)	Tithonian[169,170]
Giraffatitan brancai	Africa	Tendaguru (Middle and Upper Dinosaur Members)	L Kimmeridgian–Tithonian[169,170]
Lusotitan atalaiensis	Europe	Lourinhã	L Kimmeridgian–E Tithonian[35]
Brachiosaurus altithorax	North America	Morrison	Kimmeridgian–Tithonian[171]
Camarasaurus spp.	North America	Morrison	Kimmeridgian–Tithonian[171]
Apatosaurus spp.	North America	Morrison	Kimmeridgian–Tithonian[171]
Diplodocus spp.	North America	Morrison	Kimmeridgian–Tithonian[171]
Tehuelchesaurus benitezii	South America	Cañadón Calcareo	Kimmeridgian–E Tithonian[172]
Europasaurus holgeri	Europe	Bed 93, Langenberg Quarry	M Kimmeridgian[59,173,174]
French '*Bothriospondylus*'	Europe	Belvoye Quarry	L Oxfordian[175,176]
Mamenchisaurus spp.	Asia	Dashanpu (Shangshaximiao), Shishugou	Oxfordian–Tithonian[177,178]
Omeisaurus spp.	Asia	Dashanpu (Xiashaximiao & Shangshaximiao)	Bathonian–Oxfordian[179,180]
Shunosaurus lii	Asia	Dashanpu (Xiashaximiao)	Bathonian–Callovian[181,182]
Atlasaurus imelakei	Africa	Tilougguit	Bathonian–Callovian[183,184]
Lapparentosaurus madagascariensis	Madagascar	Isalo III	Bathonian[185,186]

point. When constraining dispersal based on palaeogeographic data, we have made the following assumptions:

(1) We assume that trans-oceanic dispersal was a very low probability event for sauropods. It is always difficult to assess the dispersal abilities of extinct organisms, especially those with body plans and/or physiologies that are not fully represented among living descendants or analogous taxa. However, large-bodied terrestrial mammals have been estimated to have very limited over-water dispersal abilities (e.g. Lawver *et al.*[187] and references therein). Moreover, the observation that dinosaurs display statistically significant patterns indicative of

continental-scale vicariance (e.g. Upchurch *et al.*[188], Ezcurra and Agnolin[189]) also suggests that these animals did not disperse across marine barriers very frequently or, at least, not frequently enough to overprint the vicariance patterns (see also Csiki-Sava *et al.*[190]). We therefore propose that the assumption of no trans-oceanic dispersal in sauropods is reasonable given our current knowledge of these animals. Therefore, when two of our continental areas are completely separated by ocean according to palaeogeographic maps or other geological data, the probability of jump dispersal between these areas is set to 0.000001. We use 0.000001 rather than 0 for two reasons. First, this is a pragmatic measure because BioGeoBEARS analyses sometimes crash or 'hang' when dispersal multiplier values of 0 are used (see http://phylo.wikidot.com/biogeobears). Second, a value of 0.000001 models the scenario that trans-oceanic dispersal of sauropods was not completely impossible, but might have occurred very infrequently. When land areas are separated by shallow and potentially episodic epicontinental seas, or where island chains might have provided 'stepping-stones' for dispersal, we evaluate the case for allowing dispersal to occur (see assumption 8 and the descriptions of each time-slice below).

(2) Two land areas are assumed to be completely isolated from each other by an oceanic barrier at approximately the time of the onset of sea floor spreading between those areas (provided the proposed oceanic crust completely severs all connections between the continental crust of the two landmasses). However, we also take into account other geological and palaeontological evidence pertinent to the nature of an oceanic barrier. For example, support for the disconnection of two continental regions can be provided by the onset of dispersal of marine taxa between two previously separate areas of ocean (as occurred when the South and Central Atlantic were connected during the mid-Cretaceous by the final separation of Africa and South America [see time slice 12]).

(3) Although climatic zones might have hindered or promoted dispersal between areas, we have not built this information into our dispersal constraints. It is probable that sauropods could not tolerate prolonged and extreme cold, and this might have meant that they generally avoided high latitudes. Indeed, sauropods appear to be absent from higher latitudes in the northern hemisphere during the Late Cretaceous, though this might reflect historical and biotic factors, such as the dominance of ornithischians[191,192], rather than climate. Nevertheless, generally warm conditions during the Cretaceous (e.g. Hay[193]), coupled with the observation that sauropod remains are known from a wide variety of terrestrial and freshwater environments[194] with a palaeolatitudinal range of 66° N–63.5° S[7,195], suggest that it would be premature to impose stringent constraints on this group's dispersal based on latitudinal climatic zones given our current knowledge.

(4) Dispersal is only allowed between 'adjacent' areas. For example, if North America is in contact with Europe, and the latter is in contact with Asia, but Asia is not in direct contact with North America, then any taxon dispersing between North America and Asia would have to do so via Europe. This is consistent with the prohibition on trans-oceanic dispersal (see assumption '(1)' above), which would severely restrict direct dispersal between North America and Asia in this situation.

(5) All dispersal probabilities between two areas are symmetrical (i.e. if dispersal from North America to Europe is allowed, then dispersal from Europe to North America is also allowed with the same level of probability). Asymmetrical dispersal probabilities can be modelled in BioGeoBEARS, and are certainly plausible in reality. For example, Sanmartin and Ronquist[196] showed that prevailing wind currents have meant that seed dispersal from

Australia to New Zealand has been more prevalent than vice versa. However, we currently have no strong grounds for proposing asymmetrical dispersal of terrestrial taxa between Mesozoic continental areas. Russell[197] employed one of the principles of 'dispersal biogeography' that states that taxa will tend to disperse from a larger geographic area to a smaller one (see also Weishampel and Jianu[198]), but we suggest that this is a phenomenon that it would be better to test *a posteriori*, than assume *a priori*. Even if the assumption of asymmetrical dispersal probabilities was desirable, it is not clear what values should be assigned to represent such a process in the case of terrestrial dinosaurs dispersing from one continent to another.

(6) None of the sauropods in our analysis occur in Antarctica. Therefore, when two of our designated areas are in contact with each other only via Antarctica (e.g. South America and Indo-Madagascar during the Early Cretaceous), we treat them as being 'adjacent' (see assumption ' (4)' above).

(7) The European area is difficult to model realistically because, during much of the Jurassic and Cretaceous, it comprised a series of islands (e.g. Upchurch *et al.*[188]; Csiki-Sava *et al.*[190], Smith *et al.*[199]; Tyson and Funnell[200]; and Bardet *et al.*[201] and references therein). Narrow and shallow epicontinental seas separated these islands and, at present, there is insufficient information available to allow a detailed model of the sequence and timing of the connections and disconnections between these islands (although resolution is improving, especially for the Late Cretaceous – see Csiki-Sava *et al.*[190]). In any case, even if it were possible to capture a more detailed palaeogeographic history of Europe, there are so few European sauropods in our phylogeny that further division of this area into smaller units is likely to result in insufficient data to resolve their biogeographic relationships. Here, therefore, we treat Europe as a single area for pragmatic reasons, pending the development of more detailed palaeogeographic and biogeographic data sets.

(8) Dispersal multiplier values are typically set to 0.000001 (effectively no dispersal allowed between the two areas concerned) when there is clear and unequivocal evidence for a barrier to terrestrial dispersal, or 1 (dispersal is allowed between the two areas, with the maximum probability used by the analytical model) when there is strong evidence that two areas were connected. In several cases, such as when palaeogeographic events are temporally or spatially poorly constrained, epicontinental seas are shallow and potentially ephemeral, and/or the presence of a barrier has been inferred mainly on palaeontological (rather than geological/geophysical) data, we have used an intermediate value of 0.5. The version of our dispersal constraints, which includes these 0.5 values, is referred to here as the 'Starting' model and can be found in the file 'srpddispersalmultipliersstarting.txt'. In order to explore the sensitivity of our results to this particular set of assumptions about dispersal, we have also applied two more extreme versions of the dispersal multiplier probabilities. In the 'Relaxed' model, all 0.5 values are reset to 1.0, so that dispersal across the 'doubtful' barriers is allowed with the same probability as dispersal between adjacent land areas (see the file 'srpddispersalmultipliersrelaxed.txt'). In the 'Harsh' model, the 0.5 values are set to 0.000001, so that effectively no dispersal is allowed across the 'doubtful' barriers (see the file 'srpddispersalmultipliersharsh.txt').

(9) We have not used an 'areas allowed' matrix to exclude one or more areas during any of the time slices. This is because we are dealing with continental areas that were present throughout the period under investigation.

(10) We have not imposed any assumptions about the probability of dispersal events based on the relative distances between areas (apart from the 'adjacent areas' rule set out in assumption 4).

The time-slice constraints on dispersal (Tables S2–23), and the palaeogeographic data that justify them, are briefly outlined below and summarised in Table S24. Note that all constraints on dispersal defined in a given time slice are retained in subsequent time slices except where stated otherwise. The timescale used here is the same as that employed when producing the time-calibrated phylogenies (see above). The boundaries between time slices are defined in the 'mid' and 'max' versions of the file 'srpdtimeperiods.txt'. The 'mid' version of this time periods file is identical to the 'max' version, except that somes dates in the former have been reduced by 0.0001 million years (e.g. 3.05 Ma. becomes 3.0499 Ma.). This has been done because several of the nodes in the srpdmidtree were exactly the same age as the boundary between two time slices, making it impossible for BioGeoBEARS to temporally-partition the tree (the algorithm cannot determine which of the two time slices should be allocated the node).

Time slice 1: 104.3–91.3 million years prior to the K/Pg boundary (= Bajocian–Oxfordian [170.3–157.3 Ma])

During much of the early Mesozoic, Laurasia and Gondwana were joined to form Pangaea, but the Tethys Ocean created a barrier between Europe and Asia to the North, and Africa and East Gondwana continental areas to the south[199,202,203]. However, a land connection existed between North America and Africa+South America during the Triassic and Early Jurassic[187,199,202-204]. According to Bardet *et al.*[201], the Hispanic corridor (separating northwest Africa and South America from North America) potentially dates back to the Pliensbachian, but this marine dispersal route between Tethys and eastern Panthalassa was probably sporadic until the opening of the Gulf of Mexico in approximately the Callovian (see below).

Bardet *et al.*[201] (and references therein) noted the presence of the Viking Corridor during the Early Jurassic (a narrow area of epicontinental sea corresponding approximately in position with the future North Atlantic rift), which might have hindered North America–Europe terrestrial dispersal at this time. However, the palaeogeographic reconstructions of Scotese[202] and Smith *et al.*[199] indicate that land connections between North America and at least some portions of Europe existed during the Middle Jurassic (see also Golonka *et al.*[205]).

The Turgai Sea (also known as the Obik Sea[206] or Uralian Sea[207]), or other areas of ocean in the vicinity of the Russian Basin, potentially produced a longitudinally oriented marine barrier separating Europe from Central (and therefore also East) Asia during the middle and Late Jurassic[199,202]. These areas of epicontinental sea have been linked to geographic isolation of Chinese and other East Asian faunas from approximately the Bajocian–Bathonian onwards[62,101,188,197,206,207], although the palaeocoastline reconstructions of Smith *et al.*[199] depict a marine barrier appearing for the first time only in the Callovian. Unfortunately, detailed information on the exact nature of this barrier during the Jurassic is lacking (unlike the Cretaceous, see below). Consequently, the effectiveness of this area of epicontinental sea as a barrier to terrestrial dispersal could have been frequently modified by relatively minor fluctuations in sea level. Such fluctuations might have produced ephemeral landbridges between Europe and Central Asia that would be difficult to detect because of limits on the temporal resolution of palaeogeographic reconstructions.

The Mongol-Okhotsk Ocean, between Siberia-Kazakhstan and Mongolia, potentially produced another barrier to dispersal from Europe to East Asia (via Central Asia) and vice versa during the Middle and Late Jurassic[62]. The timing of continental collision associated with the onset of closure of the Mongol-Okhotsk Ocean is debated. Initially, it was proposed that an area of ocean separated Central and East Asia during the Jurassic, with continental collision starting in the earliest Cretaceous and resulting in elimination of the Mongol-Okhotsk Ocean by the mid-Cretaceous[208,209]. However, more recent work suggests that the initial collision was somewhat earlier, occurring in the Late Jurassic[210] or even the late Middle Jurassic[211]. Seton *et al.*[212] also supported a Jurassic date for the initial collision between the Siberian and Mongolian blocks, and suggested that the Mongol-Okhotsk Ocean had closed by 150 Ma. Yang *et al.*[213] suggested that by the earliest Cretaceous, oceanic conditions only remained in the easternmost region of the former Mongol-Okhotsk Ocean. Here, therefore, we do not consider the Mongol-Okhotsk Ocean to be an effective barrier to terrestrial dispersal during or after the Middle Jurassic.

Gondwana appears to have been a coherent landmass during the Middle Jurassic[199,202,203], but fragmentation events might have had an impact on dispersal across this supercontinent as early as the Late Jurassic (see below).

In this time slice, therefore, we allow dispersal between all adjacent areas apart from Europe to Asia and vice versa. In our 'starting' model, the Europe-Asia dispersal multiplier is set to 0.5 during time slices 1–3 (i.e. Bajocian–Tithonian) in order to reflect the uncertain impact of the Turgai Sea/Russian Basin regions.

Table S2. Time slice 1 dispersal multiplier values.

Dispersal multipliers	Europe (E)	Africa (F)	Indo-Mad (M)	N America (N)	S America (S)	Australia (U)
Asia (A)	0.5	0.000001	0.000001	0.000001	0.000001	0.000001
Europe (E)		0.000001	0.000001	1	0.000001	0.000001
Africa (F)			1	1	1	1
Indo-Mad (M)				0.000001	1	1
N America (N)					1	0.000001
S America (S)						1

Time slice 2: 91.3–86.1 million years prior to the K/Pg boundary (= Kimmeridgian [157.3–152.1 Ma])

Sea floor spreading commenced between eastern North America and northwest Africa at approximately 190 Ma and accelerated at around 170 Ma[214,215], suggesting that these two regions were separated by an oceanic barrier (i.e. the Central Atlantic) during the Middle Jurassic (although see Lawver *et al.*[187] for an alternative scenario). The land connection between North and South America persisted until the opening of the Gulf of Mexico between approximately 170 and 158 Ma (Bajocian–early Oxfordian) according to Seton *et al.*[212]. Here, we assume that an oceanic barrier separated North and South America from the start of the Oxfordian based on these rifting event dates, and also the palaeocoastline reconstructions of Smith *et al.*[199] and Blakey[203] (N.B. the Bajocian map in Smith *et al.*[199] suggests that a marine barrier separated North and South America at this time, while a Laurasia–Gondwana land connection was maintained via North America and Africa – but this reconstruction is not supported by some recent assessments of the timing and sequence of sea floor spreading

events [e.g. Seton *et al.*[212], and references therein]). Both Wilf *et al.*[204] and Lawver *et al.*[187] proposed the existence of land connections between Gondwana and Laurasia until well into the Late Jurassic or even the earliest Cretaceous, but the former study argued that a climatic barrier imposed an effective separation between northern and southern terrestrial biotas earlier in the Late Jurassic. Thus, we accept that during the Late Jurassic, direct dispersal of terrestrial animals between Laurasia and Gondwana would have been greatly reduced or impossible from the start of the Kimmeridgian onwards, but it must be acknowledged that there is considerable uncertainty about the exact timing of this biotic separation.

The timing of the opening of the North Atlantic is debated, and its effectiveness as a marine barrier to Late Jurassic North America–Europe dispersal is even more problematic. According to Seton *et al.*[212] (and references therein), rifting and subsequent sea floor spreading in the North Atlantic region occurred in several phases during the Jurassic, Cretaceous and Cenozoic. Initially, rift basins formed in the region between Iberia and Newfoundland during the Late Triassic and Early Jurassic, but this was followed by a tectonically quiet period until the Late Jurassic[212]. This is consistent with the reconstructions of Golonka *et al.*[205], who proposed the presence of broad areas of land connecting North America and Europe during the Bajocian and Bathonian, followed by a narrow seaway during the Callovian–Kimmeridgian. Given that the oldest date for the onset of sea floor spreading between Iberia and Newfoundland is ~147 Ma (late Tithonian), any area of sea occupying this region in the Callovian–Kimmeridgian is likely to have been a relatively shallow, narrow, and potentially ephemeral epicontinental sea or series of flooded rift basins. There are several much younger estimates for the onset of sea floor spreading in the North Atlantic, ranging from 128 to 112 Ma (see references in Seton *et al.*[212]). Some palaeogeographic reconstructions do not show a broad area of ocean between North America and Europe until the Barremian (129.4 Ma)[202] or Aptian[199], and the latter study reconstructed a North America-Europe land connection during the Valanginian (perhaps reflecting a sea-level lowstand at this time). Development of the North Atlantic further north (i.e. between Greenland and northwest Europe) took place in the Late Jurassic and Early Cretaceous, with a second phase of activity at around 70 Ma, and sea floor spreading at 56–55 Ma according to Seton *et al.*[212] (and references therein). Thus, the presence of land connections between North America and Europe until at least the Kimmeridgian is palaeogeographically plausible, even if such connections were occasionally disrupted at times of higher sea level. This view is supported by some strong biogeographic evidence, such as very close, or even conspecific, relationships between several European and North American Late Jurassic dinosaurs (e.g. *Stegosaurus*[216], *Allosaurus*[217-219], *Ceratosaurus*[220], *Stokesosaurus* + *Juratyrant*[221,222], *Torvosaurus*[223], *Supersaurus* + *Dinheirosaurus*[63,71]). Therefore, we provisionally allow dispersal to occur between North America and Europe in time slices 1 and 2 (Bajocian–Kimmeridgian), and then partially restrict it during the Tithonian–Hauterivian by applying a dispersal multiplier of 0.5. From the Barremian onwards, this dispersal multiplier is set to 0.000001 because the North Atlantic was probably an effective barrier to terrestrial dispersal from this point onwards (though see time slice 21).

Rifting between southern Africa and portions of Antarctica and Madagascar started in the early Middle Jurassic, with the transition from continental rifting to sea floor spreading variously dated as occurring between 183 and 165 Ma according to Seton *et al.*[212] (and references therein). At approximately 153 Ma (close to the Kimmeridgian–Tithonian boundary), south-eastern Africa started to separate from eastern Antarctica[224-227], although Seton *et al.*[212] chose to model the initiation of sea floor spreading along the entire eastern margin of Africa at ~160 Ma. These events created the Mozambique Basin on the African side, and the Riiser-Larsen Sea on the Antarctic side[226]. The oldest oceanic crust (153 Ma) in

the Mozambique Basin is in the northwest[226], corresponding to the oldest magnetic anomalies identified in the Riiser-Larsen Sea by Jokat *et al.*[225]. The rate of spreading accelerated at ~133 Ma, apparently corresponding to the onset of the opening of the South Atlantic[226]. The palaeocoastline reconstructions of Smith *et al.*[199] suggest that the last land connection between southern Africa and Antarctica was present during the Tithonian, which is somewhat later than predicted on the basis of the onset of sea floor spreading. In contrast, the palaeogeographic reconstructions of Blakey[203] indicate the presence of at least a shallow sea between Africa and Antarctica during the Late Jurassic. In the Tithonian, Madagascar was probably already fully isolated from Africa by an area of ocean[203], and these two continents are estimated to have been separated by approximately 400 km of ocean by ~130–120 Ma (late Hauterivian–early Aptian) according to Ali and Krause[228]. The Mozambique Ridge is interpreted to have formed between 140–120 Ma but probably did not act as an island for terrestrial dispersal between Africa and Antarctica[228]. Therefore, during this time slice, we set the Africa-Indo-Madagascar and Africa-Australia dispersal multiplier values at 1.0, but this is changed to 0.5 in the Tithonian to reflect the uncertainty concerning the exact separation of Africa from East Gondwana. From the Berriasian onwards, however, these dispersal multipliers are set to 0.

The palaeogeography of the Patagonia-West Antarctica Peninsula region during the Late Jurassic and Cretaceous is problematic. As a result, there are at least two main competing models for the earliest stages of Gondwana fragmentation[229]. These alternatives have been termed the ‘Samafrica’ and ‘Africa-first’ models[229]. Both models involve rifting between southern Africa and Antarctica, and between eastern Africa and Madagascar, commencing in the Late Jurassic (see above). It is also generally accepted that rifting further west started to open the Weddell Sea between southwest Africa, southern South America, and the West Antarctic Peninsula (see Seton *et al.*[212] and references therein). This rifting commenced at approximately 167–160 Ma, with sea floor spreading initiating at around 146 Ma according to Seton *et al.*[212] and references therein. The difficulty for those wishing to model the biogeographic consequences of these events stems from the lack of clarity regarding whether or not the land connection between South America and Antarctica was severed during the Late Jurassic, or much later. Under the Samafrica model, the Jurassic rifting results in the separation of the southern tip of South America from the West Antarctic Peninsula, so that Gondwana was initially divided into separate western (Africa+South America) and eastern (Antarctica+Indo-Madagascar+Australia) portions as early as 150 Ma[199,203,230-236]. In contrast, the Africa-first model proposes that the South America-West Antarctic Peninsula connection was maintained throughout the Jurassic and Cretaceous, and was not lost until the opening of the Drake Passage during the Cenozoic at around 52 Ma[204,228,237]. Ali and Krause[228] (p. 1860) stated that, “At the Early/Late Cretaceous transition (start of the Cenomanian, 99.6 Ma; Fig. 4b), South America, Antarctica and Australia remained joined…,” but the cited palaeogeographic map depicts areas of shallow and deep sea between the southern tip of Patagonia and the West Antarctic Peninsula. J. Ali (pers. comm. to PU in 2014) has confirmed that this discrepancy is the result of an error in the palaeogeographic reconstructions. He also goes on to note that there appears to have been very little movement between these two regions until the Cenozoic, ~35–40 Ma, as can be seen in the reconstructions presented by Schettino and Scotese[238]. Vérard *et al.*[239] more-or-less supported this interpretation; however, they did suggest that South America and Antarctica were separated for an unspecified length of time between 103 and 84 Ma. The only map in which Vérard *et al.*[239] depict the separation is their 95 Ma map (Vérard *et al.*[239]: fig. 5h), and it is only in their description of this map (Vérard *et al.*[239]: p. 50) that it is explicitly stated that the two continents were separated; both their 103 and 84 Ma maps depict South America and Antarctica united (Vérard *et al.*[239]: figs.

5g and 5i). It should be noted that, even if there was a continental crust connection between South America and Antarctica during all or part of the Cretaceous, this does not necessarily mean that this provided a viable landbridge for dispersal of terrestrial taxa: changes in sea level might have periodically flooded this region, creating epicontinental seas (J. Ali pers. comm. 2014), and its relatively high latitude might also have transformed this dispersal corridor into a 'filter barrier' at times[229]. Because of this uncertainty, we have set the dispersal multiplier for dispersal between South America and Indo-Madagascar/Australia (both via Antarctica) at 0.5 in our starting model, so that the relaxed and harsh models can be used to examine, respectively, the effects of allowing and prohibiting dispersal across this landbridge in time slice 3 onwards (but note that the nature of the South America–Antarctica connection becomes less important from time slice 9 onwards because of the loss of contact between Antarctica and Indo-Madagascar).

Table S3. Time slice 2 dispersal multiplier values.

Dispersal multipliers	Europe (E)	Africa (F)	Indo-Mad (M)	N America (N)	S America (S)	Australia (U)
Asia (A)	0.5	0.000001	0.000001	0.000001	0.000001	0.000001
Europe (E)		0.000001	0.000001	1	0.000001	0.000001
Africa (F)			1	0.000001	1	1
Indo-Mad (M)				0.000001	1	1
N America (N)					0.000001	0.000001
S America (S)						1

Time slice 3: 86.1–79 million years prior to the K/Pg boundary (= Tithonian [152.1–145 Ma])

As noted in time slice 2, the dispersal multipliers for North America–Europe, Africa–Australia/Indo-Madagascar and South America–Australia/Indo-Madagascar dispersals are set to 0.5 in our starting model for this time slice.

Table S4. Time slice 3 dispersal multiplier values.

Dispersal multipliers	Europe (E)	Africa (F)	Indo-Mad (M)	N America (N)	S America (S)	Australia (U)
Asia (A)	0.5	0.000001	0.000001	0.000001	0.000001	0.000001
Europe (E)		0.000001	0.000001	0.5	0.000001	0.000001
Africa (F)			0.5	0.000001	1	0.5
Indo-Mad (M)				0.000001	0.5	1
N America (N)					0.000001	0.000001
S America (S)						0.5

Time slice 4: 79–76.4 million years prior to the K/Pg boundary (= early Berriasian [145–142.4 Ma])

Baraboshkin *et al.*[240] presented evidence that the marine connection between the Boreal and Tethys Oceans, in the region of the Russian Basin, was interrupted during the early Berriasian. Therefore, the Europe–Asia dispersal multiplier is set at 1.0 for this interval.

There is some palaeogeographic support for a land connection between Africa and Europe during the Early Cretaceous (approximately Berriasian–Barremian). This is the 'Apulian Route', supposedly created by a series of microplates lying between northern Africa and southern Europe at a time of sea-level lowstand (see Ezcurra and Agnolin[189] and references therein). This is consistent with apparent dispersal of terrestrial taxa, such as dinosaurs and mammals, between Africa and Europe during the Early Cretaceous[99,189,207,241]. This land connection seems to have been severed by the Aptian at the latest (but possibly earlier, during the Barremian, as a result of a rise in sea level), though it might have reappeared during the Campanian (Ezcurra and Agnolin[189] and references therein). Therefore, during time slices 4–6 (Berriasian–Hauterivian) we allow Europe–Africa dispersal to occur with a dispersal multiplier value of 1.0. During time slice 7 (Barremian) this value is reduced to 0.5, and from the Aptian onwards this is set to 0.000001 (but see time slices covering the Campanian).

The Africa–India/Australia dispersal multiplier values are set to 0.000001 in this time slice and all subsequent ones (see time slice 2 for details).

Table S5. Time slice 4 dispersal multiplier values.

Dispersal multipliers	Europe (E)	Africa (F)	Indo-Mad (M)	N America (N)	S America (S)	Australia (U)
Asia (A)	1	0.000001	0.000001	0.000001	0.000001	0.000001
Europe (E)		1	0.000001	0.5	0.000001	0.000001
Africa (F)			0.000001	0.000001	1	0.000001
Indo-Mad (M)				0.000001	0.5	1
N America (N)					0.000001	0.000001
S America (S)						0.5

Time slice 5: 76.4–65.15 million years prior to the K/Pg boundary (= late Berriasian–early Hauterivian [142.4–131.15 Ma])

The marine connection between the Boreal and Tethys Oceans in the Russian Basin/Turgai regions was re-established during the late Berriasian and persisted throughout the Valanginian, although it became more restricted[240]. The existence of this marine barrier has been inferred partly on geological data and partly on biotic interchanges between the Boreal and Tethyan realms (Baraboshkin *et al.*[240] and references therein). We therefore prohibit Europe–Asia dispersal during this time slice.

Table S6. Time slice 5 dispersal multiplier values.

Dispersal multipliers	Europe (E)	Africa (F)	Indo-Mad (M)	N America (N)	S America (S)	Australia (U)
Asia (A)	0.000001	0.000001	0.000001	0.000001	0.000001	0.000001
Europe (E)		1	0.000001	0.5	0.000001	0.000001
Africa (F)			0.000001	0.000001	1	0.000001
Indo-Mad (M)				0.000001	0.5	1
N America (N)					0.000001	0.000001
S America (S)						0.5

Time slice 6: 65.15–63.4 million years prior to the K/Pg boundary (= late Hauterivian [131.15–129.4 Ma])

During this time slice, the Russian Basin/Turgai marine barrier was again interrupted by a land connection between Europe and Asia, which apparently persisted until approximately the end of the Aptian[240]. We therefore set the dispersal multiplier value to 1 for Europe–Asia dispersals at this time and also in time slices 7–9.

Table S7. Time slice 6 dispersal multiplier values.

Dispersal multipliers	Europe (E)	Africa (F)	Indo-Mad (M)	N America (N)	S America (S)	Australia (U)
Asia (A)	1	0.000001	0.000001	0.000001	0.000001	0.000001
Europe (E)		1	0.000001	0.5	0.000001	0.000001
Africa (F)			0.000001	0.000001	1	0.000001
Indo-Mad (M)				0.000001	0.5	1
N America (N)					0.000001	0.000001
S America (S)						0.5

Time slice 7: 63.4–59 million years prior to the K/Pg boundary (= Barremian [129.4–125 Ma])

As noted in time slice 4, the Europe–Africa dispersal multiplier is set at 0.5 during the Barremian, in order to reflect the waning importance of the Apulian Route (Ezcurra and Agnolin[189] and references therein).

In this time slice, we set the North America–Europe dispersal multiplier to 0.000001, reflecting the presence of a well-developed North Atlantic marine barrier between these two continents (see time slice 2).

Table S8. Time slice 7 dispersal multiplier values.

Dispersal multipliers	Europe (E)	Africa (F)	Indo-Mad (M)	N America (N)	S America (S)	Australia (U)
Asia (A)	1	0.000001	0.000001	0.000001	0.000001	0.000001
Europe (E)		0.5	0.000001	0.000001	0.000001	0.000001
Africa (F)			0.000001	0.000001	1	0.000001
Indo-Mad (M)				0.000001	0.5	1
N America (N)					0.000001	0.000001
S America (S)						0.5

Time slice 8: 59–53 million years prior to the K/Pg boundary (= early Aptian [125–119 Ma])

The Europe–Africa dispersal multiplier value is set to 0.000001 to reflect the absence of the Apulian Route (see time slices 4 and 7).

Table S9. Time slice 8 dispersal multiplier values.

Dispersal multipliers	Europe (E)	Africa (F)	Indo-Mad (M)	N America (N)	S America (S)	Australia (U)
Asia (A)	1	0.000001	0.000001	0.000001	0.000001	0.000001
Europe (E)		0.000001	0.000001	0.000001	0.000001	0.000001
Africa (F)			0.000001	0.000001	1	0.000001
Indo-Mad (M)				0.000001	0.5	1
N America (N)					0.000001	0.000001
S America (S)						0.5

Time slice 9: 53–47 million years prior to the K/Pg boundary (= late Aptian [119–113 Ma])

The dispersal multiplier for North America–Asia dispersal is set to 0.5 for this time slice (see time slice 10 for details).

The timing of the separation and complete biogeographic isolation of Indo-Madagascar is controversial (see the review in Ali and Krause[228]). However, Ali and Krause[228]present strong palaeogeographic evidence to support the view that Indo-Madagascar became separated from Antarctica by a marine barrier at approximately 119 Ma. This view was also supported by Lawver *et al.*[187], although they set the latest possible date for an Antarctica–Indo-Madagascar land connection at 108 Ma. Thus, from the late Aptian time slice onwards we prohibit terrestrial dispersal between Indo-Madagascar and South America/Australia.

Table S10. Time slice 9 dispersal multiplier values.

Dispersal multipliers	Europe (E)	Africa (F)	Indo-Mad (M)	N America (N)	S America (S)	Australia (U)
Asia (A)	1	0.000001	0.000001	0.5	0.000001	0.000001
Europe (E)		0.000001	0.000001	0.000001	0.000001	0.000001
Africa (F)			0.000001	0.000001	1	0.000001
Indo-Mad (M)				0.000001	0.000001	0.000001
N America (N)					0.000001	0.000001
S America (S)						0.5

Time slice 10: 47–40.75 million years prior to the K/Pg boundary (= early Albian [113–106.75 Ma])

According to Baraboshkin *et al.*[240], the Russian Basin/Turgai Sea regions again formed a marine barrier to Europe–Asia terrestrial dispersal during the early Albian. The Europe–Asia dispersal multiplier for this time slice is therefore set to 0.000001.

There is some palaeogeographic support for the presence of the Bering landbridge between North America and Asia during the Aptian–Albian[242]. Zanno and Makovicky[243] (and references therein) argued for this, partly on the basis of biogeographic data (e.g. the presence of tyrannosauroid material in North America at ~108 Ma). This Bering landbridge probably allowed North America–Asia dispersal during the early Albian, perhaps during a 10 million year interval. Therefore, the North America–Asia dispersal multiplier value is set to 1.0 for this time slice, and 0.5 for the late Aptian and late Albian in order to reflect the uncertainties surrounding the exact dates for the presence of this late Early Cretaceous Bering landbridge.

In time slice 12, we suggest that the final land connection between South America and Africa was severed at approximately 103 Ma, as a result of the joining of the Central and South Atlantic Oceans. However, recent work by Lawver *et al.*[187] has presented evidence that some form of marine barrier between Brazil and west Africa was present as early as 112 Ma. To reflect this uncertainty, we have set the South America–Africa dispersal multiplier to 0.5 during time slices 10 and 11.

Table S11. Time slice 10 dispersal multiplier values.

Dispersal multipliers	Europe (E)	Africa (F)	Indo-Mad (M)	N America (N)	S America (S)	Australia (U)
Asia (A)	0.000001	0.000001	0.000001	1	0.000001	0.000001
Europe (E)		0.000001	0.000001	0.000001	0.000001	0.000001
Africa (F)			0.000001	0.000001	0.5	0.000001
Indo-Mad (M)				0.000001	0.000001	0.000001
N America (N)					0.000001	0.000001
S America (S)						0.5

Time slice 11: 40.75–37 million years prior to the K/Pg boundary (= the early portion of the late Albian [106.75–103 Ma])

According to Baraboshkin *et al.*[240], the Turgai Sea was again interrupted by a Europe–Asia land connection during the late Albian and Early Cenomanian. Therefore, the Europe–Asia dispersal multiplier value is set to 1.0 for this time slice and the next two.

The North America–Asia dispersal multiplier is set to 0.5 during this time slice (see time slice 10 for details).

Table S12. Time slice 11 dispersal multiplier values.

Dispersal multipliers	Europe (E)	Africa (F)	Indo-Mad (M)	N America (N)	S America (S)	Australia (U)
Asia (A)	1	0.000001	0.000001	0.5	0.000001	0.000001
Europe (E)		0.000001	0.000001	0.000001	0.000001	0.000001
Africa (F)			0.000001	0.000001	0.5	0.000001
Indo-Mad (M)				0.000001	0.000001	0.000001
N America (N)					0.000001	0.000001
S America (S)						0.5

Time slice 12: 37–34.5 million years prior to the K/Pg boundary (= latest Albian [103–100.5 Ma])

The Europe–Asia dispersal multiplier for this time slice is set to 1.0 (see time slice 11).

A key event in the palaeogeographic and biogeographic history of Gondwana during the Cretaceous concerns the final separation of South America from Africa. Some workers have proposed that a land connection persisted until as late as 80 Ma (e.g. Ezcurra and Agnolin[189] [and references therein], Sereno *et al.*[244]). Such a late connection depends on the presence of a landbridge created by the Walvis Ridge and Rio Grande Rise, but this seems improbable

according to Gheerbrant and Rage[241] and Lawver *et al.*[187]. The biogeographic support for such a late land connection has also been criticised by Upchurch[229]. In fact, palaeogeographic data support a somewhat earlier date for the final separation of South America and Africa. The initial separation of southern South America and Africa commenced at approximately 133 Ma[226], with the South Atlantic perhaps fully open by 113 Ma[245]. The final severing of the Africa–South America land connection is dated at approximately 103 Ma[246], and this is consistent with the range of dates for the connection of the South and Central Atlantic Oceans and their spreading ridge systems (103–96 Ma) presented by Seton *et al.*[212]. The 103 Ma date for the separation of South America and Africa is also supported by the palaeocoastline reconstructions of Smith *et al.*[199], which show a tenuous land connection during the Albian (map dated as 105 Ma) and a clear marine barrier in the Cenomanian (map dated at 95 Ma) (see also the maps in Blakey[203] and Ali and Krause[228]). Lawver *et al.*[187] proposed a slightly earlier date (112–106 Ma) for the final separation of South America and Africa, and this is reflected here in a dispersal multiplier value of 0.5 in time slices 10 and 11 (see above). Thus, in our starting model, we allow South America–Africa dispersal to occur prior to 103 Ma and prohibit it after this date.

Table S13. Time slice 12 dispersal multiplier values.

Dispersal multipliers	Europe (E)	Africa (F)	Indo-Mad (M)	N America (N)	S America (S)	Australia (U)
Asia (A)	1	0.000001	0.000001	0.5	0.000001	0.000001
Europe (E)		0.000001	0.000001	0.000001	0.000001	0.000001
Africa (F)			0.000001	0.000001	0.000001	0.000001
Indo-Mad (M)				0.000001	0.000001	0.000001
N America (N)					0.000001	0.000001
S America (S)						0.5

Time slice 13: 34.5–31.2 million years prior to the K/Pg boundary (= early Cenomanian [100.5–97.2 Ma])

The Europe–Asia dispersal multiplier for this time slice is set to 1.0 (see time slice 11 for details).

Table S14. Time slice 13 dispersal multiplier values.

Dispersal multipliers	Europe (E)	Africa (F)	Indo-Mad (M)	N America (N)	S America (S)	Australia (U)
Asia (A)	1	0.000001	0.000001	0.000001	0.000001	0.000001
Europe (E)		0.000001	0.000001	0.000001	0.000001	0.000001
Africa (F)			0.000001	0.000001	0.000001	0.000001
Indo-Mad (M)				0.000001	0.000001	0.000001
N America (N)					0.000001	0.000001
S America (S)						0.5

Time slice 14: 31.2–27.9 million years prior to the K/Pg boundary (= late Cenomanian [97.2–93.9 Ma])

The Turgai Sea seems to have been present from the late Cenomanian onwards throughout the rest of the Late Cretaceous[240]. However, the geological and palaeontological controls on the nature of this barrier are less clear than during the Early Cretaceous. For example, some recent studies have suggested that there were ephemeral land connections across the Turgai Sea region during the Late Cretaceous[247,248]. In particular, Csiki-Sava *et al.*[190] argued that drops in sea level during the early Turonian and late Coniacian resulted in a land connection between Europe and Asia. Therefore, to reflect this uncertainty, we set the Europe-Asia dispersal multiplier at 0.5 in our starting model for this time slice and all subsequent ones (except for the early Turonian, late Coniacian and Campanian; see below).

Table S15. Time slice 14 dispersal multiplier values.

Dispersal multipliers	Europe (E)	Africa (F)	Indo-Mad (M)	N America (N)	S America (S)	Australia (U)
Asia (A)	0.5	0.000001	0.000001	0.000001	0.000001	0.000001
Europe (E)		0.000001	0.000001	0.000001	0.000001	0.000001
Africa (F)			0.000001	0.000001	0.000001	0.000001
Indo-Mad (M)				0.000001	0.000001	0.000001
N America (N)					0.000001	0.000001
S America (S)						0.5

Time slice 15: 27.9–25.85 million years prior to the K/Pg boundary (= early Turonian [93.9–91.85 Ma])

The Europe–Asia dispersal multiplier is set to 1.0 for this time slice (see time slice 14 for details).

Table S16. Time slice 15 dispersal multiplier values.

Dispersal multipliers	Europe (E)	Africa (F)	Indo-Mad (M)	N America (N)	S America (S)	Australia (U)
Asia (A)	1	0.000001	0.000001	0.000001	0.000001	0.000001
Europe (E)		0.000001	0.000001	0.000001	0.000001	0.000001
Africa (F)			0.000001	0.000001	0.000001	0.000001
Indo-Mad (M)				0.000001	0.000001	0.000001
N America (N)					0.000001	0.000001
S America (S)						0.5

Time slice 16: 25.85–22.05 million years prior to the K/Pg boundary (= late Turonian–early Coniacian [91.85–88.05 Ma])

The Europe–Asia dispersal multiplier is set to 0.5 (see time slice 14 for details).

Table S17. Time slice 16 dispersal multiplier values.

Dispersal multipliers	Europe (E)	Africa (F)	Indo-Mad (M)	N America (N)	S America (S)	Australia (U)
Asia (A)	0.5	0.000001	0.000001	0.000001	0.000001	0.000001
Europe (E)		0.000001	0.000001	0.000001	0.000001	0.000001
Africa (F)			0.000001	0.000001	0.000001	0.000001
Indo-Mad (M)				0.000001	0.000001	0.000001
N America (N)					0.000001	0.000001
S America (S)						0.5

Time slice 17: 22.05–20.3 million years prior to the K/Pg boundary (= late Coniacian [88.05–86.3 Ma])

The Europe–Asia dispersal multiplier is set to 1.0 for this time slice (see time slice 14 for details).

Table S18. Time slice 17 dispersal multiplier values.

Dispersal multipliers	Europe (E)	Africa (F)	Indo-Mad (M)	N America (N)	S America (S)	Australia (U)
Asia (A)	1	0.000001	0.000001	0.000001	0.000001	0.000001
Europe (E)		0.000001	0.000001	0.000001	0.000001	0.000001
Africa (F)			0.000001	0.000001	0.000001	0.000001
Indo-Mad (M)				0.000001	0.000001	0.000001
N America (N)					0.000001	0.000001
S America (S)						0.5

Time slice 18: 20.3–17.6 million years prior to the K/Pg boundary (= Santonian [86.3–83.6 Ma])

The Europe–Asia dispersal multiplier is set to 0.5 (see time slice 14 for details).

Table S19. Time slice 18 dispersal multiplier values.

Dispersal multipliers	Europe (E)	Africa (F)	Indo-Mad (M)	N America (N)	S America (S)	Australia (U)
Asia (A)	0.5	0.000001	0.000001	0.000001	0.000001	0.000001
Europe (E)		0.000001	0.000001	0.000001	0.000001	0.000001
Africa (F)			0.000001	0.000001	0.000001	0.000001
Indo-Mad (M)				0.000001	0.000001	0.000001
N America (N)					0.000001	0.000001
S America (S)						0.5

Time slice 19: 17.6–11.85 million years prior to the K/Pg boundary (= early Campanian [83.6–77.85 Ma])

The Turgai Sea apparently went through a relatively deep phase during the Campanian[240]. In this time slice, and in the following one, the Europe–Asia dispersal multiplier is set to 0.000001, and is then set to 0.5 for the two Maastrichtian time slices.

Ezcurra and Agnolin[189] (and references therein) suggested that biotic interchange occurred between Africa and Europe during the Campanian and Maastrichtian via a re-emergent Apulian Route (see time slice 4). Although Ezcurra and Agnolin[189] cite some geological/palaeogeographic studies to support this claim, much of the evidence for the proposed land connection is based on biogeographic data. Palaeocoastline reconstructions for the Campanian and Maastrichtian typically do not depict a land connection, and in fact there appears to have been quite a wide area of ocean between Africa and Europe at this time[199,203]. Because of the uncertainties surrounding the presence/absence of this landbridge, we set the dispersal multiplier for Europe–Africa dispersal to 0.5 during the Campanian and Maastrichtian time slices.

Zanno and Makovicky[243] (and references therein) suggested that a second phase of North America–Asia biotic interchange occurred across the Bering landbridge during the Campanian and Maastrichtian. However, Brikiatis[249] has argued that Beringia was not fully exposed during the Maastrichtian, based on floristic data. We therefore set the dispersal multiplier for North America–Asia dispersal to 1.0 for the two Campanian time slices and 0.5 for the two Maastrichtian time slices.

Table S20. Time slice 19 dispersal multiplier values.

Dispersal multipliers	Europe (E)	Africa (F)	Indo-Mad (M)	N America (N)	S America (S)	Australia (U)
Asia (A)	0.000001	0.000001	0.000001	1	0.000001	0.000001
Europe (E)		0.5	0.000001	0.000001	0.000001	0.000001
Africa (F)			0.000001	0.000001	0.000001	0.000001
Indo-Mad (M)				0.000001	0.000001	0.000001
N America (N)					0.000001	0.000001
S America (S)						0.5

Time slice 20: 11.85–6.1 million years prior to the K/Pg boundary (= late Campanian [77.85–72.1 Ma])

Numerous workers have suggested that a land connection between North and South America formed during the latest Cretaceous, allowing biotic exchanges of terrestrial taxa[187,204,250-256]. The existence of such a landbridge has received some support from geological evidence[257]. Two competing geodynamic models have been proposed to explain the tectonic history of this region: (1) the generation of the proto-Antillean volcanic island arc[258-260]; and (2) the continentalisation of the Panamanian isthmus as a result of plate tectonic motions in the latest Cretaceous[257] or, more recently, the proposal that this isthmus had been emergent for a long period prior to the latest Cretaceous (e.g. James[261,262] and references therein). If these events did create a land connection between North and South America, then it was probably most effective as a dispersal route for terrestrial organisms during the late Campanian and early Maastrichtian[257], and a drop in sea level at this time might also have played an important role

(Loewen *et al.*[263] and references therein). Moreover, the presence of some form of land connection between North and South America during the Late Cretaceous is supported by the biogeography of marine invertebrates in the Caribbean/Gulf of Mexico and eastern Pacific regions (i.e. on either side of the proposed landbridge). According to Briggs[264,265] and James[261], marine invertebrate faunas diverged in these two regions, with the onset of differentiation dating perhaps as far back as the Barremian, and complete separation by around 88 Ma. However, the palaeogeographic and biogeographic evidence for this landbridge is not universally accepted. Some workers have proposed that this land route was interrupted during the latest Campanian or Maastrichtian[266], and Pitman *et al.*[234] suggested that this land connection was frequently breached as a result of sea level changes so that it fluctuated between being a landbridge, a filter barrier, and a true barrier (probably on time scales that are too fine to resolve in large-scale palaeogeographic reconstructions). Ezcurra and Agnolin[189] also rejected the evidence for biotic interchange between North and South America during the latest Cretaceous, partly on the basis of the absence of a satisfactory palaeogeographic mechanism, and partly because their statistically significant biogeographic patterns did not support such an event. Here, therefore, we set the North America–South America dispersal multiplier value to 0.5 during the late Campanian and early Maastrichtian.

Rifting between Antarctica and Australia commenced as early as 165 Ma, with the onset of breakup between 125 and 83 Ma (Seton *et al.*[212] and references therein). However, these two areas apparently remained connected throughout the Jurassic and Cretaceous[267,268]. Smith *et al.*[199], Scotese[202] and Blakey[203] depict a widening ocean between these two areas from the Campanian onwards (consistent with sea floor spreading between around 84 and 61 Maya according to Seton *et al.*[212]). By the end of the Maastrichtian, the land bridge between Antarctica and Australia appears to have been limited to a relatively narrow strip corresponding to modern day Tasmania[269]. Although we might expect terrestrial dispersal between Antarctica and Australia to have been at least partially hindered by this growing oceanic barrier in the latest Cretaceous, we provisionally allow dispersal to occur in our models (N.B. this assumption will not affect our analyses because the Australian sauropods in our data set all predate the Campanian).

Table S21. Time slice 20 dispersal multiplier values.

Dispersal multipliers	Europe (E)	Africa (F)	Indo-Mad (M)	N America (N)	S America (S)	Australia (U)
Asia (A)	0.000001	0.000001	0.000001	1	0.000001	0.000001
Europe (E)		0.5	0.000001	0.000001	0.000001	0.000001
Africa (F)			0.000001	0.000001	0.000001	0.000001
Indo-Mad (M)				0.000001	0.000001	0.000001
N America (N)					0.5	0.000001
S America (S)						0.5

Time-slice 21: 6.1–3.05 million years prior to the K/Pg boundary (= early Maastrichtian [72.1–69.05 Ma])

Brikiatis[249] presented evidence that the Western Interior Seaway regressed during the early Maastrichtian, and so re-connected Laramidia (western North America) and Appalachia (eastern North America). At approximately the same time, the De Geer landbridge provided a high latitude land connection between Greenland and Fenoscandia[249]. Thus, some dispersal of terrestrial taxa between North America and Europe was potentially possible during the

Maastrichtian. However, it seems unlikely that sauropods used this high latitude route, and in any case it is unlikely to affect our analyses because our data set has only one North American and no European sauropods of Maastrichtian age. Nevertheless, we allow North America–Europe dispersal with a dispersal multiplier value of 0.5 during this early Maastrichtian time slice.

The Europe–Asia, North America–Asia and Europe–Africa dispersal multipliers are set to 0.5 during this time slice and the following one (see time slices 14 and 19).

Table S22. Time slice 21 dispersal multiplier values.

Dispersal multipliers	Europe (E)	Africa (F)	Indo-Mad (M)	N America (N)	S America (S)	Australia (U)
Asia (A)	0.5	0.000001	0.000001	0.5	0.000001	0.000001
Europe (E)		0.5	0.000001	0.5	0.000001	0.000001
Africa (F)			0.000001	0.000001	0.000001	0.000001
Indo-Mad (M)				0.000001	0.000001	0.000001
N America (N)					0.5	0.000001
S America (S)						0.5

Time slice 22: 3.05–0 million years prior to the K/Pg boundary (= late Maastrichtian [69.05–66 Ma])

The North America–Europe dispersal multiplier is set to 0 for this time slice (see time slice 17).

Table S23. Time slice 22 dispersal multiplier values.

Dispersal multipliers	Europe (E)	Africa (F)	Indo-Mad (M)	N America (N)	S America (S)	Australia (U)
Asia (A)	0.5	0.000001	0.000001	0.5	0.000001	0.000001
Europe (E)		0.5	0.000001	0.000001	0.000001	0.000001
Africa (F)			0.000001	0.000001	0.000001	0.000001
Indo-Mad (M)				0.000001	0.000001	0.000001
N America (N)					0.000001	0.000001
S America (S)						0.5

Table S24. Summary of the palaeogeographic changes accounted for in the analyses conducted herein.

Time slice	Time slice stage range	Event (s)	Comments	References
1	Bajocian–Oxfordian	All areas in direct or indirect contact, except Asia (possibly isolated by Russian Basin/Turgai Sea)	Nature of Turgai Sea barrier is uncertain	Smith *et al.*[199]; Scotese[202].
2	Kimmeridgian	Separation of N. America from Africa and S. America; N. Atlantic starts to open; rifting between Africa and Indo-Madagascar+Antarctica		Seton *et al.*[212]; Blakey[203]; Smith *et al.*[199].
3	Tithonian	Possible separation of S. America from Antarctica; sea floor spreading between Africa and Indo-Madagascar+Antarctica	Effect of N. Atlantic barrier uncertain; unclear whether S. America became disconnected from Antarctica	Seton *et al.*[212]; Blakey[203]; Smith *et al.*[199]; Ali and Krause[228]
4	Early Berriasian	Landbridge across Russian Basin/Turgai region; landbridge between Africa and Europe; loss of contact between Africa and Indo-Antarctica+Madagascar+Australia		Ezcurra and Agnolin[189]; Baraboshkin *et al.*[240]
5	Late Berriasian–early Hauterivian	Russian Basin/Turgai Sea barrier re-established		Baraboshkin *et al.*[240]
6	Late Hauterivian	Landbridge across Russian Basin/Turgai region		Baraboshkin *et al.*[240]
7	Barremian	Waning importance of the Apulian Route between Africa and Europe; N. Atlantic barrier fully established		Ezcurra and Agnolin[189]; Smith *et al.*[199]; Scotese[202].
8	Early Aptian	Apulian Route absence		Ezcurra and Agnolin[189]
9	Late Aptian	Possible commencement of Bering landbridge between Asia and N. America; separation of Indo-Madagascar form Antarctica		Ali and Krause[228]; Zanno and Makovicky[243]
10	Early Albian	Turgai Sea separates Europe and Asia; Bering landbridge between N. America and Asia		Baraboshkin *et al.*[240]; Zanno and Makovicky[243]
11	Early portion of late Albian	Landbridge across the Turgai Sea; waning influence of the Bering landbridge between N. America and Asia		Baraboshkin *et al.*[240]; Zanno and Makovicky[243]
12	Latest Albian	Final separation of S. America and Africa		Seton *et al.*[212]; Heine *et al.*[246]
13	Early Cenomanian	Disappearance of the Bering landbridge between N. America and Asia		Zanno and Makovicky[243]
14	Late Cenomanian	Turgai Sea possibly prevents dispersal between Europe and Asia	Effect of Turgai Sea is uncertain	Baraboshkin *et al.*[240]; Akhmetiev and Beniamovski[247]; Akhmetiev *et al.*[248]
15	Early Turonian	Landbridge across Turgai Sea region	Results from drop in sea level	Csiki-Sava *et al.*[190]; Baraboshkin *et al.*[240] (and references therein)

Time slice	Time slice stage range	Event (s)	Comments	References
16	Late Turonian-early Coniacian	Turgai Sea possibly prevents dispersal between Europe and Asia	Effect of Turgai Sea is uncertain	Baraboshkin *et al.*[240]; Akhmetiev and Beniamovski[247]; Akhmetiev *et al.*[248]
17	Late Coniacian	Landbridge across Turgai Sea region	Results from drop in sea level	Csiki-Sava *et al.*[190]; Baraboshkin *et al.*[240] (and references therein)
18	Santonian	Turgai Sea possibly prevents dispersal between Europe and Asia	Effect of Turgai Sea is uncertain	Baraboshkin *et al.*[240]; Akhmetiev and Beniamovski[247]; Akhmetiev *et al.*[248]
19	Early Campanian	Turgai Sea became deeper and is presumed to have been a more effective barrier at this time and during the late Campanian; possible re-emergence of the Apulian Route between Europe and Africa (lasting until the end of the Maastrichtian); Bering landbridge between N. America and Asia was re-established (although its presence during the Maastrichtian has been questioned)		Ezcurra and Agnolin[189]; Baraboshkin *et al.*[240]; Zanno and Makovicky[243]; Brikiatis[249]
20	Late Campanian	A landbridge might have developed between N. and S. America at this time and during the early Maastrichtian		Loewen *et al.*[263]; Prieto-Márquez[256]
21	Early Maastrichtian	Possible landbridge between N. America and Europe via the De Geer route; possible shallowing of Turgai Sea, possible loss of the Bering landbridge		Zanno and Makovicky[243]; Brikiatis[249]
22	Late Maastrichtian	Loss of the De Geer landbridge between N. America and Europe; loss of the land connection between N. and S. America		

V. BioGeoBEARS Analyses

Background

BioGeoBEARS can implement six different models of how the spatial distributions of organisms might evolve with respect to their phylogenetic relationships[105]. These models are known as DEC, DEC+J, DIVALIKE, DIVALIKE+J, BAYAREALIKe and BAYAREALIKe+J. In all models, 'd' (dispersal) and 'e' (extinction) are free parameters that represent the rate of range expansion and contraction, respectively, along a lineage. The models differ, however, in how they deal with the inheritance of ancestral ranges at cladogenesis. DEC (Dispersal, Extinction, Cladogenesis) was proposed by Ree[270] and Ree and Smith[271]. This model allows a restricted form of vicariance in which one of the areas occupied by an ancestor is passed onto one of its two daughter lineages at cladogenesis, whereas the other daughter lineage inherits all of the other areas (e.g. if ancestor z occupies areas ABCD, then daughters x and y can inherit areas A and BCD respectively). Subset speciation is also allowed in DEC (e.g. daughters x and y could inherit areas A and ABCD from z). However, vicariance in the form in which x and y inherit AB and CD respectively, is not permitted in DEC. DIVALIKE is based on DIVA (Dispersal-Vicariance Analysis) created by Ronquist[272]: however, it is termed 'DIVA-like' in BioGeoBEARS because it is a maximum likelihood implementation of the parsimony-based DIVA. DIVALIKE allows vicariance of any form to occur at cladogenesis, but prohibits subset speciation. BAYAREALIKE is a maximum likelihood implementation of BAYAREA, a Bayesian approach to biogeography proposed by Landis *et al.*[273]. In BAYAREALIKE, only range duplication is allowed at cladogenesis (e.g. ancestor z in areas ABCD, would pass ABCD to both daughters x and y). The +J versions of DEC, DIVALIKE and BAYAREALIKE, were proposed by Matzke[105,106]. J is a third free parameter that represents the rate of founder-event speciation (a process that has previously been omitted from other biogeographic analytical approaches). Founder-event speciation occurs at cladogenesis, as a result of 'jump' dispersal across a barrier, followed by allopatry (e.g. ancestor z living in areas ABCD disperses to E, so that daughters x and y occur in ABCD and E respectively after cladogenesis). BioGeoBEARS uses each of these six models to estimate the ancestral ranges of taxa, and then applies log likelihood ratio tests and AIC analyses in order to assess which model is best fitted by the data[105,106].

Analyses

We have carried out eight analyses, each of which applies the six biogeographic models outlined above. These eight analyses were generated by applying the starting, harsh, and relaxed versions of the time-stratified dispersal multiplier matrices, as well as no constraints on dispersal, to the maximum age and midpoint age phylogenetic trees. The results are summarised in Tables S25 and S26, and the ancestral range estimations for the best fitting models for each analysis are shown in the supplementary files Figures S9–S22.

Results and interpretation

For reasons of brevity, we use the analysis name abbreviations listed in the legend to Table S25 in the following discussion. The term 'node' is used in the conventional cladistic sense, but it should be noted that the ancestral range estimates at a given node indicate the area (s) occupied by that ancestor just prior to cladogenesis. The range estimates shown at 'corners' (i.e. where a branch changes direction from vertical to horizontal in the plots shown in Figures S6–S18), indicate the area (s) occupied by the lineage just after cladogenesis (see http://phylo.wikidot.com/biogeobears). The reader should also note that some caution is

required in interpreting the ancestral range estimates produced by BioGeoBEARS. Other combinations of areas might be nearly as probable as those actually shown in the plots. The accompanying figures showing pie charts (at each node or corner) summarise the relative probabilities of various area combinations that could occur. Thus, the area(s) shown at a node or corner as the most probable range estimate might have a relatively low probability, but this probability is higher than that of any other possible range.

Comparisons of the results produced by the maximum age and midpoint age trees indicate that the differences in estimated node dates have had little impact on the biogeographic analyses (especially the unconstrained ones). The main effect is that, whereas BAYAREALIKE and BAYAREALIKE+J models are preferred in the MXst, MXha and MXre analyses, DEC+J (sometimes also with BAYAREALIKE+J) is supported as best fitting the data in the MDst and MDre analyses. Whether or not constraints on dispersal (based on palaeogeography) are applied, and the nature of those constraints, has a more marked effect (see below). Table S25 shows that, when no constraints on dispersal are applied, the data fit the three +J models significantly better than they do the models that lack founder-event speciation. Furthermore, the three +J models fit the data almost equally well. Although there is a slightly better fit between the data and the DIVALIKE+J model (based on the lowest AIC value), the AIC analyses indicate that DEC+J and BAYAREALIKE+J perform nearly as well, and we suggest that there are no grounds for favouring one of these +J models over either of the other two. Thus, when no constraint on dispersal is applied, the data are insufficient to allow a determination of the extent to which continent-scale vicariance did, or did not, play a role in the evolution of Cretaceous titanosauriforms, but do support a major role for founder-event speciation. In terms of the biogeographic origins of the two Australian lineages, the ancestral range estimations support a dispersal event from Asia to Australia (Table S26). This is based on the estimation of Asia as the range for the most recent common ancestor of *Diamantinasaurus*+*Savannasaurus* and other titanosaurs, and that of *Wintonotitan* and other somphospondylans, in the MXun and MDun analyses (Figures S9–S14).

Table S25 shows that, when time-stratified dispersal constraints are implemented, the +J version of each model still performs better than the non+J version in most cases. However, the support for +J models over non+J ones is greatly reduced: indeed, there are several instances when there is no statistically significant difference between them (e.g. BAYAREALIKE and BAYAREALIKE+J in the MDre) or when the non+J version is actually better supported based on AIC scores (e.g. BAYAREALIKE and BAYAREALIKE+J in the MDst and MDha). Thus, application of dispersal constraints significantly reduces the role of founder-event speciation in the biogeographic evolution of macronarian sauropods. This is a reasonable result given that Matzke[105] reported that estimated rates of founder-event speciation are 2–4 times higher in island clades compared to continental ones. The second major effect of imposing palaeogeography-based dispersal constraints is that BAYAREALIKE and BAYAREALIKE+J are much better supported than DEC, DEC+J, DIVALIKE and DIVALIKE+J in the MXst, MXha, MXre and MDha analyses (Table S25). If this accurately reflects the processes governing Cretaceous sauropod evolution, then it suggests very little role for continent-scale vicariance. However, this result is sensitive to how the uncertainty in taxon ages is treated: DEC+J is the best fitting model in the MDst and MDre analyses.

The palaeogeographic constraints have also had a marked impact on the biogeographic histories estimated for the two lineages leading to *Diamantinasaurus*+*Savannasaurus* and *Wintonotitan* (Compare Figs S9–14 with S15–22). With regard to the most recent common ancestor of *Diamantinasaurus*+*Savannasaurus* and other titanosaurs, all of the best supported

constrained MX analyses estimate a geographic range encompassing at least Asia and South America (Table S26). In the MDst and MDre analyses, where DEC+J is supported, this ancestral range is estimated to include Asia, Africa, Indo-Madagascar, South America and Australia (Figs S18 and S21). A virtually identical set of ancestral area estimations is generated for the most recent common ancestor of *Wintonotitan* and other somphospondylans (Table S26). These results suggest that the two Australian lineages were derived from ancestral stocks that were widespread across several continents during the Early Cretaceous (i.e. prior to the Aptian). This is a reasonable interpretation even for those results where these ancestors are estimated to have lived solely in Asia+South America at this time. Palaeogeographic information (see above) demonstrates that a single area composed exclusively of Asia and South America did not exist during the Mesozoic: therefore, an estimated ancestral range comprising just these two areas is most plausibly interpreted as representing a much more widespread range that has been obscured by missing data caused by sampling failure)). If the Australian sauropod lineages belong to groups that were widespread during the pre-Aptian Early Cretaceous, then their existence during the early Late Cretaceous can be explained through processes such as geodispersal and regional extinction and does not require the direct trans-oceanic dispersal from Asia that is implied by the results of the unconstrained analyses.

The results outlined above raise the issue of whether we should give preference to those generated with, or without, palaeogeographic constraints. Here, we propose that the constrained analyses should be given more credence, for several reasons:

1. The unconstrained analyses support some rather unlikely events in sauropod biogeographic history. For example, it seems improbable that such large-bodied terrestrial animals could have crossed thousands of kilometres of ocean in order to disperse from Asia to Australia directly.

2. The constrained analyses have produced more realistic estimates of the rates of dispersal (d), extinction (e) and founder event speciation (J), than the unconstrained ones. In the latter, when the +J models are applied, d and e are estimated to be 0, so that J is a dominant factor (see Figures S9–S14). It seems improbable that range expansion and contraction along evolving lineages played no role in determining the biogeographic ranges of sauropods, and that the latter were largely the product of founder-event speciation. As noted by Matzke[105], there is some prima facie evidence that J has lower values in continental clades compared to island ones. Thus, the constrained analyses, with their higher values for d and e, and lower values for J, appear to provide more realistic estimates of the factors controlling the spatial distribution of a terrestrial continental clade such as sauropods.

3. The addition of palaeogeographic data to a biogeographic analysis can be supported on the basis of a ‘total evidence’ argument. We know that sauropod evolution took place against the backdrop of profound changes in palaeogeography resulting from the break-up of Pangaea and fluctuations in sea level. Provided it is reasonable to assume that sauropods could not cross wide ocean barriers, we are justified in informing the biogeographic analyses of the positions and ages of such barriers.

If our constrained analyses have produced accurate estimates of macronarian biogeographic history, then our results have four main implications.

1. The main processes controlling the geographic distributions of Cretaceous macronarians appear to be geodispersal (which produced widespread clades during the earliest Cretaceous),

and regional extinction (which was at least partially responsible for continent-scale endemism in the Late Cretaceous). Vicariance driven by continental fragmentation and changes in sea level might also have played a role, but the support for this is sensitive to how the uncertainty in terminal taxon ages is handled (i.e. DEC+J is only the best fitting model when mid-point ages are used). It would be premature, however, to dismiss the role of vicariance. First, several studies (e.g. Upchurch *et al.*[188], Ezcurra and Agnolin[189]) have found statistically significant area relationships, in several dinosaurian clades, indicative of vicariance patterns. Second, it is also possible that a poorly sampled fossil record has destroyed some of the signal for vicariance, and it should be noted that the latter process is perhaps more sensitive to 'noise' in a data set than is, for example, dispersal[229].

2. Although the existence of one or more pre-Aptian titanosauriform radiations (extending across several continents) is compatible with our current knowledge of the fossil record, our ancestral range estimates nonetheless imply a hitherto unappreciated diversity of taxa that have yet to be found. For example, several of our results suggest that true titanosaurian taxa were present in the pre-Aptian Cretaceous of Indo-Madagascar, even though at present they are only known in this region from the latest Cretaceous. Presumably these gaps in our knowledge reflect poor sampling during the early Cretaceous, particularly in Gondwana (e.g. Upchurch *et al.*[36]), and in the mid-Cretaceous in general[274].

3. Our results mean that we must envisage a series of regional extinction events occurring from the Aptian onwards, as implied by the range contractions seen in our constrained biogeographic analyses. This scenario of earlier cosmopolitanism followed by regional extinction is reminiscent of the proposals made by Sereno *et al.*[275], which attempted to explain Late Cretaceous continent-scale endemism among dinosaurs. The potential role of mid-Cretaceous climate change in some of these regional extinctions is discussed briefly in the main text of this article.

4. The apparent close similarity between the mid-Cretaceous dinosaurian faunas of East Asia and Australia (which is discussed in our main text) has been explained as a result of long-distance dispersals by some workers[276]. However, our results only support such an interpretation when palaeogeographic data are ignored. Moreover, our phylogenetic results do not reconstruct either of the two Australian lineages as having sister-taxa in East Asia, as would be predicted by the long-distance dispersal hypothesis. Here, we offer an alternative biogeographic scenario that provides a more plausible explanation for the occurrence of the Australian taxa. We suggest that the Australian lineages represent mid-Cretaceous remnants of clades that were widespread during the Early Cretaceous, with these lineages entering East Gondwana via dispersal from South America (presumably across Antarctica).

Table S25. Summary of results and statistical comparisons between the six biogeographic models applied in the BioGeoBEARS analyses. N.B. the ’Ratio’ in the AIC analyses is the ratio of the AIC weight for the +J version of a model to the AIC weight for the non+J version of the same model (e.g. DEC+J/DEC). Abbreviations: ha, harsh dispersal multiplier; MD, midpoint age tree; MX, maximum age tree; re, relaxed dispersal multiplier; st, starting dispersal multiplier; un, unconstrained (i.e. no dispersal multiplier applied). An asterisk (*) marks those models that are regarded as best fitting the data in each analysis.

Analysis	Model	LnL	Ln likelihood ratio test		AIC Analysis		
			D statistic	P value	AIC	AICwt	Ratio
MXst	DEC	-138.9			281.8	$6.6e^{-6}$	
	DEC+J	-126	25.84	$3.7e^{-7}$	257.9	1.00	$1.5e^{5}$
	DIVALIKE	-170.6			345.3	$1.5e^{-17}$	
	DIVALIKE+J	-130.9	79.49	$4.8e^{-19}$	267.8	1	$6.7e^{16}$
	BAYAREALiKE	-130.2			264.3	$2.7e^{-5}$	
	*BAYAREALiKE+J	-118.6	23.05	$1.6e^{-6}$	243.3	1.00	$3.7e^{4}$
MXha	DEC	-159.7			323.3	0.0003	
	DEC+J	-150.6	18.21	$2.0e-^{5}$	307.1	1.00	3304
	DIVALIKE	-189.3			382.6	$1.1e^{-11}$	
	DIVALIKE+J	-163.1	52.46	$4.4e^{-13}$	332.2	1.00	$9.1e^{10}$
	BAYAREALiKE	-136.3			304.4	0.04	
	*BAYAREALiKE+J	-132.1	8.43	0.0037	280.7	0.96	24.93
MXre	DEC	-139.4			282.9	$3.7e^{-6}$	
	DEC+J	-125.9	26.99	$2.0e^{-7}$	257.9	1.00	$2.7e^{5}$
	DIVALIKE	-172.6			349.2	$1.5e^{-17}$	
	DIVALIKE+J	-132.9	79.5	$4.8e^{-19}$	271.7	1.00	$6.74e^{16}$
	BAYAREALiKE	-130.3			264.7	$5.7e^{-6}$	
	*BAYAREALiKE+J	-117.2	26.15	$3.2e^{-7}$	240.5	1.00	$1.8e^{5}$
MXun	DEC	-121.3			250.5	$2.5e^{-19}$	
	*DEC+J	-79.48	87.67	$7.7e^{-21}$	165	1	$4.01e^{18}$
	DIVALIKE	-120.8			245.5	$1.7e^{-18}$	
	*DIVALIKE+J	-78.85	83.83	$5.4e^{-20}$	163.7	1	$5.88e^{17}$
	BAYAREALiKE	-149.7			303.5	$4.5e^{-30}$	
	*BAYAREALiKE+J	-81.17	137.1	$1.1e^{-31}$	168.3	1	$2.21e^{29}$
MDst	DEC	-136.9			277.8	0.0028	
	*DEC+J	-130	13.78	0.0002	266	1.00	361.8
	DIVALIKE	-173.4			350.9	$2.7e^{-11}$	
	DIVALIKE+J	-148.1	50.69	$1.1e^{-12}$	302.2	1.00	$3.73e^{10}$
	*BAYAREALiKE	-131.9			267.7	0.60	
	*BAYAREALiKE+J	-131.3	1.17	0.28	268.6	0.40	0.67
MDha	DEC	-146.6			297.2	0.04	
	DEC+J	-142.3	8.61	0.0033	290.6	0.96	27.3
	DIVALIKE	-186			375.9	$6.3e^{-11}$	
	DIVALIKE+J	-161.5	48.98	$2.6e^{-12}$	328.9	1.00	$1.59e^{10}$
	*BAYAREALiKE	-136			275.9	0.73	
	*BAYAREALiKE+J	-136	-0.0002	1	277.9	0.27	0.37
MDre	DEC	-138.2			280.4	0.001	
	*DEC+J	-130.2	15.88	$6.7e^{-5}$	266.5	1.00	1035
	DIVALIKE	-175.9			355.7	$1.0e^{-14}$	
	DIVALIKE+J	-142.6	66.42	$3.6e^{-16}$	291.3	1.00	$9.76e^{13}$
	*BAYAREALiKE	-132.1			268.1	0.48	
	*BAYAREALiKE+J	-131	2.17	0.14	268	0.52	1.09
MDun	DEC	-121.6			247.2	$1.4e^{-18}$	
	*DEC+J	-79.48	84.46	$4.3e^{-20}$	165	1	$7.29e^{17}$
	DIVALIKE	-122.7			249.3	$2.6e^{-19}$	
	*DIVALIKE+J	-78.85	87.62	$7.9e^{-21}$	163.7	1	$3.91e^{18}$
	BAYAREALiKE	-150.3			304.6	$2.6e^{-30}$	
	*BAYAREALiKE+J	-81.17	138.2	$6.5e^{-32}$	168.3	1	$3.83e^{29}$

Table S26. Ancestral range estimations for Australian sauropods, based on the results of the models which best fit the data (i.e. those marked with an asterisk [*] in Table S25). Range estimates are based on the data shown in Figures S6–S17. 'First node' refers to the estimated range of the most recent common ancestor of the Australian lineage and other sauropods. For *Diamantinasaurus*+*Savannasaurus*, the first node is the most recent common ancestor of *Diamantinasaurus* and *Opisthocoelicaudia*. For *Wintonotitan*, the first node is the point where this taxon diverges from other sauropods. 'First corner' refers to the range estimation for the lineage leading to the Australian sauropods immediately above the first node. For an explanation of the difference between 'node' and a 'corner' range estimations, see the main supplementary text above. A query (?) marks those range estimations which have less than a 50% probability based on the pie charts shown in Figures S6–S17.

	Diamantinasaurus + Savannasaurus		***Wintonotitan***	
**Analysis**	**First corner**	**First node**	**First corner**	**First node**
MXst BAYAREALIKE+J (Fig. S15)	Australia	Asia+South America	Australia	Asia+South America
MXha BAYAREALIKE+J (Fig. S16)	Asia+South America+Australia	Asia+South America+Australia	Asia+South America+Australia	Asia+South America+Australia
MXre BAYAREALIKE+J (Fig. S17)	Australia	Asia+South America	Australia	Asia+South America
MXun DEC+J (Fig. S9) DIVALIKE+J (Fig. S10) BAYAREALIKE+J (Fig. S11)	Australia	Asia	Australia	Asia
MDst DEC+J (Fig. S18)	Australia	Asia+Africa+Indo-Madagascar+South America+Australia	Australia	Asia+Africa+Indo-Madagascar+South America+Australia
MDst BAYAREALIKE (Fig. S19)	Asia+South America	Asia+South America	Asia+South America	Asia+South America
MDst BAYAREALIKE+J (Fig. S19)	Australia	Asia+South America	Australia	Asia+South America
MDha BAYAREALIKE (Fig. 20) BAYAREALIKE+J (Fig. 20)	Asia+South America+Australia	Asia+South America+Australia	Asia+South America+Australia	Asia+South America+Australia
MDre DEC+J Fig. S21)	Australia	Asia+Africa+Indo-Madagascar+South America+Australia	Australia	Asia+Africa+Indo-Madagascar+South America+Australia
MDre BAYAREALIKE (Fig. S22)	Asia+South America	Asia+South America	Asia+South America	Asia+South America
MDre BAYAREALIKE+J (Fig. S22)	Australia	Asia+South America	Australia	Asia+South America
MDun DEC+J (Fig. S12) DIVALIKE+J (Fig. S13) BAYLIKE+J (Fig. S14)	Australia	Asia	Australia	Asia

References

1 Hocknull, S. A. *et al.* New mid-Cretaceous (latest Albian) dinosaurs from Winton, Queensland, Australia. *PLoS ONE* **4**, e6190, doi:10.1371/journal.pone.0006190 (2009).

2 Poropat, S. F. *et al.* Revision of the sauropod dinosaur *Diamantinasaurus matildae* Hocknull et al. 2009 from the middle Cretaceous of Australia: implications for Gondwanan titanosauriform dispersal. *Gondwana Research* **27**, 995–1033, doi:10.1016/j.gr.2014.03.014 (2015).

3 Coombs, W. P., Jr. & Molnar, R. E. Sauropoda (Reptilia, Saurischia) from the Cretaceous of Queensland. *Memoirs of the Queensland Museum* **20**, 351–373 (1981).

4 Molnar, R. E. A catalogue of fossil amphibians and reptiles in Queensland. *Memoirs of the Queensland Museum* **20**, 613–633 (1982).

5 Lees, T. Catalogue of type, figured and mentioned fossil fish, amphibians and reptiles held by the Queensland Museum. *Memoirs of the Queensland Museum* **22**, 265–288 (1986).

6 Molnar, R. E. in *VII International Symposium on Mesozoic Terrestrial Ecosystems: Asociacíon Paleontológica Argentina Publicación Especial No. 7* (ed Héctor A Leanza) 139–144 (Asociacion Paleontologica Argentina, 2001).

7 Molnar, R. E. & Salisbury, S. W. in *Thunder-lizards: The Sauropodomorph Dinosaurs Life of the Past* (eds Virginia Tidwell & Kenneth Carpenter) Ch. 20, 454–465 (Indiana University Press, 2005).

8 Poropat, S. F. *et al.* Reassessment of the non-titanosaurian somphospondylan *Wintonotitan wattsi* (Dinosauria: Sauropoda: Titanosauriformes) from the mid-Cretaceous Winton Formation, Queensland, Australia. *Papers in Palaeontology* **1**, 59–106, doi:10.1002/spp2.1004 (2015).

9 Molnar, R. E. New morphological information about Cretaceous sauropod dinosaurs from the Eromanga Basin, Queensland, Australia. *Alcheringa* **35**, 329–339, doi:10.1080/03115518.2011.533978 (2011).

10 Molnar, R. E. Taphonomic observations on eastern Australian Cretaceous sauropods. *Alcheringa* **34**, 421–429, doi:10.1080/03115518.2010.497258 (2010).

11 Molnar, R. E. Reflections on the Mesozoic of Australia. *Mesozoic Vertebrate Life* **1**, 47–60 (1980).

12 Longman, H. A. A new dinosaur from the Queensland Cretaceous. *Memoirs of the Queensland Museum* **10**, 131–144 (1933).

13 Molnar, R. Australian late Mesozoic terrestrial tetrapods: some implications. *Mémoires de la Société Géologique de France (Nouvelle Série)* **139**, 131–143 (1980).

14 Bartholomai, A. & Molnar, R. E. *Muttaburrasaurus*, a new iguanodontid (Ornithischia: Ornithopoda) dinosaur from the Lower Cretaceous of Queensland. *Memoirs of the Queensland Museum* **20**, 319–349 (1981).

15 Thulborn, T. in *The Antipodean Ark* (eds Suzanne Hand & Michael Archer) 44–46 (Angus & Robertson, 1987).

16 Molnar, R. E. in *Fossil Vertebrate Record of Australasia* (eds Patricia Vickers Rich & E M Thompson) 169–225 (Monash University Press, 1982).

17 Molnar, R. E. in *Vertebrate Palaeontology of Australasia* (eds Patricia Vickers-Rich, Jennifer M Monaghan, Robert F Baird, & Thomas Hewitt Rich) 605–702 (Pioneer Design Studio, 1991).

18 Molnar, R. E. Sauropod (Saurischia: Dinosauria) material from the Early Cretaceous Griman Creek Formation of the Surat Basin, Queensland, Australia. *Alcheringa* **35**, 303–307, doi:10.1080/03115518.2010.533975 (2011).

19 Thulborn, T., Hamley, T. & Foulkes, P. in *Aspects of Sauropod Paleobiology; Gaia, 10* (eds Martin G Lockley, Vanda F Santos, Christian A Meyer, & Adrian P Hunt) 85–94 (1994).

20 Thulborn, T. Impact of sauropod dinosaurs on lagoonal substrates in the Broome Sandstone (Lower Cretaceous), Western Australia. *PLoS ONE* **7**, e36208, doi:10.1371/journal.pone.0036208 (2012).

21 Longman, H. A. A giant dinosaur from Durham Downs, Queensland. *Memoirs of the Queensland Museum* **8**, 183–194 (1926).

22 Longman, H. A. Australia's largest fossil. The *Rhoetosaurus* dinosaur. *Australian Museum Magazine* **3**, 97–102 (1927).

23 Longman, H. A. The giant dinosaur: *Rhoetosaurus brownei*. *Memoirs of the Queensland Museum* **9**, 1–18 (1927).

24 Longman, H. A. Palaeontological notes. *Memoirs of the Queensland Museum* **9**, 249–250 (1929).

25 Wade, M. in *Prehistoric Animals of Australia* (eds Susan Quirk & Michael Archer) 26–27 (The Australian Museum, 1983).

26 Thulborn, R. A. in *Kadimakara: Extinct Vertebrates of Australia* (eds Patricia Vickers Rich, Gerard Frederick van Tets, & Frank Knight) 166–171 (Princeton University Press, 1985).

27 Nair, J. P. & Salisbury, S. W. New anatomical information on *Rhoetosaurus brownei* Longman, 1926, a gravisaurian sauropodomorph dinosaur from the Middle Jurassic of Queensland, Australia. *Journal of Vertebrate Paleontology* **32**, 369–394, doi:10.1080/02724634.2012.622324 (2012).

28 Long, J. A. First dinosaur bones from Western Australia. *The Beagle, Records of the Northern Territory Museum of Arts and Sciences* **9**, 21–27 (1992).

29 Molnar, R. E. & Wiffen, J. A Late Cretaceous polar dinosaur fauna from New Zealand. *Cretaceous Research* **15**, 689–706, doi:10.1006/cres.1994.1038 (1994).

30 Wiffen, J. Dinosaurian palaeobiology: a New Zealand perspective. *Memoirs of the Queensland Museum* **39**, 725–731 (1996).

31 Molnar, R. E. & Wiffen, J. A presumed titanosaurian vertebra from the Late Cretaceous of North Island, New Zealand. *Arquivos do Museu Nacional, Rio de Janeiro* **65**, 505–510 (2007).

32 Olivero, E. B., Ponce, J. J., Marsicano, C. A. & Martinioni, D. R. Depositional settings of the basal López de Bertodano Formation, Maastrichtian, Antarctica. *Revista de la Asociación Geológica Argentina* **62**, 521–529 (2007).

33 Reguero, M. A., Tambussi, C. P., Coria, R. A. & Marenssi, S. A. in *Antarctic Palaeoenvironments and Earth-Surface Processes. Geological Society Special Publication, 381* (eds Michael J Hambrey *et al.*) 99–116 (Geological Society, 2013).

34 Cerda, I. A. *et al.* The first record of a sauropod dinosaur from Antarctica. *Naturwissenschaften* **99**, 83–87, doi:10.1007/s00114-011-0869-x (2012).

35 Mannion, P. D., Upchurch, P., Barnes, R. N. & Mateus, O. Osteology of the Late Jurassic Portuguese sauropod dinosaur *Lusotitan atalaiensis* (Macronaria) and the evolutionary history of basal titanosauriforms. *Zoological Journal of the Linnean Society* **168**, 98–206, doi:10.1111/zoj.12029 (2013).

36 Upchurch, P., Mannion, P. D. & Taylor, M. P. The anatomy and phylogenetic relationships of "*Pelorosaurus*" *becklesii* (Neosauropoda, Macronaria) from the Early Cretaceous of England. *PLoS ONE* **10**, e0125819, doi:10.1371/journal.pone.0125819 (2015).

37 Royo-Torres, R. *et al.* The anatomy, phylogenetic relationships, and stratigraphic position of the Tithonian–Berriasian Spanish sauropod dinosaur *Aragosaurus*

ischiaticus. *Zoological Journal of the Linnean Society* **171**, 623–655, doi:10.1111/zoj.12144 (2014).

38 Carrano, M. T. & D'Emic, M. D. Osteoderms of the titanosaur sauropod dinosaur *Alamosaurus sanjuanensis* Gilmore, 1922. *Journal of Vertebrate Paleontology* **35**, e901334, doi:10.1080/02724634.2014.901334 (2015).

39 Powell, J. E. Revision of South American titanosaurid dinosaurs: palaeobiological, palaeobiogeographical and phylogenetic aspects. *Records of the Queen Victoria Museum* **111**, 1–173 (2003).

40 Martínez, R., Giménez, O., Rodríguez, J., Luna, M. & Lamanna, M. C. An articulated specimen of the basal titanosaurian (Dinosauria: Sauropoda) *Epachthosaurus sciuttoi* from the early Late Cretaceous Bajo Barreal Formation of Chubut Province, Argentina. *Journal of Vertebrate Paleontology* **24**, 107–120, doi:10.1671/9.1 (2004).

41 Calvo, J. O., Porfiri, J. D., González Riga, B. J. & Kellner, A. W. A. Anatomy of *Futalognkosaurus dukei* Calvo, Porfiri, González Riga & Kellner, 2007 (Dinosauria, Titanosauridae) from the Neuquén Group (Late Cretaceous), Patagonia, Argentina. *Arquivos do Museu Nacional, Rio de Janeiro* **65**, 511–526 (2007).

42 Calvo, J. O. in *The History of Life: A View from the Southern Hemisphere. 4th International Palaeontological Congress Abstract Volume* (ed E Cerdeño) 325A (International Palaeontological Association, 2014).

43 Jain, S. L. & Bandyopadhyay, S. New titanosaurid (Dinosauria: Sauropoda) from the Late Cretaceous of central India. *Journal of Vertebrate Paleontology* **17**, 114–136, doi:10.1080/02724634.1997.10010958 (1997).

44 Wilson, J. A. Sauropod dinosaur phylogeny: critique and cladistic analysis. *Zoological Journal of the Linnean Society* **136**, 217–276, doi:10.1046/j.1096-3642.2002.00029.x (2002).

45 Curry Rogers, K. The postcranial osteology of *Rapetosaurus krausei* (Sauropoda: Titanosauria) from the Late Cretaceous of Madagascar. *Journal of Vertebrate Paleontology* **29**, 1046–1086, doi:10.1671/039.029.0432 (2009).

46 Wilson, J. A., Malkani, M. S. & Gingerich, P. D. in *Indian Non-Marine Late Cretaceous: Advances and Challenges Gondwanan Geological Magazine Special Volume* (ed Dhananjay M Mohabey) 101–109 (Gondwana Geological Society, 2005).

47 Wilson, J. A., D'Emic, M. D., Curry Rogers, K. A., Mohabey, D. M. & Sen, S. Reassessment of the sauropod dinosaur *Jainosaurus* (="*Antarctosaurus*") *septentrionalis* from the Upper Cretaceous of India. *Contributions from the Museum of Paleontology, University of Michigan* **32**, 17–40 (2009).

48 Berman, D. S. & Jain, S. L. The braincase of a small sauropod dinosaur (Reptilia: Saurischia) from the Upper Cretaceous Lameta Group, central India, with review of Lameta Group localities. *Annals of the Carnegie Museum* **51**, 405–422 (1982).

49 Calvo, J. O., González Riga, B. J. & Porfiri, J. D. A new titanosaur sauropod from the Late Cretaceous of Neuquén, Patagonia, Argentina. *Arquivos do Museu Nacional, Rio de Janeiro* **65**, 485–504 (2007).

50 Wilson, J. A. Redescription of the Mongolian sauropod *Nemegtosaurus mongoliensis* Nowinski (Dinosauria: Saurischia) and comments on Late Cretaceous sauropod diversity *Journal of Systematic Palaeontology* **3**, 283–318, doi:10.1017/S1477201905001628 (2005).

51 Zaher, H. *et al.* A complete skull of an Early Cretaceous sauropod and the evolution of advanced titanosaurians. *PLoS ONE* **6**, e16663, doi:10.1371/journal.pone.0016663 (2011).

52 Upchurch, P., Barrett, P. M. & Dodson, P. in *The Dinosauria: Second Edition* (eds David Bruce Weishampel, Peter Dodson, & Halszka Osmólska) 259–322 (University of California Press, 2004).

53 Upchurch, P. & Mannion, P. D. (unpublished data).

54 González Riga, B. J., Previtera, E. & Pirrone, C. A. *Malarguesaurus florenciae* gen. et sp. nov., a new titanosauriform (Dinosauria, Sauropoda) from the Upper Cretaceous of Mendoza, Argentina. *Cretaceous Research* **30**, 135–148, doi:10.1016/j.cretres.2008.06.006 (2009).

55 Borsuk-Białynicka, M. A new camarasaurid sauropod *Opisthocoelicaudia skarzynskii* gen. n., sp. n. from the Upper Cretaceous of Mongolia. *Palaeontologia Polonica* **37**, 5–64 (1977).

56 D'Emic, M. D. Revision of the sauropod dinosaurs of the Lower Cretaceous Trinity Group, southern USA, with the description of a new genus. *Journal of Systematic Palaeontology* **11**, 707–726, doi:10.1080/14772019.2012.667446 (2013).

57 Upchurch, P. The phylogenetic relationships of sauropod dinosaurs. *Zoological Journal of the Linnean Society* **124**, 43–103, doi:10.1111/j.1096-3642.1998.tb00569.x (1998).

58 Whitlock, J. A. A phylogenetic analysis of Diplodocoidea (Saurischia: Sauropoda). *Zoological Journal of the Linnean Society* **161**, 872–915, doi:10.1111/j.1096-3642.2010.00665.x (2011).

59 Marpmann, J. S., Carballido, J. L., Sander, P. M. & Knötschke, N. Cranial anatomy of the Late Jurassic dwarf sauropod *Europasaurus holgeri* (Dinosauria, Camarasauromorpha): ontogenetic changes and size dimorphism. *Journal of Systematic Palaeontology* **13**, 221–263, doi:10.1080/14772019.2013.875074 (2015).

60 Carballido, J. L., Salgado, L., Pol, D., Canudo, J. I. & Garrido, A. A new basal rebbachisaurid (Sauropoda, Diplodocoidea) from the Early Cretaceous of the Neuquén Basin; evolution and biogeography of the group. *Historical Biology* **24**, 631–654, doi:10.1080/08912963.2012.672416 (2012).

61 Curry Rogers, K. A. in *The Sauropods: Evolution and Paleobiology* (eds Kristina Ann Curry Rogers & Jeffrey Alan Wilson) 50–103 (University of California Press, 2005).

62 Upchurch, P. The evolutionary history of sauropod dinosaurs. *Philosophical Transactions: Biological Sciences* **349**, 365–390, doi:10.1098/rstb.1995.0125 (1995).

63 Tschopp, E., Mateus, O. & Benson, R. B. J. A specimen-level phylogenetic analysis and taxonomic revision of Diplodocidae (Dinosauria, Sauropoda). *PeerJ* **3**, e857, doi:10.7717/peerj.857 (2015).

64 Tschopp, E. & Mateus, O. The skull and neck of a new flagellicaudatan sauropod from the Morrison Formation and its implication for the evolution and ontogeny of diplodocid dinosaurs. *Journal of Systematic Palaeontology* **11**, 853–888, doi:10.1080/14772019.2012.746589 (2013).

65 Whitlock, J. A., Wilson, J. A. & Lamanna, M. C. Description of a nearly complete juvenile skull of *Diplodocus* (Sauropoda: Diplodocoidea) from the Late Jurassic of North America. *Journal of Vertebrate Paleontology* **30**, 442–457 (2010).

66 Wilson, J. A. & Sereno, P. C. Early evolution and higher-level phylogeny of sauropod dinosaurs. *Society of Vertebrate Paleontology Memoir* **5**, 1–68, doi:10.1080/02724634.1998.10011115 (1998).

67 Gauthier, J. in *The Origin of Birds and the Evolution of Flight: Memoirs of the California Academy of Sciences, 8* Vol. 8 (ed Kevin Padian) 1–55 (1986).

68 Harris, J. D. The significance of *Suuwassea emilieae* (Dinosauria: Sauropoda) for flagellicaudatan intrarelationships and evolution. *Journal of Systematic Palaeontology* **4**, 185-198 (2006).
69 Curry Rogers, K. & Forster, C. A. The skull of *Rapetosaurus krausei* (Sauropoda: Titanosauria) from the Late Cretaceous of Madagascar. *Journal of Vertebrate Paleontology* **24**, 121–144, doi:10.1671/A1109-10 (2004).
70 Berman, D. S. & McIntosh, J. S. Skull and relationships of the Upper Jurassic sauropod *Apatosaurus* (Reptilia, Saurischia). *Bulletin of the Carnegie Museum of Natural History* **8**, 1–35 (1978).
71 Mannion, P. D., Upchurch, P., Mateus, O., Barnes, R. N. & Jones, M. E. H. New information on the anatomy and systematic position of *Dinheirosaurus lourinhanensis* (Sauropoda: Diplodocoidea) from the Late Jurassic of Portugal, with a review of European diplodocoids. *Journal of Systematic Palaeontology* **10**, 521–551 (2012).
72 McIntosh, J. S. in *The Dinosauria* (eds David Bruce Weishampel, Peter Dodson, & Halszka Osmólska) 345–401 (University of California Press, 1990).
73 Remes, K. Taxonomy of Late Jurassic diplodocid sauropods from Tendaguru (Tanzania). *Fossil Record* **12**, 23–46, doi:10.1002/mmng.200800008 (2009).
74 Paulina Carabajal, A. Neuroanatomy of titanosaurid dinosaurs from the Upper Cretaceous of Patagonia, with comments on endocranial variability within Sauropoda. *The Anatomical Record* **295**, 2141–2156, doi:10.1002/ar.22572 (2012).
75 Salgado, L. & Calvo, J. O. Evolution of titanosaurid sauropods. II: The cranial evidence. *Ameghiniana* **34**, 33–48 (1997).
76 González Riga, B. J. & Ortíz David, L. A new titanosaur (Dinosauria, Sauropoda) from the Upper Cretaceous (Cerro Lisandro Formation) of Mendoza Province, Argentina. *Ameghiniana* **51**, 3–25, doi:10.5710/AMEGH.26.12.1013.1889 (2014).
77 Upchurch, P., Barrett, P. M. & Galton, P. M. in *Evolution and Palaeobiology of Early Sauropodomorph Dinosaurs; Special Papers in Palaeontology, 77* (eds Paul Michael Barrett & David J Batten) 57–90 (2007).
78 Rauhut, O. W. M., Remes, K., Fechner, R., Cladera, G. & Puerta, P. Discovery of a short-necked sauropod dinosaur from the Late Jurassic period of Patagonia. *Nature* **435**, 670–672, doi:10.1038/nature03623 (2005).
79 González Riga, B. J. Nuevos restos fósiles de *Mendozasaurus neguyelap* (Sauropoda, Titanosauria) del Cretácico Tardío de Mendoza, Argentina. *Ameghiniana* **42**, 535–548 (2005).
80 D'Emic, M. D. Early evolution of titanosauriform sauropod dinosaurs. *Zoological Journal of the Linnean Society* **166**, 624–671, doi:10.1111/j.1096-3642.2012.00853.x (2012).
81 Salgado, L., Coria, R. A. & Calvo, J. O. Evolution of titanosaurid sauropods. I: Phylogenetic analysis based on the postcranial evidence. *Ameghiniana* **34**, 3–32 (1997).
82 González Riga, B. J. A new titanosaur (Dinosauria, Sauropoda) from the Upper Cretaceous of Mendoza Province, Argentina. *Ameghiniana* **40**, 155–172 (2003).
83 Gallina, P. A. Notes on the axial skeleton of the titanosaur *Bonitasaura salgadoi* (Dinosauria-Sauropoda). *Anais da Academia Brasileira de Ciências* **83**, 235–245 (2011).
84 Gallina, P. A. & Apesteguía, S. Cranial anatomy and phylogenetic position of the titanosaurian sauropod *Bonitasaura salgadoi*. *Acta Palaeontologica Polonica* **56**, 45–60, doi:10.4202/app.2010.0011 (2011).
85 Salgado, L., Apesteguía, S. & Heredia, S. E. A new specimen of *Neuquensaurus australis*, a Late Cretaceous saltasaurine titanosaur from north Patagonia. *Journal of*

Vertebrate Paleontology **25**, 623–634, doi:10.1671/0272-4634(2005)025[0623:ANSONA]2.0.CO;2 (2005).
86 D'Emic, M. D. & Wilson, J. A. New remains attributable to the holotype of the sauropod dinosaur *Neuquensaurus australis*, with implications for saltasaurine systematics. *Acta Palaeontologica Polonica* **56**, 61–73, doi:10.4202/app.2009.0149 (2011).
87 D'Emic, M. D. *et al.* Osteology of *Huabeisaurus allocotus* (Sauropoda: Titanosauriformes) from the Upper Cretaceous of China. *PLoS ONE* **8**, e69375, doi:10.1371/journal.pone.0069375 (2013).
88 Santucci, R. M. & Arruda-Campos, A. C. A new sauropod (Macronaria, Titanosauria) from the Adamantina Formation, Bauru Group, Upper Cretaceous of Brazil and the phylogenetic relationships of Aeolosaurini. *Zootaxa* **3085**, 1–33 (2011).
89 Mannion, P. D. & Calvo, J. O. Anatomy of the basal titanosaur (Dinosauria, Sauropoda) *Andesaurus delgadoi* from the mid-Cretaceous (Albian–early Cenomanian) Río Limay Formation, Neuquén Province, Argentina: implications for titanosaur systematics. *Zoological Journal of the Linnean Society* **163**, 155–181, doi:10.1111/j.1096-3642.2011.00699.x (2011).
90 Kellner, A. W. A., Campos, D. d. A. & Trotta, M. N. F. Description of a titanosaurid caudal series from the Bauru Group, Late Cretaceous of Brazil. *Arquivos do Museu Nacional, Rio de Janeiro* **63**, 529–564 (2005).
91 Powell, J. E. in *Anais do X Congresso Brasileiro de Paleontología* 155–171 (1987).
92 Sanz, J. L., Powell, J. E., Le Loeuff, J., Martínez, R. & Pereda Suberbiola, X. Sauropod remains from the Upper Cretaceous of Laño (northcentral Spain), titanosaur phylogenetic relationships. *Estudios del Museo de Ciencias Naturales de Alava* **14**, 235–255 (1999).
93 Bonaparte, J. F., González Riga, B. J. & Apesteguía, S. *Ligabuesaurus leanzai* gen. et sp. nov. (Dinosauria, Sauropoda), a new titanosaur from the Lohan Cura Formation (Aptian, Lower Cretaceous) of Neuquén, Patagonia, Argentina. *Cretaceous Research* **27**, 364–376, doi:10.1016/j.cretres.2005.07.004 (2006).
94 Otero, A. The appendicular skeleton of *Neuquensaurus*, a Late Cretaceous saltasaurine sauropod from Patagonia, Argentina. *Acta Palaeontologica Polonica* **55**, 399–426, doi:10.4202/app.2009.0099 (2010).
95 Harris, J. D. The appendicular skeleton of *Suuwassea emilieae* (Sauropoda: Flagellicaudata) from the Upper Jurassic Morrison Formation of Montana (USA). *Geobios* **40**, 501-522 (2007).
96 Sekiya, T. Re-examination of *Chuanjiesaurus ananensis* (Dinosauria: Sauropoda) from the Middle Jurassic Chuanjie Formation, Lufeng County, Yunnan Province, southwest China. *Memoir of the Fukui Prefectural Dinosaur Museum* **10**, 1–54 (2011).
97 Apesteguía, S. in *Thunder-lizards: The Sauropodomorph Dinosaurs* (eds Virginia Tidwell & Kenneth Carpenter) 321–345 (Indiana University Press, 2005).
98 Salgado, L. & Carvalho, I. d. S. *Uberabatitan ribeiroi*, a new titanosaur from the Marília Formation (Bauru Group, Upper Cretacous), Minas Gerais, Brazil. *Palaeontology* **51**, 881–901, doi:10.1111/j.1475-4983.2008.00781.x (2008).
99 Sereno, P. C. *et al.* Structural extremes in a Cretaceous dinosaur. *PLoS ONE* **2**, e1230 (2007).
100 Wilson, J. A. & Carrano, M. T. Titanosaurs and the origin of "wide-gauge" trackways: a biomechanical and systematic perspective on sauropod locomotion. *Paleobiology* **25**, 252–267 (1999).

101 Wilson, J. A. & Upchurch, P. Redescription and reassessment of *Euhelopus zdanskyi* (Dinosauria: Sauropoda) from the Early Cretaceous of China. *Journal of Systematic Palaeontology* **7**, 199–239, doi:10.1017/S1477201908002691 (2009).
102 Goloboff, P. A., Farris, J. S. & Nixon, K. C. TNT: a free program for phylogenetic analysis. *Cladistics* **24**, 774–786, doi:10.1111/j.1096-0031.2008.00217.x (2008).
103 Goloboff, P. A., Mattoni, C. I. & Quinteros, A. S. Continuous characters analyzed as such. *Cladistics* **22**, 589–601, doi:10.1111/j.1096-0031.2006.00122.x (2006).
104 Goloboff, P. A., Farris, J. S. & Nixon, K. C. *TNT: tree analysis using new technologies*, 2003).
105 Matzke, N. J. Probabilistic historical biogeography: new models for founder-event speciation, imperfect detection, and fossils allow improved accuracy and model-testing. *Frontiers of Biogeography* **5**, 242–248 (2013).
106 Matzke, N. J. Model selection in historical biogeography reveals that founder-event speciation is a crucial process in island clades. *Systematic Biology* **63**, 951–970, doi:10.1093/sysbio/syu056 (2014).
107 R: A language and environment for statistical computing (R Foundation for Statistical Computing, Vienna, Austria, 2013).
108 Bapst, D. W. paleotree: an R package for paleontological and phylogenetic analyses of evolution. *Methods in Ecology and Evolution* **3**, 803–807, doi:10.1111/j.2041-210X.2012.00223.x (2012).
109 Bapst, D. W. A stochastic rate-calibrated method for time-scaling phylogenies of fossil taxa. *Methods in Ecology and Evolution* **4**, 724–733, doi:10.1111/2041-210X.12081 (2013).
110 Bell, M. A. & Lloyd, G. T. strap: an R package for plotting phylogenies against stratigraphy and assessing their stratigraphic congruence. *Palaeontology* **58**, 379–389, doi:10.1111/pala.12142 (2015).
111 Cohen, K. M., Finney, S. C., Gibbard, P. L. & Fan, J.-X. The ICS International Chronostratigraphic Chart. *Episodes* **36**, 199–204 (2013; updated).
112 Brusatte, S. L., Benton, M. J., Ruta, M. & Lloyd, G. T. The first 50 Myr of dinosaur evolution: macroevolutionary pattern and morphological disparity. *Biology Letters* **4**, 733–736, doi:10.1098/rsbl.2008.0441 (2008).
113 Rogers, R. R., Hartman, J. H. & Krause, D. W. Stratigraphic analysis of Upper Cretaceous rocks in the Mahajanga Basin, Madagascar: implications for ancient and modern faunas. *Journal of Geology* **108**, 275–301, doi:10.1086/314403 (2000).
114 Rogers, R. R. *et al.* A new, richly fossiliferous member comprised of tidal deposits in the Upper Cretaceous Maevarano Formation, northwestern Madagascar. *Cretaceous Research* **44**, 12–29, doi:10.1016/j.cretres.2013.03.008 (2013).
115 Williamson, T. E. & Weil, A. Stratigraphic distribution of sauropod in the Upper Cretaceous of the San Juan Basin, New Mexico, with comments on North America's Cretaceous 'sauropod hiatus'. *Journal of Vertebrate Paleontology* **28**, 1218–1223, doi:10.1671/0272-4634-28.4.1218 (2008).
116 Khosla, A., Chin, K., Alimohammadin, H. & Dutta, D. Ostracods, plant tissues, and other inclusions in coprolites from the Late Cretaceous Lameta Formation at Pisdura, India: taphonomical and palaeoecological implications. *Palaeogeography, Palaeoclimatology, Palaeoecology* **418**, 90–100, doi:10.1016/j.palaeo.2014.11.003 (2015).
117 Jerzykiewicz, T. in *The Age of Dinosaurs in Russia and Mongolia* (eds Michael J Benton, Mikhail A Shishkin, David M Unwin, & Evgenii N Kurochkin) 279–296 (Cambridge University Press, 2000).

118 Quattrocchio, M. E., Volkheimer, W., Marquillas, R. A. & Salfity, J. A. Palynostratigraphy, palaeobiogeography and evolutionary significance of the late Senonian and early Palaeogene palynofloras of the Salta Group, northern Argentina. *Revista Española de Micropaleontología* **37**, 259–272 (2005).
119 González Riga, B. J., Lamanna, M. C., Ortiz David, L. D., Calvo, J. O. & Coria, J. P. A gigantic new dinosaur from Argentina and the evolution of the sauropod hind foot. *Scientific Reports* **6**, 19165, doi:10.1038/srep19165 (2016).
120 Leanza, H. A., Apesteguía, S., Novas, F. E. & de la Fuente, M. S. Cretaceous terrestrial beds from the Neuquén Basin (Argentina) and their tetrapod assemblages. *Cretaceous Research* **25**, 61–87, doi:10.1016/j.cretres.2003.10.005 (2004).
121 Leanza, H. A. & Hugo, C. A. in *VII International Symposium on Mesozoic Terrestrial Ecosystems: Asociacíon Paleontológica Argentina Publicación Especial No. 7* (ed Héctor A Leanza) 117–122 (Asociacion Paleontologica Argentina, 2001).
122 Mateus, O. *et al. Angolatitan adamastor*, a new sauropod dinosaur and the first record from Angola. *Anais da Academia Brasileira de Ciências* **83**, 221–233 (2011).
123 Antunes, M. T. *O Neocretácico e o Cenozóico do litoral de Angola*. (Junta de Investigações do Ultramar, 1964).
124 Martínez, R. D. & Novas, F. E. *Aniksosaurus darwini* gen. et sp. nov., a new coelurosaurian theropod from the early Late Cretaceous of central Patagonia, Argentina. *Revista del Museo Argentino de Ciencias Naturales* **8**, 243–259 (2006).
125 Ksepka, D. T. & Norell, M. A. The illusory evidence for Asian Brachiosauridae: new material of *Erketu ellisoni* and a phylogenetic reappraisal of basal Titanosauriformes. *American Museum Novitates* **3700**, 1–27 (2010).
126 Lü, J. *et al.* A new gigantic sauropod dinosaur with the deepest known body cavity from the Cretaceous of Asia. *Acta Geol. Sin.* **81**, 167–176, doi:10.1111/j.1755-6724.2007.tb00941.x (2007).
127 Lü, J.-c. *et al.* A new gigantic sauropod dinosaur from the Cretaceous of Ruyang, Henan, China. *Geological Bulletin of China* **28**, 1–10 (2009).
128 Lü, J. *et al.* A preliminary report on the new dinosaurian fauna from the Cretaceous of the Ruyang Basin, Henan Province of central China. *Journal of the Paleontological Society of Korea* **25**, 43–56 (2009).
129 Liang, X., Wen, S., Yang, D., Zhou, S. & Wu, S. Dinosaur eggs and dinosaur egg-bearing deposits (Upper Cretaceous) of Henan Province, China: occurrences, palaeoenvironments, taphonomy and preservation. *Progress in Natural Science* **19**, 1587–1601, doi:10.1016/j.pnsc.2009.06.012 (2009).
130 Zhang, X. *et al.* A new sauropod dinosaur from the Late Cretaceous Gaogou Formation of Nanyang, Henan Province. *Acta Geol. Sin.* **83**, 212–221, doi:10.1111/j.1755-6724.2009.00032.x (2009).
131 Yu, Y.-w., Jin, X.-s., Wu, X.-l. & Zhang, Z.-f. The epochs of dinosaurs and fossil eggs from Zhejiang Province. *Geology in China* **37**, 94–100 (2010).
132 Bryan, S. E. *et al.* Early–mid Cretaceous tectonic evolution of eastern Gondwana: from silicic LIP magmatism to continental rupture. *Episodes* **35**, 142–152 (2012).
133 Tucker, R. T., Roberts, E. M., Hu, Y., Kemp, A. I. S. & Salisbury, S. W. Detrital zircon age constraints for the Winton Formation, Queensland: contextualizing Australia's Late Cretaceous dinosaur faunas. *Gondwana Research* **24**, 767–779, doi:10.1016/j.gr.2012.12.009 (2013).
134 Suárez, M., Márquez, M., De La Cruz, R., Navarrete, C. & Fanning, M. Cenomanian-? early Turonian minimum age of the Chubut Group, Argentina: SHRIMP U-Pb geochronology. *Journal of South American Earth Sciences* **50**, 67–74, doi:10.1016/j.jsames.2013.10.008 (2014).

135 Carballido, J. L., Pol, D., Cerda, I. & Salgado, L. The osteology of *Chubutisaurus insignis* Del Corro, 1975 (Dinosauria: Neosauropoda) from the 'Middle' Cretaceous of Central Patagonia, Argentina. *Journal of Vertebrate Paleontology* **31**, 93–110, doi:10.1080/02724634.2011.539651 (2011).
136 Ratkevich, R. New Cretaceous brachiosaurid dinosaur, *Sonorasaurus thompsoni* gen. et sp. nov., from Arizona. *Journal of the Arizona-Nevada Academy of Science* **31**, 71–82 (1998).
137 Chure, D., Britt, B. B., Whitlock, J. A. & Wilson, J. A. First complete sauropod dinosaur skull from the Cretaceous of the Americas and the evolution of sauropod dentition. *Naturwissenschaften* **97**, 379–391, doi:10.1007/s00114-010-0650-6 (2010).
138 D'Emic, M. D. & Foreman, B. Z. The beginning of the sauropod hiatus in North America: insights from the Cloverly Formation of Wyoming. *Journal of Vertebrate Paleontology* **32**, 883–902, doi:10.1080/02724634.2012.671204 (2012).
139 Tang, F. *et al.* Biostratigraphy and palaeoenvironment of the dinosaur-bearing sediments in Lower Cretaceous of Mazongshan area, Gansu Province, China. *Cretaceous Research* **22**, 115–129, doi:10.1006/cres.2000.0242 (2001).
140 You, H., Tang, F. & Luo, Z. A new basal titanosaur (Dinosauria: Sauropoda) from the Early Cretaceous of China. *Acta Geol. Sin.* **77**, 424–429, doi:10.1111/j.1755-6724.2003.tb00123.x (2003).
141 Jacobs, L. L. & Winkler, D. A. in *Advances in Vertebrate Paleontology and Geochronology: National Science Museum Monograph 14* (eds Y Tomida, I J Flynn, & Louis L Jacobs) 253–280 (1998).
142 Adams, T. L. Small crocodyliform from the Lower Cretaceous (late Aptian) of central Texas and its systematic relationship to the evolution of Eusuchia. *Journal of Paleontology* **88**, 1031–1049, doi:10.1666/12-089 (2014).
143 Mannion, P. D. A reassessment of *Mongolosaurus haplodon* Gilmore, 1933, a titanosaurian sauropod dinosaur from the Early Cretaceous of Inner Mongolia, People's Republic of China. *Journal of Systematic Palaeontology* **9**, 355–378, doi:10.1080/14772019.2010.527379 (2011).
144 Kirkland, J. I. *et al.* in *Vertebrate Paleontology in Utah: Utah Geological Survey Miscellaneous Publication, 99-1* (ed David D Gillette) 201–217 (1997).
145 Taylor, M. P., Wedel, M. J. & Cifelli, R. L. A new sauropod dinosaur from the Lower Cretaceous Cedar Mountain Formation, Utah, USA. *Acta Palaeontologica Polonica* **56**, 75–98, doi:10.4202/app.2010.0073 (2011).
146 Jacobs, L. L., Winkler, D. A. & Murry, P. A. On the age and correlation of the Trinity mammal, Early Cretaceous of Texas, U.S.A. *Newsletters on Stratigraphy* **24**, 35–43 (1991).
147 Wedel, M. J., Cifelli, R. L. & Sanders, R. K. *Sauroposeidon proteles*, a new sauropod from the Early Cretaceous of Oklahoma. *Journal of Vertebrate Paleontology* **20**, 109–114, doi:10.1671/0272-4634(2000)020[0109:SPANSF]2.0.CO;2 (2000).
148 Taquet, P. *Géologie et paléontologie du gisement de Gadoufaoua (Aptien du Niger).* (Editions du Centre National de la Recherche Scientifique, 1976).
149 Sereno, P. C. *et al.* Cretaceous sauropods from the Sahara and the uneven rate of skeletal evolution among dinosaurs. *Science* **286**, 1342–1347 (1999).
150 You, H.-L. & Li, D.-Q. The first well-preserved Early Cretaceous brachiosaurid dinosaur in Asia. *Proceedings of the Royal Society B* **276**, 4077–4082 (2009).
151 Allain, R. *et al.* Un nouveau genre de dinosaure sauropode de la formation des Grès supérieurs (Aptien-Albien) du Laos. *Comptes Rendus de l'Académie des Sciences - Series IIA - Earth and Planetary Science* **329**, 609–616 (1999).

152 You, H.-l., Li, D.-q., Zhou, L.-q. & Ji, Q. *Daxiatitan binglingi*: a giant sauropod dinosaur from the Early Cretaceous of China. *Gansu Geology* **17**, 1–10 (2008).
153 Suarez, M. Chemostratigraphy of Early Cretaceous terrestrial strata in Gansu Province, China. *AAPG Search and Discovery Article* **90083** (2008).
154 Mo, J., Wang, W., Huang, Z., Huang, X. & Xu, X. A basal titanosauriform from the Early Cretaceous of Guangxi, China. *Acta Geol. Sin.* **80**, 486–489, doi:10.1111/j.1755-6724.2006.tb00267.x (2006).
155 Buffetaut, E., Suteethorn, V. & Tong, H. in *Papers from the 2005 Heyuan International Dinosaur Symposium* (eds Junchang Lü, Yoshitsugu Kobayashi, Dong Huang, & Yuong-Nam Lee) 19–37 (Geological Publishing House, 2006).
156 Mo, J., Xu, X. & Buffetaut, E. A new eusauropod dinosaur from the Lower Cretaceous of Guangxi Province, southern China. *Acta Geol. Sin.* **84**, 1328–1335, doi:10.1111/j.1755-6724.2010.00331.x (2010).
157 Gomani, E. M. Sauropod dinosaurs from the Early Cretaceous of Malawi, Africa. *Palaeontologia Electronica* **8**, 27A (2005).
158 Gorscak, E., O'Connor, P. M., Stevens, N. J. & Roberts, E. M. The basal titanosaurian *Rukwatitan bisepultus* (Dinosauria, Sauropoda) from the middle Cretaceous Galula Formation, Rukwa Rift Basin, southwestern Tanzania. *Journal of Vertebrate Paleontology* **34**, 1133–1154, doi:10.1080/02724634.2014.845568 (2014).
159 Moreno-Bedmar, J. A. *et al.* Biostratigraphic characterization by means of ammonoids of the lower Aptian Oceanic Anoxic Event (OAE 1a) in the eastern Iberian Chain (Maestrat Basin, eastern Spain). *Cretaceous Research* **30**, 864–872, doi:10.1016/j.cretres.2009.02.004 (2009).
160 Greenhalgh, B. W. & Britt, B. B. in *Central Utah - diverse geology of a dynamic landscape. Utah Geological Association Publication, 36* (eds G C Willis, M D Hylland, D L Clark, & T C Chidsey, Jr.) 81–100 (Utah Geological Association, 2007).
161 Racey, A. & Goodall, J. G. S. in *Late Palaeozoic and Mesozoic Ecosystems in SE Asia; Geological Society of London Special Publication, 315* (eds Eric Buffetaut, Gilles Cuny, Jean Le Loeuff, & Varavudh Suteethorn) 69–83 (Geological Society, 2009).
162 Kirkland, J. I. & Madsen, S. K. The Lower Cretaceous Cedar Mountain Formation, eastern Utah: the view up an always interesting learning curve. *Utah Geological Association Publication* **35**, 1–108 (2007).
163 Sha, J. Cretaceous stratigraphy of northeast China: non-marine and marine correlation. *Cretaceous Research* **28**, 146–170, doi:10.1016/j.cretres.2006.12.002 (2007).
164 Wang, X. *et al. Dongbeititan dongi*, the first sauropod dinosaur from the Lower Cretaceous Jehol Group of western Liaoning Province, China. *Acta Geol. Sin.* **81**, 911–916 (2007).
165 Azuma, Y. & Shibata, M. *Fukuititan nipponensis*, a new titanosauriform sauropod from the Early Cretaceous Tetori Group of Fukui Prefecture, Japan. *Acta Geol. Sin.* **84**, 454–462 (2010).
166 Horne, D. J. in *Ostracods in British Stratigraphy. Micropalaeontological Society Special Publication, 3* (eds John E Whittaker & M B Hart) 289–308 (Geological Society of London, 2009).
167 Sánchez-Hernández, B. *Galveosaurus herreroi*, a new sauropod dinosaur from Villar del Arzobispo Formation (Tithonian–Berriasian) of Spain. *Zootaxa* **1034**, 1–20 (2005).
168 Remes, K. A second Gondwanan diplodocid dinosaur from the Upper Jurassic Tendaguru Beds of Tanzania, East Africa. *Palaeontology* **50**, 653–667, doi:10.1111/j.1475-4983.2007.00652.x (2007).

169 Bussert, R., Heinrich, W.-D. & Aberhan, M. The Tendaguru Formation (Late Jurassic to Early Cretaceous, southern Tanzania): definition, palaeoenvironments, and sequence stratigraphy. *Fossil Record* **12**, 141–174, doi:10.1002/mmng.200900004 (2009).
170 Aberhan, M. *et al.* Palaeoecology and depositional environments of the Tendaguru Beds (Late Jurassic to Early Cretaceous, Tanzania). *Mitteilungen aus dem Museum für Naturkunde in Berlin, Geowissenschaftliche Reihe* **5**, 19–44, doi:10.1002/mmng.20020050103 (2002).
171 Kowallis, B. J. *et al.* The age of the Morrison Formation. *Modern Geology* **22**, 235–260 (1998).
172 Cúneo, R. *et al.* High-precision U-Pb geochronology and a new chronostratigraphy for the Cañadón Asfalto Basin, Chubut, central Patagonia: Implications for terrestrial faunal and floral evolution in Jurassic. *Gondwana Research* **24**, 1267–1275, doi:10.1016/j.gr.2013.01.010 (2013).
173 Carballido, J. L. & Sander, P. M. Postcranial axial skeleton of *Europasaurus holgeri*, (Dinosauria, Sauropoda) from the Upper Jurassic of Germany: implications for sauropod ontogeny and phylogenetic relationships of basal Macronaria. *Journal of Systematic Palaeontology* **12**, 335–387 (2014).
174 Sander, P. M., Mateus, O., Laven, T. & Knötschke, N. Bone histology indicates insular dwarfism in a new Late Jurassic sauropod dinosaur. *Nature* **441**, 739–741 (2006).
175 Lapparent, A. F. d. Les dinosauriens jurassiques de Damparis (Jura). *Mémoires de la Société Géologique de France (Nouvelle Série)* **47**, 5–20 (1943).
176 Buffetaut, E. Les restes de dinosaures de l'Oxfordien supérieur de Damparis (Jura): preuves d'emersion sur place. *Revue de Paléobiologie* **7**, 301–306 (1988).
177 Ouyang, H. & Ye, Y. *The first mamenchisaurian skeleton with complete skull, Mamenchisaurus youngi*. (Sichuan Science and Technology Press, 2002).
178 Young, C.-C. & Zhao, X.-J. *Mamenchisaurus hochuanensis* sp. nov. *Institute of Vertebrate Paleontology and Paleoanthropology, Monograph Series I* **8**, 1–30 (1972).
179 Tang, F., Jin, X., Kang, X. & Zhang, G. *Omeisaurus maoianus – a complete Sauropoda from Jingyan, Sichuan*. (China Ocean Press, 2001).
180 He, X., Li, K. & Cai, K. *The Middle Jurassic Dinosaur Fauna from Dashanpu, Zigong, Sichuan. Vol. IV. Sauropod dinosaurs (2) Omeisaurus tianfuensis*. Vol. IV (Sichuan Publishing House of Science and Technology, 1988).
181 Zhang, Y. *The Middle Jurassic Dinosaurian Fauna from Dashanpu, Zigong, Sichuan. Vol. III. Sauropod dinosaur (1) Shunosaurus*. Vol. III (Sichuan Publishing House of Science and Technology, 1988).
182 Chatterjee, S. & Zheng, Z. Cranial anatomy of *Shunosaurus*, a basal sauropod dinosaur from the Middle Jurassic of China. *Zoological Journal of the Linnean Society* **136**, 145–169, doi:10.1046/j.1096-3642.2002.00037.x (2002).
183 Jenny, J., Le Marrec, A. & Monbaron, M. Les Couches Rouges du Jurassique moyen du Haut Atlas central (Maroc): corrélations lithostratigraphiques, éléments de datation et cadre tectonosédimentaire. *Bulletin de la Société Géologique de France* **23 (Série 7)**, 627–639 (1981).
184 Monbaron, M., Russell, D. A. & Taquet, P. *Atlasaurus imelakei* n.g., n.s., a brachiosaurid-like sauropod from the Middle Jurassic of Morocco. *Comptes Rendus des Séances de l'Académie des Sciences, Paris, de la Terre et Planètes* **329**, 519–526 (1999).
185 Collignon, M. Découverte de dinosauriens à Tsinjorano, district d'Ambato-Boeni. *Bulletin de l'Academie Malgache* **31**, 59–61 (1953).

186 Buffetaut, E. A new sauropod dinosaur with prosauropod-like teeth from the Middle Jurassic of Madagascar. *Bulletin de la Société Géologique de France* **176**, 467–473 (2005).

187 Lawver, L. A., Dalziel, I. W. D. & Gahagan, L. M. in *Origins and Evolution of Cenozoic South American Mammals* (eds Alfred I Rosenberger & Margarita Fernández Tejedor) (Springer, in press).

188 Upchurch, P., Hunn, C. A. & Norman, D. B. An analysis of dinosaurian biogeography: evidence for the existence of vicariance and dispersal patterns caused by geological events. *Proceedings of the Royal Society B* **269**, 613–621, doi:10.1098/rspb.2001.1921 (2002).

189 Ezcurra, M. D. & Agnolin, F. L. A new global palaeobiogeographical model for the late Mesozoic and early Tertiary. *Systematic Biology* **61**, 553–566, doi:10.1093/sysbio/syr115 (2012).

190 Csiki-Sava, Z., Buffetaut, E., Ősi, A., Pereda-Suberbiola, X. & Brusatte, S. L. Island life in the Cretaceous – faunal composition, biogeography, evolution, and extinction of land-living vertebrates on the Late Cretaceous European archipelago. *ZooKeys* **469**, 1–161, doi:10.3897/zookeys.469.8439 (2015).

191 Mannion, P. D. *et al.* A temperate palaeodiversity peak in Mesozoic dinosaurs and evidence for Late Cretaceous geographical partitioning. *Global Ecology and Biogeography* **21**, 898–908, doi:10.1111/j.1466-8238.2011.00735.x (2012).

192 Benson, R. B. J. *et al.* Cretaceous tetrapod fossil record sampling and faunal turnover: implications for biogeography and the rise of modern clades. *Palaeogeography, Palaeoclimatology, Palaeoecology* **372**, 88–107, doi:10.1016/j.palaeo.2012.10.028 (2013).

193 Hay, W. W. Evolving ideas about the Cretaceous climate and ocean circulation. *Cretaceous Research* **29**, 725–753, doi:10.1016/j.cretres.2008.05.025 (2008).

194 Mannion, P. D. & Upchurch, P. A quantitative analysis of environmental associations in sauropod dinosaurs. *Paleobiology* **36**, 253–282, doi:10.1666/08085.1 (2010).

195 Averianov, A. O., Voronkevich, A. V., Maschenk, E. N., Leshchinskiy, S. V. & Fayngertz, A. V. A sauropod foot from the Early Cretaceous of Western Siberia, Russia. *Acta Palaeontologica Polonica* **47**, 117–124 (2002).

196 Sanmartin, I. & Ronquist, F. Southern Hemisphere biogeography inferred by event-based models: plant versus animal patterns. *Systematic Biology* **53**, 216–243 (2004).

197 Russell, D. A. The role of Central Asia in dinosaurian biogeography. *Canadian Journal of Earth Sciences* **30**, 2002–2012 (1993).

198 Weishampel, D. B. & Jianu, C.-M. The importance of phylogeny in paleobiogeographic analyses with examples from North-American hadrosaurids and European titanosaurids. *Sargetia* **17**, 261–278 (1997).

199 Smith, A. G., Smith, D. G. & Funnell, B. M. *Atlas of Mesozoic and Cenozoic Coastlines*. (Cambridge University Press, 2004).

200 Tyson, R. V. & Funnell, B. M. European Cretaceous shorelines, stage by stage. *Palaeogeography, Palaeoclimatology, Palaeoecology* **59**, 69–91, doi:10.1016/0031-0182(87)90075-7 (1987).

201 Bardet, N. *et al.* Mesozoic marine reptile palaeobiogeography in response to drifting plates. *Gondwana Research* **26**, 869–887, doi:10.1016/j.gr.2014.05.005 (2014).

202 Scotese, C. R. in *Frontiers of Biogeography* (eds Mark V Lomolino & Lawrence R Heaney) 9–26 (Sinauer, 2004).

203 Blakey, R. C. in *Resolving the Late Paleozoic Ice Age in Time and Space* Vol. 441 *Geological Society of America Special Paper* (eds Christopher R Fielding, Tracy D Frank, & John L Isbell) 1–28 (Geological Society of America, 2008).

204 Wilf, P., Cúneo, N. R., Escapa, I. H., Pol, D. & Woodburne, M. O. Splendid and seldom isolated: the paleobiogeography of Patagonia. *Annual Review of Earth and Planetary Sciences* **41**, 561–603, doi:10.1146/annurev-earth-050212-124217 (2013).

205 Golonka, J. *et al.* in *The Continental Jurassic. Museum of Northern Arizona Bulletin 60* (ed Michael Morales) 1–5 (Museum of Northern Arizona, 1996).

206 Milner, A. R. & Norman, D. B. in *Third Symposium on Mesozoic Terrestrial Ecosystems, Short Papers* (eds Wolf-Ernst Reif & Frank Westphal) 145–150 (University Press, 1984).

207 Le Loeuff, J. in *Encyclopedia of Dinosaurs* (eds Philip John Currie & Kevin Padian) 51–56 (Academic Press, 1997).

208 Enkin, R. J., Yang, Z., Chen, Y. & Courtillot, V. Paleomagnetic constraints on the geodynamic history of the major blocks of China from the Permian to the present. *Journal of Geophysical Research: Solid Earth* **97**, 13953–13989, doi:10.1029/92JB00648 (1992).

209 Scotese, C. R. *Atlas of Earth History*. (PALEOMAP Project, 2001).

210 Cogné, J.-P., Kravchinsky, V. A., Halim, N. & Hankard, F. Late Jurassic–Early Cretaceous closure of the Mongol-Okhotsk Ocean demonstrated by new Mesozoic palaeomagnetic results from the Trans-Baïkal area (SE Siberia). *Geophysical Journal International* **163**, 813–832, doi:10.1111/j.1365-246X.2005.02782.x (2005).

211 Tomurtogoo, O., Windley, B. F., Kröner, A., Badarch, G. & Liu, D. Zircon age and occurrence of the Adaatsag ophiolite and Muron shear zone, central Mongolia: constraints on the evolution of the Mongol–Okhotsk ocean, suture and orogen. *Journal of the Geological Society* **162**, 125–134, doi:10.1144/0016-764903-146 (2005).

212 Seton, M. *et al.* Global continental and ocean basin reconstructions since 200 Ma. *Earth-Science Reviews* **113**, 212–270, doi:10.1016/j.earscirev.2012.03.002 (2012).

213 Yang, Y.-T., Guo, Z.-X., Song, C.-C., Li, X.-B. & He, S. A short-lived but significant Mongol–Okhotsk collisional orogeny in latest Jurassic–earliest Cretaceous. *Gondwana Research*, doi:10.1016/j.gr.2014.09.010 (in press).

214 Labails, C., Olivet, J.-L., Aslanian, D. & Roest, W. R. An alternative early opening scenario for the Central Atlantic Ocean. *Earth and Planetary Science Letters* **297**, 355–368 (2010).

215 Gaina, C. *et al.* The African Plate: A history of oceanic crust accretion and subduction since the Jurassic. *Tectonophysics* **604**, 4–25, doi:10.1016/j.tecto.2013.05.037 (2013).

216 Escaso, F. *et al.* New evidence of shared dinosaur across Upper Jurassic proto-North Atlantic: *Stegosaurus* from Portugal. *Naturwissenschaften* **94**, 367–374, doi:10.1007/s00114-006-0209-8 (2007).

217 Dantas, P. *et al.* O dinossáurio carnívoro *Allosaurus fragilis* no Jurássico Superior Português. *Al-Madam* **8**, 23–28 (1999).

218 Pérez-Moreno, B. P. *et al.* On the presence of *Allosaurus fragilis* (Theropoda: Carnosauria) in the Upper Jurassic of Portugal: first evidence of an intercontinental dinosaur species. *Journal of the Geological Society of London* **156**, 449–452, doi:10.1144/gsjgs.156.3.0449 (1999).

219 Rauhut, O. W. M. & Fechner, R. Early development of the facial region in a non-avian theropod dinosaur. *Proceedings of the Royal Society B* **272**, 1179–1183, doi:10.1098/rspb.2005.3071 (2005).

220 Malafaia, E., Ortega, F., Escaso, F. & Silva, B. New evidence of Ceratosaurus (Dinosauria: Theropoda) from the Late Jurassic of the Lusitanian Basin, Portugal. *Historical Biology*, doi:10.1080/08912963.2014.915820 (in press).

221 Benson, R. B. J. New information on *Stokesosaurus*, a tyrannosauroid (Dinosauria: Theropoda) from North America and the United Kingdom. *Journal of Vertebrate*

Paleontology **28**, 732–750, doi:10.1671/0272-4634(2008)28[732:NIOSAT]2.0.CO;2 (2008).

222 Brusatte, S. L. & Benson, R. B. J. The systematics of Late Jurassic tyrannosauroids (Dinosauria: Theropoda) from Europe and North America. *Acta Palaeontologica Polonica* **58**, 47–54, doi:10.4202/app.2011.0141 (2013).

223 Hendrickx, C. & Mateus, O. *Torvosaurus gurneyi* n. sp., the largest terrestrial predator from Europe, and a proposed terminology of the maxilla anatomy in nonavian theropods. *PLoS ONE* **9**, e88905, doi:10.1371/journal.pone.0088905 (2014).

224 Gaina, C., Müller, R. D., Brown, B., Ishihara, T. & Ivanov, S. Breakup and early seafloor spreading between India and Antarctica. *Geophysical Journal International* **170**, 151–169, doi:10.1111/j.1365-246X.2007.03450.x (2007).

225 Jokat, W., Boebel, T., König, M. & Meyer, U. Timing and geometry of early Gondwana breakup. *Journal of Geophysical Research: Solid Earth* **108**, 2428, doi:10.1029/2002JB001802 (2003).

226 König, M. & Jokat, W. Advanced insights into magmatism and volcanism of the Mozambique Ridge and Mozambique Basin in the view of new potential field data. *Geophysical Journal International* **180**, 158–180, doi:10.1111/j.1365-246X.2009.04433.x (2010).

227 Roeser, H. A., Fritsch, J. & Hinz, K. in *Weddell Sea Tectonics and Gondwana Break-up. Geology Society Special Publication 108* (eds Bryan C Storey, Edward C King, & Roy A Livermore) 243–264 (Geological Society, 1996).

228 Ali, J. R. & Krause, D. W. Late Cretaceous bioconnections between Indo-Madagascar and Antarctica: refutation of the Gunnerus Ridge causeway hypothesis. *Journal of Biogeography* **38**, 1855–1872, doi:10.1111/j.1365-2699.2011.02546.x (2011).

229 Upchurch, P. Gondwana break-up: legacies of a lost world? *Trends in Ecology & Evolution* **23**, 229–236, doi:10.1016/j.tree.2007.11.006 (2008).

230 Ziegler, A. M., Scotese, C. R. & Barrett, S. F. in *Tidal Friction and the Earth's Rotation II* (eds Peter Brosche & Jürgen Sündermann) 240–252 (Springer-Verlag, 1982).

231 Barron, E. J. Cretaceous plate tectonic reconstructions. *Palaeogeography, Palaeoclimatology, Palaeoecology* **59**, 3–29, doi:10.1016/0031-0182(87)90071-X (1987).

232 Scotese, C. R., Gahagan, L. M. & Larson, R. L. Plate tectonic reconstructions of the Cretaceous and Cenozoic ocean basins. *Tectonophysics* **155**, 27–48, doi:10.1016/0040-1951(88)90259-4 (1988).

233 Scotese, C. R. Jurassic and Cretaceous plate tectonic reconstructions. *Palaeogeography, Palaeoclimatology, Palaeoecology* **87**, 493–501, doi:10.1016/0031-0182(91)90145-H (1991).

234 Pitman, W. C., III, Cande, S. C., LaBrecque, J. L. & Pindell, J. in *Biological Relationships between Africa and South America* (ed Peter Goldblatt) 15–34 (Yale University Press, 1993).

235 Smith, A. G., Smith, D. G. & Funnell, B. M. *Atlas of Mesozoic and Cenozoic Coastlines*. (Cambridge University Press, 1994).

236 Eagles, G. & König, M. A model of plate kinematics in Gondwana breakup. *Geophysical Journal International* **173**, 703–717, doi:10.1111/j.1365-246X.2008.03753.x (2008).

237 Hay, W. W. *et al.* in *Evolution of Cretaceous Ocean-Climate System. Geological Society of America, Special Paper, 332* (eds Enriqueta Berrera & Claudia C Johnson) 1–47 (1999).

238 Schettino, A. & Scotese, C. R. Apparent polar wander paths for the major continents (200 Ma to the present day): a palaeomagnetic reference frame for global plate tectonic reconstructions. *Geophysical Journal International* **163**, 727–759, doi:10.1111/j.1365-246X.2005.02638.x (2005).
239 Vérard, C., Flores, K. & Stampfli, G. Geodynamic reconstructions of the South America–Antarctica plate system. *Journal of Geodynamics* **53**, 43–60, doi:10.1016/j.jog.2011.07.007 (2012).
240 Baraboshkin, E. Y., Alekseev, A. S. & Kopaevich, L. F. Cretaceous palaeogeography of the North-Eastern Peri-Tethys. *Palaeogeography, Palaeoclimatology, Palaeoecology* **196**, 177–208, doi:10.1016/S0031-0182(03)00318-3 (2003).
241 Gheerbrant, E. & Rage, J.-C. Paleobiogeography of Africa: how distinct from Gondwana and Laurasia? *Palaeogeography, Palaeoclimatology, Palaeoecology* **241**, 224–246 (2006).
242 Plafker, G. & Berg, H. C. 1068 (Geological Society of America, 1994).
243 Zanno, L. E. & Makovicky, P. J. On the earliest record of Cretaceous tyrannosauroids in western North America: implications for an Early Cretaceous Laurasian interchange event. *Historical Biology* **23**, 317–325, doi:10.1080/08912963.2010.543952 (2011).
244 Sereno, P. C., Wilson, J. A. & Conrad, J. L. New dinosaurs link southern landmasses in the mid-Cretaceous. *Proceedings of the Royal Society B* **271**, 1325–1330, doi:10.1098/rspb.2004.2692 (2004).
245 Stica, J. M., Zalán, P. V. & Ferrari, A. L. The evolution of rifting on the volcanic margin of the Pelotas Basin and the contextualization of the Paraná–Etendeka LIP in the separation of Gondwana in the South Atlantic. *Marine and Petroleum Geology* **50**, 1–21 (2014).
246 Heine, C., Zoethout, J. & Müller, R. D. Kinematics of the South Atlantic rift. *Solid Earth* **4**, 215–253, doi:10.5194/se-4-215-2013 (2013).
247 Akhmetiev, M. A. & Beniamovski, V. N. Paleogene floral assemblages around epicontinental seas and straits in Northern Central Eurasia: proxies for climatic and paleogeographic evolution. *Geologica Acta* **7**, 297–309, doi:10.1344/105.000000278 (2009).
248 Akhmetiev, M. A. *et al.* The Paleogene history of the Western Siberian seaway – a connection of the Peri-Tethys to the Arctic ocean. *Austrian Journal of Earth Sciences* **105**, 50–67 (2012).
249 Brikiatis, L. The De Geer, Thulean and Beringia routes: key concepts for understanding early Cenozoic biogeography. *Journal of Biogeography* **41**, 1036–1054, doi:10.1111/jbi.12310 (2014).
250 Simpson, G. G. History of the fauna of Latin America. *American Scientist* **38**, 361–389 (1950).
251 Rage, J.-C. Les continents péri-atlantiques au Crétacé supérieur: migrations des faunes continentales et problèmes paléogéographiques. *Cretaceous Research* **2**, 65–84, doi:10.1016/S0195-6671(81)80005-5 (1981).
252 Bonaparte, J. F. Nuevas pruebas de la conexión físca entre Sudamérica y Norteamérica en el Cretácico Tardío (Campaniano). *Actas III Congresso, Argentina Paleontologia*, 141–149 (1984).
253 Lucas, S. G. & Hunt, A. P. in *Paleobiology of the Dinosaurs Geological Society of America Special Paper* (ed James Orville Farlow) 75–86 (Geological Society of America, 1989).
254 Gayet, M., Rage, J.-C., Sempere, T. & Gagnier, P.-Y. Modalités des échanges de vertébrés continentaux entre l'Amérique du Nord et l'Amérique du Sud au Crétacé

supérieur et au Paléocène. *Bulletin de la Société Géologique de France* **163**, 781–791 (1992).

255 Lucas, S. G. & Sullivan, R. M. in *Dinosaurs of New Mexico New Mexico Museum of Natural History and Science Bulletin* (eds Spencer G Lucas & Andrew B Heckert) 147–156 (New Mexico Museum of Natural History and Science, 2000).

256 Prieto-Márquez, A. Skeletal morphology of *Kritosaurus navajovius* (Dinosauria: Hadrosauridae) from the Late Cretaceous of the North American south-west, with an evaluation of the phylogenetic systematics and biogeography of Kritosaurini. *Journal of Systematic Palaeontology* **12**, 133–175 (2014).

257 Schmidt-Effing, R. Alter und genese des Nicoya-Komplexes, einer ozeanischen Paláokruste (Oberjura bis Eozàn) im südlichen Zentralamerika. *Geologische Rundschau* **68**, 457–494 (1979).

258 Hedges, S. B. Paleogeography of the Antilles and the origin of West Indian terrestrial vertebrates. *Annals of the Missouri Botanical Garden* **93**, 231–244 (2006).

259 Iturralde-Vinent, M. A. & MacPhee, R. D. E. Paleogeography of the Caribbean region: implications for Cenozoic biogeography. *Bulletin of the American Museum of Natural History* **238**, 1–95 (1999).

260 Pough, F. H. *et al. Herpetology (Third Edition).* (Benjamin Cummings, 2004).

261 James, K. H. in *The Origin and Evolution of the Caribbean Plate. Geological Society of London Special Publication 328* (eds Keith H James, Maria Antonieta Lorente, & James L Pindell) 127–138 (Geological Society, 2009).

262 James, K. H. in *The Origin and Evolution of the Caribbean Plate. Geological Society of London Special Publication 328* (eds Keith H James, Maria Antonieta Lorente, & James L Pindell) 77–125 (Geological Society, 2009).

263 Loewen, M. A., Irmis, R. B., Sertich, J. J. W., Currie, P. J. & Sampson, S. D. Tyrant dinosaur evolution tracks the rise and fall of Late Cretaceous oceans. *PLoS ONE* **8**, e79420, doi:10.1371/journal.pone.0079420 (2013).

264 Briggs, J. C. The genesis of Central America – biology versus geophysics. *Global Ecology and Biogeography Letters* **4**, 169–172, doi:10.2307/2997648 (1994).

265 Briggs, J. C. *Global Biogeography*. (Elsevier, 1995).

266 Pindell, J. & Kennan, L. in *Petroleum Systems of Deep-Water Basins: Global and Gulf of Mexico Experience. Transactions of the GCSSEPM 21st Annual Research Conference* 193–220 (GCSSEPM, 2001).

267 Betts, P. G., Giles, D., Lister, G. S. & Frick, L. R. Evolution of the Australian lithosphere. *Australian Journal of Earth Sciences* **49**, 661–695, doi:10.1046/j.1440-0952.2002.00948.x (2002).

268 Veevers, J. J., Powell, C. M. & Roots, S. R. Review of seafloor spreading around Australia. I. synthesis of the patterns of spreading. *Australian Journal of Earth Sciences* **38**, 373–389 (1991).

269 Veevers, J. J. Updated Gondwana (Permian–Cretaceous) earth history of Australia. *Gondwana Research* **9**, 231–260, doi:10.1016/j.gr.2005.11.005 (2006).

270 Ree, R. H. Detecting the historical signature of key innovations using stochastic models of character evolution and cladogenesis. *Evolution* **59**, 257–265, doi:10.1111/j.0014-3820.2005.tb00986.x (2005).

271 Ree, R. H. & Smith, S. A. Maximum likelihood inference of geographic range evolution by dispersal, local extinction, and cladogenesis. *Systematic Biology* **57**, 4–14, doi:10.1080/10635150701883881 (2008).

272 Ronquist, F. Dispersal-Vicariance Analysis: A new approach to the quantification of historical biogeography. *Systematic Biology* **46**, 195–203, doi:10.1093/sysbio/46.1.195 (1997).

273 Landis, M. J., Matzke, N. J., Moore, B. R. & Huelsenbeck, J. P. Bayesian analysis of biogeography when the number of areas is large. *Systematic Biology* **62**, 789–804, doi:10.1093/sysbio/syt040 (2013).
274 Mannion, P. D. & Upchurch, P. A re-evaluation of the 'mid-Cretaceous sauropod hiatus' and the impact of uneven sampling of the fossil record on patterns of regional dinosaur extinction. *Palaeogeography, Palaeoclimatology, Palaeoecology* **299**, 529–540, doi:10.1016/j.palaeo.2010.12.003 (2011).
275 Sereno, P. C. *et al.* Predatory dinosaurs from the Sahara and Late Cretaceous faunal differentiation. *Science* **272**, 986–991, doi:10.1126/science.272.5264.986 (1996).
276 Rich, T. H. & Vickers-Rich, P. Protoceratopsian? ulnae from Australia. *Records of the Queen Victoria Museum* **113**, 1–12 (2003).

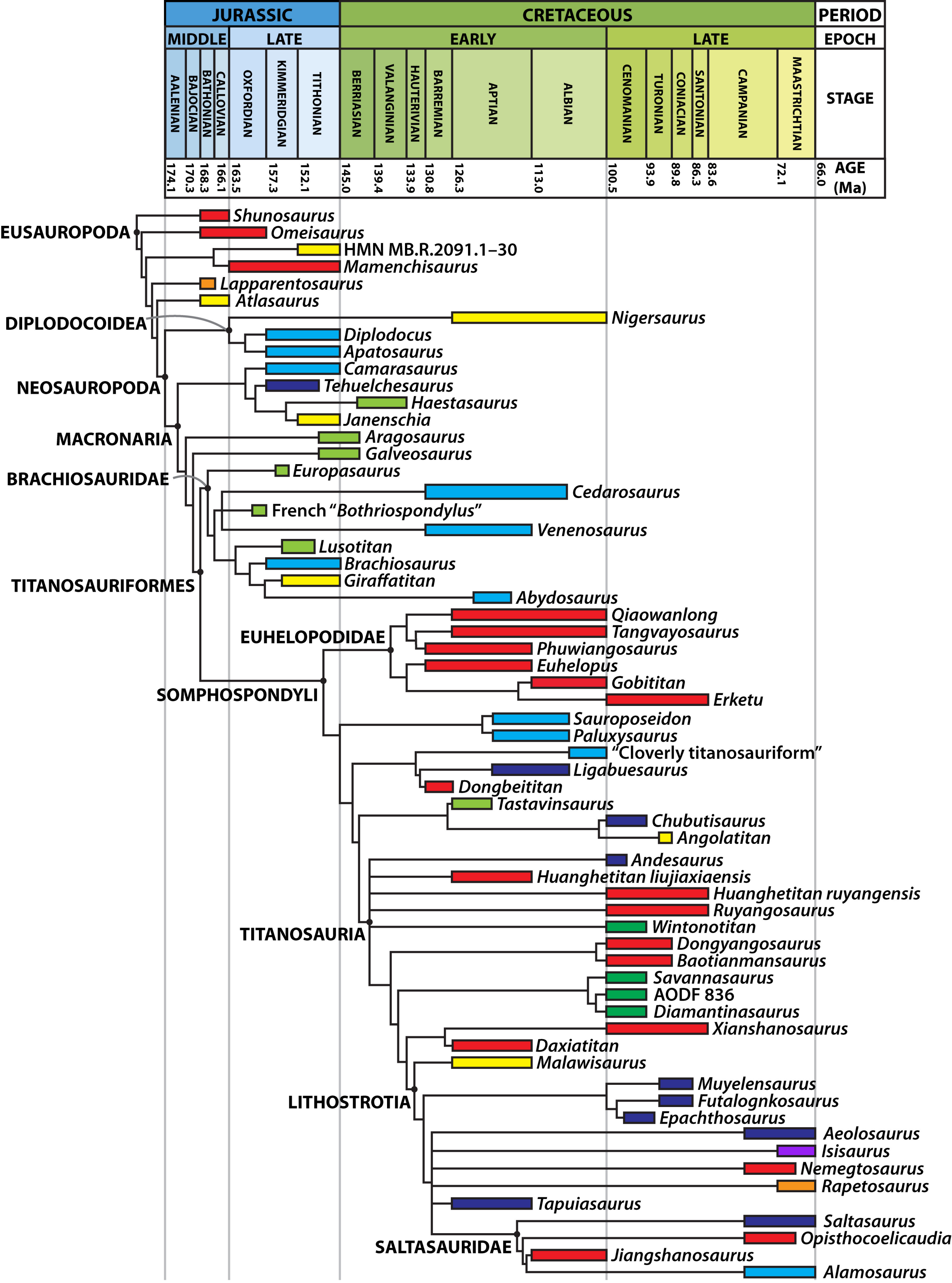

JURASSIC
CRETACEOUS
PERIOD
MIDDLE
LATE
EARLY
LATE
EPOCH
AALENIAN
BAJOCIAN
BATHONIAN
CALLOVIAN
OXFORDIAN
KIMMERIDGIAN
TITHONIAN
BERRIASIAN
VALANGINIAN
HAUTERIVIAN
BARREMIAN
APTIAN
ALBIAN
CENOMANIAN
TURONIAN
CONIACIAN
SANTONIAN
CAMPANIAN
MAASTRICHTIAN
STAGE
174.1
170.3
168.3
166.1
163.5
157.3
152.1
145.0
139.4
133.9
130.8
126.3
113.0
100.5
93.9
89.8
86.3
83.6
72.1
66.0
AGE (Ma)
EUSAUROPODA
DIPLODOCOIDEA
NEOSAUROPODA
MACRONARIA
BRACHIOSAURIDAE
TITANOSAURIFORMES
EUHELOPODIDAE
SOMPHOSPONDYLI
TITANOSAURIA
LITHOSTROTIA
SALTASAURIDAE
Shunosaurus
Omeisaurus
HMN MB.R.2091.1–30
Mamenchisaurus
Lapparentosaurus
Atlasaurus
Nigersaurus
Diplodocus
Apatosaurus
Camarasaurus
Tehuelchesaurus
Haestasaurus
Janenschia
Aragosaurus
Galveosaurus
Europasaurus
Cedarosaurus
French “Bothriospondylus”
Venenosaurus
Lusotitan
Brachiosaurus
Giraffatitan
Abydosaurus
Qiaowanlong
Tangvayosaurus
Phuwiangosaurus
Euhelopus
Gobititan
Erketu
Sauroposeidon
Paluxysaurus
“Cloverly titanosauriform”
Ligabuesaurus
Dongbeititan
Tastavinsaurus
Chubutisaurus
Angolatitan
Andesaurus
Huanghetitan liujiaxiaensis
Huanghetitan ruyangensis
Ruyangosaurus
Wintonotitan
Dongyangosaurus
Baotianmansaurus
Savannasaurus
AODF 836
Diamantinasaurus
Xianshanosaurus
Daxiatitan
Malawisaurus
Muyelensaurus
Futalognkosaurus
Epachthosaurus
Aeolosaurus
Isisaurus
Nemegtosaurus
Rapetosaurus
Tapuiasaurus
Saltasaurus
Opisthocoelicaudia
Jiangshanosaurus
Alamosaurus

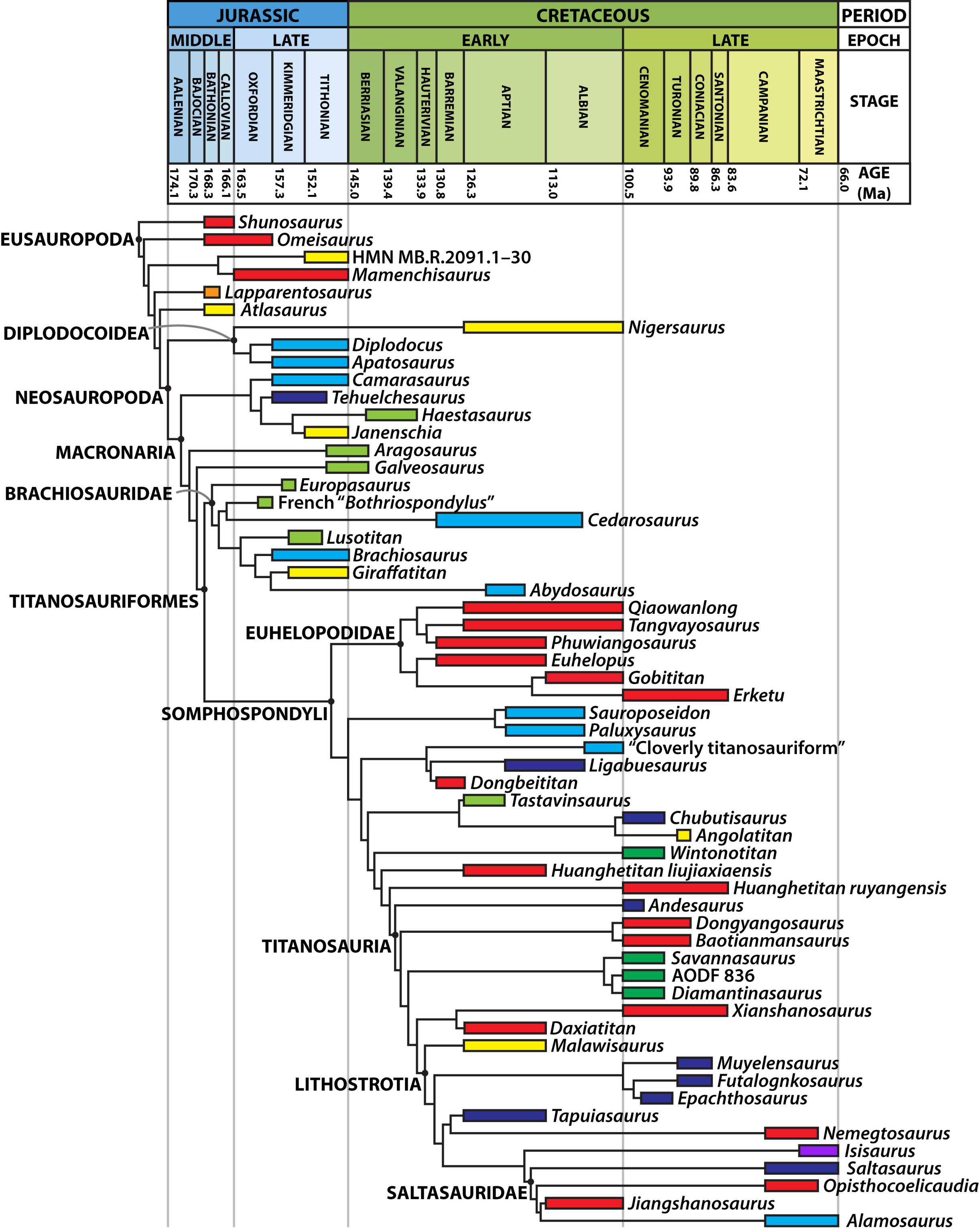
JURASSIC
CRETACEOUS
PERIOD
MIDDLE
LATE
EARLY
LATE
EPOCH
AALENIAN
BAJOCIAN
BATHONIAN
CALLOVIAN
OXFORDIAN
KIMMERIDGIAN
TITHONIAN
BERRIASIAN
VALANGINIAN
HAUTERIVIAN
BARREMIAN
APTIAN
ALBIAN
CENOMANIAN
TURONIAN
CONIACIAN
SANTONIAN
CAMPANIAN
MAASTRICHTIAN
STAGE
174.1
170.3
168.3
166.1
163.5
157.3
152.1
145.0
139.4
133.9
130.8
126.3
113.0
100.5
93.9
89.8
86.3
83.6
72.1
66.0
AGE (Ma)
EUSAUROPODA
DIPLODOCOIDEA
NEOSAUROPODA
MACRONARIA
BRACHIOSAURIDAE
TITANOSAURIFORMES
EUHELOPODIDAE
SOMPHOSPONDYLI
TITANOSAURIA
LITHOSTROTIA
SALTASAURIDAE
Shunosaurus
Omeisaurus
HMN MB.R.2091.1–30
Mamenchisaurus
Lapparentosaurus
Atlasaurus
Nigersaurus
Diplodocus
Apatosaurus
Camarasaurus
Tehuelchesaurus
Haestasaurus
Janenschia
Aragosaurus
Galveosaurus
Europasaurus
French "Bothriospondylus"
Cedarosaurus
Lusotitan
Brachiosaurus
Giraffatitan
Abydosaurus
Qiaowanlong
Tangvayosaurus
Phuwiangosaurus
Euhelopus
Gobititan
Erketu
Sauroposeidon
Paluxysaurus
"Cloverly titanosauriform"
Ligabuesaurus
Dongbeititan
Tastavinsaurus
Chubutisaurus
Angolatitan
Wintonotitan
Huanghetitan liujiaxiaensis
Huanghetitan ruyangensis
Andesaurus
Dongyangosaurus
Baotianmansaurus
Savannasaurus
AODF 836
Diamantinasaurus
Xianshanosaurus
Daxiatitan
Malawisaurus
Muyelensaurus
Futalognkosaurus
Epachthosaurus
Tapuiasaurus
Nemegtosaurus
Isisaurus
Saltasaurus
Opisthocoelicaudia
Jiangshanosaurus
Alamosaurus

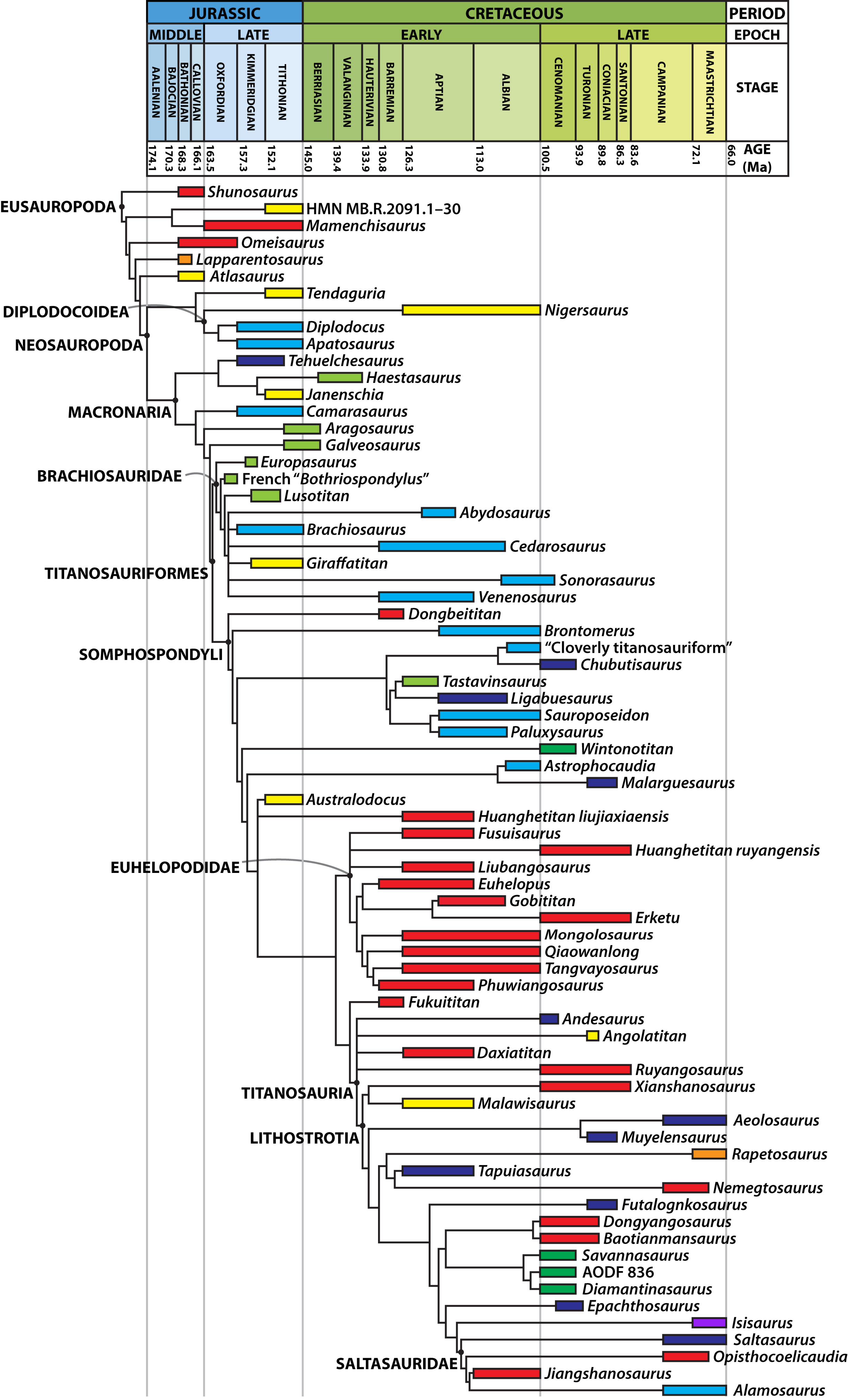

JURASSIC
CRETACEOUS
PERIOD
MIDDLE
LATE
EARLY
LATE
EPOCH
AALENIAN
BAJOCIAN
BATHONIAN
CALLOVIAN
OXFORDIAN
KIMMERIDGIAN
TITHONIAN
BERRIASIAN
VALANGINIAN
HAUTERIVIAN
BARREMIAN
APTIAN
ALBIAN
CENOMANIAN
TURONIAN
CONIACIAN
SANTONIAN
CAMPANIAN
MAASTRICHTIAN
STAGE
174.1
170.3
168.3
166.1
163.5
157.3
152.1
145.0
139.4
133.9
130.8
126.3
113.0
100.5
93.9
89.8
86.3
83.6
72.1
66.0
AGE (Ma)
EUSAUROPODA
DIPLODOCOIDEA
NEOSAUROPODA
MACRONARIA
BRACHIOSAURIDAE
TITANOSAURIFORMES
SOMPHOSPONDYLI
EUHELOPODIDAE
TITANOSAURIA
LITHOSTROTIA
SALTASAURIDAE
Shunosaurus
HMN MB.R.2091.1–30
Mamenchisaurus
Omeisaurus
Lapparentosaurus
Atlasaurus
Tendaguria
Nigersaurus
Diplodocus
Apatosaurus
Tehuelchesaurus
Haestasaurus
Janenschia
Camarasaurus
Aragosaurus
Galveosaurus
Europasaurus
French "Bothriospondylus"
Lusotitan
Abydosaurus
Brachiosaurus
Cedarosaurus
Giraffatitan
Sonorasaurus
Venenosaurus
Dongbeititan
Brontomerus
"Cloverly titanosauriform"
Chubutisaurus
Tastavinsaurus
Ligabuesaurus
Sauroposeidon
Paluxysaurus
Wintonotitan
Astrophocaudia
Malarguesaurus
Australodocus
Huanghetitan liujiaxiaensis
Fusuisaurus
Huanghetitan ruyangensis
Liubangosaurus
Euhelopus
Gobititan
Erketu
Mongolosaurus
Qiaowanlong
Tangvayosaurus
Phuwiangosaurus
Fukuititan
Andesaurus
Angolatitan
Daxiatitan
Ruyangosaurus
Xianshanosaurus
Malawisaurus
Aeolosaurus
Muyelensaurus
Rapetosaurus
Tapuiasaurus
Nemegtosaurus
Futalognkosaurus
Dongyangosaurus
Baotianmansaurus
Savannasaurus
AODF 836
Diamantinasaurus
Epachthosaurus
Isisaurus
Saltasaurus
Opisthocoelicaudia
Jiangshanosaurus
Alamosaurus

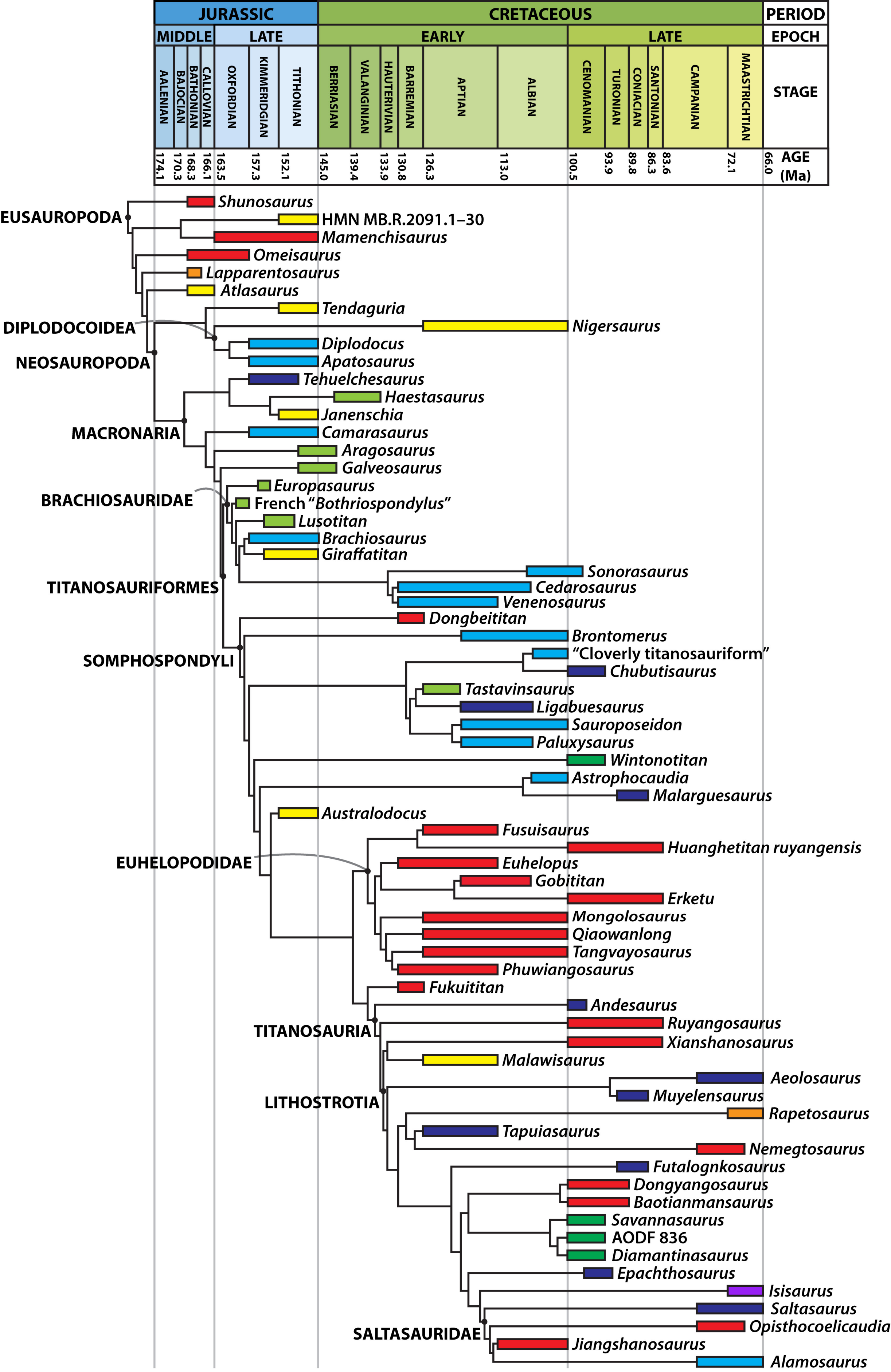

JURASSIC
CRETACEOUS
PERIOD
MIDDLE
LATE
EARLY
LATE
EPOCH
AALENIAN
BAJOCIAN
BATHONIAN
CALLOVIAN
OXFORDIAN
KIMMERIDGIAN
TITHONIAN
BERRIASIAN
VALANGINIAN
HAUTERIVIAN
BARREMIAN
APTIAN
ALBIAN
CENOMANIAN
TURONIAN
CONIACIAN
SANTONIAN
CAMPANIAN
MAASTRICHTIAN
STAGE
174.1
170.3
168.3
166.1
163.5
157.3
152.1
145.0
139.4
133.9
130.8
126.3
113.0
100.5
93.9
89.8
86.3
83.6
72.1
66.0
AGE (Ma)
EUSAUROPODA
DIPLODOCOIDEA
NEOSAUROPODA
MACRONARIA
BRACHIOSAURIDAE
TITANOSAURIFORMES
SOMPHOSPONDYLI
EUHELOPODIDAE
TITANOSAURIA
LITHOSTROTIA
SALTASAURIDAE
Shunosaurus
HMN MB.R.2091.1–30
Mamenchisaurus
Omeisaurus
Lapparentosaurus
Atlasaurus
Tendaguria
Nigersaurus
Diplodocus
Apatosaurus
Tehuelchesaurus
Haestasaurus
Janenschia
Camarasaurus
Aragosaurus
Galveosaurus
Europasaurus
French "Bothriospondylus"
Lusotitan
Brachiosaurus
Giraffatitan
Sonorasaurus
Cedarosaurus
Venenosaurus
Dongbeititan
Brontomerus
"Cloverly titanosauriform"
Chubutisaurus
Tastavinsaurus
Ligabuesaurus
Sauroposeidon
Paluxysaurus
Wintonotitan
Astrophocaudia
Malarguesaurus
Australodocus
Fusuisaurus
Huanghetitan ruyangensis
Euhelopus
Gobititan
Erketu
Mongolosaurus
Qiaowanlong
Tangvayosaurus
Phuwiangosaurus
Fukuititan
Andesaurus
Ruyangosaurus
Xianshanosaurus
Malawisaurus
Aeolosaurus
Muyelensaurus
Rapetosaurus
Tapuiasaurus
Nemegtosaurus
Futalognkosaurus
Dongyangosaurus
Baotianmansaurus
Savannasaurus
AODF 836
Diamantinasaurus
Epachthosaurus
Isisaurus
Saltasaurus
Opisthocoelicaudia
Jiangshanosaurus
Alamosaurus

GC values, 5000 replicates, cut=1 (tree 0) - Symmetric Resampling (P=33)

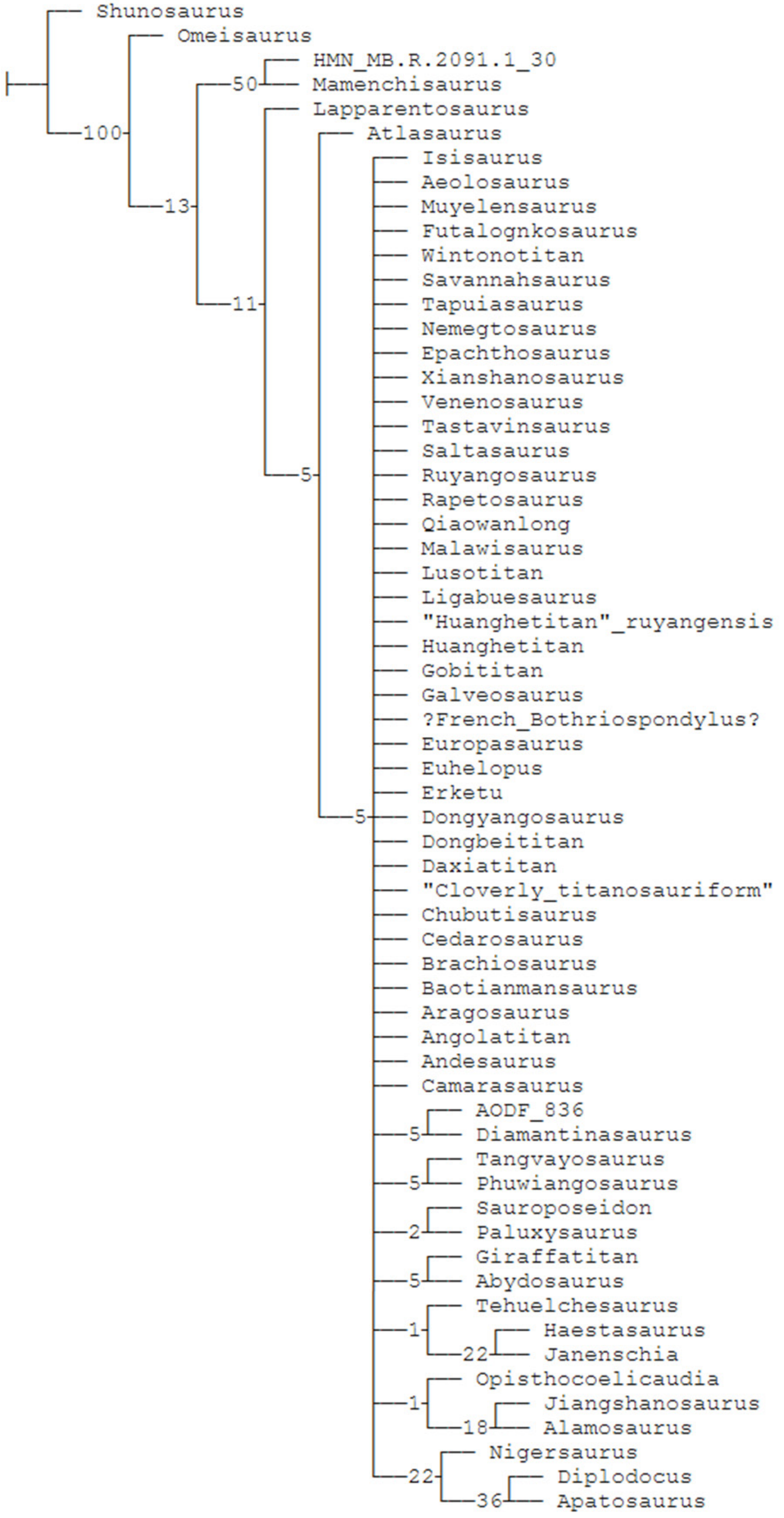

GC values, 5000 replicates, cut=1 (tree 0) - Symmetric Resampling (P=33)

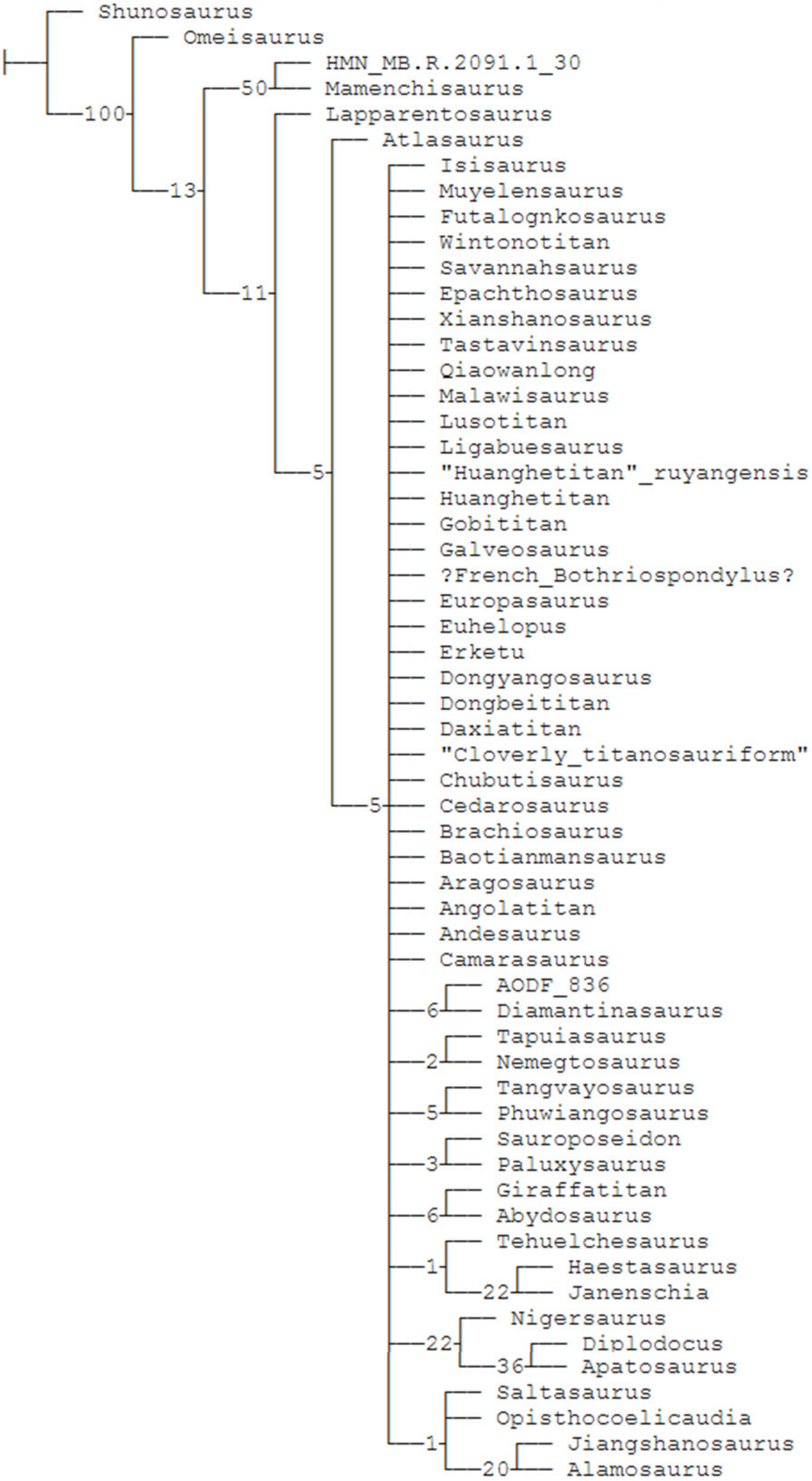

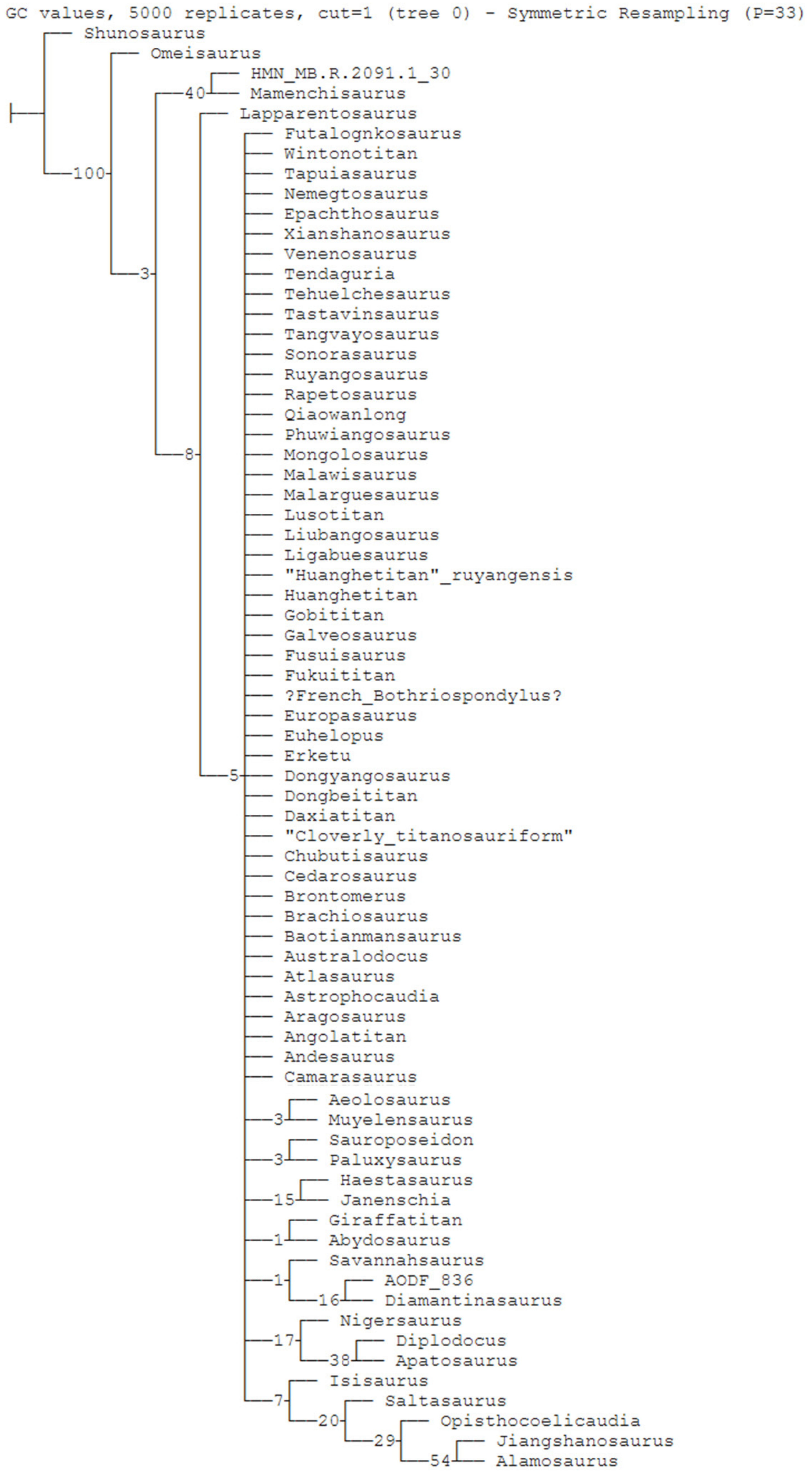
GC values, 5000 replicates, cut=1 (tree 0) - Symmetric Resampling (P=33)
Shunosaurus
Omeisaurus
HMN_MB.R.2091.1_30
Mamenchisaurus
40
Lapparentosaurus
Futalognkosaurus
Wintonotitan
Tapuiasaurus
Nemegtosaurus
Epachthosaurus
Xianshanosaurus
Venenosaurus
Tendaguria
Tehuelchesaurus
Tastavinsaurus
Tangvayosaurus
Sonorasaurus
Ruyangosaurus
Rapetosaurus
Qiaowanlong
Phuwiangosaurus
Mongolosaurus
Malawisaurus
Malarguesaurus
Lusotitan
Liubangosaurus
Ligabuesaurus
"Huanghetitan"_ruyangensis
Huanghetitan
Gobititan
Galveosaurus
Fusuisaurus
Fukuititan
?French_Bothriospondylus?
Europasaurus
Euhelopus
Erketu
Dongyangosaurus
Dongbeititan
Daxiatitan
"Cloverly_titanosauriform"
Chubutisaurus
Cedarosaurus
Brontomerus
Brachiosaurus
Baotianmansaurus
Australodocus
Atlasaurus
Astrophocaudia
Aragosaurus
Angolatitan
Andesaurus
Camarasaurus
Aeolosaurus
Muyelensaurus
3
Sauroposeidon
Paluxysaurus
3
Haestasaurus
Janenschia
15
Giraffatitan
Abydosaurus
1
Savannahsaurus
AODF_836
Diamantinasaurus
16
1
Nigersaurus
Diplodocus
Apatosaurus
38
17
Isisaurus
Saltasaurus
Opisthocoelicaudia
Jiangshanosaurus
Alamosaurus
54
29
20
7
5
8
3
100

GC values, 5000 replicates, cut=1 (tree 0) - Symmetric Resampling (P=33)

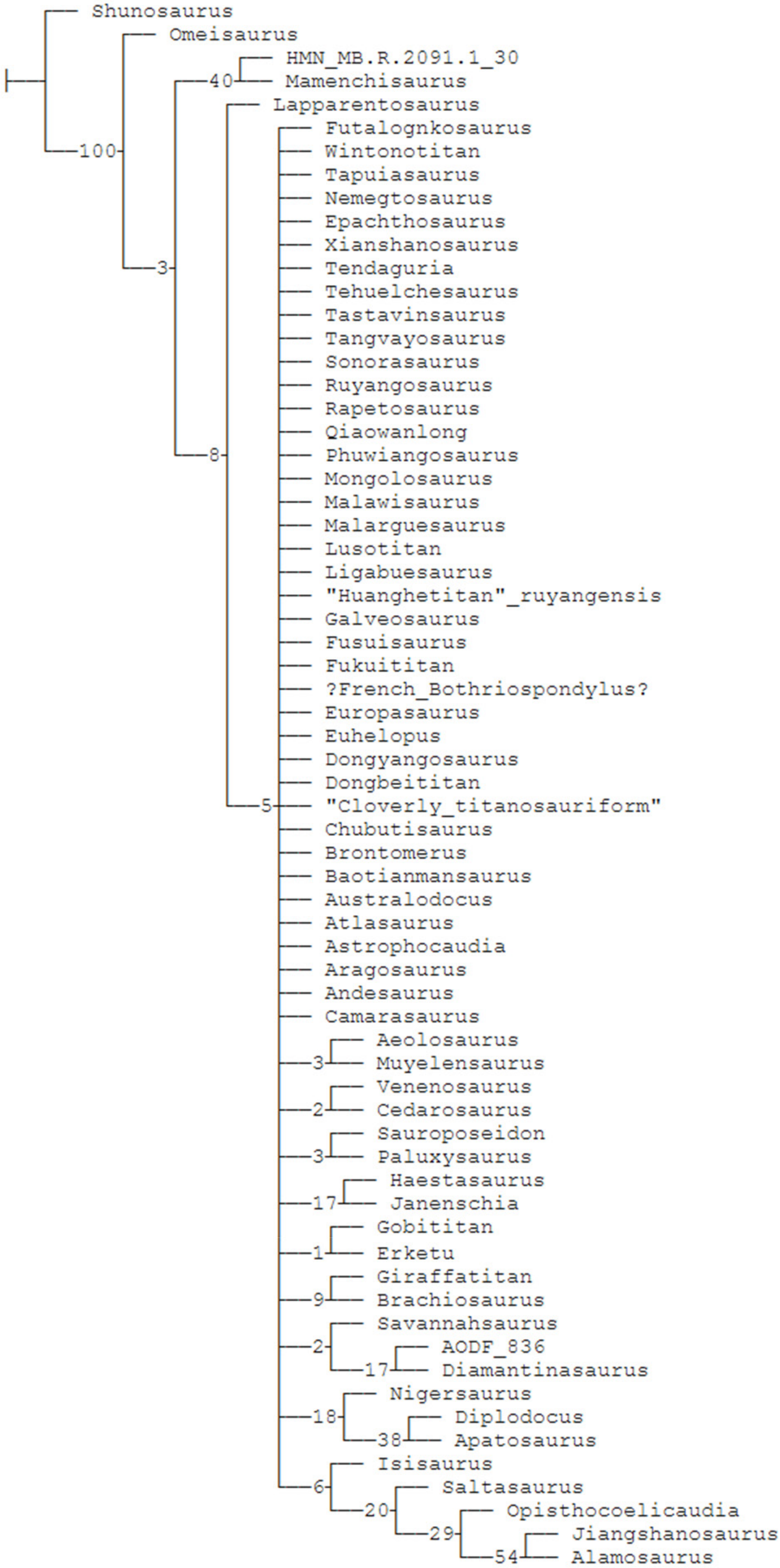

BioGeoBEARS DEC on Upchurch M3_stratified
ancstates: global optim, 7 areas max. d=0.0028; e=0.0082; j=0; LnL=−123.32

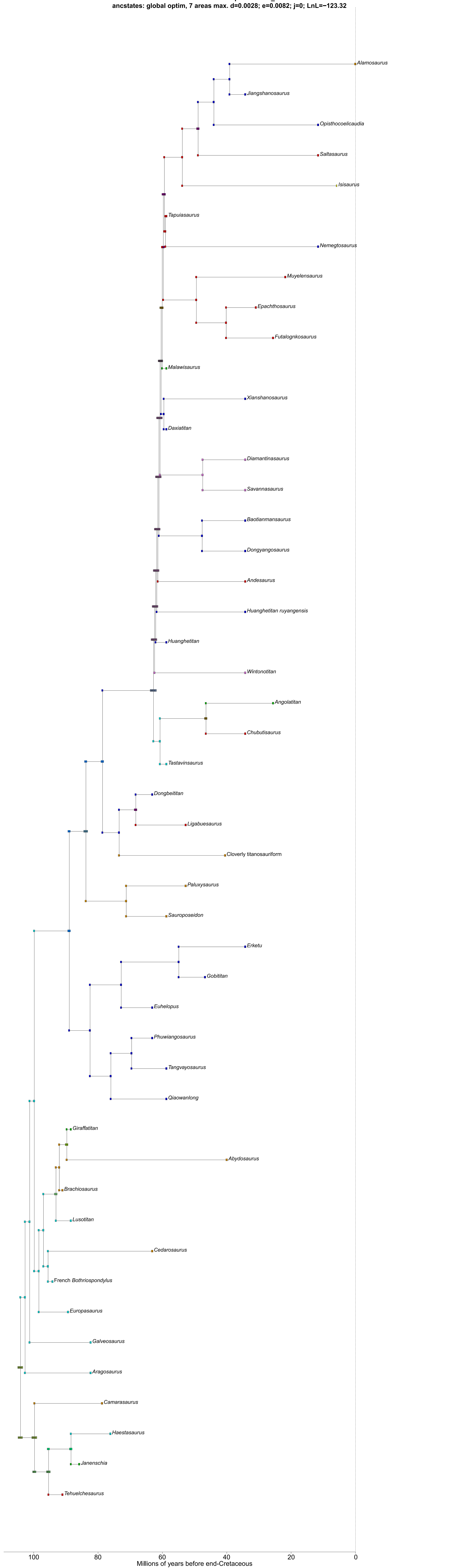

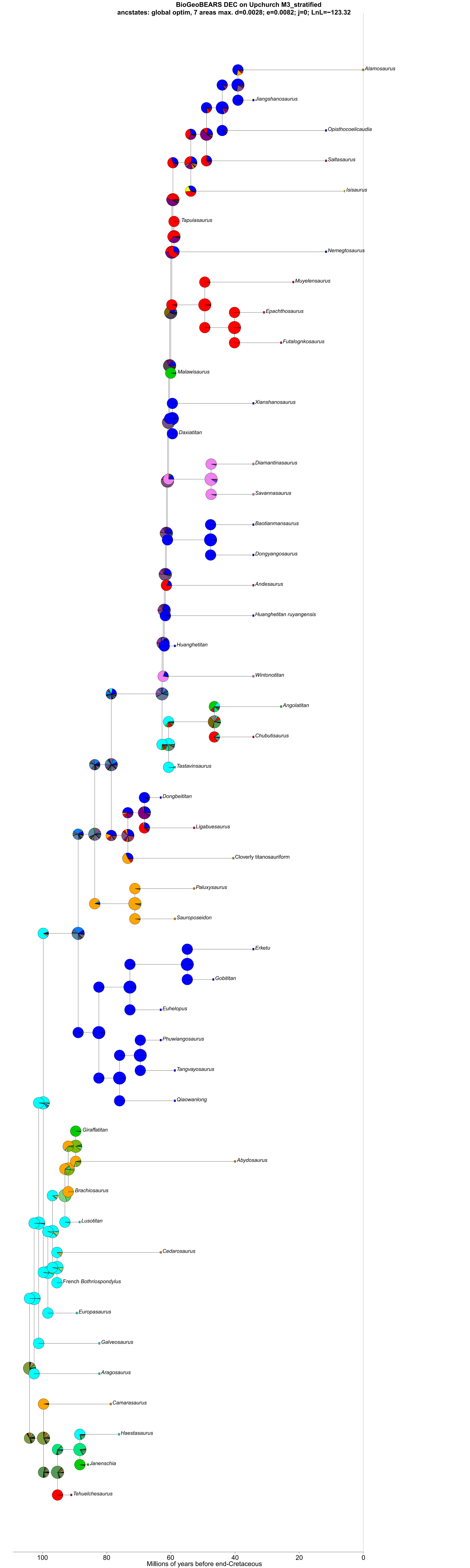
BioGeoBEARS DEC on Upchurch M3_stratified
ancstates: global optim, 7 areas max. d=0.0028; e=0.0082; j=0; LnL=-123.32
Alamosaurus
Jiangshanosaurus
Opisthocoelicaudia
Saltasaurus
Isisaurus
Tapuiasaurus
Nemegtosaurus
Muyelensaurus
Epachthosaurus
Futalognkosaurus
Malawisaurus
Xianshanosaurus
Daxiatitan
Diamantinasaurus
Savannasaurus
Baotianmansaurus
Dongyangosaurus
Andesaurus
Huanghetitan ruyangensis
Huanghetitan
Wintonotitan
Angolatitan
Chubutisaurus
Tastavinsaurus
Dongbeititan
Ligabuesaurus
Cloverly titanosauriform
Paluxysaurus
Sauroposeidon
Erketu
Gobititan
Euhelopus
Phuwiangosaurus
Tangvayosaurus
Qiaowanlong
Giraffatitan
Abydosaurus
Brachiosaurus
Lusotitan
Cedarosaurus
French Bothriospondylus
Europasaurus
Galveosaurus
Aragosaurus
Camarasaurus
Haestasaurus
Janenschia
Tehuelchesaurus
100
80
60
40
20
0
Millions of years before end-Cretaceous

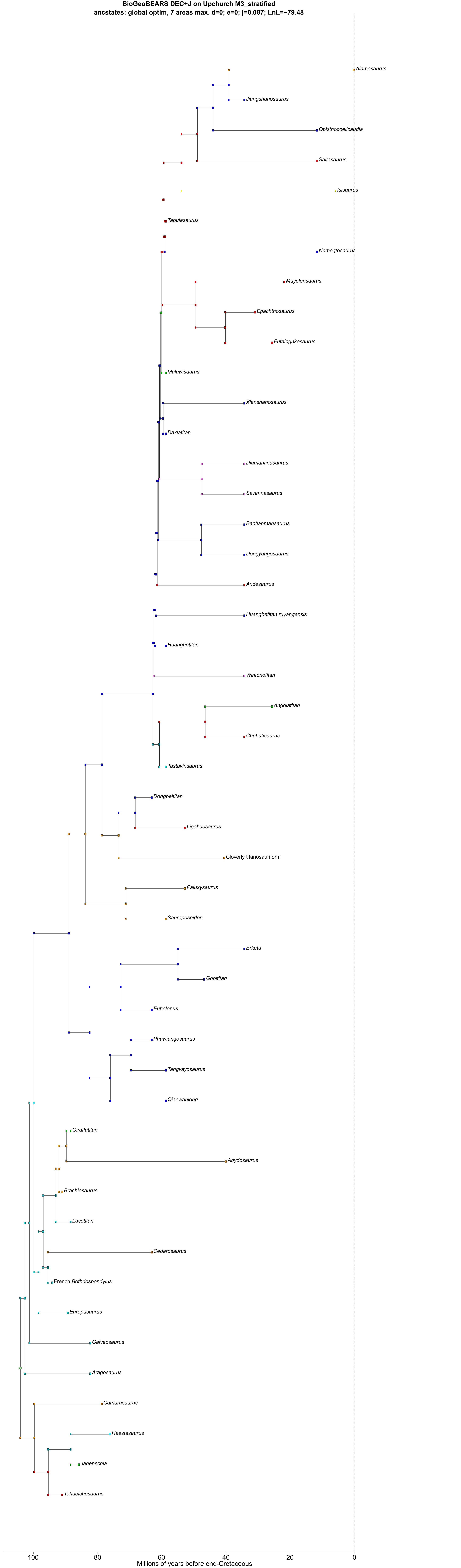

BioGeoBEARS DEC+J on Upchurch M3_stratified
ancstates: global optim, 7 areas max. d=0; e=0; j=0.087; LnL=−79.48
Alamosaurus
Jiangshanosaurus
Opisthocoelicaudia
Saltasaurus
Isisaurus
Tapuiasaurus
Nemegtosaurus
Muyelensaurus
Epachthosaurus
Futalognkosaurus
Malawisaurus
Xianshanosaurus
Daxiatitan
Diamantinasaurus
Savannasaurus
Baotianmansaurus
Dongyangosaurus
Andesaurus
Huanghetitan ruyangensis
Huanghetitan
Wintonotitan
Angolatitan
Chubutisaurus
Tastavinsaurus
Dongbeititan
Ligabuesaurus
Cloverly titanosauriform
Paluxysaurus
Sauroposeidon
Erketu
Gobititan
Euhelopus
Phuwiangosaurus
Tangvayosaurus
Qiaowanlong
Giraffatitan
Abydosaurus
Brachiosaurus
Lusotitan
Cedarosaurus
French Bothriospondylus
Europasaurus
Galveosaurus
Aragosaurus
Camarasaurus
Haestasaurus
Janenschia
Tehuelchesaurus
100
80
60
40
20
0
Millions of years before end-Cretaceous

BioGeoBEARS DEC+J on Upchurch M3_stratified
ancstates: global optim, 7 areas max. d=0; e=0; j=0.087; LnL=-79.48

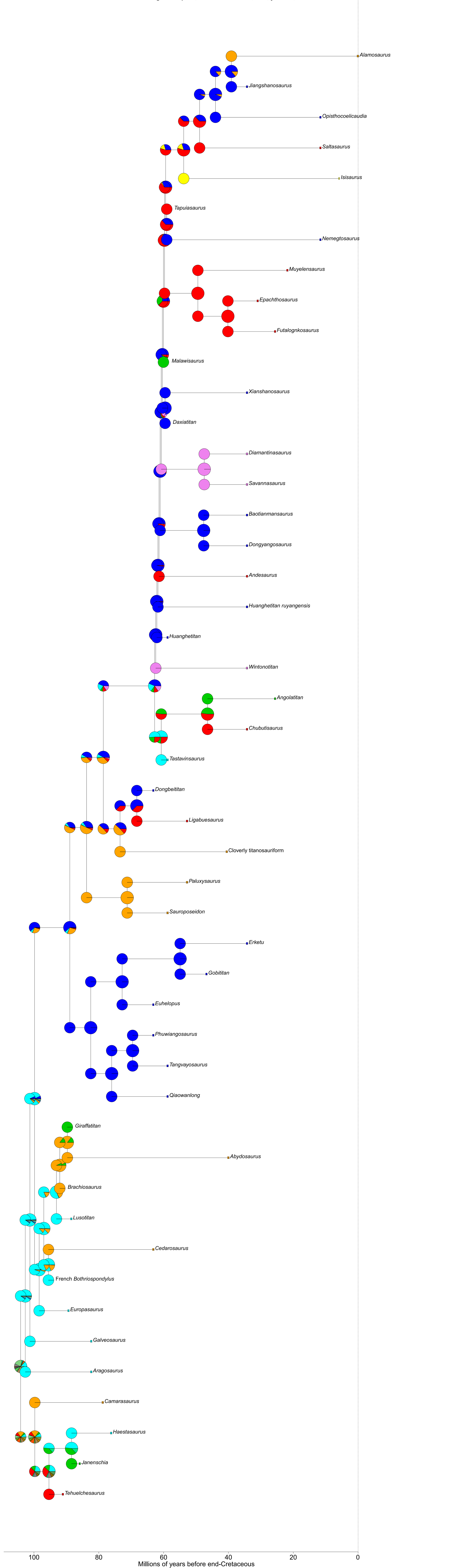

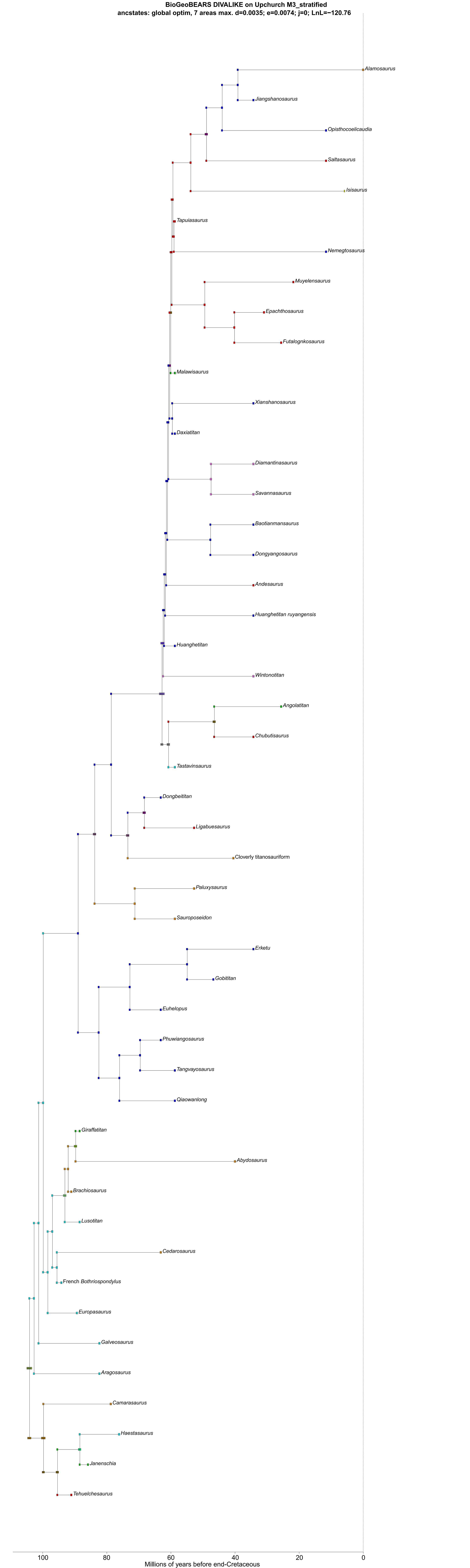

BioGeoBEARS DIVALIKE on Upchurch M3_stratified
ancstates: global optim, 7 areas max. d=0.0035; e=0.0074; j=0; LnL=−120.76
Alamosaurus
Jiangshanosaurus
Opisthocoelicaudia
Saltasaurus
Isisaurus
Tapuiasaurus
Nemegtosaurus
Muyelensaurus
Epachthosaurus
Futalognkosaurus
Malawisaurus
Xianshanosaurus
Daxiatitan
Diamantinasaurus
Savannasaurus
Baotianmansaurus
Dongyangosaurus
Andesaurus
Huanghetitan ruyangensis
Huanghetitan
Wintonotitan
Angolatitan
Chubutisaurus
Tastavinsaurus
Dongbeititan
Ligabuesaurus
Cloverly titanosauriform
Paluxysaurus
Sauroposeidon
Erketu
Gobititan
Euhelopus
Phuwiangosaurus
Tangvayosaurus
Qiaowanlong
Giraffatitan
Abydosaurus
Brachiosaurus
Lusotitan
Cedarosaurus
French Bothriospondylus
Europasaurus
Galveosaurus
Aragosaurus
Camarasaurus
Haestasaurus
Janenschia
Tehuelchesaurus
100
80
60
40
20
0
Millions of years before end-Cretaceous

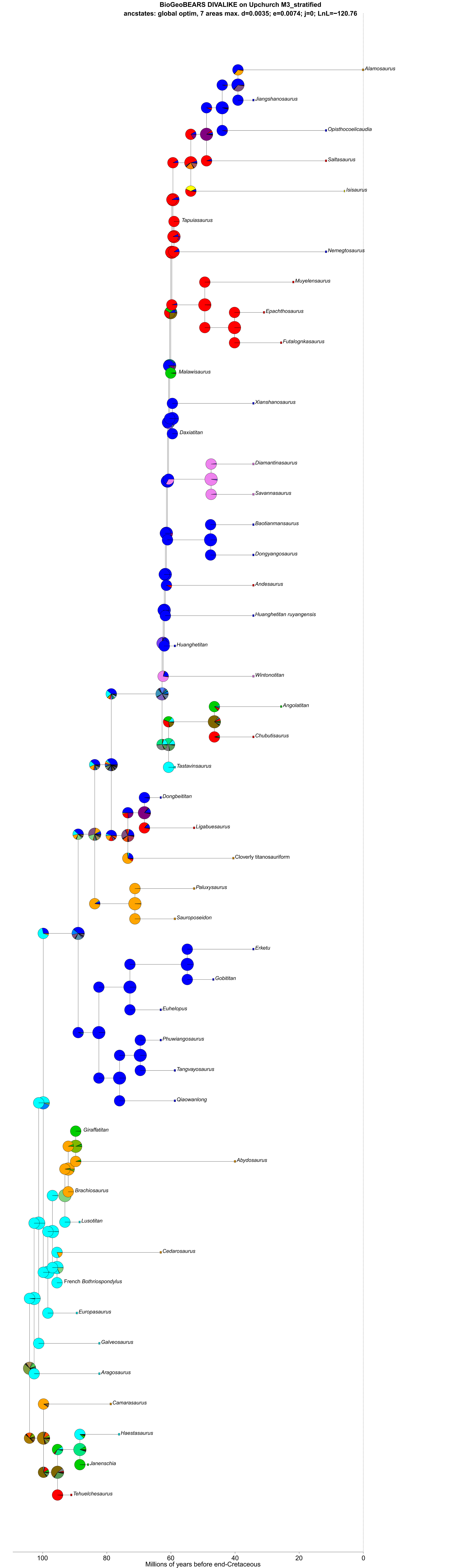
BioGeoBEARS DIVALIKE on Upchurch M3_stratified
ancstates: global optim, 7 areas max. d=0.0035; e=0.0074; j=0; LnL=-120.76
Alamosaurus
Jiangshanosaurus
Opisthocoelicaudia
Saltasaurus
Isisaurus
Tapuiasaurus
Nemegtosaurus
Muyelensaurus
Epachthosaurus
Futalognkosaurus
Malawisaurus
Xianshanosaurus
Daxiatitan
Diamantinasaurus
Savannasaurus
Baotianmansaurus
Dongyangosaurus
Andesaurus
Huanghetitan ruyangensis
Huanghetitan
Wintonotitan
Angolatitan
Chubutisaurus
Tastavinsaurus
Dongbeititan
Ligabuesaurus
Cloverly titanosauriform
Paluxysaurus
Sauroposeidon
Erketu
Gobititan
Euhelopus
Phuwiangosaurus
Tangvayosaurus
Qiaowanlong
Giraffatitan
Abydosaurus
Brachiosaurus
Lusotitan
Cedarosaurus
French Bothriospondylus
Europasaurus
Galveosaurus
Aragosaurus
Camarasaurus
Haestasaurus
Janenschia
Tehuelchesaurus
100
80
60
40
20
0
Millions of years before end-Cretaceous

BioGeoBEARS DIVALIKE+J on Upchurch M3_stratified
ancstates: global optim, 7 areas max. d=0; e=0; j=0.0845; LnL=−78.85

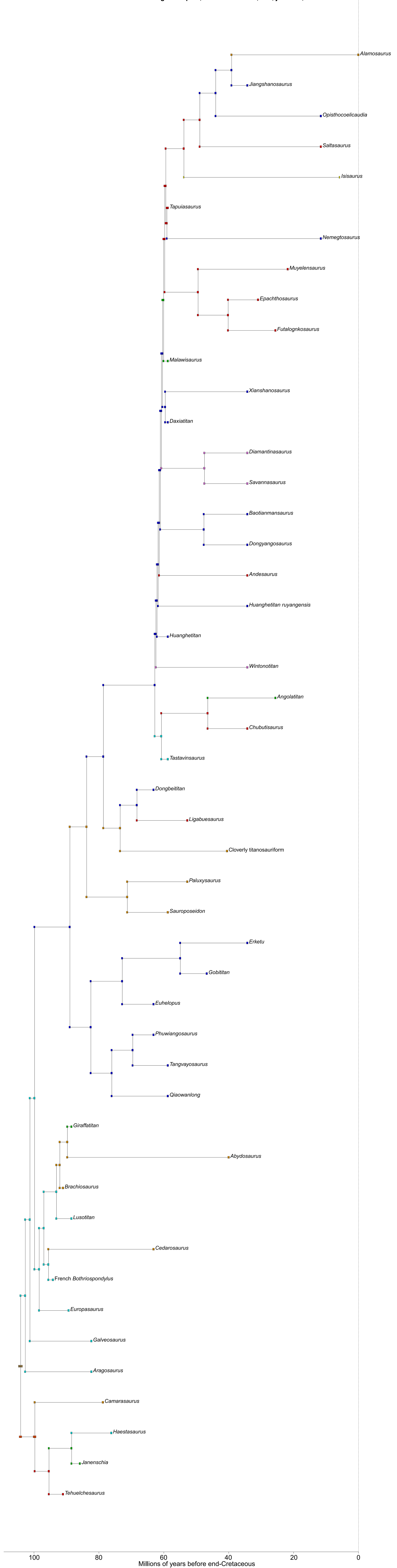

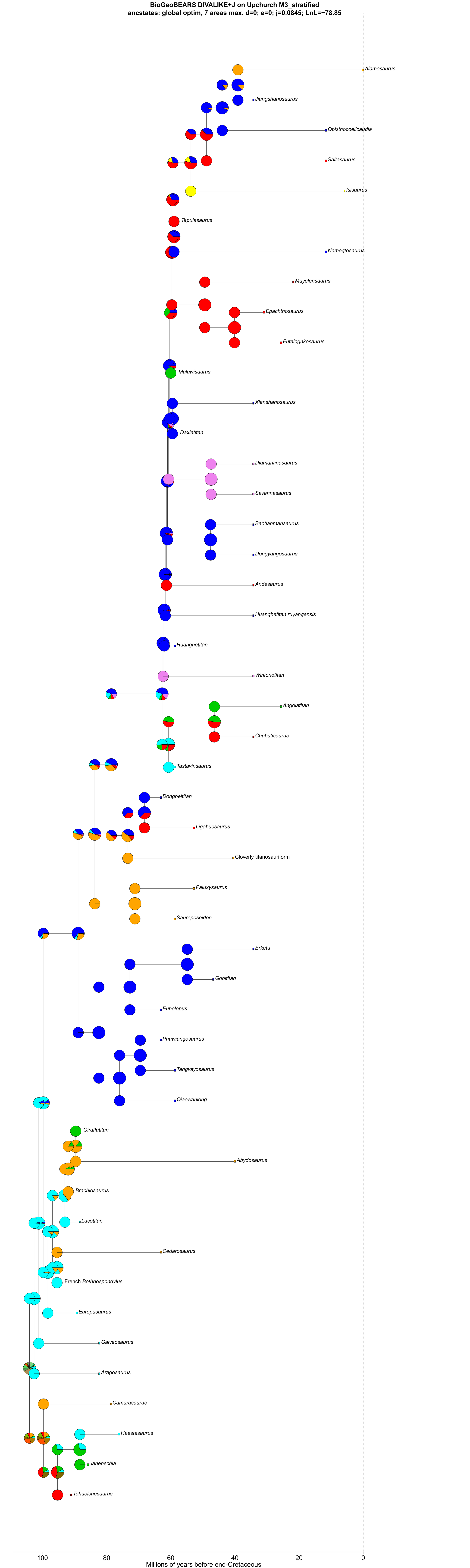
BioGeoBEARS DIVALIKE+J on Upchurch M3_stratified
ancstates: global optim, 7 areas max. d=0; e=0; j=0.0845; LnL=−78.85
Alamosaurus
Jiangshanosaurus
Opisthocoelicaudia
Saltasaurus
Isisaurus
Tapuiasaurus
Nemegtosaurus
Muyelensaurus
Epachthosaurus
Futalognkosaurus
Malawisaurus
Xianshanosaurus
Daxiatitan
Diamantinasaurus
Savannasaurus
Baotianmansaurus
Dongyangosaurus
Andesaurus
Huanghetitan ruyangensis
Huanghetitan
Wintonotitan
Angolatitan
Chubutisaurus
Tastavinsaurus
Dongbeititan
Ligabuesaurus
Cloverly titanosauriform
Paluxysaurus
Sauroposeidon
Erketu
Gobititan
Euhelopus
Phuwiangosaurus
Tangvayosaurus
Qiaowanlong
Giraffatitan
Abydosaurus
Brachiosaurus
Lusotitan
Cedarosaurus
French Bothriospondylus
Europasaurus
Galveosaurus
Aragosaurus
Camarasaurus
Haestasaurus
Janenschia
Tehuelchesaurus
100
80
60
40
20
0
Millions of years before end-Cretaceous

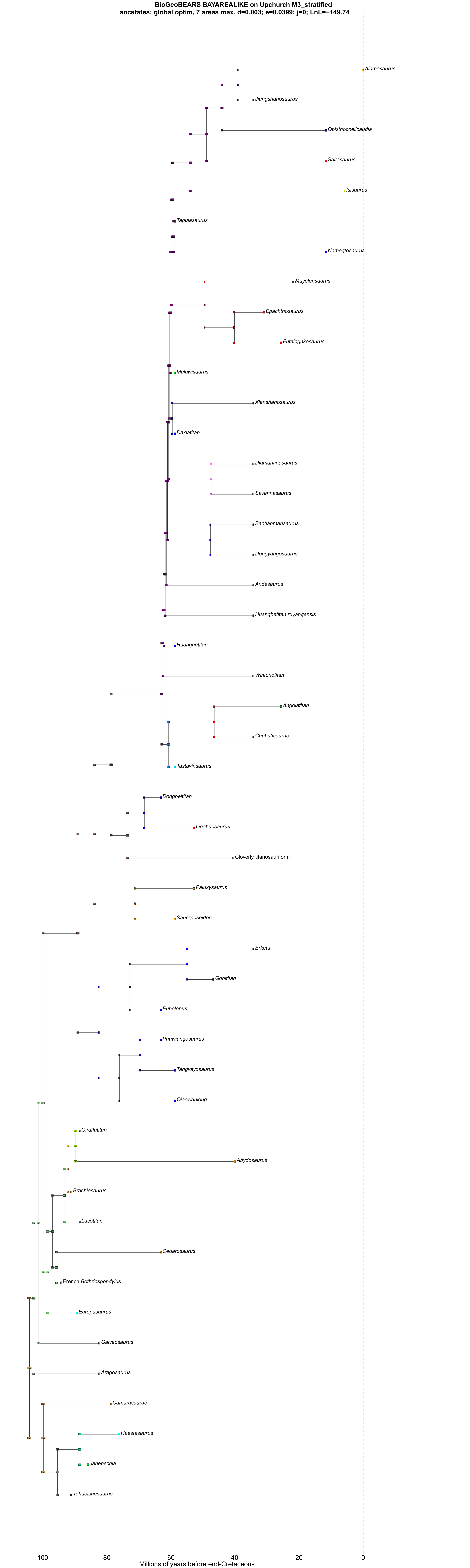
BioGeoBEARS BAYAREALIKE on Upchurch M3_stratified
ancstates: global optim, 7 areas max. d=0.003; e=0.0399; j=0; LnL=-149.74
Alamosaurus
Jiangshanosaurus
Opisthocoelicaudia
Saltasaurus
Isisaurus
Tapuiasaurus
Nemegtosaurus
Muyelensaurus
Epachthosaurus
Futalognkosaurus
Malawisaurus
Xianshanosaurus
Daxiatitan
Diamantinasaurus
Savannasaurus
Baotianmansaurus
Dongyangosaurus
Andesaurus
Huanghetitan ruyangensis
Huanghetitan
Wintonotitan
Angolatitan
Chubutisaurus
Tastavinsaurus
Dongbeititan
Ligabuesaurus
Cloverly titanosauriform
Paluxysaurus
Sauroposeidon
Erketu
Gobititan
Euhelopus
Phuwiangosaurus
Tangvayosaurus
Qiaowanlong
Giraffatitan
Abydosaurus
Brachiosaurus
Lusotitan
Cedarosaurus
French Bothriospondylus
Europasaurus
Galveosaurus
Aragosaurus
Camarasaurus
Haestasaurus
Janenschia
Tehuelchesaurus
100
80
60
40
20
0
Millions of years before end-Cretaceous

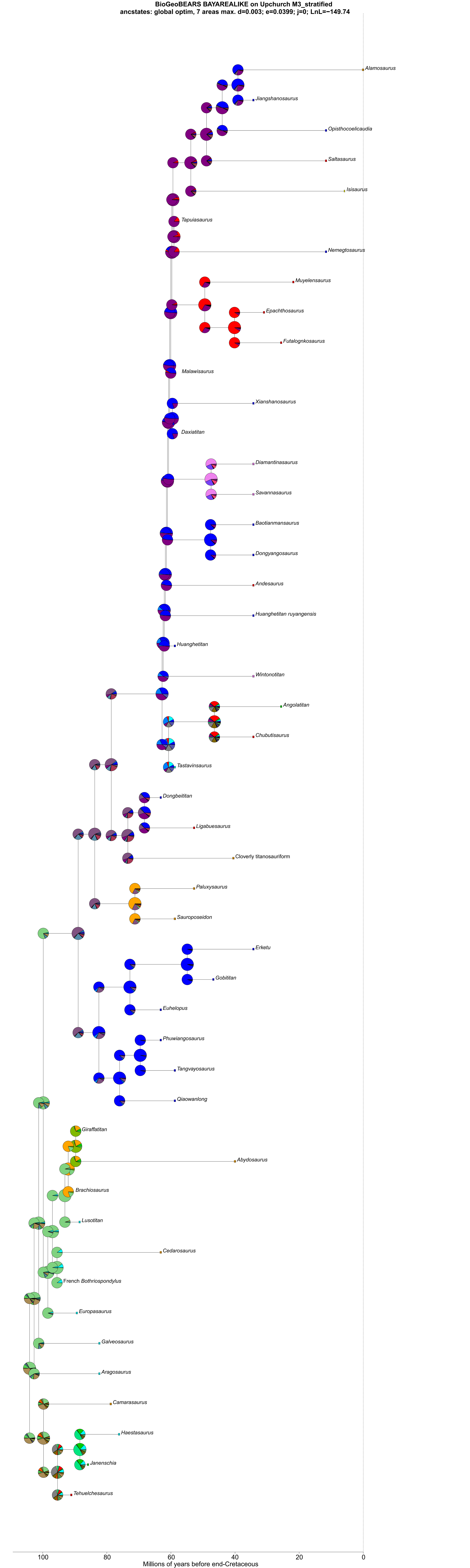
BioGeoBEARS BAYAREALIKE on Upchurch M3_stratified
ancstates: global optim, 7 areas max. d=0.003; e=0.0399; j=0; LnL=-149.74
Alamosaurus
Jiangshanosaurus
Opisthocoelicaudia
Saltasaurus
Isisaurus
Tapuiasaurus
Nemegtosaurus
Muyelensaurus
Epachthosaurus
Futalognkosaurus
Malawisaurus
Xianshanosaurus
Daxiatitan
Diamantinasaurus
Savannasaurus
Baotianmansaurus
Dongyangosaurus
Andesaurus
Huanghetitan ruyangensis
Huanghetitan
Wintonotitan
Angolatitan
Chubutisaurus
Tastavinsaurus
Dongbeititan
Ligabuesaurus
Cloverly titanosauriform
Paluxysaurus
Sauroposeidon
Erketu
Gobititan
Euhelopus
Phuwiangosaurus
Tangvayosaurus
Qiaowanlong
Giraffatitan
Abydosaurus
Brachiosaurus
Lusotitan
Cedarosaurus
French Bothriospondylus
Europasaurus
Galveosaurus
Aragosaurus
Camarasaurus
Haestasaurus
Janenschia
Tehuelchesaurus
100
80
60
40
20
0
Millions of years before end-Cretaceous

BioGeoBEARS BAYAREALIKE+J on Upchurch M3_stratified
ancstates: global optim, 7 areas max. d=0; e=0; j=0.0865; LnL=−81.17

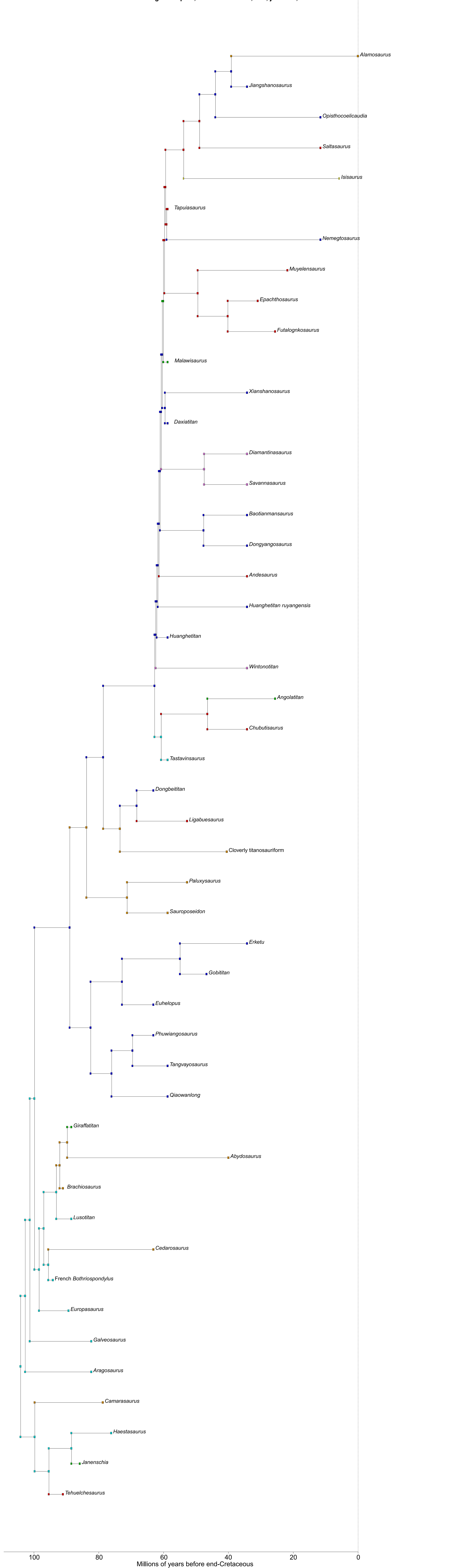

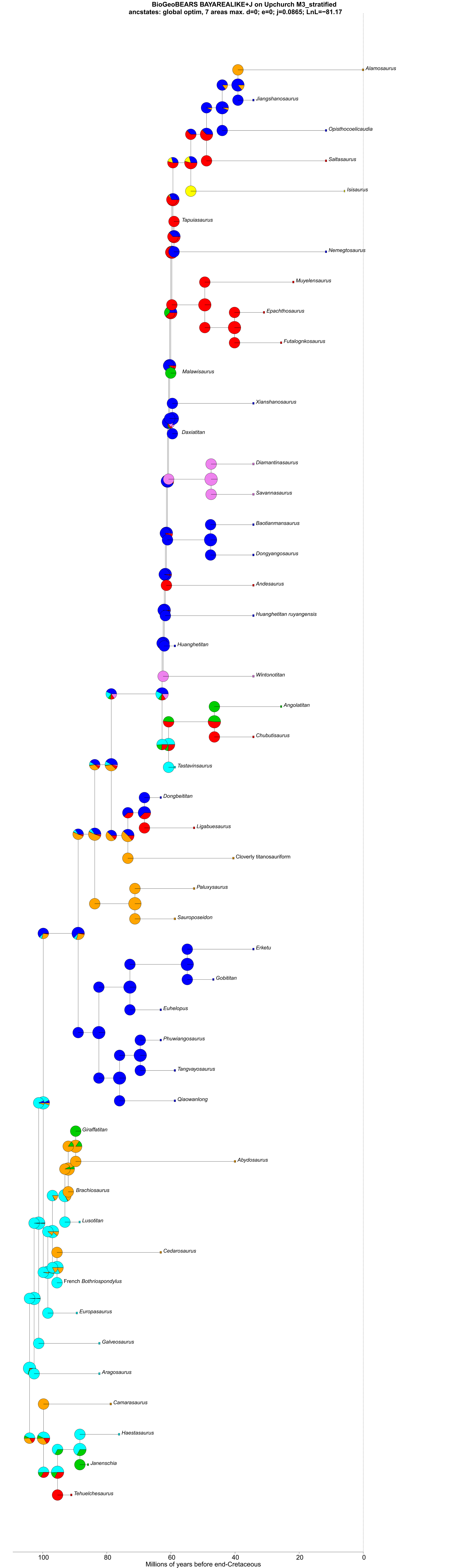
BioGeoBEARS BAYAREALIKE+J on Upchurch M3_stratified
ancstates: global optim, 7 areas max. d=0; e=0; j=0.0865; LnL=−81.17
Alamosaurus
Jiangshanosaurus
Opisthocoelicaudia
Saltasaurus
Isisaurus
Tapuiasaurus
Nemegtosaurus
Muyelensaurus
Epachthosaurus
Futalognkosaurus
Malawisaurus
Xianshanosaurus
Daxiatitan
Diamantinasaurus
Savannasaurus
Baotianmansaurus
Dongyangosaurus
Andesaurus
Huanghetitan ruyangensis
Huanghetitan
Wintonotitan
Angolatitan
Chubutisaurus
Tastavinsaurus
Dongbeititan
Ligabuesaurus
Cloverly titanosauriform
Paluxysaurus
Sauroposeidon
Erketu
Gobititan
Euhelopus
Phuwiangosaurus
Tangvayosaurus
Qiaowanlong
Giraffatitan
Abydosaurus
Brachiosaurus
Lusotitan
Cedarosaurus
French Bothriospondylus
Europasaurus
Galveosaurus
Aragosaurus
Camarasaurus
Haestasaurus
Janenschia
Tehuelchesaurus
100
80
60
40
20
0
Millions of years before end-Cretaceous

BioGeoBEARS DEC on Upchurch M3_stratified
ancstates: global optim, 7 areas max. d=0.0025; e=0.0071; j=0; LnL=−121.61

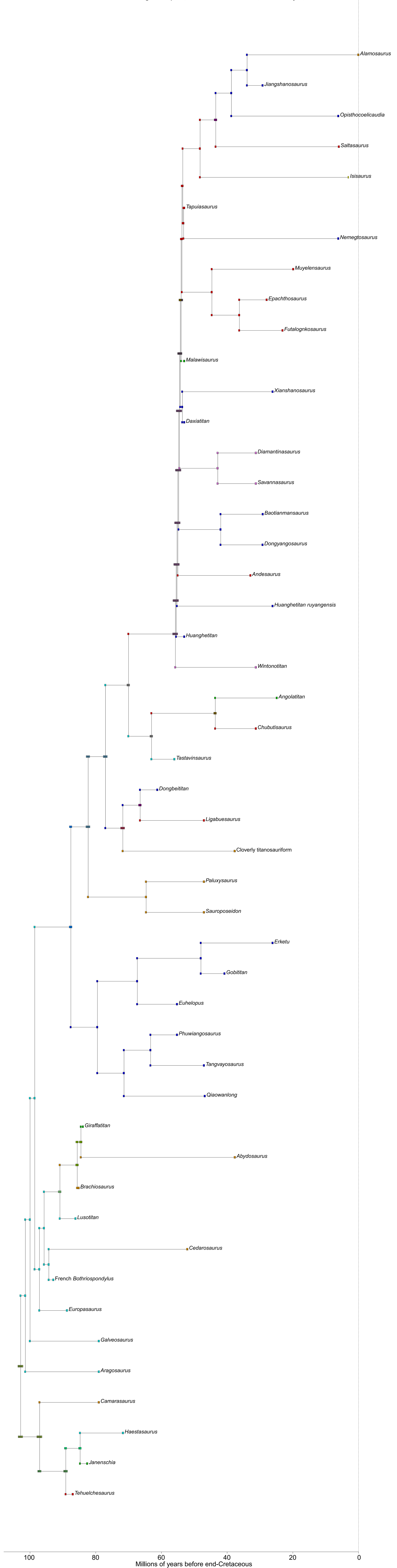

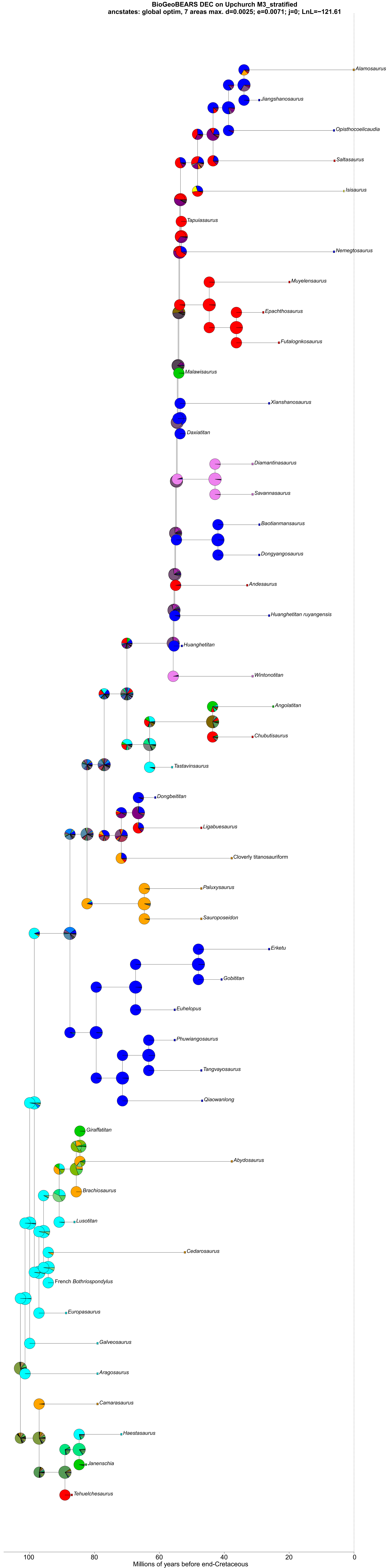
BioGeoBEARS DEC on Upchurch M3_stratified
ancstates: global optim, 7 areas max. d=0.0025; e=0.0071; j=0; LnL=-121.61
Alamosaurus
Jiangshanosaurus
Opisthocoelicaudia
Saltasaurus
Isisaurus
Tapuiasaurus
Nemegtosaurus
Muyelensaurus
Epachthosaurus
Futalognkosaurus
Malawisaurus
Xianshanosaurus
Daxiatitan
Diamantinasaurus
Savannasaurus
Baotianmansaurus
Dongyangosaurus
Andesaurus
Huanghetitan ruyangensis
Huanghetitan
Wintonotitan
Angolatitan
Chubutisaurus
Tastavinsaurus
Dongbeititan
Ligabuesaurus
Cloverly titanosauriform
Paluxysaurus
Sauroposeidon
Erketu
Gobititan
Euhelopus
Phuwiangosaurus
Tangvayosaurus
Qiaowanlong
Giraffatitan
Abydosaurus
Brachiosaurus
Lusotitan
Cedarosaurus
French Bothriospondylus
Europasaurus
Galveosaurus
Aragosaurus
Camarasaurus
Haestasaurus
Janenschia
Tehuelchesaurus
100
80
60
40
20
0
Millions of years before end-Cretaceous

BioGeoBEARS DEC+J on Upchurch M3_stratified
ancstates: global optim, 7 areas max. d=0; e=0; j=0.087; LnL=−79.48

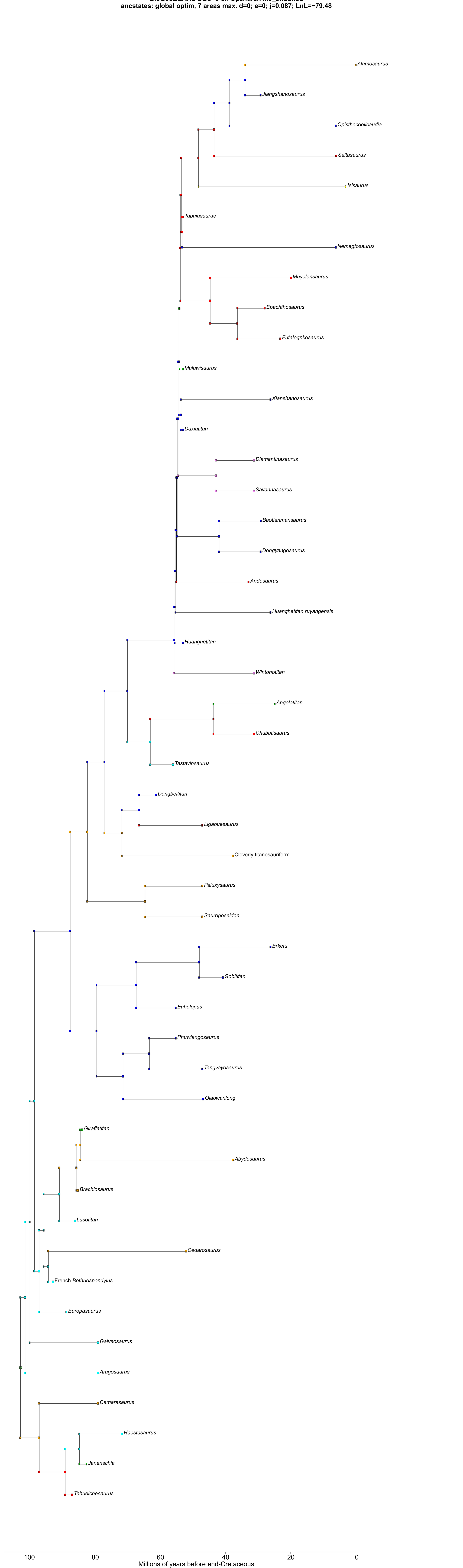

BioGeoBEARS DEC+J on Upchurch M3_stratified
ancstates: global optim, 7 areas max. d=0; e=0; j=0.087; LnL=-79.48

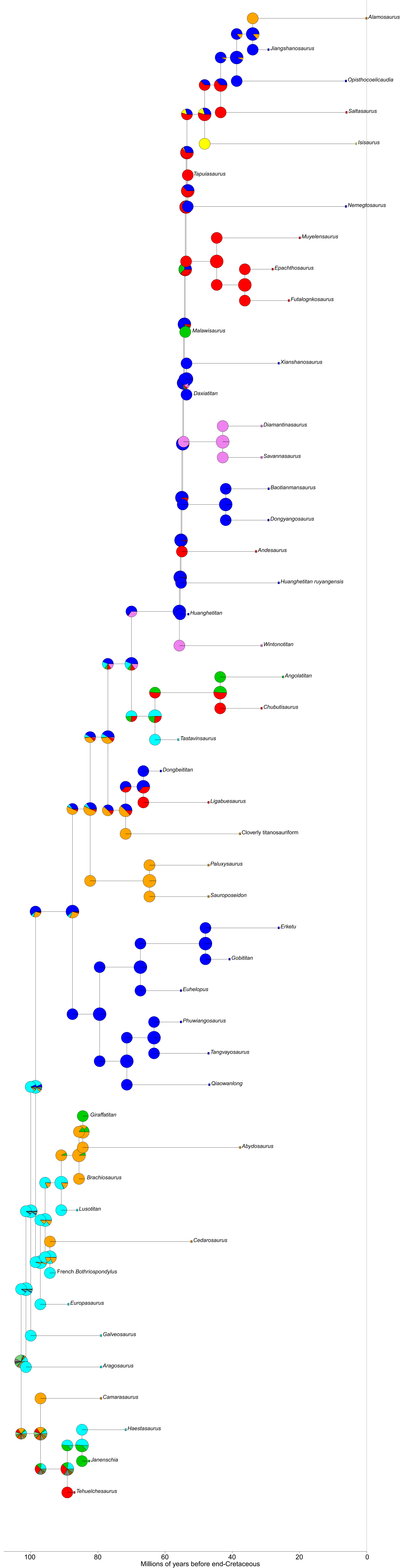

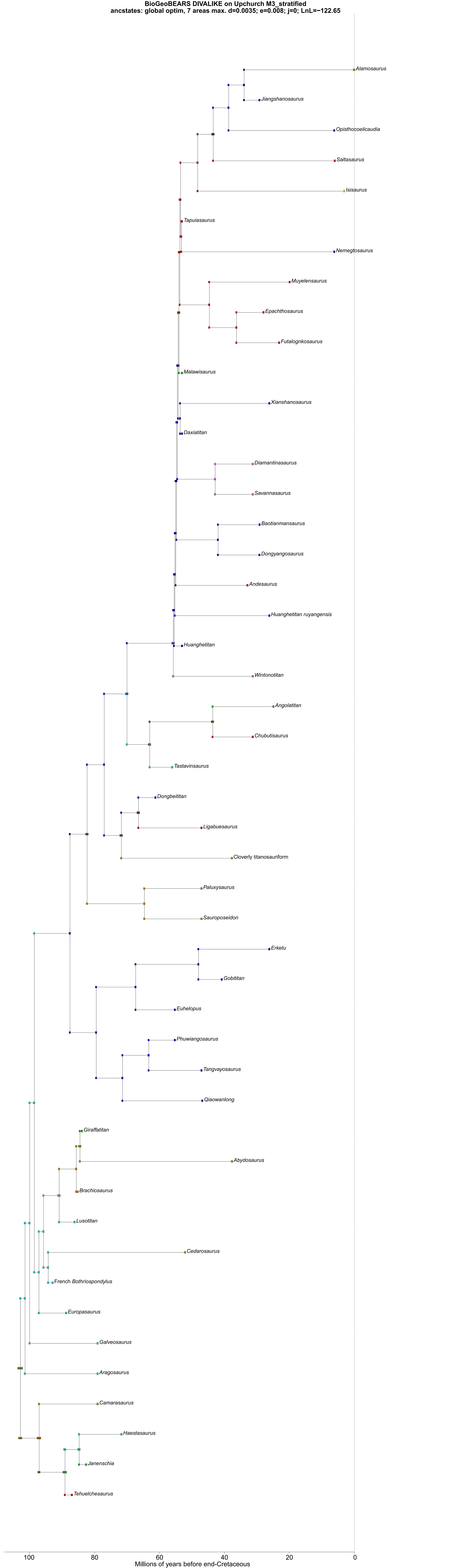
BioGeoBEARS DIVALIKE on Upchurch M3_stratified
ancstates: global optim, 7 areas max. d=0.0035; e=0.008; j=0; LnL=-122.65
Alamosaurus
Jiangshanosaurus
Opisthocoelicaudia
Saltasaurus
Isisaurus
Tapuiasaurus
Nemegtosaurus
Muyelensaurus
Epachthosaurus
Futalognkosaurus
Malawisaurus
Xianshanosaurus
Daxiatitan
Diamantinasaurus
Savannasaurus
Baotianmansaurus
Dongyangosaurus
Andesaurus
Huanghetitan ruyangensis
Huanghetitan
Wintonotitan
Angolatitan
Chubutisaurus
Tastavinsaurus
Dongbeititan
Ligabuesaurus
Cloverly titanosauriform
Paluxysaurus
Sauroposeidon
Erketu
Gobititan
Euhelopus
Phuwiangosaurus
Tangvayosaurus
Qiaowanlong
Giraffatitan
Abydosaurus
Brachiosaurus
Lusotitan
Cedarosaurus
French Bothriospondylus
Europasaurus
Galveosaurus
Aragosaurus
Camarasaurus
Haestasaurus
Janenschia
Tehuelchesaurus
100
80
60
40
20
0
Millions of years before end-Cretaceous

BioGeoBEARS DIVALIKE on Upchurch M3_stratified
ancstates: global optim, 7 areas max. d=0.0035; e=0.008; j=0; LnL=-122.65

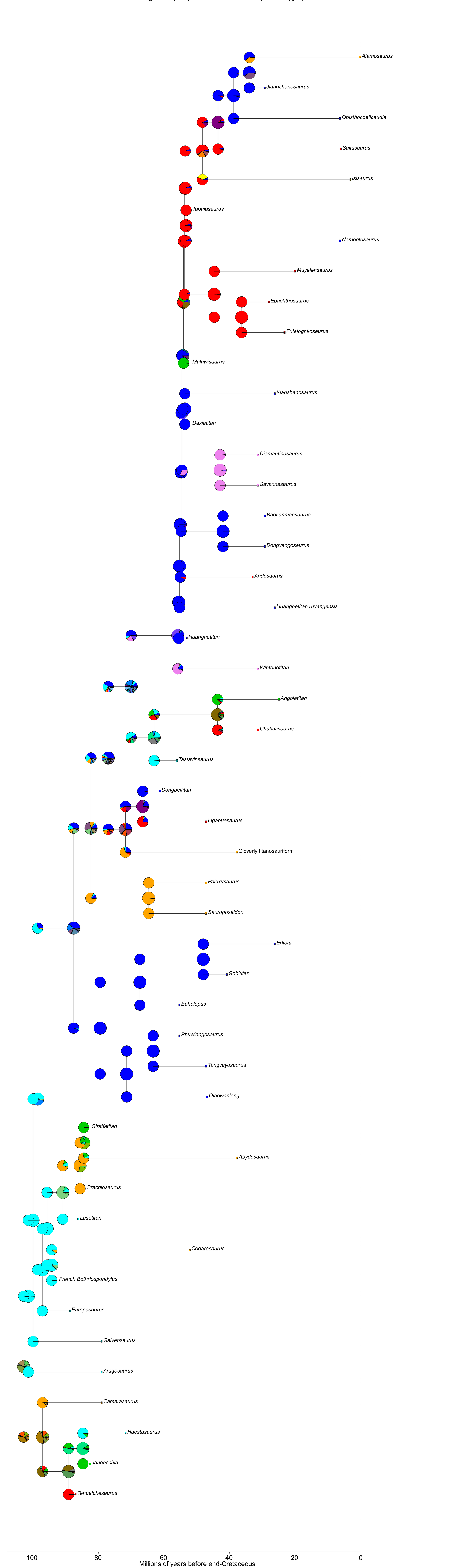

BioGeoBEARS DIVALIKE+J on Upchurch M3_stratified
ancstates: global optim, 7 areas max. d=0; e=0; j=0.0845; LnL=-78.85

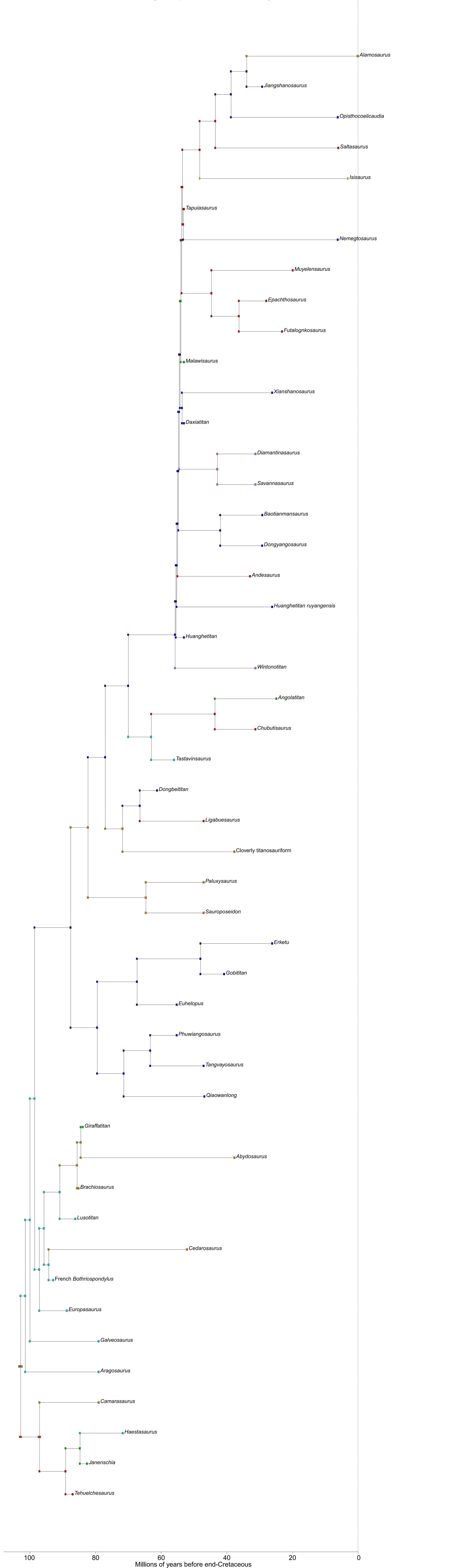

BioGeoBEARS DIVALIKE+J on Upchurch M3_stratified
ancstates: global optim, 7 areas max. d=0; e=0; j=0.0845; LnL=-78.85

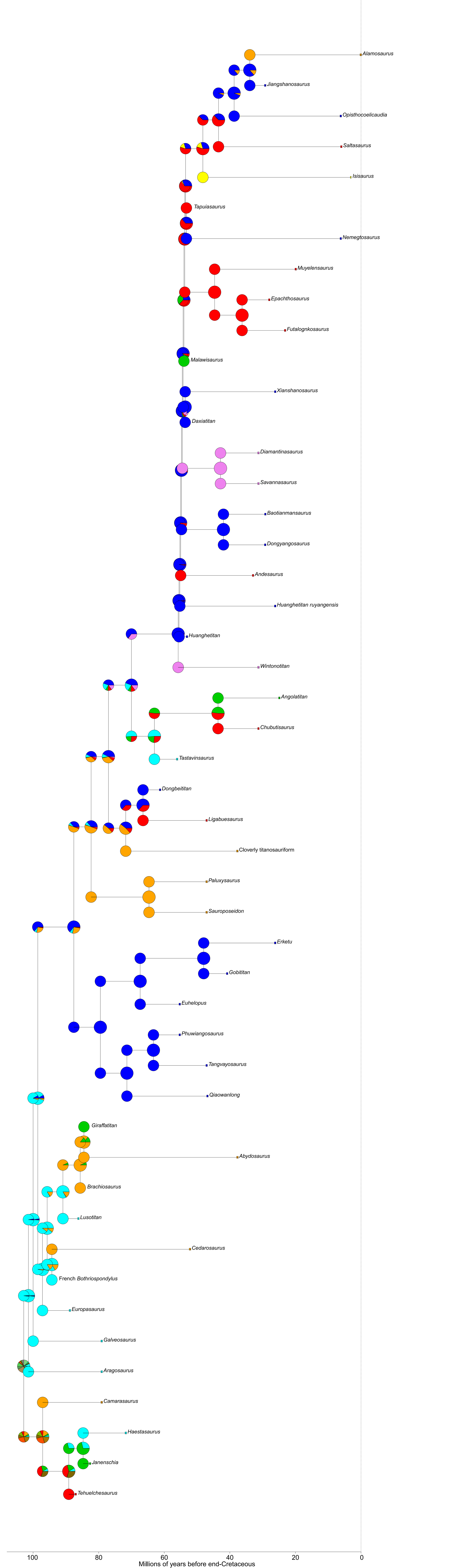

BioGeoBEARS BAYAREALIKE on Upchurch M3_stratified
ancstates: global optim, 7 areas max. d=0.003; e=0.0377; j=0; LnL=-150.29

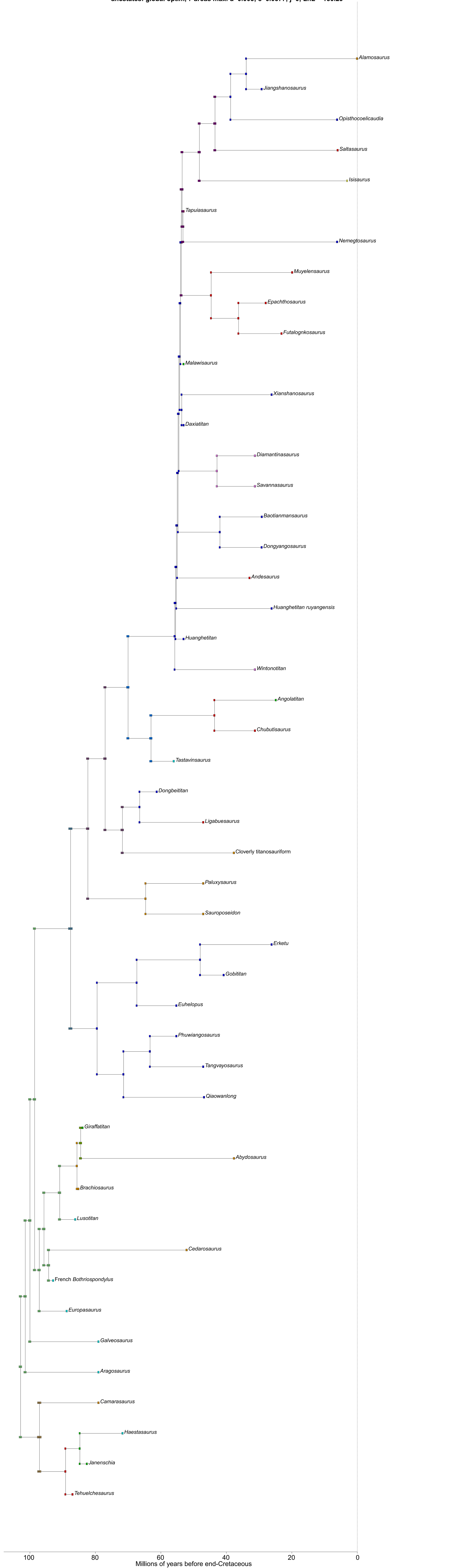

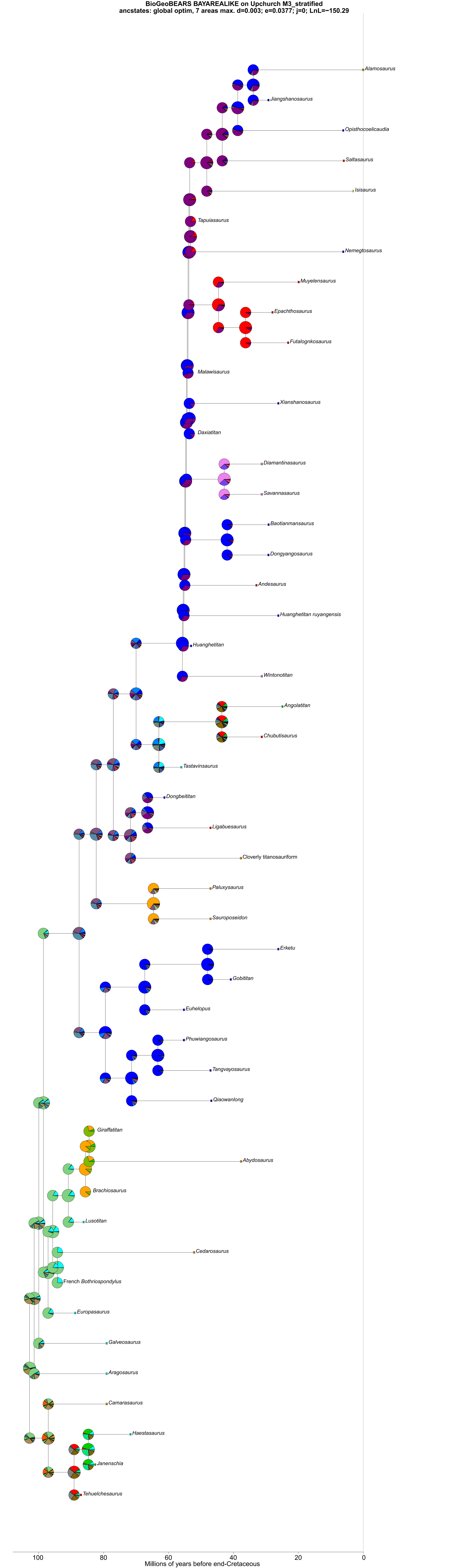

BioGeoBEARS BAYAREALIKE on Upchurch M3_stratified
ancstates: global optim, 7 areas max. d=0.003; e=0.0377; j=0; LnL=-150.29
Alamosaurus
Jiangshanosaurus
Opisthocoelicaudia
Saltasaurus
Isisaurus
Tapuiasaurus
Nemegtosaurus
Muyelensaurus
Epachthosaurus
Futalognkosaurus
Malawisaurus
Xianshanosaurus
Daxiatitan
Diamantinasaurus
Savannasaurus
Baotianmansaurus
Dongyangosaurus
Andesaurus
Huanghetitan ruyangensis
Huanghetitan
Wintonotitan
Angolatitan
Chubutisaurus
Tastavinsaurus
Dongbeititan
Ligabuesaurus
Cloverly titanosauriform
Paluxysaurus
Sauroposeidon
Erketu
Gobititan
Euhelopus
Phuwiangosaurus
Tangvayosaurus
Qiaowanlong
Giraffatitan
Abydosaurus
Brachiosaurus
Lusotitan
Cedarosaurus
French Bothriospondylus
Europasaurus
Galveosaurus
Aragosaurus
Camarasaurus
Haestasaurus
Janenschia
Tehuelchesaurus
100
80
60
40
20
0
Millions of years before end-Cretaceous

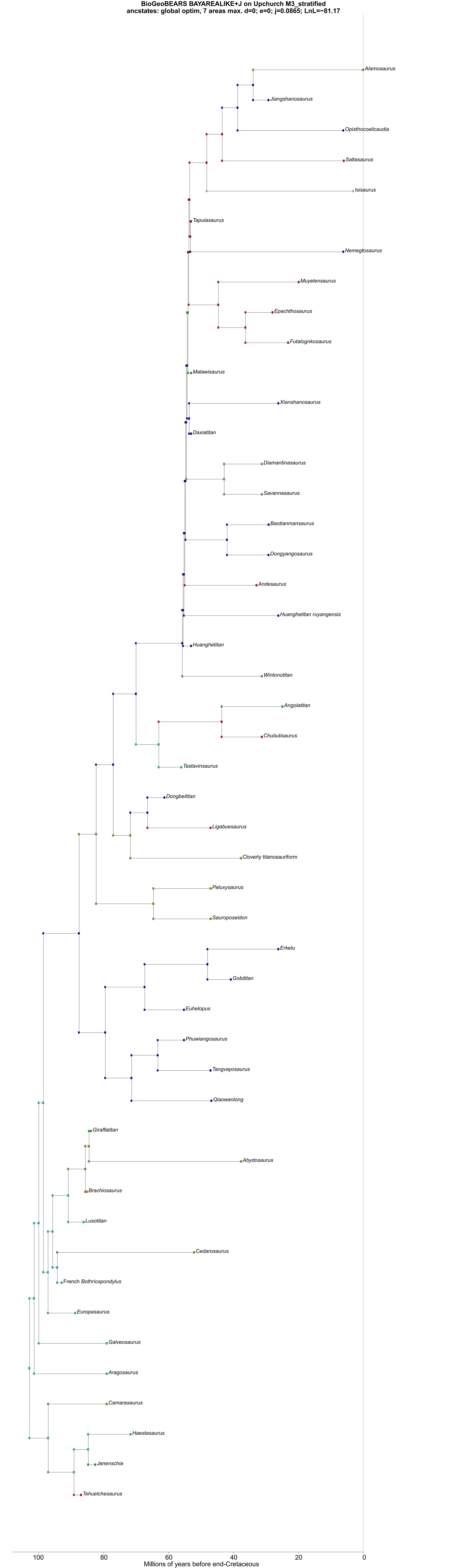
BioGeoBEARS BAYAREALIKE+J on Upchurch M3_stratified
ancstates: global optim, 7 areas max. d=0; e=0; j=0.0865; LnL=−81.17
Alamosaurus
Jiangshanosaurus
Opisthocoelicaudia
Saltasaurus
Isisaurus
Tapuiasaurus
Nemegtosaurus
Muyelensaurus
Epachthosaurus
Futalognkosaurus
Malawisaurus
Xianshanosaurus
Daxiatitan
Diamantinasaurus
Savannasaurus
Baotianmansaurus
Dongyangosaurus
Andesaurus
Huanghetitan ruyangensis
Huanghetitan
Wintonotitan
Angolatitan
Chubutisaurus
Tastavinsaurus
Dongbeititan
Ligabuesaurus
Cloverly titanosauriform
Paluxysaurus
Sauroposeidon
Erketu
Gobititan
Euhelopus
Phuwiangosaurus
Tangvayosaurus
Qiaowanlong
Giraffatitan
Abydosaurus
Brachiosaurus
Lusotitan
Cedarosaurus
French Bothriospondylus
Europasaurus
Galveosaurus
Aragosaurus
Camarasaurus
Haestasaurus
Janenschia
Tehuelchesaurus
100
80
60
40
20
0
Millions of years before end-Cretaceous

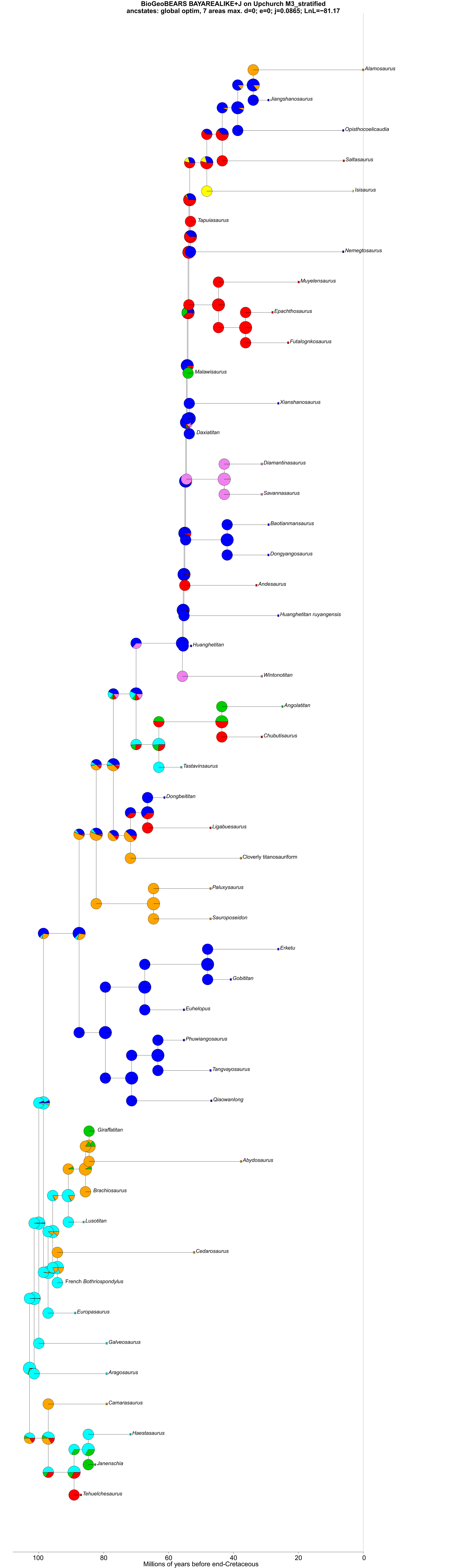
BioGeoBEARS BAYAREALIKE+J on Upchurch M3_stratified
ancstates: global optim, 7 areas max. d=0; e=0; j=0.0865; LnL=−81.17
Alamosaurus
Jiangshanosaurus
Opisthocoelicaudia
Saltasaurus
Isisaurus
Tapuiasaurus
Nemegtosaurus
Muyelensaurus
Epachthosaurus
Futalognkosaurus
Malawisaurus
Xianshanosaurus
Daxiatitan
Diamantinasaurus
Savannasaurus
Baotianmansaurus
Dongyangosaurus
Andesaurus
Huanghetitan ruyangensis
Huanghetitan
Wintonotitan
Angolatitan
Chubutisaurus
Tastavinsaurus
Dongbeititan
Ligabuesaurus
Cloverly titanosauriform
Paluxysaurus
Sauroposeidon
Erketu
Gobititan
Euhelopus
Phuwiangosaurus
Tangvayosaurus
Qiaowanlong
Giraffatitan
Abydosaurus
Brachiosaurus
Lusotitan
Cedarosaurus
French Bothriospondylus
Europasaurus
Galveosaurus
Aragosaurus
Camarasaurus
Haestasaurus
Janenschia
Tehuelchesaurus
100
80
60
40
20
0
Millions of years before end-Cretaceous

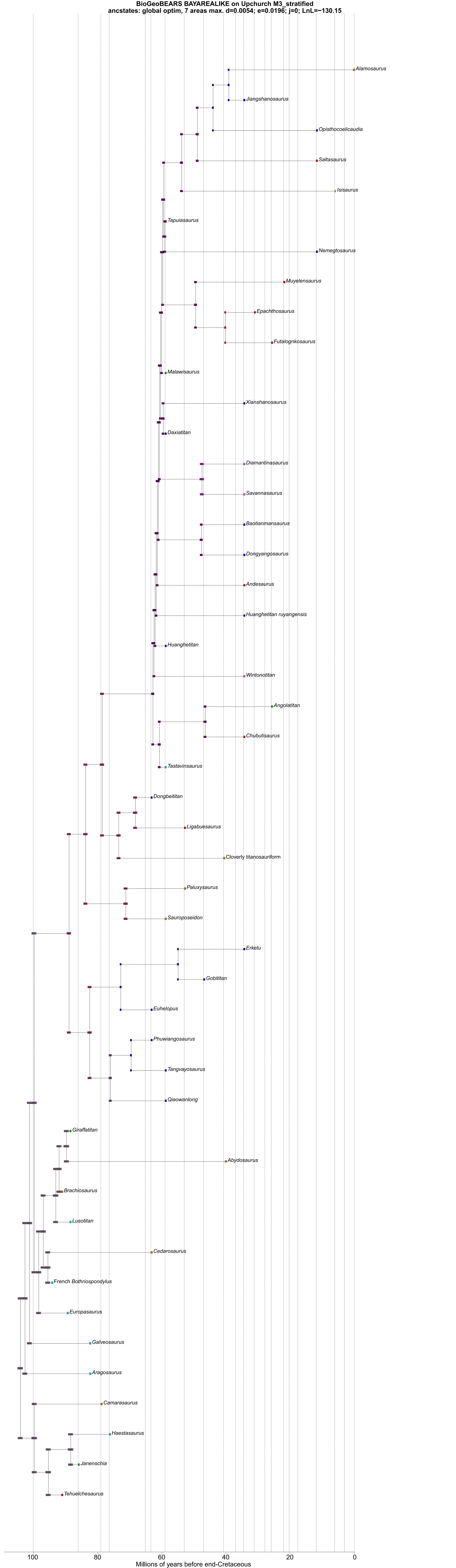
BioGeoBEARS BAYAREALIKE on Upchurch M3_stratified
ancstates: global optim, 7 areas max. d=0.0054; e=0.0196; j=0; LnL=-130.15
Alamosaurus
Jiangshanosaurus
Opisthocoelicaudia
Saltasaurus
Isisaurus
Tapuiasaurus
Nemegtosaurus
Muyelensaurus
Epachthosaurus
Futalognkosaurus
Malawisaurus
Xianshanosaurus
Daxiatitan
Diamantinasaurus
Savannasaurus
Baotianmansaurus
Dongyangosaurus
Andesaurus
Huanghetitan ruyangensis
Huanghetitan
Wintonotitan
Angolatitan
Chubutisaurus
Tastavinsaurus
Dongbeititan
Ligabuesaurus
Cloverly titanosauriform
Paluxysaurus
Sauroposeidon
Erketu
Gobititan
Euhelopus
Phuwiangosaurus
Tangvayosaurus
Qiaowanlong
Giraffatitan
Abydosaurus
Brachiosaurus
Lusotitan
Cedarosaurus
French Bothriospondylus
Europasaurus
Galveosaurus
Aragosaurus
Camarasaurus
Haestasaurus
Janenschia
Tehuelchesaurus
100
80
60
40
20
0
Millions of years before end-Cretaceous

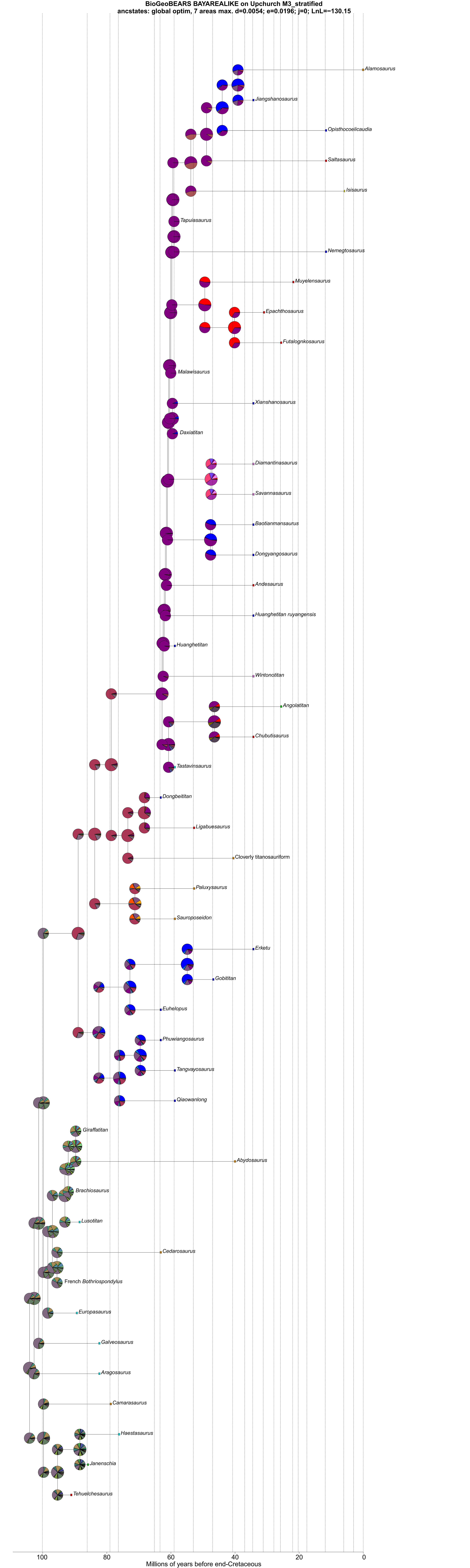
BioGeoBEARS BAYAREALIKE on Upchurch M3_stratified
ancstates: global optim, 7 areas max. d=0.0054; e=0.0196; j=0; LnL=-130.15
Alamosaurus
Jiangshanosaurus
Opisthocoelicaudia
Saltasaurus
Isisaurus
Tapuiasaurus
Nemegtosaurus
Muyelensaurus
Epachthosaurus
Futalognkosaurus
Malawisaurus
Xianshanosaurus
Daxiatitan
Diamantinasaurus
Savannasaurus
Baotianmansaurus
Dongyangosaurus
Andesaurus
Huanghetitan ruyangensis
Huanghetitan
Wintonotitan
Angolatitan
Chubutisaurus
Tastavinsaurus
Dongbeititan
Ligabuesaurus
Cloverly titanosauriform
Paluxysaurus
Sauroposeidon
Erketu
Gobititan
Euhelopus
Phuwiangosaurus
Tangvayosaurus
Qiaowanlong
Giraffatitan
Abydosaurus
Brachiosaurus
Lusotitan
Cedarosaurus
French Bothriospondylus
Europasaurus
Galveosaurus
Aragosaurus
Camarasaurus
Haestasaurus
Janenschia
Tehuelchesaurus
100
80
60
40
20
0
Millions of years before end-Cretaceous

BioGeoBEARS BAYAREALIKE+J on Upchurch M3_stratified
ancstates: global optim, 7 areas max. d=0; e=0.0146; j=0.0979; LnL=-118.63

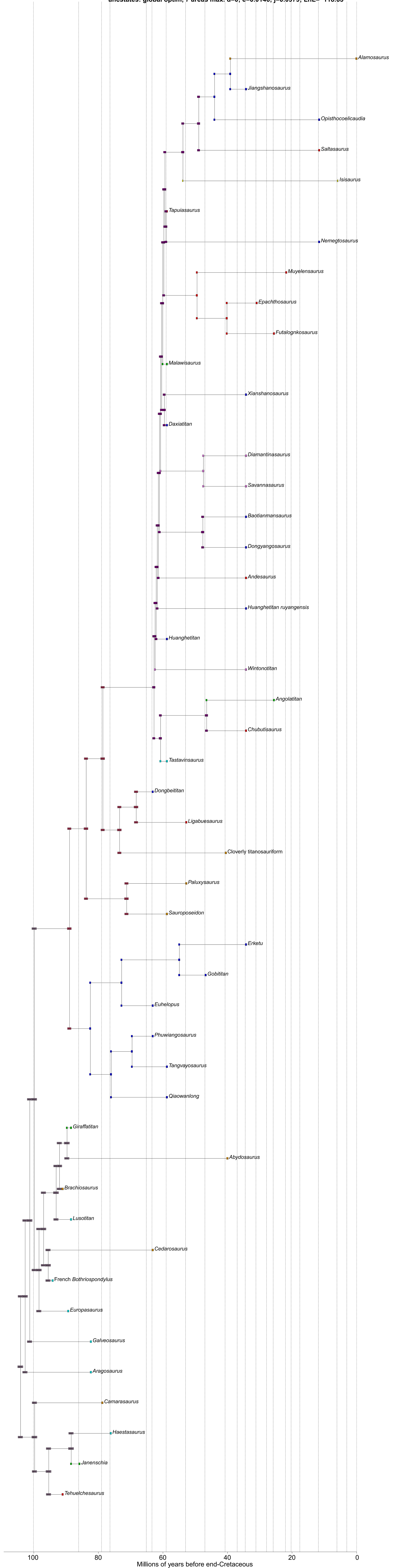

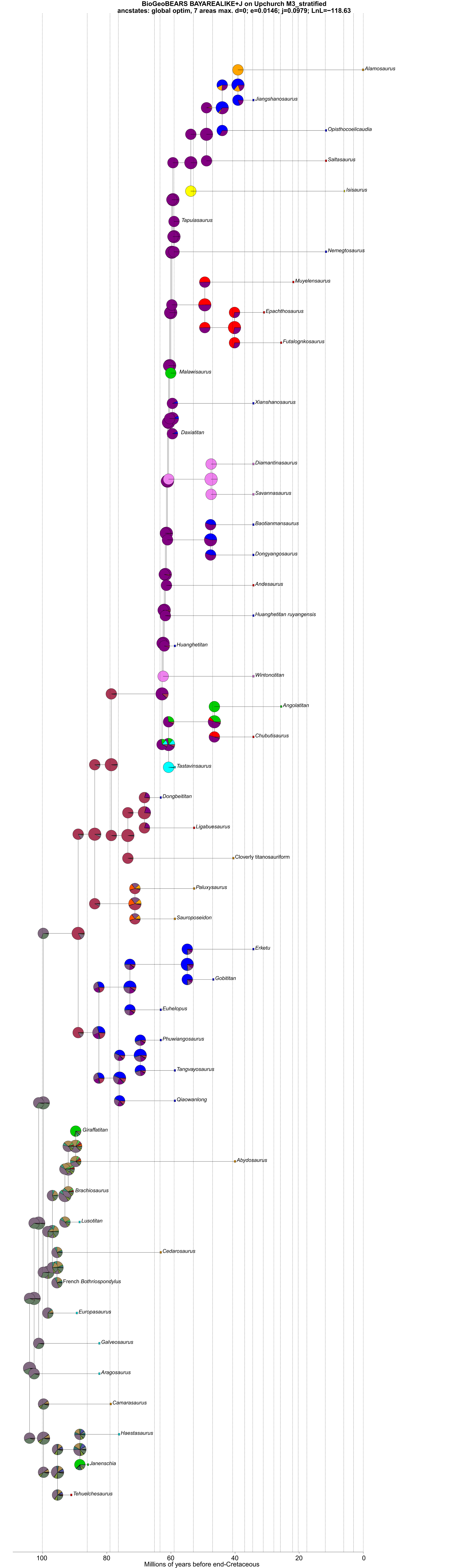
BioGeoBEARS BAYAREALIKE+J on Upchurch M3_stratified
ancstates: global optim, 7 areas max. d=0; e=0.0146; j=0.0979; LnL=-118.63
Alamosaurus
Jiangshanosaurus
Opisthocoelicaudia
Saltasaurus
Isisaurus
Tapuiasaurus
Nemegtosaurus
Muyelensaurus
Epachthosaurus
Futalognkosaurus
Malawisaurus
Xianshanosaurus
Daxiatitan
Diamantinasaurus
Savannasaurus
Baotianmansaurus
Dongyangosaurus
Andesaurus
Huanghetitan ruyangensis
Huanghetitan
Wintonotitan
Angolatitan
Chubutisaurus
Tastavinsaurus
Dongbeititan
Ligabuesaurus
Cloverly titanosauriform
Paluxysaurus
Sauroposeidon
Erketu
Gobititan
Euhelopus
Phuwiangosaurus
Tangvayosaurus
Qiaowanlong
Giraffatitan
Abydosaurus
Brachiosaurus
Lusotitan
Cedarosaurus
French Bothriospondylus
Europasaurus
Galveosaurus
Aragosaurus
Camarasaurus
Haestasaurus
Janenschia
Tehuelchesaurus
100
80
60
40
20
0
Millions of years before end-Cretaceous

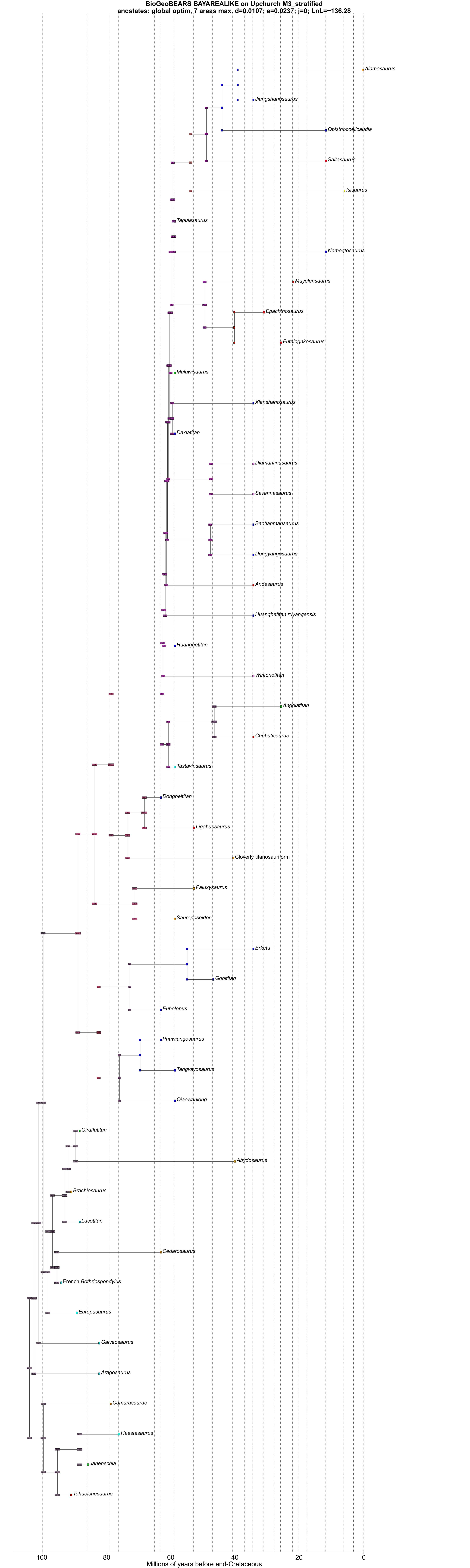
BioGeoBEARS BAYAREALIKE on Upchurch M3_stratified
ancstates: global optim, 7 areas max. d=0.0107; e=0.0237; j=0; LnL=-136.28
Alamosaurus
Jiangshanosaurus
Opisthocoelicaudia
Saltasaurus
Isisaurus
Tapuiasaurus
Nemegtosaurus
Muyelensaurus
Epachthosaurus
Futalognkosaurus
Malawisaurus
Xianshanosaurus
Daxiatitan
Diamantinasaurus
Savannasaurus
Baotianmansaurus
Dongyangosaurus
Andesaurus
Huanghetitan ruyangensis
Huanghetitan
Wintonotitan
Angolatitan
Chubutisaurus
Tastavinsaurus
Dongbeititan
Ligabuesaurus
Cloverly titanosauriform
Paluxysaurus
Sauroposeidon
Erketu
Gobititan
Euhelopus
Phuwiangosaurus
Tangvayosaurus
Qiaowanlong
Giraffatitan
Abydosaurus
Brachiosaurus
Lusotitan
Cedarosaurus
French Bothriospondylus
Europasaurus
Galveosaurus
Aragosaurus
Camarasaurus
Haestasaurus
Janenschia
Tehuelchesaurus
100
80
60
40
20
0
Millions of years before end-Cretaceous

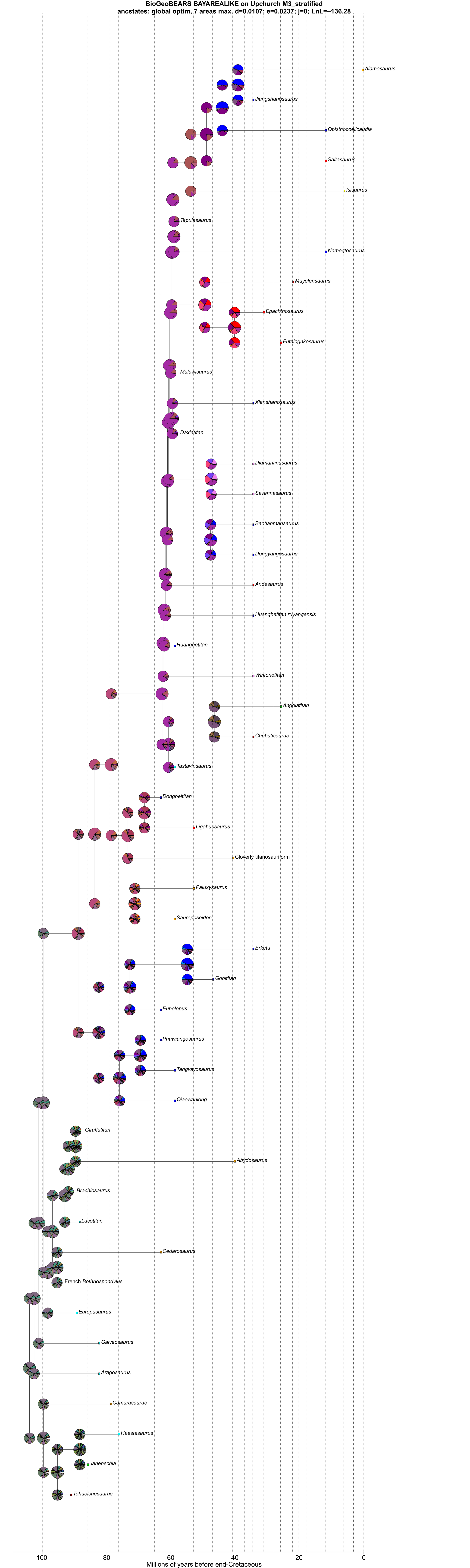
BioGeoBEARS BAYAREALIKE on Upchurch M3_stratified
ancstates: global optim, 7 areas max. d=0.0107; e=0.0237; j=0; LnL=-136.28
Alamosaurus
Jiangshanosaurus
Opisthocoelicaudia
Saltasaurus
Isisaurus
Tapuiasaurus
Nemegtosaurus
Muyelensaurus
Epachthosaurus
Futalognkosaurus
Malawisaurus
Xianshanosaurus
Daxiatitan
Diamantinasaurus
Savannasaurus
Baotianmansaurus
Dongyangosaurus
Andesaurus
Huanghetitan ruyangensis
Huanghetitan
Wintonotitan
Angolatitan
Chubutisaurus
Tastavinsaurus
Dongbeititan
Ligabuesaurus
Cloverly titanosauriform
Paluxysaurus
Sauroposeidon
Erketu
Gobititan
Euhelopus
Phuwiangosaurus
Tangvayosaurus
Qiaowanlong
Giraffatitan
Abydosaurus
Brachiosaurus
Lusotitan
Cedarosaurus
French Bothriospondylus
Europasaurus
Galveosaurus
Aragosaurus
Camarasaurus
Haestasaurus
Janenschia
Tehuelchesaurus
100
80
60
40
20
0
Millions of years before end-Cretaceous

BioGeoBEARS BAYAREALIKE+J on Upchurch M3_stratified
ancstates: global optim, 7 areas max. d=0.0075; e=0.0222; j=0.0233; LnL=-132.06

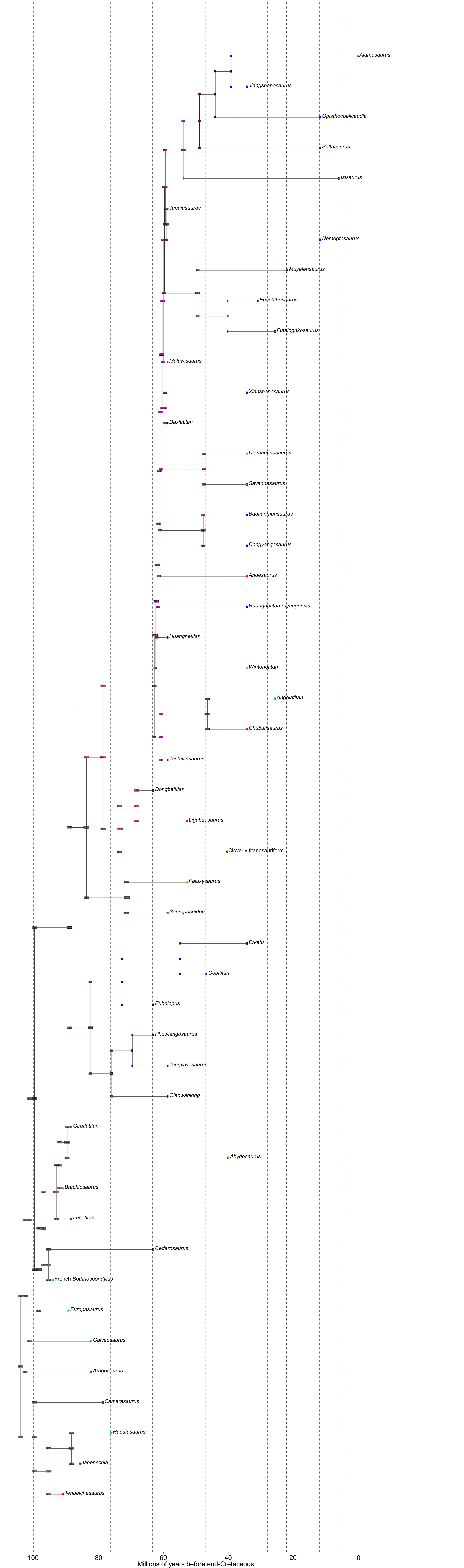

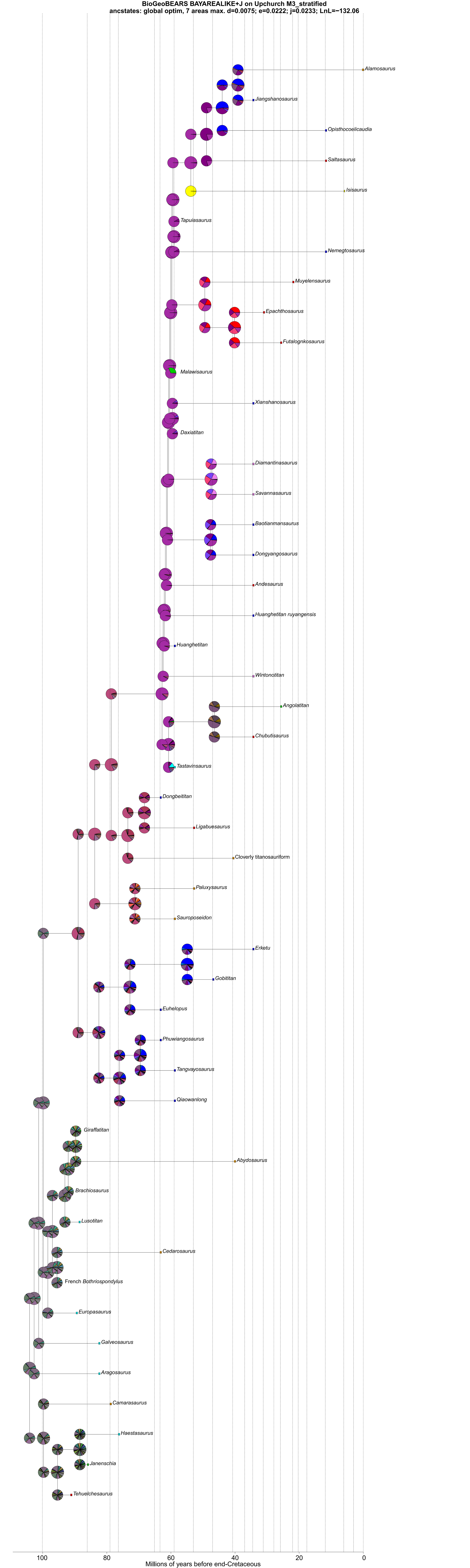
BioGeoBEARS BAYAREALIKE+J on Upchurch M3_stratified
ancstates: global optim, 7 areas max. d=0.0075; e=0.0222; j=0.0233; LnL=-132.06
Alamosaurus
Jiangshanosaurus
Opisthocoelicaudia
Saltasaurus
Isisaurus
Tapuiasaurus
Nemegtosaurus
Muyelensaurus
Epachthosaurus
Futalognkosaurus
Malawisaurus
Xianshanosaurus
Daxiatitan
Diamantinasaurus
Savannasaurus
Baotianmansaurus
Dongyangosaurus
Andesaurus
Huanghetitan ruyangensis
Huanghetitan
Wintonotitan
Angolatitan
Chubutisaurus
Tastavinsaurus
Dongbeititan
Ligabuesaurus
Cloverly titanosauriform
Paluxysaurus
Sauroposeidon
Erketu
Gobititan
Euhelopus
Phuwiangosaurus
Tangvayosaurus
Qiaowanlong
Giraffatitan
Abydosaurus
Brachiosaurus
Lusotitan
Cedarosaurus
French Bothriospondylus
Europasaurus
Galveosaurus
Aragosaurus
Camarasaurus
Haestasaurus
Janenschia
Tehuelchesaurus
100
80
60
40
20
0
Millions of years before end-Cretaceous

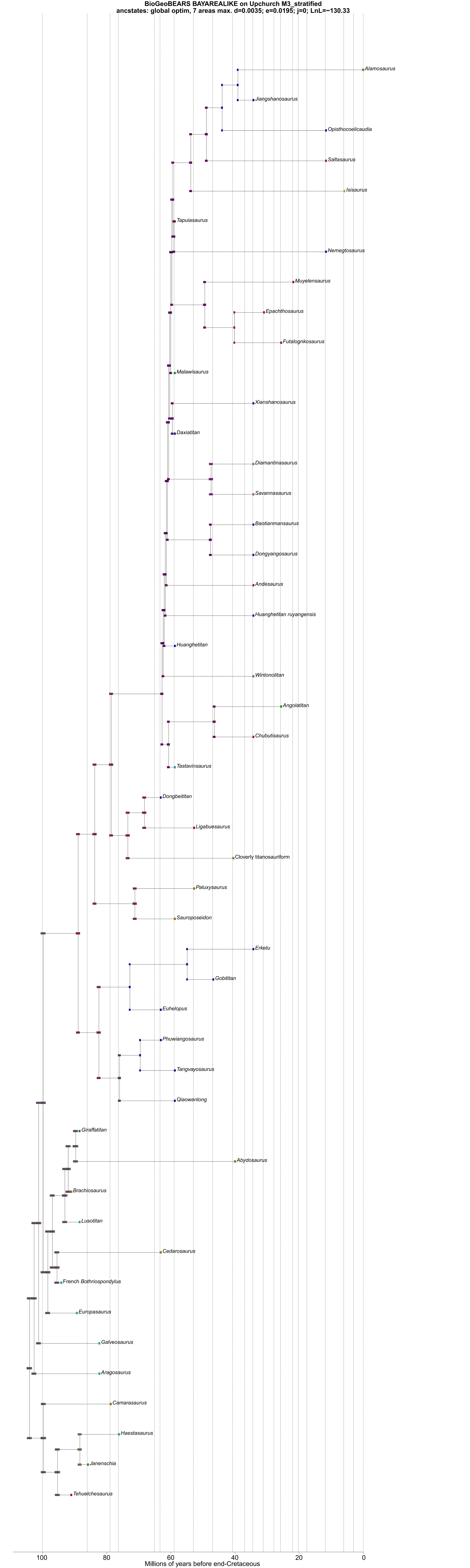
BioGeoBEARS BAYAREALIKE on Upchurch M3_stratified
ancstates: global optim, 7 areas max. d=0.0035; e=0.0195; j=0; LnL=-130.33
Alamosaurus
Jiangshanosaurus
Opisthocoelicaudia
Saltasaurus
Isisaurus
Tapuiasaurus
Nemegtosaurus
Muyelensaurus
Epachthosaurus
Futalognkosaurus
Malawisaurus
Xianshanosaurus
Daxiatitan
Diamantinasaurus
Savannasaurus
Baotianmansaurus
Dongyangosaurus
Andesaurus
Huanghetitan ruyangensis
Huanghetitan
Wintonotitan
Angolatitan
Chubutisaurus
Tastavinsaurus
Dongbeititan
Ligabuesaurus
Cloverly titanosauriform
Paluxysaurus
Sauroposeidon
Erketu
Gobititan
Euhelopus
Phuwiangosaurus
Tangvayosaurus
Qiaowanlong
Giraffatitan
Abydosaurus
Brachiosaurus
Lusotitan
Cedarosaurus
French Bothriospondylus
Europasaurus
Galveosaurus
Aragosaurus
Camarasaurus
Haestasaurus
Janenschia
Tehuelchesaurus
100
80
60
40
20
0
Millions of years before end-Cretaceous

BioGeoBEARS BAYAREALIKE on Upchurch M3_stratified
ancstates: global optim, 7 areas max. d=0.0035; e=0.0195; j=0; LnL=-130.33

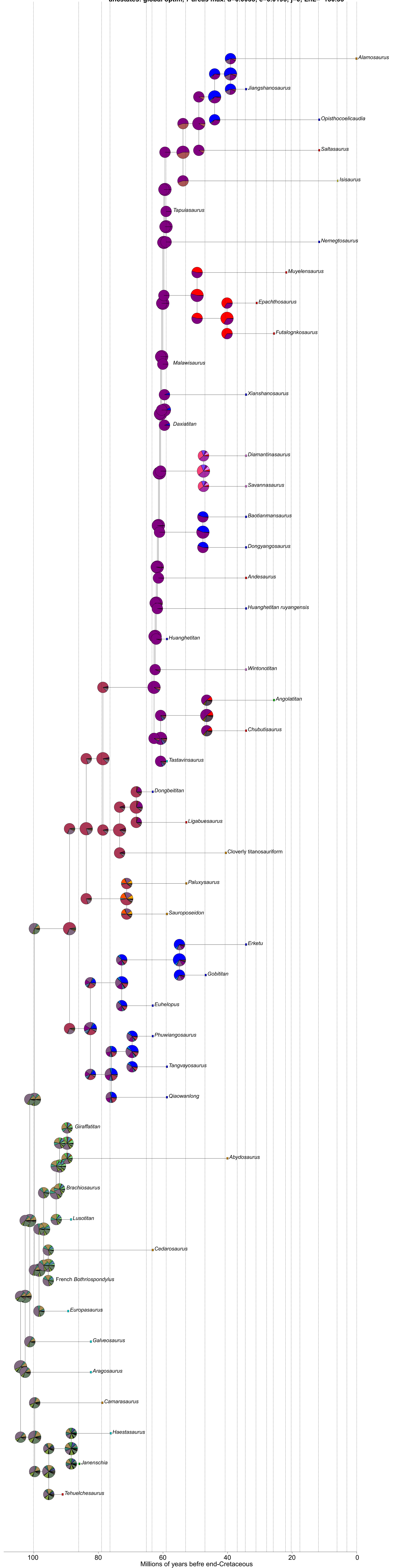

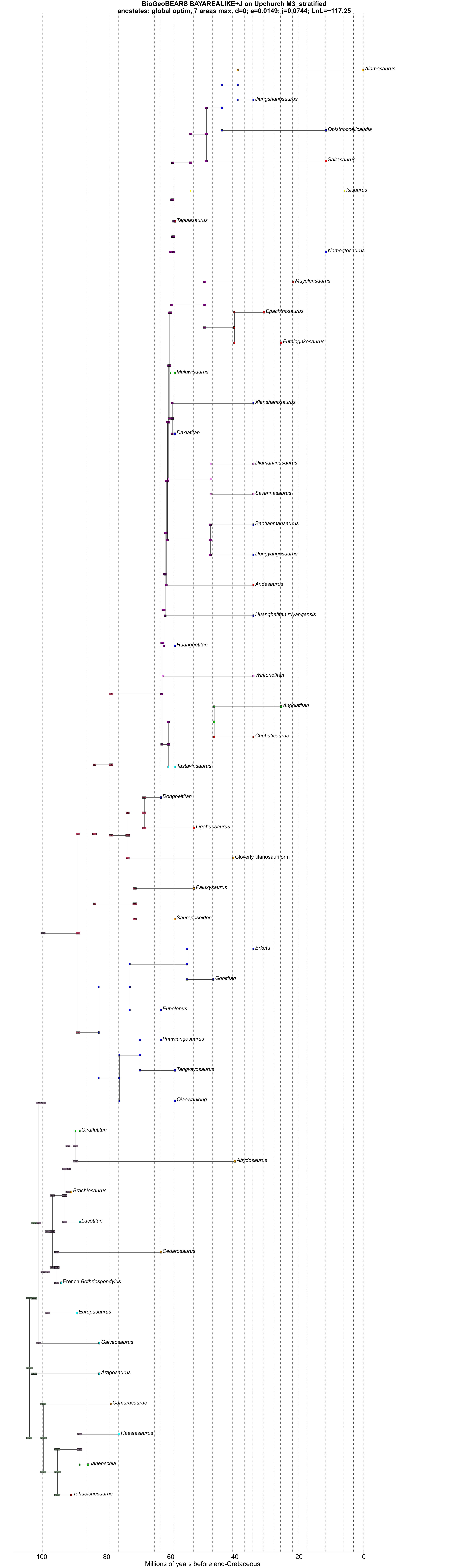
BioGeoBEARS BAYAREALIKE+J on Upchurch M3_stratified
ancstates: global optim, 7 areas max. d=0; e=0.0149; j=0.0744; LnL=-117.25
Alamosaurus
Jiangshanosaurus
Opisthocoelicaudia
Saltasaurus
Isisaurus
Tapuiasaurus
Nemegtosaurus
Muyelensaurus
Epachthosaurus
Futalognkosaurus
Malawisaurus
Xianshanosaurus
Daxiatitan
Diamantinasaurus
Savannasaurus
Baotianmansaurus
Dongyangosaurus
Andesaurus
Huanghetitan ruyangensis
Huanghetitan
Wintonotitan
Angolatitan
Chubutisaurus
Tastavinsaurus
Dongbeititan
Ligabuesaurus
Cloverly titanosauriform
Paluxysaurus
Sauroposeidon
Erketu
Gobititan
Euhelopus
Phuwiangosaurus
Tangvayosaurus
Qiaowanlong
Giraffatitan
Abydosaurus
Brachiosaurus
Lusotitan
Cedarosaurus
French Bothriospondylus
Europasaurus
Galveosaurus
Aragosaurus
Camarasaurus
Haestasaurus
Janenschia
Tehuelchesaurus
100
80
60
40
20
0
Millions of years before end-Cretaceous

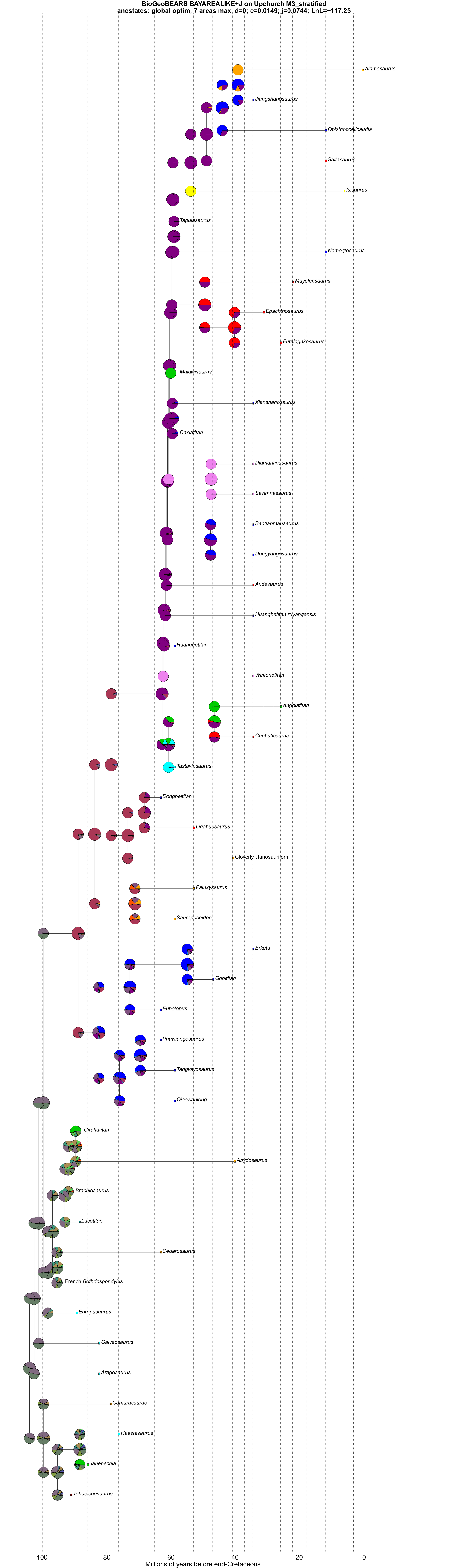
BioGeoBEARS BAYAREALIKE+J on Upchurch M3_stratified
ancstates: global optim, 7 areas max. d=0; e=0.0149; j=0.0744; LnL=-117.25
Alamosaurus
Jiangshanosaurus
Opisthocoelicaudia
Saltasaurus
Isisaurus
Tapuiasaurus
Nemegtosaurus
Muyelensaurus
Epachthosaurus
Futalognkosaurus
Malawisaurus
Xianshanosaurus
Daxiatitan
Diamantinasaurus
Savannasaurus
Baotianmansaurus
Dongyangosaurus
Andesaurus
Huanghetitan ruyangensis
Huanghetitan
Wintonotitan
Angolatitan
Chubutisaurus
Tastavinsaurus
Dongbeititan
Ligabuesaurus
Cloverly titanosauriform
Paluxysaurus
Sauroposeidon
Erketu
Gobititan
Euhelopus
Phuwiangosaurus
Tangvayosaurus
Qiaowanlong
Giraffatitan
Abydosaurus
Brachiosaurus
Lusotitan
Cedarosaurus
French Bothriospondylus
Europasaurus
Galveosaurus
Aragosaurus
Camarasaurus
Haestasaurus
Janenschia
Tehuelchesaurus
100
80
60
40
20
0
Millions of years before end-Cretaceous

BioGeoBEARS DEC on Upchurch M3_stratified
ancstates: global optim, 7 areas max. d=0.0083; e=0.0041; j=0; LnL=-136.91

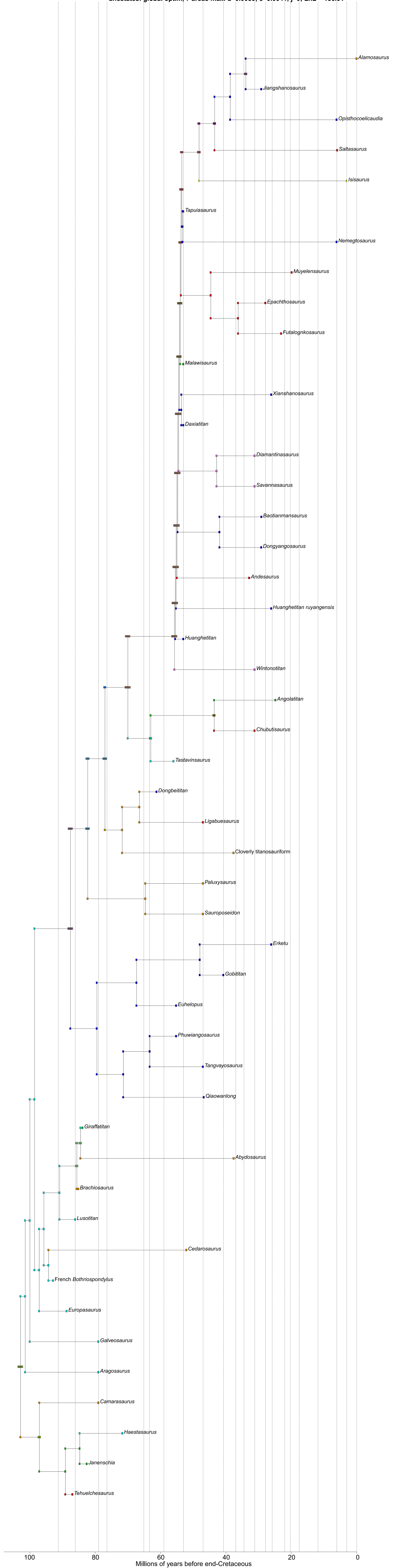

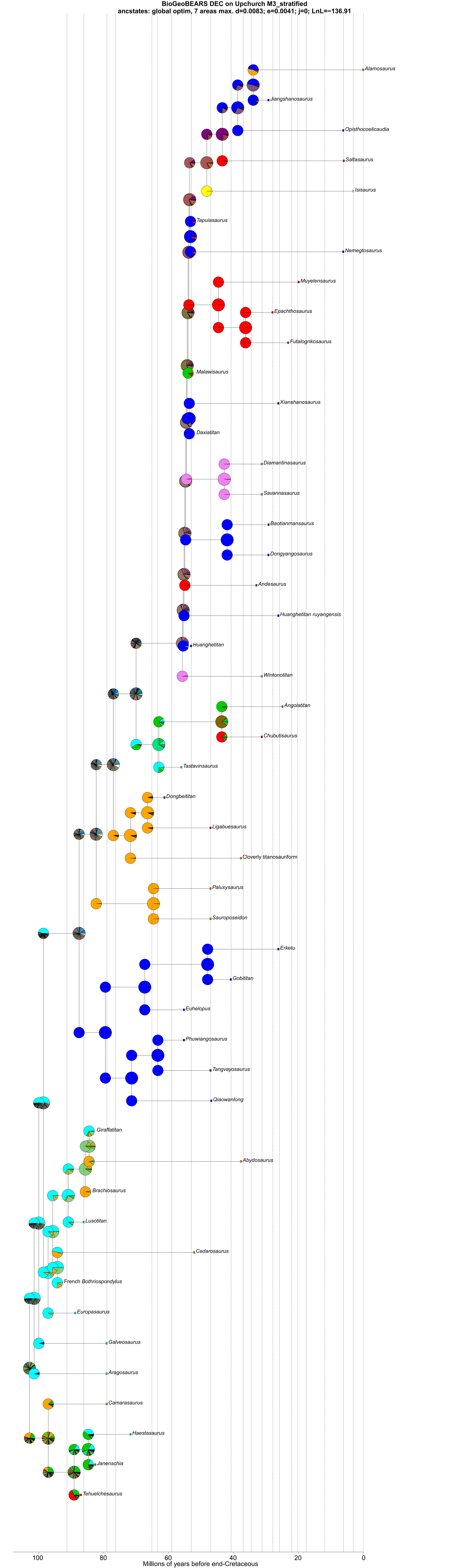
BioGeoBEARS DEC on Upchurch M3_stratified
ancstates: global optim, 7 areas max. d=0.0083; e=0.0041; j=0; LnL=-136.91
Alamosaurus
Jiangshanosaurus
Opisthocoelicaudia
Saltasaurus
Isisaurus
Tapuiasaurus
Nemegtosaurus
Muyelensaurus
Epachthosaurus
Futalognkosaurus
Malawisaurus
Xianshanosaurus
Daxiatitan
Diamantinasaurus
Savannasaurus
Baotianmansaurus
Dongyangosaurus
Andesaurus
Huanghetitan ruyangensis
Huanghetitan
Wintonotitan
Angolatitan
Chubutisaurus
Tastavinsaurus
Dongbeititan
Ligabuesaurus
Cloverly titanosauriform
Paluxysaurus
Sauroposeidon
Erketu
Gobititan
Euhelopus
Phuwiangosaurus
Tangvayosaurus
Qiaowanlong
Giraffatitan
Abydosaurus
Brachiosaurus
Lusotitan
Cedarosaurus
French Bothriospondylus
Europasaurus
Galveosaurus
Aragosaurus
Camarasaurus
Haestasaurus
Janenschia
Tehuelchesaurus
100
80
60
40
20
0
Millions of years before end-Cretaceous

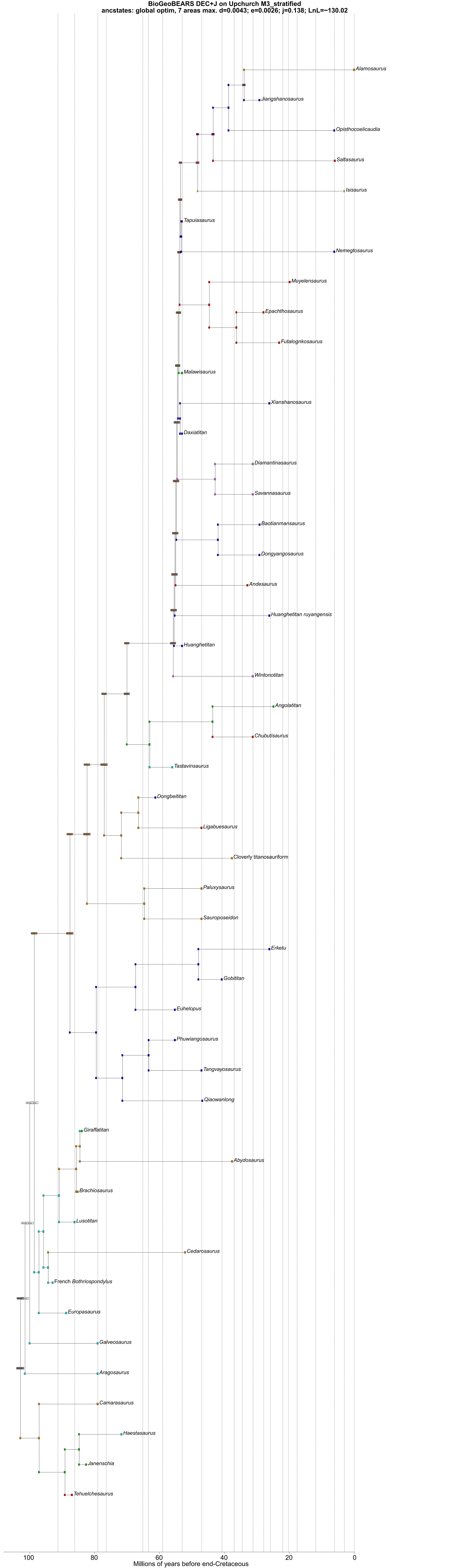
BioGeoBEARS DEC+J on Upchurch M3_stratified
ancstates: global optim, 7 areas max. d=0.0043; e=0.0026; j=0.138; LnL=−130.02
Alamosaurus
Jiangshanosaurus
Opisthocoelicaudia
Saltasaurus
Isisaurus
Tapuiasaurus
Nemegtosaurus
Muyelensaurus
Epachthosaurus
Futalognkosaurus
Malawisaurus
Xianshanosaurus
Daxiatitan
Diamantinasaurus
Savannasaurus
Baotianmansaurus
Dongyangosaurus
Andesaurus
Huanghetitan ruyangensis
Huanghetitan
Wintonotitan
Angolatitan
Chubutisaurus
Tastavinsaurus
Dongbeititan
Ligabuesaurus
Cloverly titanosauriform
Paluxysaurus
Sauroposeidon
Erketu
Gobititan
Euhelopus
Phuwiangosaurus
Tangvayosaurus
Qiaowanlong
Giraffatitan
Abydosaurus
Brachiosaurus
Lusotitan
Cedarosaurus
French Bothriospondylus
Europasaurus
Galveosaurus
Aragosaurus
Camarasaurus
Haestasaurus
Janenschia
Tehuelchesaurus
100
80
60
40
20
0
Millions of years before end-Cretaceous

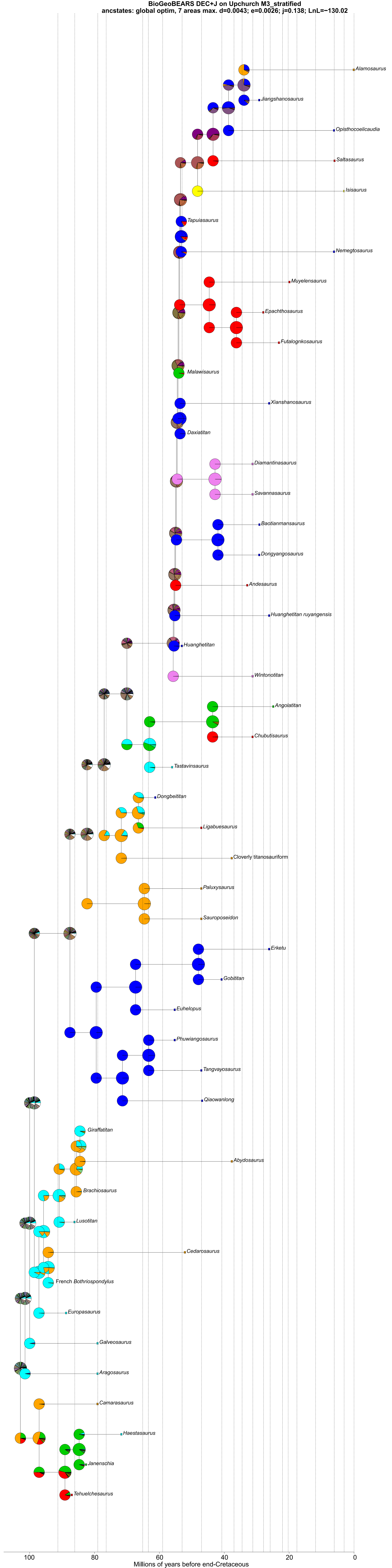
BioGeoBEARS DEC+J on Upchurch M3_stratified
ancstates: global optim, 7 areas max. d=0.0043; e=0.0026; j=0.138; LnL=-130.02
Alamosaurus
Jiangshanosaurus
Opisthocoelicaudia
Saltasaurus
Isisaurus
Tapuiasaurus
Nemegtosaurus
Muyelensaurus
Epachthosaurus
Futalognkosaurus
Malawisaurus
Xianshanosaurus
Daxiatitan
Diamantinasaurus
Savannasaurus
Baotianmansaurus
Dongyangosaurus
Andesaurus
Huanghetitan ruyangensis
Huanghetitan
Wintonotitan
Angolatitan
Chubutisaurus
Tastavinsaurus
Dongbeititan
Ligabuesaurus
Cloverly titanosauriform
Paluxysaurus
Sauroposeidon
Erketu
Gobititan
Euhelopus
Phuwiangosaurus
Tangvayosaurus
Qiaowanlong
Giraffatitan
Abydosaurus
Brachiosaurus
Lusotitan
Cedarosaurus
French Bothriospondylus
Europasaurus
Galveosaurus
Aragosaurus
Camarasaurus
Haestasaurus
Janenschia
Tehuelchesaurus
100
80
60
40
20
0
Millions of years before end-Cretaceous

BioGeoBEARS BAYAREALIKE on Upchurch M3_stratified
ancstates: global optim, 7 areas max. d=0.0053; e=0.0199; j=0; LnL=-131.87

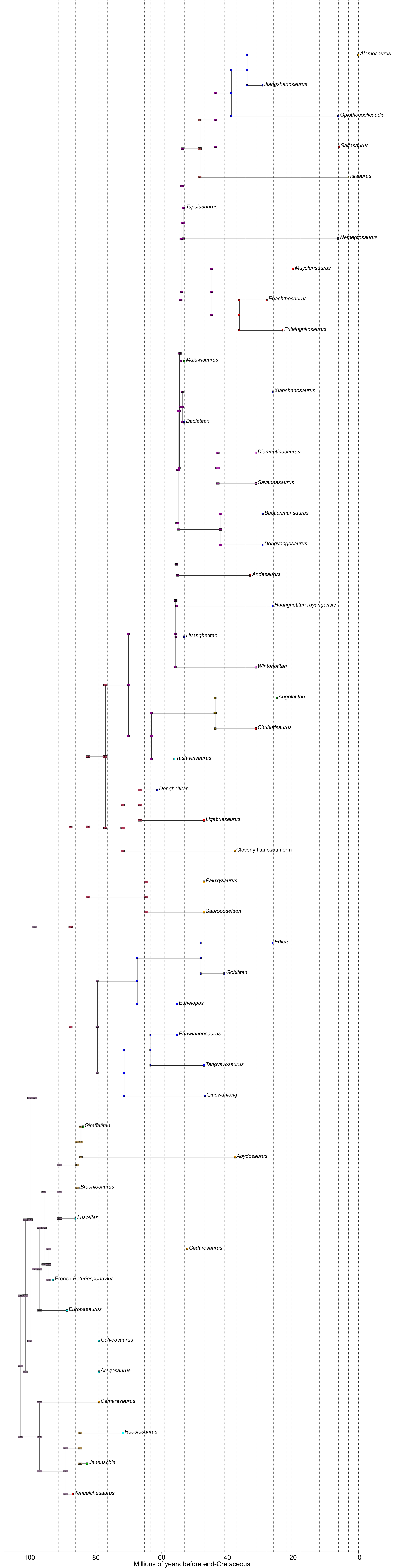

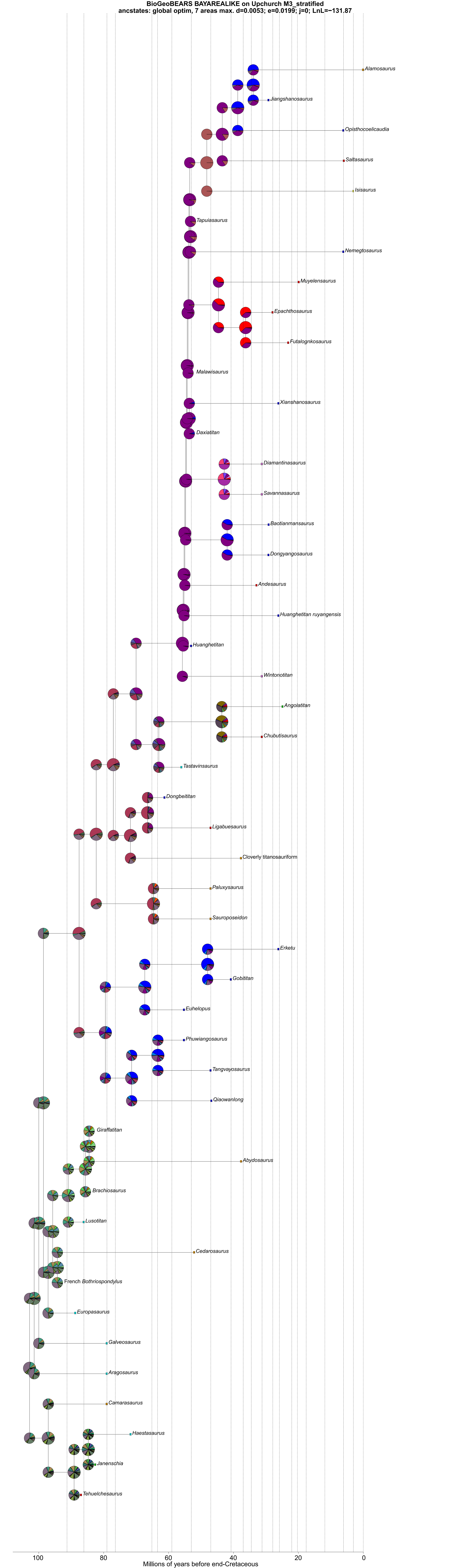
BioGeoBEARS BAYAREALIKE on Upchurch M3_stratified
ancstates: global optim, 7 areas max. d=0.0053; e=0.0199; j=0; LnL=-131.87
Alamosaurus
Jiangshanosaurus
Opisthocoelicaudia
Saltasaurus
Isisaurus
Tapuiasaurus
Nemegtosaurus
Muyelensaurus
Epachthosaurus
Futalognkosaurus
Malawisaurus
Xianshanosaurus
Daxiatitan
Diamantinasaurus
Savannasaurus
Baotianmansaurus
Dongyangosaurus
Andesaurus
Huanghetitan ruyangensis
Huanghetitan
Wintonotitan
Angolatitan
Chubutisaurus
Tastavinsaurus
Dongbeititan
Ligabuesaurus
Cloverly titanosauriform
Paluxysaurus
Sauroposeidon
Erketu
Gobititan
Euhelopus
Phuwiangosaurus
Tangvayosaurus
Qiaowanlong
Giraffatitan
Abydosaurus
Brachiosaurus
Lusotitan
Cedarosaurus
French Bothriospondylus
Europasaurus
Galveosaurus
Aragosaurus
Camarasaurus
Haestasaurus
Janenschia
Tehuelchesaurus
100
80
60
40
20
0
Millions of years before end-Cretaceous

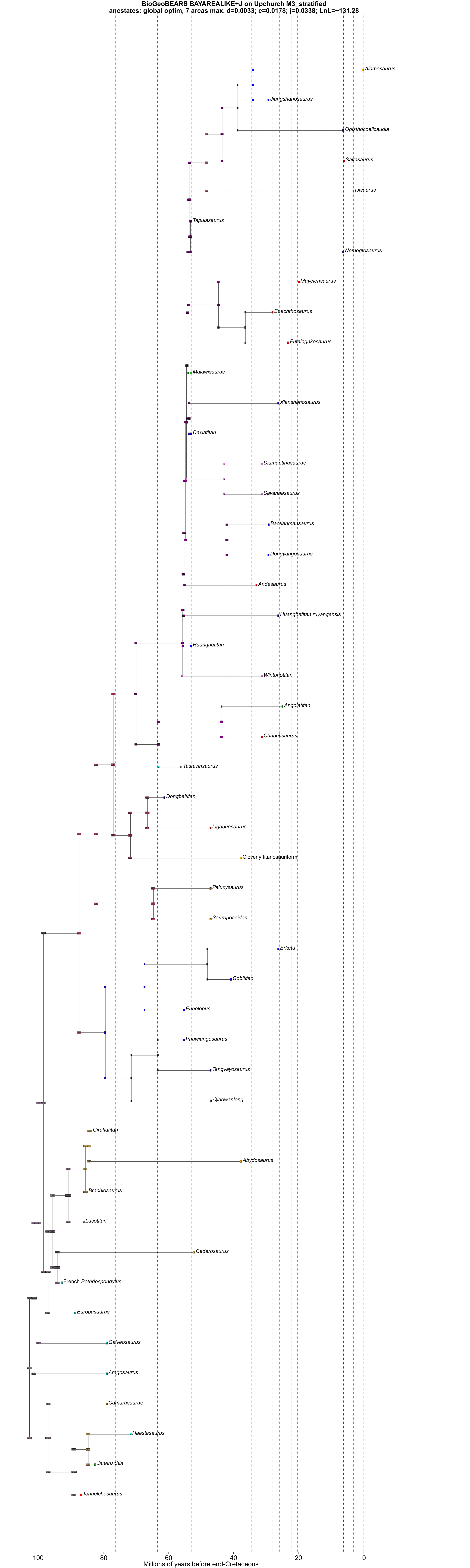
BioGeoBEARS BAYAREALIKE+J on Upchurch M3_stratified
ancstates: global optim, 7 areas max. d=0.0033; e=0.0178; j=0.0338; LnL=-131.28
Alamosaurus
Jiangshanosaurus
Opisthocoelicaudia
Saltasaurus
Isisaurus
Tapuiasaurus
Nemegtosaurus
Muyelensaurus
Epachthosaurus
Futalognkosaurus
Malawisaurus
Xianshanosaurus
Daxiatitan
Diamantinasaurus
Savannasaurus
Baotianmansaurus
Dongyangosaurus
Andesaurus
Huanghetitan ruyangensis
Huanghetitan
Wintonotitan
Angolatitan
Chubutisaurus
Tastavinsaurus
Dongbeititan
Ligabuesaurus
Cloverly titanosauriform
Paluxysaurus
Sauroposeidon
Erketu
Gobititan
Euhelopus
Phuwiangosaurus
Tangvayosaurus
Qiaowanlong
Giraffatitan
Abydosaurus
Brachiosaurus
Lusotitan
Cedarosaurus
French Bothriospondylus
Europasaurus
Galveosaurus
Aragosaurus
Camarasaurus
Haestasaurus
Janenschia
Tehuelchesaurus
100
80
60
40
20
0
Millions of years before end-Cretaceous

BioGeoBEARS BAYAREALIKE+J on Upchurch M3_stratified
ancstates: global optim, 7 areas max. d=0.0033; e=0.0178; j=0.0338; LnL=-131.28

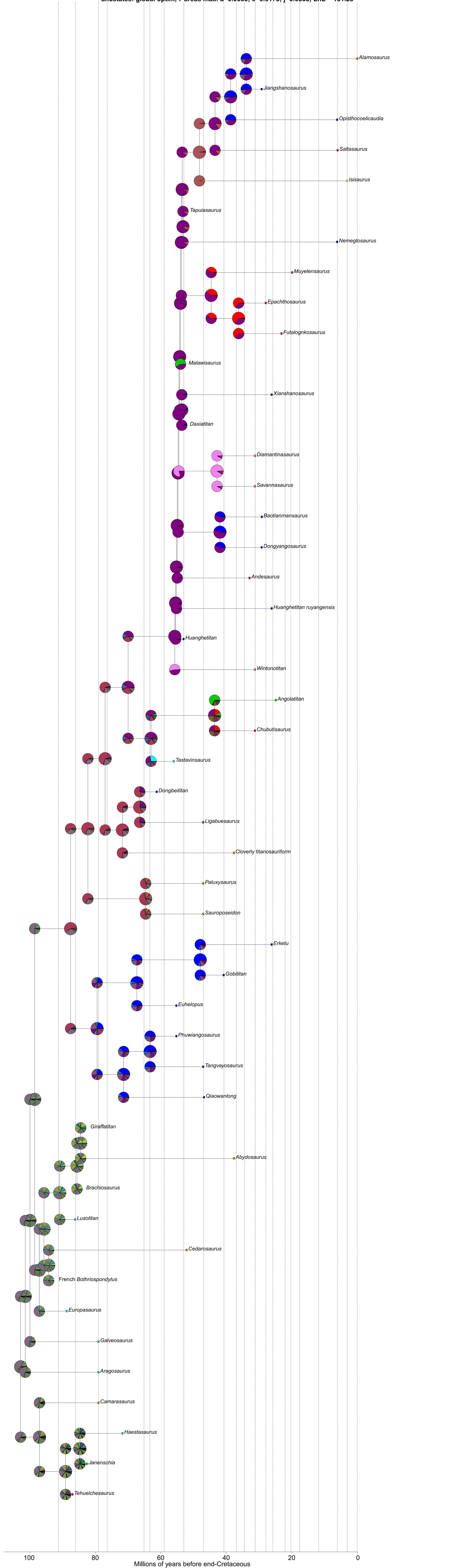

BioGeoBEARS BAYAREALIKE on Upchurch M3_stratified
ancstates: global optim, 7 areas max. d=0.0108; e=0.0239; j=0; LnL=-135.97

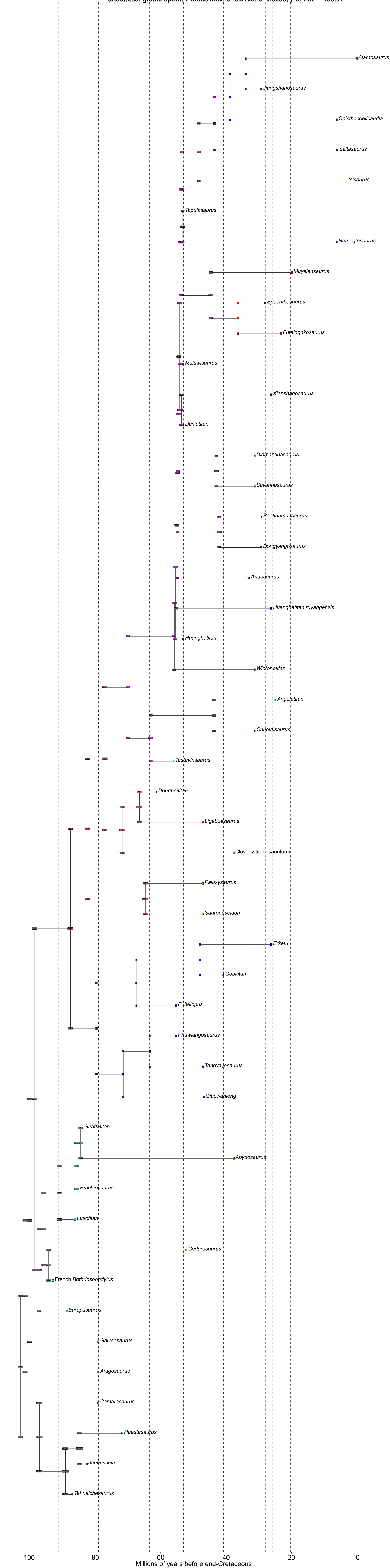

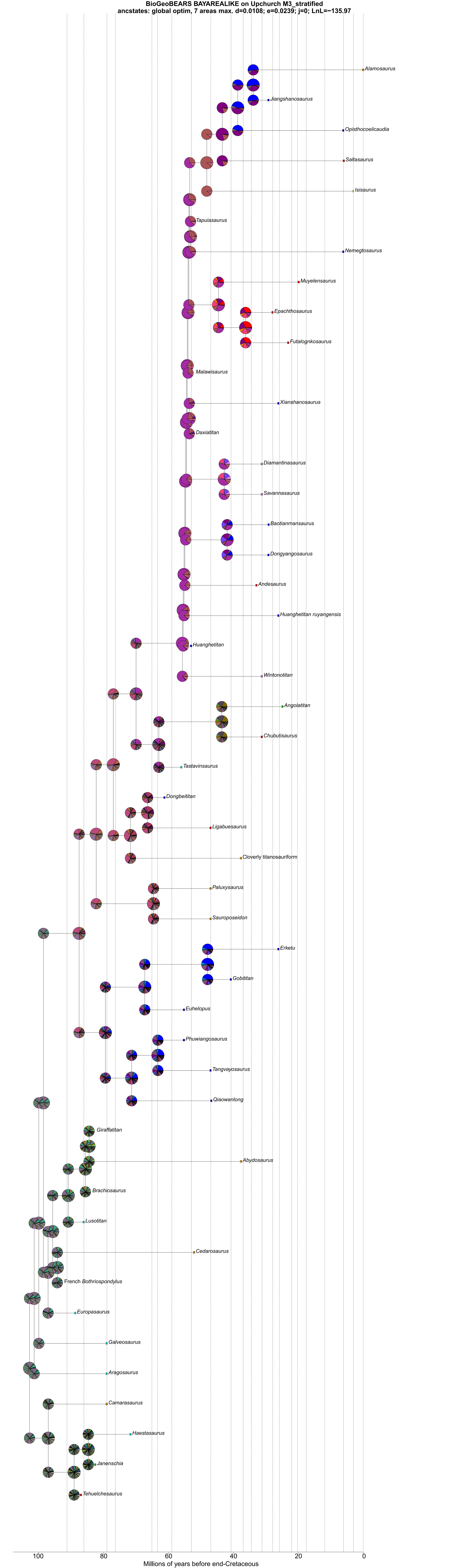
BioGeoBEARS BAYAREALIKE on Upchurch M3_stratified
ancstates: global optim, 7 areas max. d=0.0108; e=0.0239; j=0; LnL=-135.97
Alamosaurus
Jiangshanosaurus
Opisthocoelicaudia
Saltasaurus
Isisaurus
Tapuiasaurus
Nemegtosaurus
Muyelensaurus
Epachthosaurus
Futalognkosaurus
Malawisaurus
Xianshanosaurus
Daxiatitan
Diamantinasaurus
Savannasaurus
Baotianmansaurus
Dongyangosaurus
Andesaurus
Huanghetitan ruyangensis
Huanghetitan
Wintonotitan
Angolatitan
Chubutisaurus
Tastavinsaurus
Dongbeititan
Ligabuesaurus
Cloverly titanosauriform
Paluxysaurus
Sauroposeidon
Erketu
Gobititan
Euhelopus
Phuwiangosaurus
Tangvayosaurus
Qiaowanlong
Giraffatitan
Abydosaurus
Brachiosaurus
Lusotitan
Cedarosaurus
French Bothriospondylus
Europasaurus
Galveosaurus
Aragosaurus
Camarasaurus
Haestasaurus
Janenschia
Tehuelchesaurus
100
80
60
40
20
0
Millions of years before end-Cretaceous

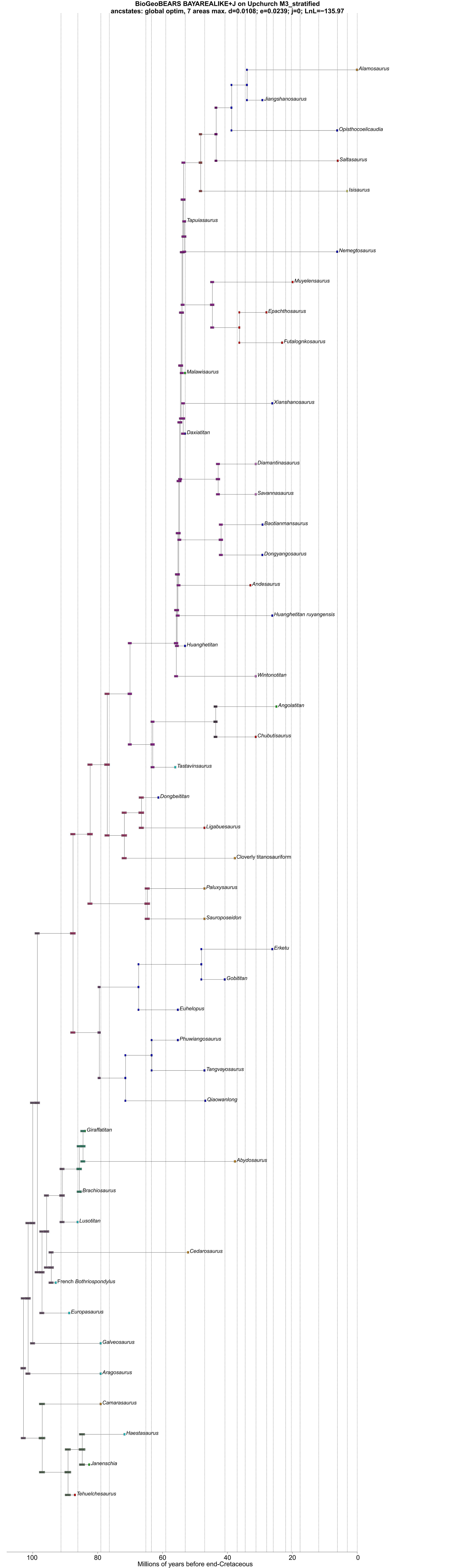
BioGeoBEARS BAYAREALIKE+J on Upchurch M3_stratified
ancstates: global optim, 7 areas max. d=0.0108; e=0.0239; j=0; LnL=-135.97
Alamosaurus
Jiangshanosaurus
Opisthocoelicaudia
Saltasaurus
Isisaurus
Tapuiasaurus
Nemegtosaurus
Muyelensaurus
Epachthosaurus
Futalognkosaurus
Malawisaurus
Xianshanosaurus
Daxiatitan
Diamantinasaurus
Savannasaurus
Baotianmansaurus
Dongyangosaurus
Andesaurus
Huanghetitan ruyangensis
Huanghetitan
Wintonotitan
Angolatitan
Chubutisaurus
Tastavinsaurus
Dongbeititan
Ligabuesaurus
Cloverly titanosauriform
Paluxysaurus
Sauroposeidon
Erketu
Gobititan
Euhelopus
Phuwiangosaurus
Tangvayosaurus
Qiaowanlong
Giraffatitan
Abydosaurus
Brachiosaurus
Lusotitan
Cedarosaurus
French Bothriospondylus
Europasaurus
Galveosaurus
Aragosaurus
Camarasaurus
Haestasaurus
Janenschia
Tehuelchesaurus
100
80
60
40
20
0
Millions of years before end-Cretaceous

BioGeoBEARS BAYAREALIKE+J on Upchurch M3_stratified
ancstates: global optim, 7 areas max. d=0.0108; e=0.0239; j=0; LnL=−135.97

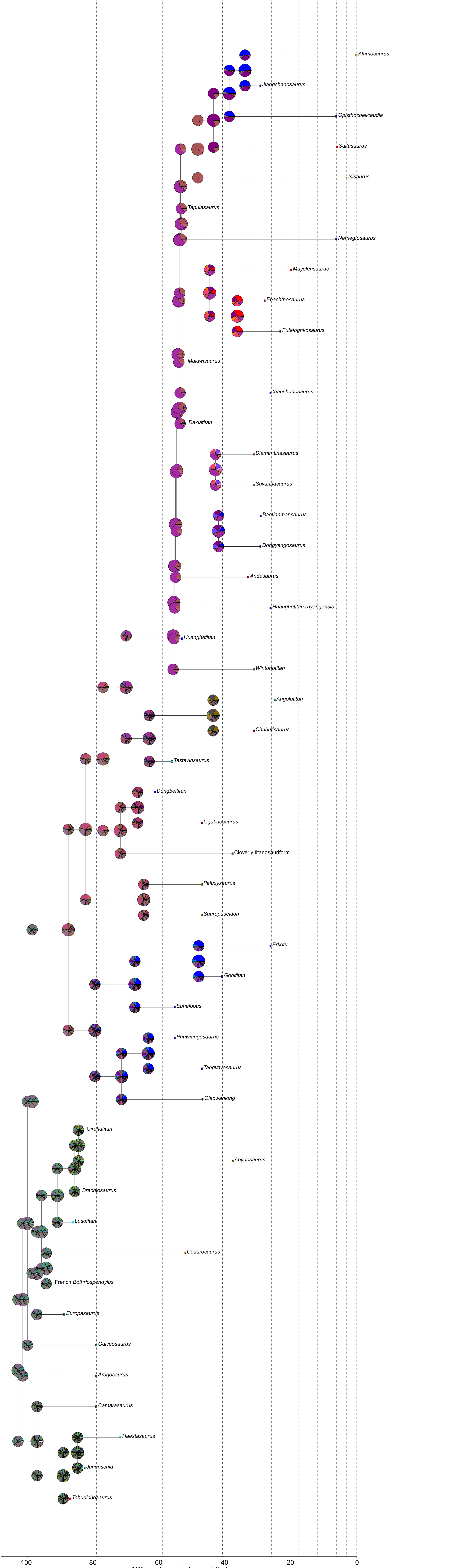

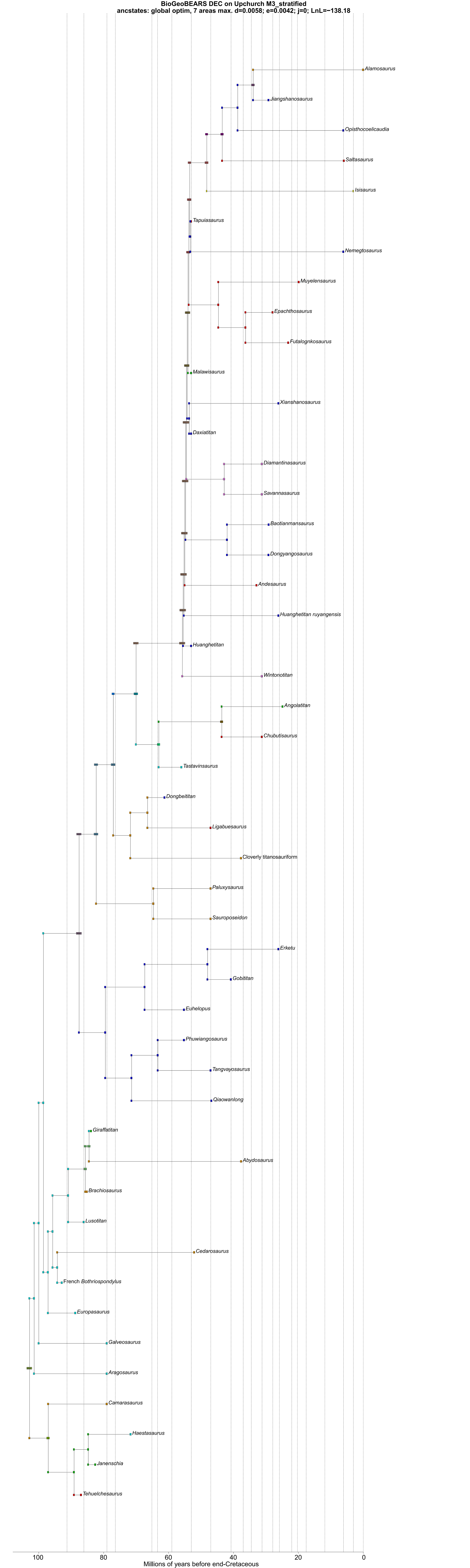
BioGeoBEARS DEC on Upchurch M3_stratified
ancstates: global optim, 7 areas max. d=0.0058; e=0.0042; j=0; LnL=−138.18
Alamosaurus
Jiangshanosaurus
Opisthocoelicaudia
Saltasaurus
Isisaurus
Tapuiasaurus
Nemegtosaurus
Muyelensaurus
Epachthosaurus
Futalognkosaurus
Malawisaurus
Xianshanosaurus
Daxiatitan
Diamantinasaurus
Savannasaurus
Baotianmansaurus
Dongyangosaurus
Andesaurus
Huanghetitan ruyangensis
Huanghetitan
Wintonotitan
Angolatitan
Chubutisaurus
Tastavinsaurus
Dongbeititan
Ligabuesaurus
Cloverly titanosauriform
Paluxysaurus
Sauroposeidon
Erketu
Gobititan
Euhelopus
Phuwiangosaurus
Tangvayosaurus
Qiaowanlong
Giraffatitan
Abydosaurus
Brachiosaurus
Lusotitan
Cedarosaurus
French Bothriospondylus
Europasaurus
Galveosaurus
Aragosaurus
Camarasaurus
Haestasaurus
Janenschia
Tehuelchesaurus
100
80
60
40
20
0
Millions of years before end-Cretaceous

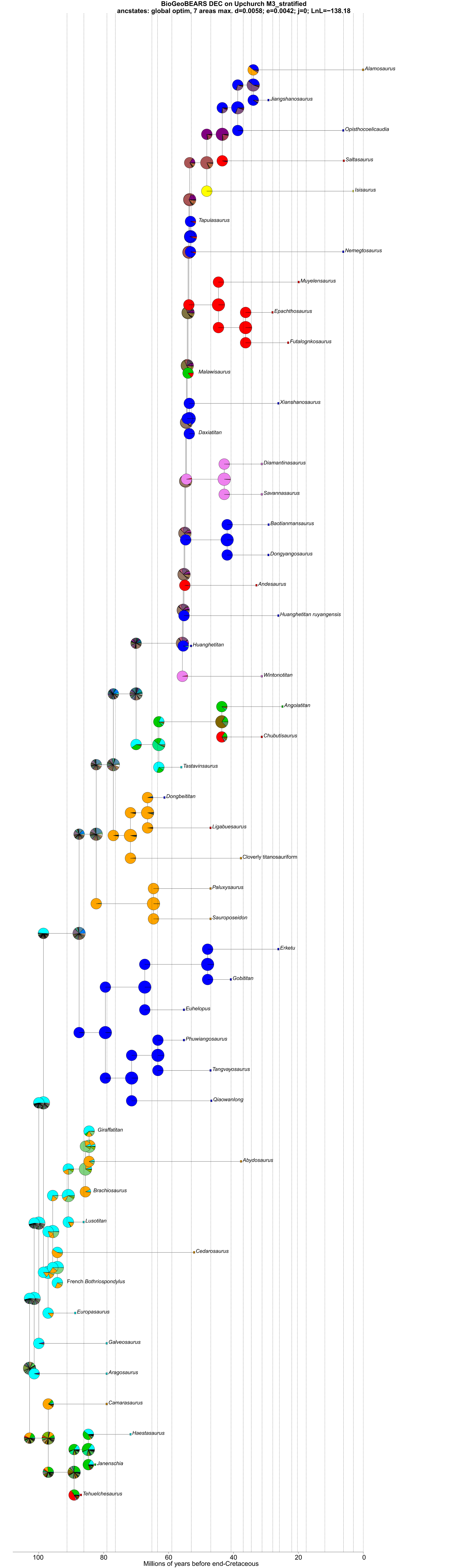

BioGeoBEARS DEC on Upchurch M3_stratified
ancstates: global optim, 7 areas max. d=0.0058; e=0.0042; j=0; LnL=-138.18
Alamosaurus
Jiangshanosaurus
Opisthocoelicaudia
Saltasaurus
Isisaurus
Tapuiasaurus
Nemegtosaurus
Muyelensaurus
Epachthosaurus
Futalognkosaurus
Malawisaurus
Xianshanosaurus
Daxiatitan
Diamantinasaurus
Savannasaurus
Baotianmansaurus
Dongyangosaurus
Andesaurus
Huanghetitan ruyangensis
Huanghetitan
Wintonotitan
Angolatitan
Chubutisaurus
Tastavinsaurus
Dongbeititan
Ligabuesaurus
Cloverly titanosauriform
Paluxysaurus
Sauroposeidon
Erketu
Gobititan
Euhelopus
Phuwiangosaurus
Tangvayosaurus
Qiaowanlong
Giraffatitan
Abydosaurus
Brachiosaurus
Lusotitan
Cedarosaurus
French Bothriospondylus
Europasaurus
Galveosaurus
Aragosaurus
Camarasaurus
Haestasaurus
Janenschia
Tehuelchesaurus
100
80
60
40
20
0
Millions of years before end-Cretaceous

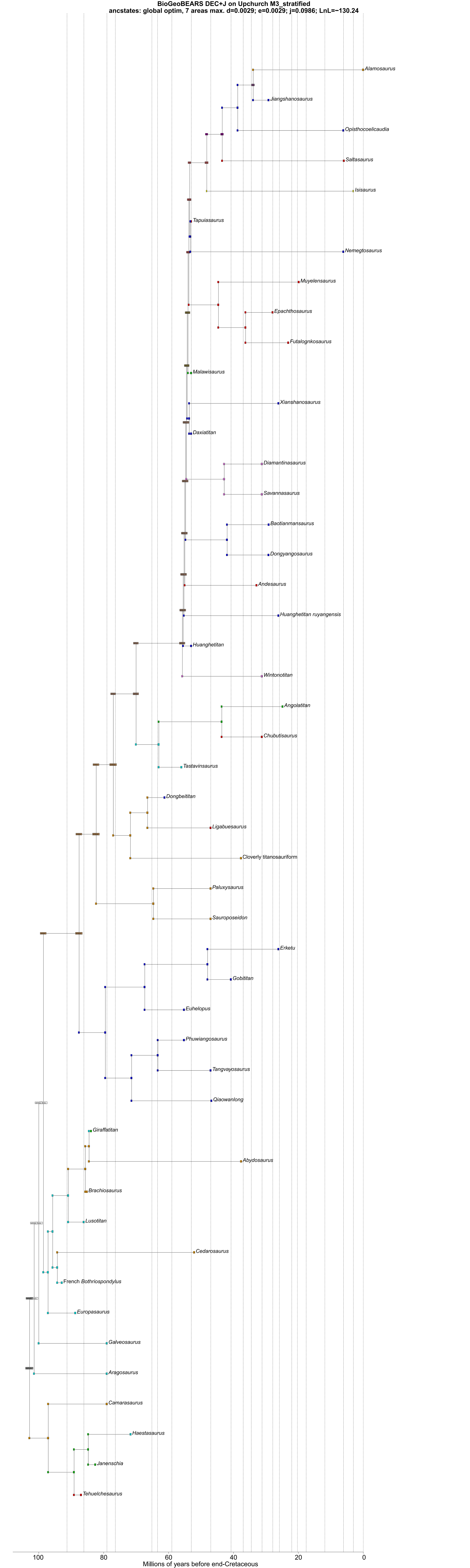
BioGeoBEARS DEC+J on Upchurch M3_stratified
ancstates: global optim, 7 areas max. d=0.0029; e=0.0029; j=0.0986; LnL=-130.24
Alamosaurus
Jiangshanosaurus
Opisthocoelicaudia
Saltasaurus
Isisaurus
Tapuiasaurus
Nemegtosaurus
Muyelensaurus
Epachthosaurus
Futalognkosaurus
Malawisaurus
Xianshanosaurus
Daxiatitan
Diamantinasaurus
Savannasaurus
Baotianmansaurus
Dongyangosaurus
Andesaurus
Huanghetitan ruyangensis
Huanghetitan
Wintonotitan
Angolatitan
Chubutisaurus
Tastavinsaurus
Dongbeititan
Ligabuesaurus
Cloverly titanosauriform
Paluxysaurus
Sauroposeidon
Erketu
Gobititan
Euhelopus
Phuwiangosaurus
Tangvayosaurus
Qiaowanlong
Giraffatitan
Abydosaurus
Brachiosaurus
Lusotitan
Cedarosaurus
French Bothriospondylus
Europasaurus
Galveosaurus
Aragosaurus
Camarasaurus
Haestasaurus
Janenschia
Tehuelchesaurus
100
80
60
40
20
0
Millions of years before end-Cretaceous

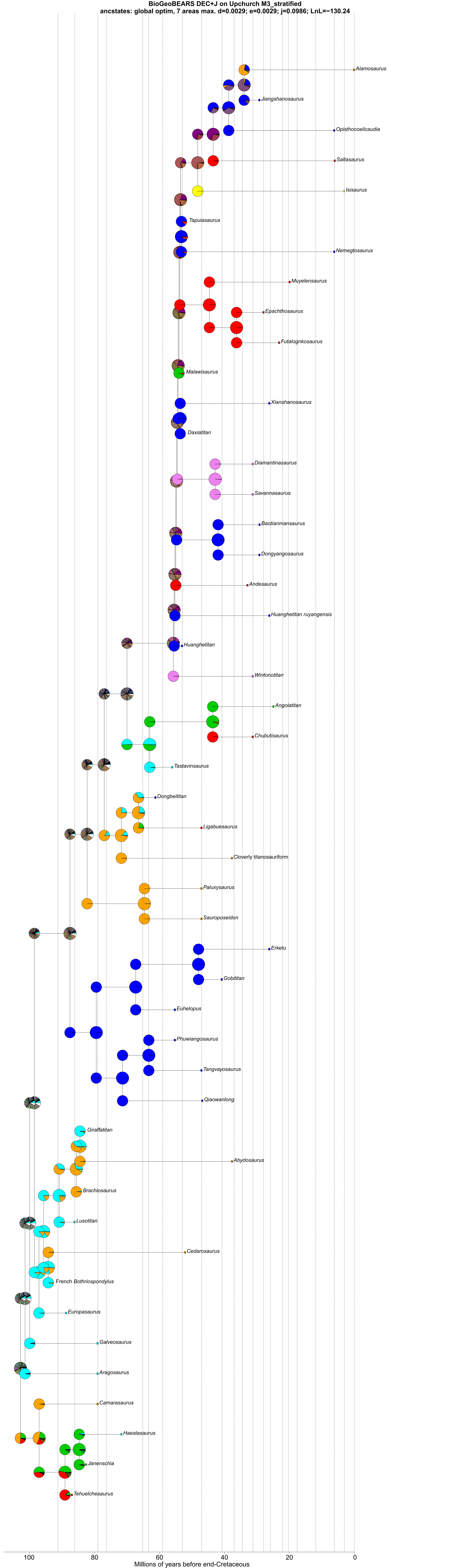
BioGeoBEARS DEC+J on Upchurch M3_stratified
ancstates: global optim, 7 areas max. d=0.0029; e=0.0029; j=0.0986; LnL=-130.24
Alamosaurus
Jiangshanosaurus
Opisthocoelicaudia
Saltasaurus
Isisaurus
Tapuiasaurus
Nemegtosaurus
Muyelensaurus
Epachthosaurus
Futalognkosaurus
Malawisaurus
Xianshanosaurus
Daxiatitan
Diamantinasaurus
Savannasaurus
Baotianmansaurus
Dongyangosaurus
Andesaurus
Huanghetitan ruyangensis
Huanghetitan
Wintonotitan
Angolatitan
Chubutisaurus
Tastavinsaurus
Dongbeititan
Ligabuesaurus
Cloverly titanosauriform
Paluxysaurus
Sauroposeidon
Erketu
Gobititan
Euhelopus
Phuwiangosaurus
Tangvayosaurus
Qiaowanlong
Giraffatitan
Abydosaurus
Brachiosaurus
Lusotitan
Cedarosaurus
French Bothriospondylus
Europasaurus
Galveosaurus
Aragosaurus
Camarasaurus
Haestasaurus
Janenschia
Tehuelchesaurus
100
80
60
40
20
0
Millions of years before end-Cretaceous

BioGeoBEARS BAYAREALIKE on Upchurch M3_stratified
ancstates: global optim, 7 areas max. d=0.0034; e=0.0198; j=0; LnL=-132.06

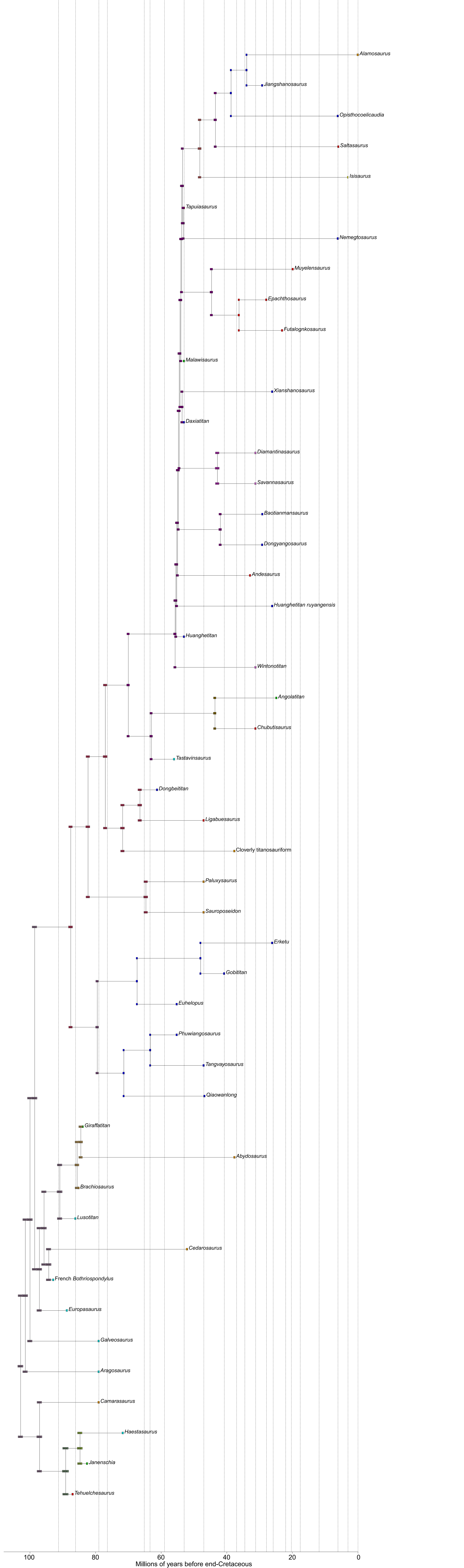

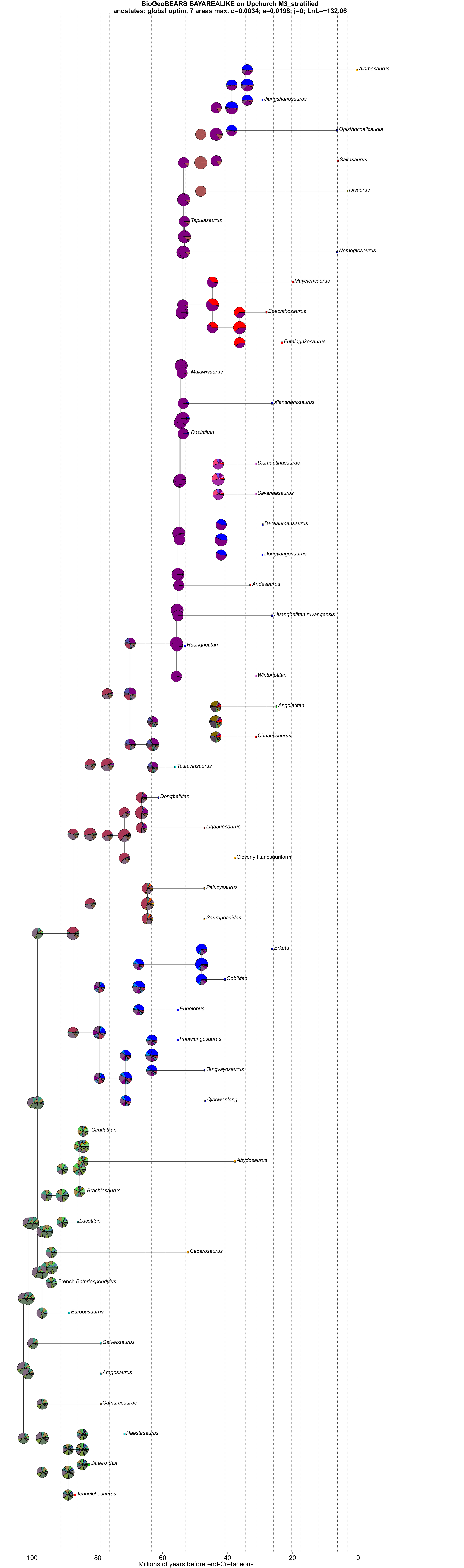
BioGeoBEARS BAYAREALIKE on Upchurch M3_stratified
ancstates: global optim, 7 areas max. d=0.0034; e=0.0198; j=0; LnL=-132.06
Alamosaurus
Jiangshanosaurus
Opisthocoelicaudia
Saltasaurus
Isisaurus
Tapuiasaurus
Nemegtosaurus
Muyelensaurus
Epachthosaurus
Futalognkosaurus
Malawisaurus
Xianshanosaurus
Daxiatitan
Diamantinasaurus
Savannasaurus
Baotianmansaurus
Dongyangosaurus
Andesaurus
Huanghetitan ruyangensis
Huanghetitan
Wintonotitan
Angolatitan
Chubutisaurus
Tastavinsaurus
Dongbeititan
Ligabuesaurus
Cloverly titanosauriform
Paluxysaurus
Sauroposeidon
Erketu
Gobititan
Euhelopus
Phuwiangosaurus
Tangvayosaurus
Qiaowanlong
Giraffatitan
Abydosaurus
Brachiosaurus
Lusotitan
Cedarosaurus
French Bothriospondylus
Europasaurus
Galveosaurus
Aragosaurus
Camarasaurus
Haestasaurus
Janenschia
Tehuelchesaurus
100
80
60
40
20
0
Millions of years before end-Cretaceous

BioGeoBEARS BAYAREALIKE+J on Upchurch M3_stratified
ancstates: global optim, 7 areas max. d=0.0019; e=0.0175; j=0.0303; LnL=−130.98

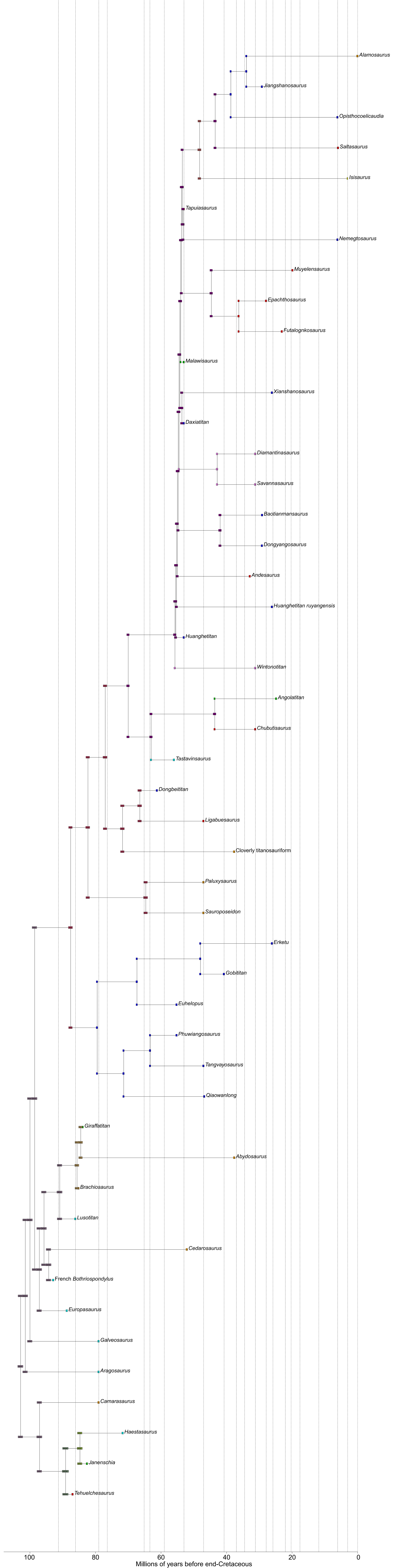

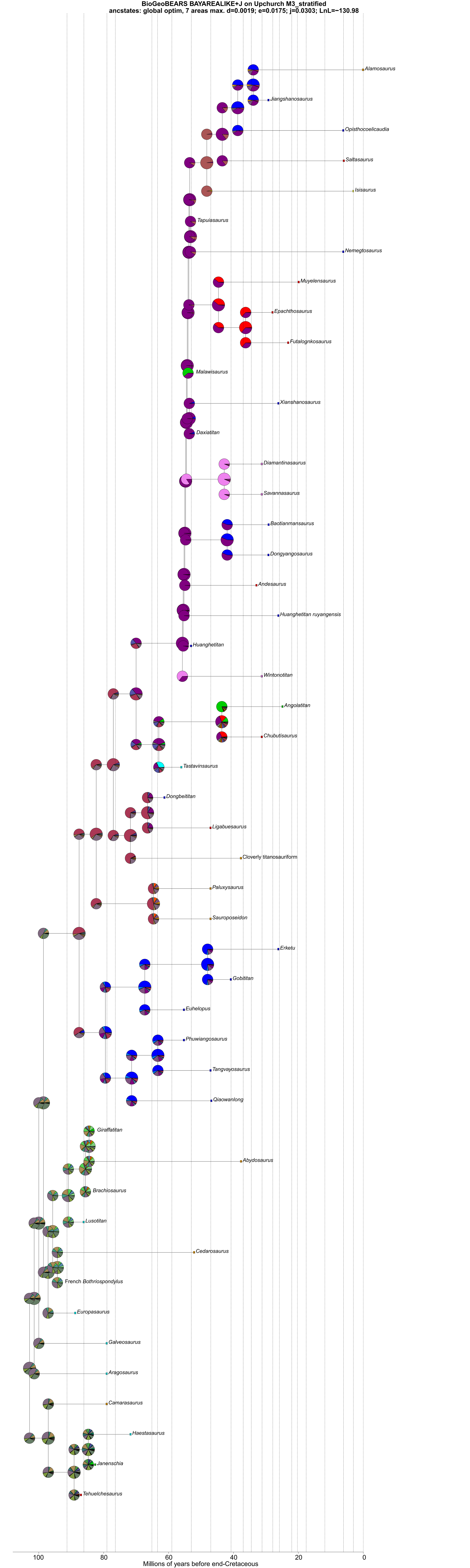
BioGeoBEARS BAYAREALIKE+J on Upchurch M3_stratified
ancstates: global optim, 7 areas max. d=0.0019; e=0.0175; j=0.0303; LnL=−130.98
Alamosaurus
Jiangshanosaurus
Opisthocoelicaudia
Saltasaurus
Isisaurus
Tapuiasaurus
Nemegtosaurus
Muyelensaurus
Epachthosaurus
Futalognkosaurus
Malawisaurus
Xianshanosaurus
Daxiatitan
Diamantinasaurus
Savannasaurus
Baotianmansaurus
Dongyangosaurus
Andesaurus
Huanghetitan ruyangensis
Huanghetitan
Wintonotitan
Angolatitan
Chubutisaurus
Tastavinsaurus
Dongbeititan
Ligabuesaurus
Cloverly titanosauriform
Paluxysaurus
Sauroposeidon
Erketu
Gobititan
Euhelopus
Phuwiangosaurus
Tangvayosaurus
Qiaowanlong
Giraffatitan
Abydosaurus
Brachiosaurus
Lusotitan
Cedarosaurus
French Bothriospondylus
Europasaurus
Galveosaurus
Aragosaurus
Camarasaurus
Haestasaurus
Janenschia
Tehuelchesaurus
100
80
60
40
20
0
Millions of years before end-Cretaceous
